# Metal‐Free Hydrosilylation of Ketenes with Silicon Electrophiles: Access to Fully Substituted Aldehyde‐Derived Silyl Enol Ethers

**DOI:** 10.1002/chem.202100877

**Published:** 2021-05-06

**Authors:** Avijit Roy, Martin Oestreich

**Affiliations:** ^1^ Institut für Chemie Technische Universität Berlin Straße des 17. Juni 115 10623 Berlin Germany

**Keywords:** boron, hydrosilylation, Lewis acids, silicon, silylium ions

## Abstract

Little‐explored hydrosilylation of ketenes promoted by main‐group catalysts is reported. The boron Lewis acid tris(pentafluorophenyl)borane accelerates the slow uncatalyzed reaction of ketenes and hydrosilanes, thereby providing a convenient access to the new class of β,β‐di‐ and β‐monoaryl‐substituted aldehyde‐derived silyl enol ethers. Yields are moderate to high, and *Z* configuration is preferred. The corresponding silyl bis‐enol ethers are also available when using dihydrosilanes. The related trityl‐cation‐initiated hydrosilylation involving self‐regeneration of silylium ions is far less effective.

## Graphical abstract

**β,β‐Di‐ and β‐monoaryl‐substituted silyl enol ethers** derived from aldehydes are difficult to prepare. This rare class of compounds has been made accessible by a transition‐metal‐free hydrosilylation of ketenes with B(C_6_F_5_)_3_ as catalyst and Et_3_SiH as stoichiometric reductant. The substrate scope is decent, and the stereoselectivity moderate to good in selected cases.

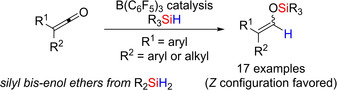

Little is known about the hydrosilylation of ketenes despite the direct formation of otherwise difficult to prepare aldehyde‐derived silyl enol ethers.^[1]^ These are synthetically valuable building blocks to access α‐branched aldehydes. Aside from an earlier report in the Russian literature,^[2]^ it was Frainnet and Caussé to disclose platinum‐ and nickel‐catalyzed protocols for the hydrosilylation of a small set of ketenes (Scheme 1, top).^[3]^ An uncatalyzed addition of hydrosilanes across ketenes also traces back to those authors, yet no details were reported.^[4]^ Decades later, Olah and co‐workers investigated the Lewis pair formation of ketenes and trialkylsilylium ions.^[5]^ For disubstituted ketenes, quantum‐chemical calculations predicted the formation of the O‐adduct to be energetically favorable over the C‐adduct. However, the stoichiometric reaction of these ketenes and trialkylsilylium ions selectively furnished the C‐adduct at cryogenic temperature (Scheme 1, middle). A silylium‐ion‐promoted ketene hydrosilylation relying on the strategy of self regeneration of the silylium ion^[6]^ was not described, perhaps because of the unexpected existence the C‐adduct in solution. To develop a catalytic process based the generation of silicon electrophiles, we seeked to examine the trityl‐cation‐initiated, silylium‐ion‐promoted hydrosilylation of ketenes (Scheme 1, bottom). As an alternative to that approach, we also probed B(C_6_F_5_)_3_/hydrosilane combinations to achieve a Piers‐type ketene hydrosilylation (Scheme 1, bottom).^[7]^ The key difference between these methods lies in the hydride source, being the hydrosilane in the former and the in‐situ‐generated borohydride in the latter system. Herein, we report the hydrosilylation of ketenes promoted by main‐group Lewis acid catalysts to access fully substituted aldehyde‐derived silyl enol ethers with at least one aryl substituent.

**Scheme 1:**
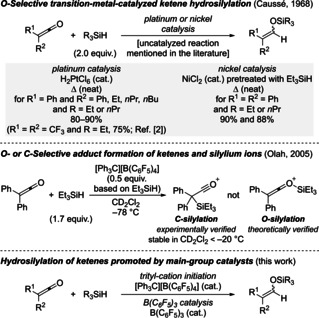
Transition‐metal‐catalyzed hydrosilylation of ketenes and Lewis pair formation of ketenes and silylium ions leading towards Lewis‐acid‐promoted ketene hydrosilylation.

To countercheck the unverified mention of an uncatalyzed reaction between ketenes and hydrosilanes,^[4]^ a blank reaction was run with diphenylketene (**1 a**) and 1.2 equiv. of Et_3_SiH (**2 a**) in C_6_H_5_F at room temperature (Table 1, entry 1). The silyl enol ether **3 aa** did form in 17 % yield after 24 h. No reaction occurred when using catalytic amounts of the trityl cation [Ph_3_C][B(C_6_F_5_)_4_] as an initiator in CH_2_Cl_2_ at −78 °C (Olah's setup; entry 2). The yield of **3 aa** remained at the level of the uncatalyzed reaction in arene solvents (entries 3–5; see Table S1 in the Supporting Information). A higher yield of 45 % was obtained only when benzene was used as solvent (entry 6). This led us to conclude that the silylium‐ion‐promoted hydrosilylation of ketenes is possible but not efficient. Conversely, 2.0 mol % of B(C_6_F_5_)_3_ and 4.0 equiv. of hydrosilane **2 a** in C_6_H_5_F afforded the desired silyl enol ether **3 aa** in 75 % yield (entry 7). However, excess Et_3_SiH caused the formation of a large amount of (Et_3_Si)_2_O, rendering isolation and purification of **3 aa** problematic. To address this issue, the reaction was optimized with lower amount of the hydrosilane (1.2 equiv.) and higher catalyst loading (5.0 mol %). Under these reaction conditions, the yield of **3 aa** did improve to 90 % after maintaining the reaction at room temperature for 12 h (entry 8). Lower yields were obtained with less hydrosilane, decreased concentration of the reactants, and at elevated reaction temperature (entries 9–11).

**Table 1:** Selected examples for optimization of main‐group Lewis acid‐promoted hydrosilylation of diphenylketene.

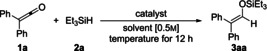					
Entry^[a]^	Catalyst [mol %]	Et_3_SiH [equiv.]	Solvent [0.5 M]	Temp. [°C]	Yield [%]^[b]^
1^[c]^	–	1.2	C_6_H_5_F	RT	17
2	[Ph_3_C][B(C_6_F_5_)_4_] (5.0)	1.5	CH_2_Cl_2_	−78	trace
3^[d]^	[Ph_3_C][B(C_6_F_5_)_4_] (2.0)	1.2	Toluene‐*d*_8_	−78	17
4	[Ph_3_C][B(C_6_F_5_)_4_] (2.0)	1.2	Toluene‐*d*_8_	RT	15
5	[Ph_3_C][B(C_6_F_5_)_4_] (2.0)	1.2	C_6_H_5_F	RT	17
6	[Ph_3_C][B(C_6_F_5_)_4_] (2.0)	1.2	C_6_H_6_	RT	45
7	B(C_6_F_5_)_3_ (2.0)	4.0	C_6_H_5_F	RT	75
8	B(C_6_F_5_)_3_ (5.0)	1.2	C_6_H_5_F	RT	90
9	B(C_6_F_5_)_3_ (5.0)	1.0	C_6_H_5_F	RT	76
10	B(C_6_F_5_)_3_ (5.0)	1.2	C_6_H_5_F	70	73
11^[e]^	B(C_6_F_5_)_3_ (5.0)	1.2	C_6_H_5_F	RT	78

[a] All reactions were performed on a 0.10–0.20 mmol scale. [b] Yield determined by ^1^H NMR spectroscopy with mesitylene as an internal standard. [c] For 24 h. [d] Performed in a J‐Young tube under argon atmosphere. After addition of all reactants at −78 °C, the mixture was stirred at RT for 12 h. [e] Performed at 0.25 M.

During the optimization of the reaction, trace amounts of the corresponding silyl ether were detected (not shown). This is believed to originate from the hydrosilylation of the acid chloride introduced with ketene **1 a**. Ketene formation was found to be generally slow, affording the ketene as a mixture with unreacted acid chloride. Most of the distillable disubstituted ketenes could be purified with the exception of ketenes **1 f**, **1 h**, and **1 i**. As for silyl enol ether **3 aa**, the combined isolated yields of the silyl enol ether and the silyl ether are reported for these transformations. The purity of the ketene was important, and attempts to start directly from the acid chloride followed by the hydrosilylation in the same pot or after simple filtration were unsuccessful (see the Supporting Information for procedures).

With an optimized procedure in hand, we began to investigate the scope for model substrate **1 a** and alkylaryl‐substituted ketene **1 f** with various hydrosilanes (Table 2); monosubstituted ketenes such as phenylketene were not included because of their strong tendency to undergo self‐reaction. With **1 a**, the reactions with tertiary hydrosilanes Et_3_SiH (**2 a**), *n*Bu_3_SiH (**2 b**), and Me_2_PhSiH (**2 c**) proceeded smoothly to provide products **3 aa**‐**ac** in good yields (entries 1, 3, and 5). Sterically more hindered *t*BuMe_2_SiH (**2 d**) and *i*Pr_3_SiH (**2 e**) led to lower yield (as for **3 ad**; entry 7) or little conversion (as for **3 ae**; entry 9). Yields were generally lower for ketene **1 f** but the influence of the steric demand of the hydrosilane was less pronounced (entries 2, 4, 6, and 8). Silyl enol ethers **3 fa**–**fc** formed with moderate *Z* selectivity while *E* configuration was preferred in the case of **3 fd**.

**Table 2:** Scope I: Variation of the hydrosilane.

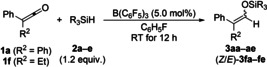				
Entry^[a]^	Ketene	Hydrosilane	*Z*/*E* ratio of **3 f** ^[b]^	Yield of **3 a** or **3 f** [%]^[c]^
1	**1 a**	Et_3_SiH (**2 a**)	–	**3 aa**: 84 (94)
2	**1 f**	Et_3_SiH (**2 a**)	86 : 14	**3 fa**: 51 (67)^[d]^
3	**1 a**	*n*Bu_3_SiH (**2 b**)	–	**3 ab**: 82 (94)
4	**1 f**	*n*Bu_3_SiH (**2 b**)	84 : 16	**3 fb**: 78 (74)^[d]^
5	**1 a**	Me_2_PhSiH (**2 c**)	–	**3 ac**: ‐^[e]^ (98)
6	**1 f**	Me_2_PhSiH (**2 c**)	62 : 38	**3 fc**: ‐^[e]^ (89)
7	**1 a**	*t*BuMe_2_SiH (**2 d**)	–	**3 ad**: 56 (67)
8	**1 f**	*t*BuMe_2_SiH (**2 d**)	31 : 69	**3 fd**: 70 (68)^[d]^
9	**1 a**	*i*Pr_3_SiH (**2 e**)	–	**3 ae**: ‐(8)

[a] All reactions were performed on a 0.20 mmol scale. [b] *Z*/*E*‐ratio was determined by ^1^H NMR analysis of the crude reaction mixture. [c] Unless otherwise noted, yields are isolated yield of silyl enol ethers (in parentheses yields determined by ^1^H NMR spectroscopy with CH_2_Br_2_ as an internal standard). [d] Combined isolated yield of **3 f** and the corresponding silyl ether. [e] Silyl enol ether decomposed during column chromatography on alumina.

We continued exploring the scope of diaryl‐ and alkylaryl‐substituted ketenes using Et_3_SiH (**2 a**) under the standard protocol (cf. Table 1, entry 8). Extension to other diarylketenes was possible but limited due to the difficulty in their synthesis and isolation in analytically pure form (Scheme 2). A gram‐scale synthesis of **3 aa** from **1 a** with lower catalyst loading (2.0 mol %) brought about 84 % isolated yield. Because of their low polarity and lack of stability during column chromatography on alumina, the yields of silyl enol ethers **3 aa**–**ea** were determined by ^1^H NMR spectroscopy with an internal standard. Sterically demanding aryl groups such as α‐naphthyl were detrimental; the yield of 39 % was improved to 67 % at 10 mol % catalyst loading. No conversion was achieved with mesityl groups (not shown).

**Scheme 2:**
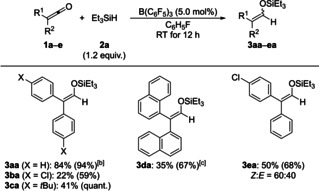
Scope II: B(C_6_F_5_)_3_‐catalyzed hydrosilylation of diaryl‐substituted ketenes. All reactions were performed on a 0.20–0.40 mmol scale. Unless otherwise noted, yields are isolated yield of silyl enol ethers (in parentheses yields determined by ^1^H NMR spectroscopy with CH_2_Br_2_ as an internal standard). [b] Isolated yield was 84 % on a gram scale (1.3 g). [c] With 10 mol % of B(C_6_F_5_)_3_.

As previously seen for ketene **1 f** (see Table 2), silyl enol ethers derived from other alkylaryl‐substituted ketenes formed with moderate to good *Z* selectivity (**1 f**–**q**→**3fa**–**qa**; Scheme 3). The best stereoselectivity of *Z : E*=93 : 7 was obtained for a cyclopentyl group as the alkyl substituent (**1 i**→**3ia**). Both the alkyl group (top) and the substituent on the aryl group (bottom) were modified, including Ibuprofen‐derived **1 j** (gray box). Electron‐withdrawing and electron‐donating groups at the aryl substituent were tolerated. Again, the bulky α‐naphthyl group required a higher catalyst loading (**1 q**→**3qa**): 77 % with 10 mol % of B(C_6_F_5_)_3_ versus 34 % with 5.0 mol % of B(C_6_F_5_)_3_.

**Scheme 3:**
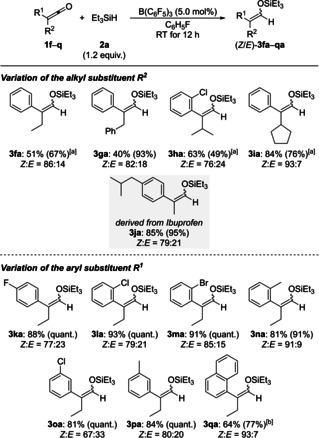
Scope III: B(C_6_F_5_)_3_‐catalyzed hydrosilylation of alkylaryl‐substituted ketenes. All reactions were performed on a 0.20–0.40 mmol scale. Unless otherwise noted, yields are isolated yield of silyl enol ethers (in parentheses yields determined by ^1^H NMR spectroscopy with CH_2_Br_2_ as an internal standard). [a] Combined isolated yield of silyl enol ether and silyl ether. [b] With 10 mol % of B(C_6_F_5_)_3_.

Mironov and co‐workers had already accomplished the platinum‐catalyzed addition of tetramethyldisiloxane across two molecules of perfluorinated dimethylketene.^[2]^ The new protocol could be extended to the same two‐fold addition (Scheme 4). With 1.0 equiv. of dihydrosilane Et_2_SiH_2_ (**2 f**), bis‐hydrosilylation of **1 a** and **1 c** proceeded in acceptable yields; the silyl bis‐enol ethers **4 af** and **4 cf** were isolated in 43 % and 50 %, respectively. Silyl bis‐enol ethers are attractive precursors for diastereoselective oxidative coupling homo‐ and cross‐coupling.^[8]^

**Scheme 4:**
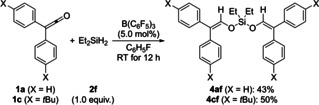
Scope IV: B(C_6_F_5_)_3_‐catalyzed bis‐hydrosilylation of diaryl‐substituted ketenes. Reactions were performed on a 0.20 mmol scale. Yields are isolated yield of the silyl bis‐enol ethers.

The assumed C=O hydrosilylation was confirmed by a deuterium‐labeling experiment with Et_3_SiD (**2 a**‐*d*_1_) to yield the α‐deuterated silyl enol ether (**1 a**→**3aa**‐*d*_1_; Scheme 5, top). The catalytic cycle is thought to follow the general Piers mechanism of B(C_6_F_5_)_3_‐mediated Si−H bond activation, S_N_Si substitution by the Lewis‐basic substrate, and subsequent borohydride reduction of the silylated onium‐ion intermediate (Scheme 5, bottom).[^7b^, ^9^] Hence, the ketene oxygen atom is the nucleophile. The thus‐formed O‐adduct is then reduced by [HB(C_6_F_5_)_3_]^−^ to give the silyl enol ether. This is different from Olah's system where treatment of ketenes with silylium ions leads to the stable C‐adducts (see Scheme 1, middle).^[5]^ These C‐adducts are reluctant to accept hydride from excess hydrosilane as experimentally verified by us (cf. Table 1, entries 2–6). We think that the ketene β‐carbon atom is sterically not accessible for the B(C_6_F_5_)_3_/hydrosilane pair and, hence, cannot act as a nucleophile in the B(C_6_F_5_)_3_ catalysis. The *Z* selectivity, although moderate, is set in the hydride transfer to the in‐plane LUMO of the C=O double bond of the O‐adduct from the sterically less hindered side.^[10]^ For completion, the uncatalyzed hydrosilylation of ketenes with unactivated hydrosilanes (see Table 1, entry 1)^[4]^ can be described by a four‐membered transition state involving the C=O and Si−H bonds (not shown).

**Scheme 5:**
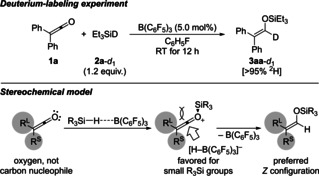
Control experiment and stereochemical model.

In conclusion, we showed here that the main‐group Lewis acid B(C_6_F_5_)_3_ catalyzes the hydrosilylation of ketenes with Et_3_SiH as the stoichiometric reductant. This mild transition‐metal‐free procedure enables the synthesis of a new class of β,β‐di‐ and β‐monoaryl‐substituted silyl enol ethers derived from aldehydes in decent yields and with moderate *Z* selectivity. Future work will be directed towards the assessment of their reactivity and their use in stereoselective synthesis.^[1]^

## Conflict of interest

The authors declare no conflict of interest.

## Supporting information

As a service to our authors and readers, this journal provides supporting information supplied by the authors. Such materials are peer reviewed and may be re‐organized for online delivery, but are not copy‐edited or typeset. Technical support issues arising from supporting information (other than missing files) should be addressed to the authors.

Supplementary
