# Supplementary material for: Metal‐Free Hydrosilylation of Ketenes with Silicon Electrophiles: Access to Fully Substituted Aldehyde‐Derived Silyl Enol Ethers
Source: Chemistry. 2021 May 6;27(32):8273–6. doi: 10.1002/chem.202100877 (PMC8251732; doi:10.1002/chem.202100877)
Supplement: Supplementary file 1 — Supplementary [file CHEM-27-8273-s001.pdf]

Chemistry–A European Journal

Supporting Information

Metal-Free Hydrosilylation of Ketenes with Silicon Electrophiles: Access to Fully Substituted Aldehyde-Derived Silyl Enol Ethers

Avijit Roy and Martin Oestreich*

Table of Contents

**1**	**General Information**	**S2**
**2**	**Optimization of the Hydrosilylation of Ketenes**	**S3**
**3**	**Experimental Details for the Synthesis of α,α-Disubstituted Acetic Acids**	**S6**
3.1	Synthesis of the Non-Commercial α,α-Diarylacetic Acids	S6
3.2	Synthesis of the α-Alkyl-α-arylacetic Acids	S10
**4**	**Experimental Details for the Synthesis of α,α-Disubstituted Acetyl Chlorides**	**S12**
**5**	**Experimental Details for the Synthesis of Ketenes**	**S13**
5.1	General Procedure for the Synthesis of Diarylketenes	S13
5.2	General Procedure for the Synthesis of Alkylarylketenes	S16
**6**	**Experimental Details for the Hydrosilylation of Ketenes with $B(C_6F_5)_3$**	**S18**
6.1	General Procedure for the Hydrosilylation of Ketenes with $B(C_6F_5)_3$	S18
6.2	Characterization Data for the Hydrosilylation of Ketenes with $B(C_6F_5)_3$	S19
**7**	**Gram-Scale Synthesis of Silyl Enol Ether 3aa**	**S36**
**8**	**Experimental Details for the Attempted One-Pot Sequence**	**S37**
**9**	**NMR Spectra**	**S38**
**10**	**References**	**S133**

1 General Information

All reactions were performed in flame-dried glassware using an *MBraun* glovebox or conventional Schlenk techniques under a static pressure of argon (glovebox) or nitrogen (fume hood) unless otherwise stated. Standard solvents and reagents were obtained from commercial suppliers and used as received unless otherwise stated. Technical grade solvents for extraction or chromatography (CH_2Cl_2, cyclohexane, Et_2O, and *n*-pentane) were distilled prior to use. Et_2O and THF were dried over potassium/benzophenone and freshly distilled prior to use. All hydrosilanes, dihydrosilanes as well as fluorobenzene (C_6H_5F) were dried over CaH_2, distilled, degassed by three freeze-pump-thaw cycles, and stored in a glovebox over thermally activated 4 Å molecular sieves. $B(C_6F_5)_3$ was purchased from Boulder Scientific Company, sublimed under vacuum at 85 °C prior to use, and stored in an argon-filled glovebox. ^{1}H, ^{13}C, ^{19}F, and ^{29}Si NMR spectra were recorded in C_6D_6, $CDCl_3$, or CD_2Cl_2 on a *Bruker* AV400 and AV500 instrument, respectively. Analytical thin-layer chromatography (TLC) was performed on silica gel 60 F254 glass plates. Flash column chromatography was performed on silica gel 60 (40–63 µm, 230–400 mesh, ASTM) and aluminium oxide 90 (standardized, activity II-III, 63–200 µm, 70–230 mesh, ASTM). Chemical shifts are reported in parts per million (ppm) and are referenced to the residual solvent resonance as the internal standard (C_6D_5H: δ 7.16 ppm for ^{1}H NMR and C_6D_6: δ 128.06 ppm for ^{13}C NMR; $CHCl_3$: δ 7.26 ppm for ^{1}H NMR and $CDCl_3$: δ 77.16 ppm for ^{13}C NMR; $CDCl_2H$: $\delta = 5.32$ ppm for ^{1}H NMR and CD_2Cl_2 $\delta = 53.84$ ppm for ^{13}C NMR). ^{19}F and ^{29}Si NMR spectra are referenced in compliance with the unified scale for NMR chemical shifts as recommended by the IUPAC stating the chemical shift relative to CCl_3F, and TMS, respectively.[1] Data are reported as follows: chemical shift, multiplicity (s = singlet, d = doublet, t = triplet, q = quartet, sept = septet, m = multiplet, m_c = centrosymmetric multiplet, br = broad signal), coupling constants (Hz) and integration. Infrared (IR) spectra were recorded on an *Agilent Technologies* Cary 630 instrument. For air-sensitive compound, IR spectra were recorded in a glovebox using a *Thermo* Nicolet Magna-IR 750 spectrophotometer equipped with an ATR unit. The signals are reported in wavenumbers (cm^{-1}). High resolution mass spectra (HRMS) were obtained from the *Laboratory of Mass Spectrometry* at the *Institut für Chemie*, *Technische Universität Berlin*.

2 Optimization of the Hydrosilylation of Ketenes

2.1 General Procedure for the Optimization of the Hydrosilylation of Ketenes with $[Ph_3C]^+[B(C_6F_5)_4]^-$ as Initiator

In an argon-filled glovebox, the trityl salt (2.0–5.0 mol%) was suspended in the desired solvent in a GC vial. Triethylsilane (**2a**, 1.2–1.5 equiv) was added dropwise by a microsyringe. After stirring at room temperature for ~1 min, the dark yellow color of the trityl salt's solution faded, indicating the formation of respective silylium ion. Then, the solution of diphenylketene (**1a**, 0.10–0.20 mmol, 1.0 equiv) in the indicated solvent was added dropwise at room temperature. The resulting mixture was maintained at this temperature for additional 12 h. After taking the reaction vessel out of the glovebox, the internal standard (mesitylene, 0.50 equiv) was added to the reaction mixture to determine the yield by ^1H NMR spectroscopy. In case of the reaction performed at –78 °C, the suspension of silylium ion in the indicated solvent was cooled to –78 °C, and the solution of diphenylketene (pre-cooled at –78 °C) was added dropwise by a cannula. After complete addition, the mixture was allowed to warm to room temperature.

2.2 General Procedure for the Optimization of the Hydrosilylation of Ketenes with $B(C_6F_5)_3$ as Catalyst

In an argon-filled glovebox, $B(C_6F_5)_3$ (2.0–5.0 mol%) was dissolved in the desired solvent in a GC vial. The solution of diphenylketene (**1a**, 0.10 mmol, 1.0 equiv) in the same solvent was added dropwise at room temperature. Triethylsilane (**2a**, 1.0–4.0 equiv) was then added dropwise by a microsyringe. The resulting mixture was maintained at this temperature for additional 12 h. After taking the reaction vessel out of the glovebox, the internal standard (mesitylene, 0.50 equiv) was added to the reaction mixture to determine the yield by ^1H NMR spectroscopy.

Table S1: Detailed Optimization of the Hydrosilylation of Diphenylketene (**1a**) with Et_3SiH using $[Ph_3C]^+[B(C_6F_5)_4]^-$ as Initiator

Entry[a]	$[Ph_3C]^+[B(C_6F_5)_4]^-$ [mol%]	Et_3SiH [equiv]	Solvent [0.5M]	Temp [°C]	Yield [%][b]
1	5.0	1.5	CH_2Cl_2	–78 °C	trace
2[c]	2.0	1.2	Toluene-d_8	–78 °C	17
3[c]	2.0	1.2	Toluene-d_8	RT	15
4	2.0	1.2	Toluene-d_8	RT	23
5	2.0	1.2	Toluene	RT	27
6	2.0	1.2	C_6H_6	RT	45
7	2.0	1.2	C_6H_5F	RT	17
8	2.0	1.2	C_6H_5Cl	RT	15
9	2.0	1.2	1,2-$C_6H_4Cl_2$	RT	13
10	2.0	1.2	Cyclohexane	RT	35

[a] All reactions were performed on a 0.20 mmol scale. [b] Yield determined by ^1H NMR spectroscopy with mesitylene as an internal standard. [c] Performed in a J-Young tube under argon atmosphere (without constant stirring).

Table S2: Detailed Optimization of the Hydrosilylation of Diphenylketene (**1a**) with Et_3SiH using $B(C_6F_5)_3$ as Catalyst

Entry[a]	$B(C_6F_5)_3$ [mol%]	Et_3SiH [equiv]	Solvent [0.5M]	Temp [°C]	Yield [%][b]
1	2.0	4.0	CH_2Cl_2	RT	84%
2	2.0	4.0	Toluene	RT	79%
3	2.0	4.0	C_6H_6	RT	80%
4	2.0	4.0	C_6H_5F	RT	75%
5	2.0	4.0	C_6H_5Cl	RT	82%
6	2.0	4.0	1,2-$C_6H_4Cl_2$	RT	78%
7	2.0	1.2	CH_2Cl_2	RT	84%
8	2.0	1.2	C_6H_5F	RT	85%
9	2.0	2.0	C_6H_5F	RT	86%
**10**	**5.0**	**1.2**	**C_6H_5F**	**RT**	**90%**
11	5.0	1.0	C_6H_5F	RT	76%
12	5.0	1.2	C_6H_5F	70 °C	73%
13[c]	5.0	1.2	C_6H_5F	RT	78%
14[d]	–	1.2	C_6H_5F	RT	17%

[a] All reactions were performed on a 0.10 mmol scale. [b] Yield determined by ^1H NMR spectroscopy with mesitylene as an internal standard. [c] Performed at 0.25 M. [d] For 24 h.

3 Experimental Details for the Synthesis of α,α-Disubstituted Acetic Acids

3.1 Synthesis of Non-Commercial α,α-Diarylacetic Acids

3.1.1 2,2-Bis(4-chlorophenyl)acetic acid

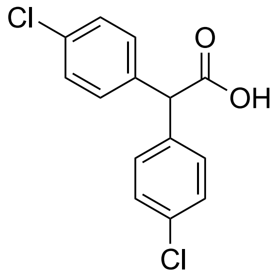

$C_{14}H_{10}Cl_2O_2$
M = 281.13 g/mol

Chlorobenzene (1.0 mL, 10 mmol, 2.0 equiv) was added to a solution of glyoxylic acid monohydrate (0.46 g, 5.0 mmol, 1.0 equiv) in AcOH (6 mL). The mixture was then cooled to 0 °C, conc. H_2SO_4 (5 mL) was added dropwise, and stirred at 80 °C for 12 h. After cooling to room temperature, the reaction mixture was quenched with water (10 mL) and a dark brown solid was precipitated. The solid was washed with water (2 × 10 mL) followed by *n*-hexane (3 × 10 mL). The yellowish solid can further be purified by trituration with *n*-hexane and EtOAc to get the desired acid (0.35 g, 25%) as white solid.

^{1}H NMR (400 MHz, $CDCl_3$, 298 K): δ = 4.99 (s, 1H), 7.22–7.25 (m, 4H), 7.30–7.33 (m, 4H) ppm.
^{13}C{^{1}H} NMR (101 MHz, $CDCl_3$, 298 K): δ = 55.5, 129.1, 130.1, 133.9, 136.0, 176.6 ppm.

3.1.2 2,2-Bis(4-(*tert*-butyl)phenyl)acetic acid

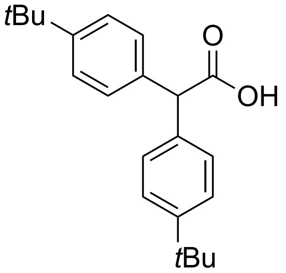

$C_{22}H_{28}O_2$
M = 324.46 g/mol

Tert-butylbenzene (6.2 mL, 40 mmol, 2.0 equiv) was added to a solution of glyoxylic acid monohydrate (1.8 g, 20 mmol, 1.0 equiv) in AcOH (24 mL). The mixture was then cooled to 0 °C, conc. H_2SO_4 (20 mL) was added dropwise, and stirred at 80 °C for 12 h. After cooling to room temperature, the reaction mixture was quenched with water (40 mL) and a dark brown solid was precipitated. The solid was washed with water (2 × 40 mL) followed by *n*-hexane (3

× 40 mL). The yellowish solid can further be purified by trituration with *n*-hexane and EtOAc to get the desired acid (3.8 g, 59%) as white solid.

^{1}H NMR (400 MHz, $CDCl_3$, 298 K): δ = 1.30 (s, 18H), 4.99 (s, 1H), 7.27 (d, *J* = 7.8 Hz, 4H), 7.34 (d, *J* = 8.5 Hz, 4H) ppm.
^{13}C{^{1}H} NMR (101 MHz, $CDCl_3$, 298 K): δ = 31.4, 34.6, 56.1, 125.7, 128.4, 135.2, 150.4, 177.7 ppm.

3.1.3 2-(4-Chlorophenyl)-2-phenylacetic acid

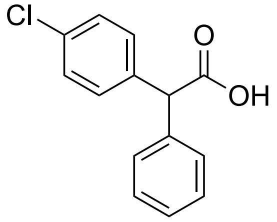

$C_{14}H_{11}ClO_2$
M = 246.69 g/mol

According to a literature reported procedure,[2] a mixture of (*R*)-(–)-mandelic acid (1.5 g, 10 mmol, 1.0 equiv) and chlorobenzene (6 ml) was heated at 70 °C. $SnCl_4$ (1.9 mL, 16 mmol, 1.6 equiv) was added dropwise into the hot mixture and then heated to reflux for 12 h. After cooling to room temperature, the solution is poured onto ice and extracted with $CHCl_3$ (2 × 15 mL). The combined extracts were washed with aq. HCl (1 N, 10 mL) and water (2 × 10 mL), dried over $MgSO_4$ and the solvent was removed in vacuum. Flash column chromatography using cyclohexane/ethyl acetate (9:1) afforded the desired acid (1.4 g, 57%) as white solid. The spectroscopic data were in accordance with those reported.[3]

^{1}H NMR (400 MHz, $CDCl_3$, 298 K): δ = 5.02 (s, 1H), 7.25–7.37 (m, 10H) ppm.
^{13}C{^{1}H} NMR (101 MHz, $CDCl_3$, 298 K): δ = 56.3, 127.9, 128.7, 128.9, 130.2, 133.7, 136.5, 137.6, 177.8 ppm.

3.1.4 2,2-Di(naphthalen-1-yl)acetic acid

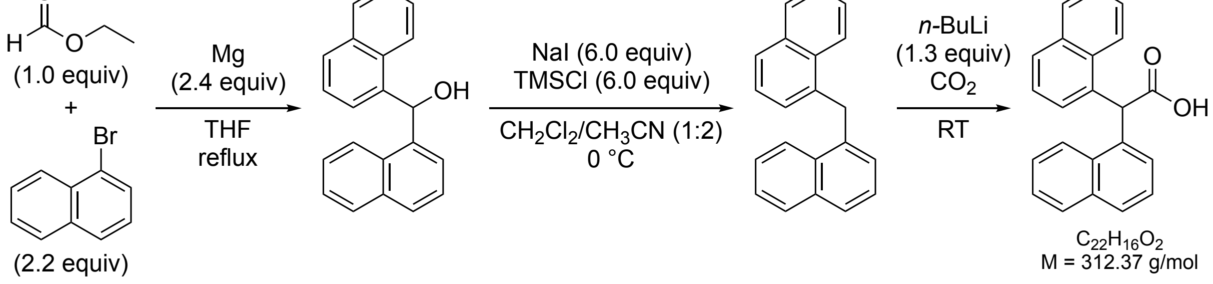

$C_{22}H_{16}O_2$
M = 312.37 g/mol

Di(naphthalen-1-yl)methanol: A solution of 1-bromonaphthalene (6.7 mL, 48 mmol, 2.2 equiv) in THF (10 mL) was added dropwise to a suspension of magnesium turnings (1.3 g, 53 mmol,

2.4 equiv) and iodine (one crystal) in THF (40 mL) at room temperature. After complete addition, the mixture was heated to 65 °C and stirred at this temperature for 2 h. The oil bath was removed and ethyl formate (1.8 mL, 22 mmol, 1.0 equiv) was added dropwise to the hot reaction mixture. After stirring at room temperature for 2 h, the reaction mixture was quenched by the addition of saturated aqueous NH_4Cl solution (30 mL). The phases were separated, and the aqueous phase was extracted with EtOAc (3 × 50 mL). The combined organic phases were dried over anhydrous $MgSO_4$, and the solvent was removed under reduced pressure. Purification by flash column chromatography on silica gel using cyclohexane/ethyl acetate (10:1) afforded the desired alcohol (5.8 g, 93%) as white solid.

^{1}H NMR (400 MHz, $CDCl_3$, 298 K): δ = 2.52 (s, 1H), 7.27 (br s, 1H), 7.53–7.38 (m, 8H), 7.84 (d, *J* = 7.9 Hz, 2H), 7.92 (dd, *J* = 8.3, 1.0 Hz, 2H), 8.03 (d, J = 8.5 Hz, 2H).
^{13}C{^{1}H} NMR (101 MHz, $CDCl_3$, 298 K): δ = 69.7, 123.8, 125.1, 125.6, 125.8, 126.6, 128.7, 129.0, 131.2, 134.0, 138.5 ppm.

Di(naphthalen-1-yl)methane: Under a nitrogen atmosphere, NaI (6.0 g, 40 mmol, 6.0 equiv) was suspended in CH_3CN (7 mL), and TMSCl (5.0 mL, 40 mmol, 6.0 equiv) was added. After stirring for 20 min, the reaction mixture was cooled to 0 °C, and a solution of di(naphthalen-1-yl)methanol (1.9 g, 6.7 mmol, 1.0 equiv) in CH_2Cl_2/CH_3CN (1:1 v/v, 14 mL) was added over 1 h by a syringe pump. After stirring for additional 30 min and the reaction mixture was allowed to warm to room temperature, and then slowly poured into cold aqueous NaOH solution (10% w/v), additional NaOH solution was added to adjust pH ~ 7. The phases were separated, and the aqueous phase was extracted with EtOAc (3 × 20 mL). The combined organic layers were washed with aqueous $Na_2S_2O_3$ (10% w/v, 10 mL) followed by water, and then dried over $MgSO_4$. After evaporation of solvent, crude product was purified by flash column chromatography on silica gel using cyclohexane/ethyl acetate (50:1) to afford the desired compound (1.7 g, 94%) as white solid.

^{1}H NMR (400 MHz, $CDCl_3$, 298 K): δ = 4.90 (s, 2H), 7.09 (dd, *J* = 7.1, 1.0 Hz, 2H), 7.35 (dd, *J* = 8.2, 7.1 Hz, 2H), 7.55–7.45 (m, 4H), 7.77 (d, *J* = 8.2 Hz, 2H), 7.95–7.88 (m, 2H), 8.08–8.01 (m, 2H).
^{13}C{^{1}H} NMR (101 MHz, $CDCl_3$, 298 K): δ = 35.8, 124.1, 125.8 (2C), 126.2, 127.2 (2C), 128.9, 133.9, 136.3 ppm.

2,2-Di(naphthalen-1-yl)acetic acid: To a stirred suspension of di(naphthalen-1-yl)methane (6.3 mmol, 1.7 g, 1.0 equiv) in THF (12 mL), under an atmosphere of nitrogen, *n*-BuLi (3.2 ml of a 2.5 M solution in hexane, 8.1 mmol, 1.3 equiv) was added at room temperature. After 1 h, dry CO_2 was bubbled through the reaction mixture and stirred for 12 h. The reaction mixture was quenched by the addition of aqueous NaOH solution (2 N, 20 mL) and stirred for 30 min. The

aqueous solution was washed with Et_2O and separated, acidified with conc. HCl (pH ~ 1) and extracted with EtOAc (3 × 30 mL). The combined organic layers were washed with water (10 mL), and then dried over $MgSO_4$. The EtOAc was removed by evaporation, which provided a white solid (1.8 g, 91% yield) that was used in the next step without further purification.

^{1}H NMR (400 MHz, $CDCl_3$, 298 K): δ = 6.55 (s, 1H), 7.34 (d, *J* = 7.0 Hz, 2H), 7.41 (t, *J* = 7.7 Hz, 2H), 7.46–7.53 (m, 4H), 7.84 (d, *J* = 8.1 Hz, 2H), 7.90–7.98 (m, 4H) ppm.
^{13}C{^{1}H} NMR (101 MHz, $CDCl_3$, 298 K): δ = 50.1, 123.1, 125.6, 126.0, 126.8, 127.0, 128.6, 129.2, 131.7, 133.5, 134.1, 178.6 ppm.

3.1.5 2,2-Dimesitylacetic acid

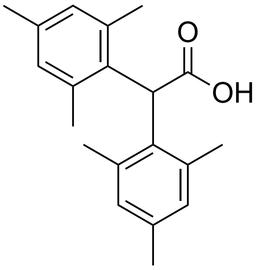

$C_{20}H_{24}O_2$
M = 296.41 g/mol

According to a literature reported procedure,[4] glyoxylic acid monohydrate (0.46 g, 5.0 mmol, 1.0 equiv) and silica sulfuric acid (0.46 g, 1 mass equivalent) were suspended in AcOH (2.2 mL). Mesitylene (4.9 mL, 35 mmol, 7.0 equiv) was then added dropwise at room temperature and the reaction mixture was heated to 120 °C for 12 h. After cooling to room temperature, the mixture was diluted with EtOAc (20 mL) and filtered, the solvent was removed in vacuum. Flash column chromatography using cyclohexane/ethyl acetate (4:1) afforded the desired acid (0.44 g, 30%) as white solid. The spectroscopic data were in accordance with those reported.[4]

^{1}H NMR (500 MHz, $CDCl_3$, 298 K): δ = 2.11 (s, 12H), 2.24 (s, 6H), 5.39 (s, 1H), 6.80 (s, 4H), 11.47 (br s, 1H) ppm.
^{13}C{^{1}H} NMR (126 MHz, $CDCl_3$, 298 K): δ = 20.8, 21.0, 50.8, 130.5, 132.3, 136.6, 137.4, 180.6 ppm.

3.2 Synthesis of α-Alkyl-α-arylacetic Acids

3.2.1 General Procedure 1 (GP 1)

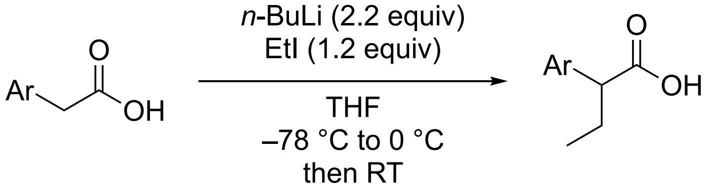

A flame-dried flask was charged with the α-arylacetic acid (10 mmol, 1.0 equiv) and dry THF (c = 0.35 M). The mixture was cooled to −78 °C and *n*-BuLi (8.8 ml of a 2.5 M solution in hexane, 22 mmol, 2.2 equiv) was added over 10 min. The solution was warmed to 0 °C and stirred for 2 h, and then ethyl iodide (1.0 mL, 12 mmol, 1.2 equiv) was added in one portion. The solution was allowed to warm to room temperature and stirred overnight. The reaction was quenched by the addition of water (20 mL), followed by the addition of conc. HCl (pH ~ 1) and extracted with EtOAc (3 × 30 mL). The combined organic layers were washed with aqueous $Na_2S_2O_3$ (10% w/v, 10 mL) followed by water, and then dried over $MgSO_4$. The EtOAc was removed by evaporation, which provided either yellowish liquid or white solid that was used in the next step without further purification.

2-Phenylbutanoic acid, 2-(*o*-tolyl)butanoic acid, 2-(*m*-tolyl)butanoic acid, and 2-(naphthalen-1-yl)butanoic acid were synthesized according to this general procedure.

3.2.2 General Procedure 2 (GP 2)

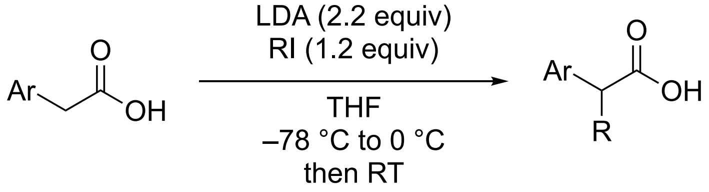

Under a nitrogen atmosphere, a solution of *n*-BuLi (13.2 mL of a 2.5 M solution in hexane, 33 mmol, 2.2 equiv) was added over 5 min to a solution of diisopropylamine (4.7 mL, 33 mmol, 2.2 equiv) in dry THF (c = 1.0 M) at −78 °C. The mixture was stirred at −78 °C for 30 min. Then, a solution of the α-arylacetic acid (15 mmol, 1.0 equiv) in THF (10 mL) was added over 5 min. The solution was warmed to 0 °C and stirred for 1 h, and then alkyl iodide (33 mmol, 2.2 equiv) was added in one portion. The solution was allowed to warm to room temperature and stirred overnight. The reaction was quenched by the addition of water (30 mL), followed by the addition of conc. HCl (pH ~ 1) and extracted with EtOAc (3 × 40 mL). The combined organic layers were washed with aqueous $Na_2S_2O_3$ (10% w/v, 20 mL) followed by water, and then dried

over $MgSO_4$. The EtOAc was removed by evaporation, which provided either yellowish liquid or white solid that was used in the next step without further purification.

2,3-Diphenylpropanoic acid, 2-(2-chlorophenyl)-3-methylbutanoic acid, 2-cyclopentyl-2-phenylacetic acid, 2-(4-fluorophenyl)butanoic acid, 2-(2-chlorophenyl)butanoic acid, 2-(2-bromophenyl)butanoic acid, and 2-(3-chlorophenyl)butanoic acid were synthesized according to this general procedure.

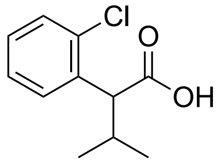

$C_{11}H_{13}ClO_2$
M = 212.67 g/mol

2-(2-Chlorophenyl)-3-methylbutanoic acid: Prepared from 2-(2-chlorophenyl)acetic acid (3.4 g, 20 mmol, 1.0 equiv) according to **GP 2**. The product was obtained as yellowish solid which was directly used in the next step without further purification.

^{1}H NMR (400 MHz, $CDCl_3$, 298 K): δ = 0.75 (d, *J* = 6.7 Hz, 3H), 1.13 (d, *J* = 6.5 Hz, 3H), 2.33 (m_c, 1H), 3.98 (d, *J* = 10.5 Hz, 1H), 7.17–7.21 (m, 1H), 7.23–7.27 (m, 1H), 7.39 (dd, *J* = 7.9, 1.7 Hz, 1H), 7.53 (dd, *J* = 7.7, 1.8 Hz, 1H), 11.57 (br s, 1H) ppm.
^{13}C{^{1}H} NMR (101 MHz, $CDCl_3$, 298 K): δ = 19.7, 21.4, 32.2, 54.2, 127.2, 128.6, 129.1, 129.7, 134.9, 135.6, 179.5 ppm.

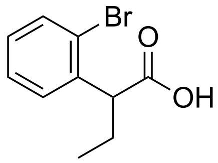

$C_{10}H_{11}BrO_2$
M = 243.10 g/mol

2-(2-Bromophenyl)butanoic acid: Prepared from 2-(2-bromophenyl)acetic acid (2.1 g, 10 mmol, 1.0 equiv) according to **GP 2**. The product was obtained as yellowish liquid which was directly used in the next step without further purification.

^{1}H NMR (500 MHz, $CDCl_3$, 298 K): δ = 0.94 (t, *J* = 7.4 Hz, 3H), 1.89–1.78 (m, 1H), 2.16–2.06 (m, 1H), 4.14 (t, *J* = 7.5 Hz, 1H), 7.13 (ddd, *J* = 7.9, 7.4, 1.7 Hz, 1H), 7.30 (td, *J* = 7.5, 1.3 Hz, 1H), 7.38 (dd, *J* = 7.8, 1.7 Hz, 1H), 7.58 (dd, *J* = 8.0, 1.3 Hz, 1H), 11.16 (br s, 1H).
^{13}C{^{1}H} NMR (101 MHz, $CDCl_3$, 298 K): δ = 12.0, 26.2, 51.4, 125.3, 127.9, 128.9 (2C), 133.2, 138.1, 179.0 ppm.

4 Experimental Details for Synthesis of α,α-Disubstituted Acetyl Chlorides

4.1 General Procedure for the Synthesis of α,α-Diarylacetyl Chlorides (GP 3)

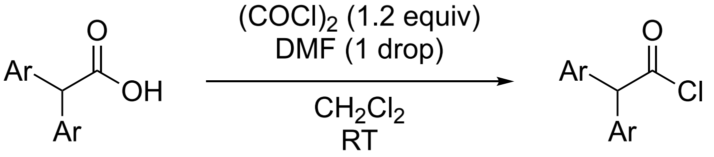

Under a nitrogen atmosphere, α,α-diarylacetic acid (1.0 equiv) was dissolved in freshly distilled dry CH_2Cl_2 (c = 0.4 M). The solution was cooled to 0 °C and oxalyl chloride (1.2 equiv) was added dropwise followed by the addition of a drop of dry DMF. The solution was allowed to warm to room temperature and stirred until all the starting materials were consumed (typically 4–12 h). After completion of the reaction, the volatiles were evaporated and the crude material was dissolved in hot hexanes, activated charcoal was added, and refluxed for 5 min. The hot mixture was filtered and cooled to 0 °C. After 2 h the white solid was filtered and rinsed with cold hexanes. The acid chlorides were generally used immediately.

4.2 General Procedure for Synthesis of α-Alkyl-α-arylacetyl Chlorides (GP 4)

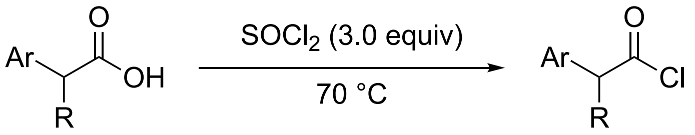

Under a nitrogen atmosphere, $SOCl_2$ (3.0 equiv) was directly added in one portion to α-alkyl-α-arylacetic acid (1.0 equiv). The reaction mixture was stirred at 70 °C for 4–12 h. After cooling to room temperature, excess $SOCl_2$ was carefully removed in vacuo and the residue was purified by bulb-to-bulb distillation to afford the desired product as colorless oil. The acid chlorides were generally used immediately.

5 Experimental Details for the Synthesis of Ketenes

5.1 General Procedure for the Synthesis of Diarylketenes (GP 5)

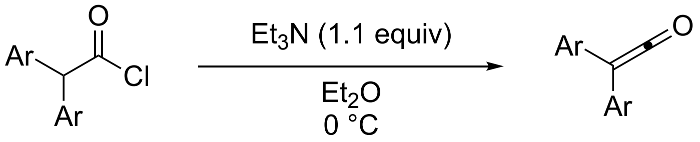

Under a nitrogen atmosphere, acid chloride (1.0 equiv) was dissolved in freshly distilled dry Et_2O (c = 0.5 M). The mixture was cooled to 0 °C and freshly distilled Et_3N (1.1 equiv) was added dropwise over 30 min. The reaction mixture was stirred at 0 °C for 18 h, and then warmed to room temperature. The white precipitate was filtered off using a flame-dried Schlenk frit equipped with a flame-dried collecting flask. The precipitate was thoroughly rinsed with dry Et_2O under nitrogen atmosphere. The solvent and excess Et_3N were carefully removed from the filtrate in vacuo under Schlenk line. The crude ketene was then purified by Kugelrhor distillation at the indicated temperature and pressure. After distillation, ketenes were directly transferred into a Schlenk tube, degassed by three freeze-pump-thaw pump cycles, taken in the glove box, and yield was determined.

2,2-Diphenylethen-1-one was characterized in previous literature.[5]

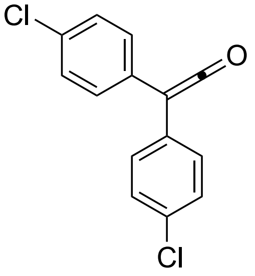

1b
$C_{14}H_8Cl_2O$
M = 263.12 g/mol

2,2-Bis(4-chlorophenyl)ethen-1-one (1b): Prepared from 2,2-bis(4-chlorophenyl)acetyl chloride (0.36 g, 1.2 mmol, 1.0 equiv) according to **GP 5**. This compound was purified by dissolving it in cold *n*-pentane (–40 °C) followed by filtration, and evaporation of the solvent (0.19 g, 59%). The spectroscopic data were in accordance with those reported.[6]

^{1}H NMR (500 MHz, CD_2Cl_2, 298 K): δ = 7.15 (d, *J* = 8.5, 4H), 7.36 (d, *J* = 8.5, 4H) ppm.
^{13}C{^{1}H} NMR (126 MHz, CD_2Cl_2, 298 K): δ = 46.2, 129.3, 129.5, 129.9, 132.4, 200.2 ppm.
HRMS (APCI): calculated for $C_{14}H_8Cl_2O^{+\bullet}$ $[M]^{+\bullet}$: 261.9952; found 261.9948.

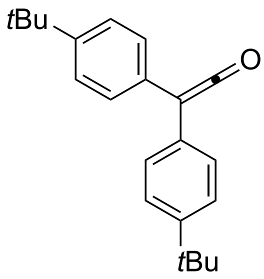

1c
$C_{22}H_{26}O$
M = 306.45 g/mol

2,2-Bis(4-(tert-butyl)phenyl)ethen-1-one (1c): Prepared from 2,2-bis(4-(*tert*-butyl)phenyl)acetyl chloride (2.0 g, 5.8 mmol, 1.0 equiv) according to **GP 5**. Purification by Kugelrhor distillation (0.6 mbar/ 150 °C) afforded the ketene **1c** (0.32 g, 18%) as orange-red viscous oil.

^{1}H NMR (500 MHz, CD_2Cl_2, 298 K): δ = 1.33 (s, 18H), 7.15 (d, *J* = 8.6, 4H), 7.40 (d, *J* = 8.6, 4H) ppm.
^{13}C{^{1}H} NMR (126 MHz, CD_2Cl_2, 298 K): δ = 31.5, 34.8, 46.2, 126.6, 127.7, 128.0, 149.7, 203.3 ppm.
HRMS (APCI): calculated for $C_{22}H_{26}O^{+\bullet}$ $[M]^{+\bullet}$: 306.1984; found 306.1978.

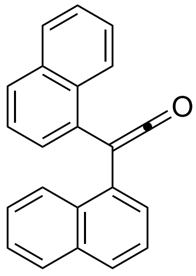

1d
$C_{22}H_{14}O$
M = 294.35 g/mol

2,2-Di(naphthalen-1-yl)ethen-1-one (1d): Prepared from 2,2-di(naphthalen-1-yl)acetyl chloride (1.0 g, 3.0 mmol, 1.0 equiv) according to **GP 5**. This compound was purified by dissolving it in cold *n*-pentane (–40 °C) followed by filtration, and evaporation of the solvent (0.13 g, 15%). The yellow solid was found to be stable in air.

IR (ATR): ṽ = 2923, 2086 (C=O), 1811, 1719, 1570, 1504, 1392, 1267, 1018, 767 cm^{-1}.
^{1}H NMR (500 MHz, $CDCl_3$, 298 K): δ = 7.32 (dd, *J* = 7.2, 1.0 Hz, 2H), 7.42 (t, *J* = 7.7, 2H), 7.46–7.54 (m_c, 4H), 7.81 (d, *J* = 8.3, 2H), 7.92 (d, *J* = 8.0, 2H), 8.09 (d, *J* = 8.4, 2H) ppm.
^{13}C{^{1}H} NMR (126 MHz, $CDCl_3$, 298 K): δ = 40.7, 124.8, 126.1, 126.3, 126.7, 127.9, 128.1, 129.0, 129.1, 131.8, 134.6, 195.2 ppm.
HRMS (APCI): calculated for $C_{22}H_{14}O^{+\bullet}$ $[M]^{+\bullet}$: 294.1045; found 294.1043.

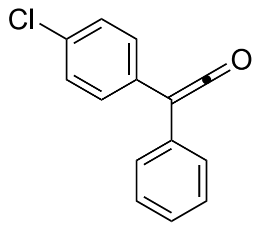

1e
$C_{14}H_9ClO$
M = 228.68 g/mol

2-(4-Chlorophenyl)-2-phenylethen-1-one (1e): Prepared from 2-(4-chlorophenyl)-2-phenylacetyl chloride (1.4 g, 5.5 mmol, 1.0 equiv) according to **GP 5**. Purification by Kugelrhor distillation (1.1 mbar/ 120 °C) afforded the ketene **1e** (0.85 g, 68%) as orange-red oil.

^{1}H NMR (500 MHz, CD_2Cl_2, 298 K): δ = 7.15–7.17 (m, 2H), 7.21–7.27 (m, 3H), 7.33–7.40 (m, 4H) ppm.
^{13}C{^{1}H} NMR (126 MHz, CD_2Cl_2, 298 K): δ = 46.8, 127.0, 128.2, 129.2, 129.8, 130.1, 130.6, 131.8, 132.0, 201.0 ppm.
HRMS (APCI): calculated for $C_{14}H_{10}ClO^{+\bullet}$ $[M+H]^{+\bullet}$: 229.0415; found 229.0418.

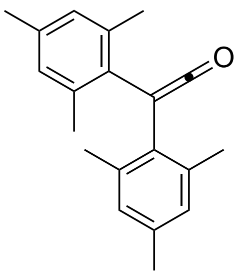

1r
$C_{20}H_{22}O$
M = 278.40 g/mol

2,2-Dimesitylethen-1-one (1r): Prepared from 2,2-dimesitylacetyl chloride (0.41 g, 1.3 mmol, 1.0 equiv) according to **GP 5**. Purification by Kugelrhor distillation (0.9 mbar/ 140 °C) afforded the ketene **1r** as yellow solid (0.23 g, 63%). This ketene was found to be stable in air.

IR (ATR): ṽ = 2914, 2090 (C=O), 1699, 1457, 1376, 1033, 854, 730 cm^{-1}.
^{1}H NMR (500 MHz, $CDCl_3$, 298 K): δ = 2.11 (s, 12H), 2.27 (s, 6H), 6.87 (s, 4H) ppm.
^{13}C{^{1}H} NMR (126 MHz, $CDCl_3$, 298 K): δ = 21.0, 39.0, 126.3, 129.8, 136.5, 137.8, 191.6 ppm.
HRMS (APCI): calculated for $C_{20}H_{22}O^{+\bullet}$ $[M]^{+\bullet}$: 278.1671; found 278.1669.

5.2 General Procedure for the Synthesis of Alkylarylketenes (GP 6)

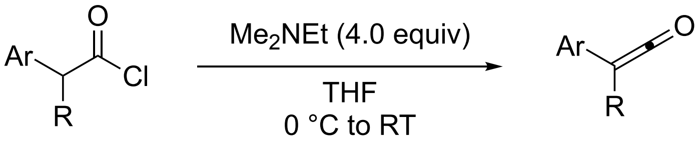

Under a nitrogen atmosphere, acid chloride (1.0 equiv) was dissolved in freshly distilled dry Et_2O (c = 0.5 M). The mixture was cooled to 0 °C and Me_2NEt (4.0 equiv) was added dropwise. The mixture was stirred at 0 °C (or at room temperature) for 24 h, and then warmed to room temperature. The white precipitate was filtered off using a flame-dried Schlenk frit equipped with a flame-dried collecting flask. The precipitate was thoroughly rinsed with dry Et_2O under nitrogen atmosphere. The solvent and excess Me_2NEt were carefully removed from the filtrate in vacuo. The crude ketene was then purified by Kugelrhor distillation at the indicated temperature and pressure. After distillation, ketenes were directly transferred into a Schlenk tube, degassed by three freeze-pump-thaw pump cycles, taken in the glove box, and yield was determined.

2-Phenylbut-1-en-1-one, 2-cyclopentyl-2-phenylethen-1-one, 2,3-diphenylprop-1-en-1-one, 2-(*p*-tolyl)but-1-en-1-one, 2-(4-fluorophenyl)but-1-en-1-one, 2-(*o*-tolyl)but-1-en-1-one, 2-(2-chlorophenyl)but-1-en-1-one, 2-(*m*-tolyl)but-1-en-1-one, 2-(3-chlorophenyl)but-1-en-1-one, and 2-(naphthalen-1-yl)but-1-en-1-one were characterized in the previous literatures.[7–11]

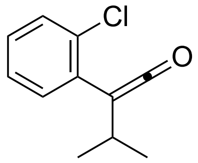

1h
$C_{11}H_{11}ClO$
M = 194.66 g/mol

2-(2-Chlorophenyl)-3-methylbut-1-en-1-one (1h): Prepared from 2-(2-chlorophenyl)-3-methylbutanoyl chloride (1.6 g, 6.9 mmol, 1.0 equiv) according to **GP 6**. After addition of Me_2NEt at 0 °C, the reaction mixture was stirred at room temperature for 24 h. Purification by Kugelrhor distillation (10 mbar/ 80 °C) afforded the ketene **1h** (~ 80% pure by ^{1}H NMR, 0.55 g, 41%) as yellow oil.

^{1}H NMR (400 MHz, CD_2Cl_2, 298 K): δ = 1.18 (d, *J* = 6.7 Hz, 6H), 2.90 (sept, *J* = 6.7 Hz, 1H), 7.08 (ddd, *J* = 8.0, 7.3, 1.7 Hz, 1H), 7.14–7.18 (m, 1H), 7.24 (dd, *J* = 7.3, 1.4 Hz, 1H), 7.33–7.36 (m, 1H) ppm.
^{13}C{^{1}H} NMR (101 MHz, CD_2Cl_2, 298 K): δ = 22.1, 26.3, 45.6, 126.5, 127.6, 127.8, 130.0, 131.7, 133.0, 198.6 ppm.

NMR data of the unreacted acid chloride:

^{1}H NMR (400 MHz, $CDCl_3$, 298 K): δ = 0.74 (d, *J* = 6.8 Hz, 3H), 1.15 (d, *J* = 6.5 Hz, 3H), 2.41 (m_c, 1H), 4.42 (d, *J* = 10.1 Hz, 1H), 7.25–7.36 (m, 3H), 7.42–7.48 (m, 1H) ppm.
^{13}C{^{1}H} NMR (101 MHz, $CDCl_3$, 298 K): δ = 19.6, 21.3, 32.8, 66.8, 128.0, 129.5, 129.9, 130.5, 133.7, 135.6, 174.1 ppm.

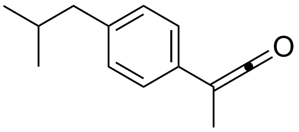

1j
$C_{13}H_{16}O$
M = 188.27 g/mol

2-(4-Isobutylphenyl)prop-1-en-1-one (1j): Prepared from 2-(4-isobutylphenyl)propanoyl chloride (1.5 g, 6.7 mmol, 1.0 equiv) according to **GP 6**. After addition of Me_2NEt at 0 °C, the reaction mixture was stirred at room temperature for 18 h. Purification by Kugelrhor distillation (1.2 mbar/ 70 °C) afforded the ketene **1j** (0.57 g, 45%) as orange oil. This ketene was used immediately as storing for even few days led to complete decomposition.

^{1}H NMR (500 MHz, CD_2Cl_2, 298 K): δ = 0.90 (d, *J* = 6.6 Hz, 6H), 1.83 (sept, *J* = 6.8 Hz, 1H), 1.99 (s, 3H), 2.44 (d, *J* = 7.2, 2H), 6.95 (d, *J* = 8.2 Hz, 2H), 7.11 (d, *J* = 8.2 Hz, 2H) ppm.
^{13}C{^{1}H} NMR (126 MHz, CD_2Cl_2, 298 K): δ = 8.9, 22.5, 30.7, 33.7, 45.2, 123.8, 130.1, 130.6, 138.2, 207.4 ppm.
HRMS (APCI): calculated for $C_{13}H_{16}O^{+\bullet}$ $[M]^{+\bullet}$: 188.1201; found 188.1196.

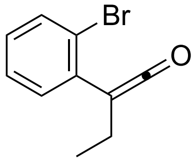

1m
$C_{10}H_9BrO$
M = 225.09 g/mol

2-(2-Bromophenyl)but-1-en-1-one (1m): Prepared from 2-(2-bromophenyl)butanoyl chloride (2.2 g, 8.3 mmol, 1.0 equiv) according to **GP 6**. Purification by Kugelrhor distillation (0.6 mbar/ 70 °C) afforded the ketene **1m** (1.1 g, 58%) as orange oil.

^{1}H NMR (500 MHz, CD_2Cl_2, 298 K): δ = 1.19 (t, *J* = 7.4 Hz, 3H), 2.51 (q, *J* = 7.4 Hz, 2H), 7.01 (td, *J* = 7.9, 1.6 Hz, 1H), 7.11 (dd, *J* = 8.0, 1.5 Hz, 1H), 7.31 (td, *J* = 8.0, 1.3 Hz, 1H), 7.54 (dd, *J* = 8.0, 1.2 Hz, 1H) ppm.
^{13}C{^{1}H} NMR (126 MHz, CD_2Cl_2, 298 K): δ = 13.2, 20.6, 41.8, 123.5, 126.7, 127.3, 128.2, 133.4, 133.8, 198.5 ppm.
HRMS (APCI): calculated for $C_{10}H_9BrO^{+\bullet}$ $[M]^{+\bullet}$: 223.9837; found 223.9835.

6 Experimental Details for the Hydrosilylation of Ketenes with $B(C_6F_5)_3$

6.1 General Procedure for Hydrosilylation of Ketenes with $B(C_6F_5)_3$ (GP 7)

In an argon-filled glovebox, $B(C_6F_5)_3$ (5.0 mol%) was dissolved in C_6H_5F in a GC vial, and the solution of ketene (1.0 equiv) in C_6H_5F (c = 0.5 M) was added dropwise at room temperature. The desired trialkylsilane (1.2 equiv) or dihydrosilane (1.0 equiv) was then added dropwise by a microsyringe. The resulting mixture was maintained at this temperature for additional 12 h. After taking the reaction vessel out of the glovebox, the internal standard (CH_2Br_2, 0.5 equiv) was added to the reaction mixture to determine the yield by ^{1}H NMR spectroscopy. The solvent and other volatiles were removed by evaporation. The crude oil was purified by flash column chromatography on alumina using *n*-pentane as eluent to afford the silyl enol ether in analytically pure form.

The *E*:*Z* ratio was determined by ^{1}H NMR analysis of the crude reaction mixture.

The relative configuration of diastereomers was determined from 2D-NOESY experiments.

Peaks in ^{13}C NMR spectrum were assigned based on ^{13}C-DEPT, ^{1}H/^{13}C-HMQC, and ^{1}H/^{13}C-HMBC measurements.

Silyl enol ethers were found to partially decomposed in $CDCl_3$ on keeping for long NMR measurements.

In case of the mixture, the desired compound (silyl enol ether) was found to begin eluting before the side product (silyl ether). Therefore, a small quantity was isolated with high purity for NMR analysis to aid unambiguous characterization.

6.2 Characterization Data for Hydrosilylation of Ketenes with $B(C_6F_5)_3$

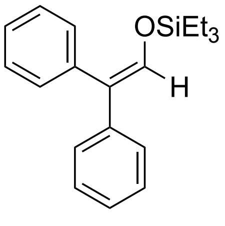

3aa
$C_{20}H_{26}OSi$
M = 310.51 g/mol

((2,2-Diphenylvinyl)oxy)triethylsilane (3aa): Prepared from 2,2-diphenylethen-1-one (**1a**, 39 mg, 0.20 mmol, 1.0 equiv) according to **GP 7**. CH_2Br_2 (7.0 µL, 0.10 mmol, 0.50 equiv) was added as internal standard to determine the yield by NMR spectroscopy (94%). Purification by flash column chromatography on alumina using *n*-pentane as eluent afforded the silyl enol ether **3aa** (52 mg, 84%) as colorless oil.

R_f = 0.51 (1% ethyl acetate in cyclohexane).
IR (ATR): ṽ = 2954, 2875, 1620, 1494, 1458, 1208, 1115, 1003, 952, 792, 693 cm^{-1}.
^{1}H NMR (500 MHz, $CDCl_3$, 298 K): δ = 0.72 (q, *J* = 7.9 Hz, 6H), 1.00 (t, *J* = 7.9 Hz, 9H), 6.74 (s, 1H), 7.19–7.24 (m, 4H), 7.28–7.32 (m, 4H), 7.41–7.43 (m, 2H) ppm.
^{13}C{^{1}H} NMR (126 MHz, $CDCl_3$, 298 K): δ = 4.7, 6.7, 123.8, 126.4, 126.5, 127.8, 128.3, 128.8, 130.1, 138.1, 139.1, 141.1 ppm.
^{29}Si DEPT NMR (99 MHz, $CDCl_3$, 298 K, optimized for *J* = 7 Hz): δ = 23.7 ppm.
HRMS (APCI): calculated for $C_{20}H_{26}OSi^{+\bullet}$ $[M]^{+\bullet}$: 310.1753; found 310.1750.

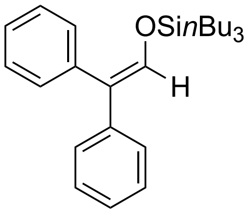

3ab
$C_{26}H_{38}OSi$
M = 394.67 g/mol

Tributyl((2,2-diphenylvinyl)oxy)silane (3ab): Prepared from 2,2-diphenylethen-1-one (**1a**, 39 mg, 0.20 mmol, 1.0 equiv) according to **GP 7**. CH_2Br_2 (7.0 µL, 0.10 mmol, 0.50 equiv) was added as internal standard to determine the yield by NMR spectroscopy (94%). Purification by flash column chromatography on alumina using *n*-pentane as eluent afforded the silyl enol ether **3ab** (65 mg, 82%) as colorless oil.

R_f = 0.59 (1% ethyl acetate in cyclohexane).

IR (ATR): ṽ = 2954, 2920, 1620, 1495, 1460, 1212, 1117, 1075, 953, 883, 793, 760, 694 cm^{-1}.

^{1}H NMR (500 MHz, $CDCl_3$, 298 K): δ = 0.69–0.73 (m, 6H), 0.88–0.91 (m, 9H), 1.32–1.37 (m, 12H), 6.73 (s, 1H), 7.19–7.26 (m, 4H), 7.28–7.32 (m, 4H), 7.41–7.43 (m, 2H) ppm.

^{13}C{^{1}H} NMR (126 MHz, $CDCl_3$, 298 K): δ = 13.4, 13.9, 25.3, 26.6, 123.8, 126.3, 126.5, 127.8, 128.3, 128.7, 130.1, 138.1, 139.1, 141.1 ppm.

^{29}Si DEPT NMR (99 MHz, $CDCl_3$, 298 K, optimized for *J* = 7 Hz): δ = 21.4 ppm.

HRMS (APCI): calculated for $C_{26}H_{38}OSi^{+\bullet}$ $[M]^{+\bullet}$: 394.2692; found 394.2690.

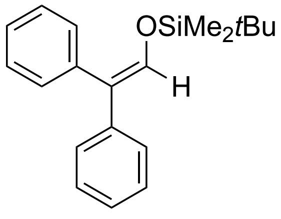

3ad
$C_{20}H_{26}OSi$
M = 310.51 g/mol

***Tert*-butyl((2,2-diphenylvinyl)oxy)dimethylsilane (3ad):** Prepared from 2,2-diphenylethen-1-one (**1a**, 39 mg, 0.20 mmol, 1.0 equiv) according to **GP 7**. CH_2Br_2 (7.0 µL, 0.10 mmol, 0.50 equiv) was added as internal standard to determine the yield by NMR spectroscopy (67%). Purification by flash column chromatography on alumina using *n*-pentane as eluent afforded the silyl enol ether **3ad** (35 mg, 56%) as colorless oil.

R_f = 0.49 (1% ethyl acetate in cyclohexane).

IR (ATR): ṽ = 2928, 2856, 1621, 1494, 1442, 1254, 1210, 1116, 953, 828, 762, 694 cm^{-1}.

^{1}H NMR (500 MHz, $CDCl_3$, 298 K): δ = 0.19 (s, 6H), 0.92 (s, 9H), 6.71 (s, 1H), 7.19–7.26 (m, 4H), 7.28–7.32 (m, 4H), 7.41–7.43 (m, 2H) ppm.

^{13}C{^{1}H} NMR (126 MHz, $CDCl_3$, 298 K): δ = –5.1, 18.3, 25.7, 123.9, 126.4, 126.5, 127.8, 128.3, 128.7, 130.1, 138.1, 139.1, 141.0 ppm.

^{29}Si DEPT NMR (99 MHz, $CDCl_3$, 298 K, optimized for *J* = 7 Hz): δ = 24.1 ppm.

HRMS (APCI): calculated for $C_{20}H_{26}OSi^{+\bullet}$ $[M]^{+\bullet}$: 310.1753; found 310.1747.

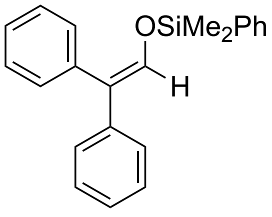

3ae
$C_{22}H_{22}OSi$
M = 330.50 g/mol

((2,2-Diphenylvinyl)oxy)dimethyl(phenyl)silane (3ae): Prepared from 2,2-diphenylethen-1-one (**1a**, 39 mg, 0.20 mmol, 1.0 equiv) according to **GP 7**. CH_2Br_2 (7.0 µL, 0.10 mmol, 0.50 equiv) was added as internal standard to determine the yield by NMR spectroscopy (98%). Attempt to purification by flash column chromatography on alumina using *n*-pentane as eluent led to decomposition of the silyl enol ether **3ae**. For NMR characterization, the reaction was performed in J-Young tube under argon atmosphere in CD_2Cl_2 solvent.

Selected NMR characterization data of the crude reaction mixture:
^{1}H NMR (500 MHz, CD_2Cl_2, 298 K): δ = 0.53 (s, 6H), 6.76 (s, 1H), 7.18–7.20 (m, 2H), 7.24–7.30 (m, 4H), 7.33–7.36 (m, 2H), 7.38–7.46 (m, 5H), 7.62–7.63 (m, 2H) ppm.
^{29}Si DEPT NMR (99 MHz, $CDCl_3$, 298 K, optimized for *J* = 7 Hz): δ = 12.1 ppm.

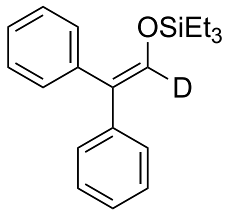

3aa-*d*$_1$
[>95% ^{2}H]
$C_{20}H_{25}DOSi$
M = 311.52 g/mol

((2,2-Diphenylvinyl-1-*d*)oxy)triethylsilane (3aa-*d*$_1$): Prepared from 2,2-diphenylethen-1-one (**1a**, 39 mg, 0.20 mmol, 1.0 equiv) according to **GP 7**. Purification by flash column chromatography on alumina using *n*-pentane as eluent afforded the silyl enol ether **3aa**-*d*$_1$ (47 mg, 75%, >99% ^{2}H) as colorless oil.

R$_f$ = 0.51 (1% ethyl acetate in cyclohexane).
IR (ATR): ṽ = 2954, 2876, 1602, 1493, 1441, 1223, 1108, 1005, 793, 693 cm^{-1}.
^{1}H NMR (500 MHz, $CDCl_3$, 298 K): δ = 0.73 (q, *J* = 7.9 Hz, 6H), 1.01 (t, *J* = 7.9 Hz, 9H), 7.19–7.25 (m, 4H), 7.29–7.32 (m, 4H), 7.43–7.45 (m, 2H) ppm.
^{13}C{^{1}H} NMR (126 MHz, $CDCl_3$, 298 K): δ = 4.6, 6.7, 123.7, 126.3, 126.5, 127.8, 128.3, 128.7, 130.1, 138.1, 141.1 ppm.
^{29}Si DEPT NMR (99 MHz, $CDCl_3$, 298 K, optimized for *J* = 7 Hz): δ = 23.7 ppm.
^{2}H NMR (500 MHz, $CDCl_3$, 298 K): δ = 6.79 ppm.
HRMS (APCI): calculated for $C_{20}H_{25}DOSi^{+•}$ $[M]^{+•}$: 311.1816; found 311.1809.

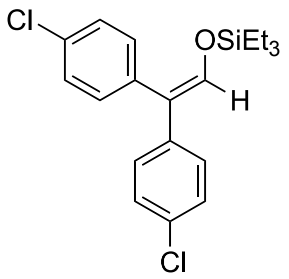

3ba
$C_{20}H_{24}Cl_2OSi$
M = 379.40 g/mol

((2,2-Bis(4-chlorophenyl)vinyl)oxy)triethylsilane (3ba): Prepared from 2,2-bis(4-chlorophenyl)ethen-1-one (**1b**, 53 mg, 0.20 mmol, 1.0 equiv) according to **GP 7**. CH_2Br_2 (7.0 µL, 0.10 mmol, 0.50 equiv) was added as internal standard to determine the yield by NMR spectroscopy (59%). Purification by flash column chromatography on alumina using *n*-pentane as eluent afforded the silyl enol ether **3ba** (17 mg, 22%) as colorless oil.

R*f* = 0.58 (1% ethyl acetate in cyclohexane).
IR (ATR): ṽ = 2953, 2875, 1651, 1486, 1267, 1172, 1009, 925, 748, 667 cm^{-1}.
^{1}H NMR (500 MHz, $CDCl_3$, 298 K): δ = 0.72 (q, *J* = 7.9 Hz, 6H), 0.99 (t, *J* = 7.9 Hz, 9H), 6.70 (s, 1H), 7.10–7.12 (m, 2H), 7.24–7.26 (m, 2H), 7.27–7.28 (m, 2H) 7.31–7.34 (m, 2H) ppm.
^{13}C{^{1}H} NMR (126 MHz, $CDCl_3$, 298 K): δ = 4.6, 6.6, 121.7, 128.1, 128.6, 129.9, 131.3, 132.1, 132.5, 136.1, 139.1, 139.7 ppm.
^{29}Si DEPT NMR (99 MHz, $CDCl_3$, 298 K, optimized for *J* = 7 Hz): δ = 24.7 ppm.
HRMS (APCI): calculated for $C_{20}H_{24}Cl_2OSi^{+\bullet}$ $[M]^{+\bullet}$: 378.0973; found 378.0973.

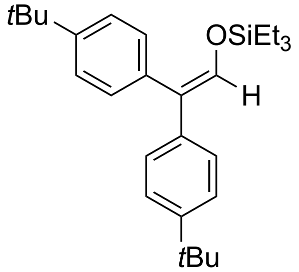

3ca
$C_{28}H_{42}OSi$
M = 422.73 g/mol

((2,2-Bis(4-(*tert*-butyl)phenyl)vinyl)oxy)triethylsilane (3ca): Prepared from 2,2-bis(4-(tert-butyl)phenyl)ethen-1-one (**1c**, 123 mg, 0.40 mmol, 1.0 equiv) according to **GP 7**. CH_2Br_2 (14 µL, 0.20 mmol, 0.50 equiv) was added as internal standard to determine the yield by NMR spectroscopy (quant.). Purification by flash column chromatography on alumina using *n*-pentane as eluent afforded the silyl enol ether **3ca** (69 mg, 41%) as colorless oil.

R*f* = 0.44 (1% ethyl acetate in cyclohexane).

IR (ATR): ṽ = 2954, 2875, 1620, 1512, 1459, 1361, 1213, 1128, 1013, 959, 832, 797, 730 cm^{-1}.

^{1}H NMR (500 MHz, $CDCl_3$, 298 K): δ = 0.73 (q, *J* = 7.9 Hz, 6H), 1.01 (t, *J* = 7.9 Hz, 9H), 1.34 (s, 9H), 1.33 (s, 9H), 6.70 (s, 1H), 7.17–7.20 (m, 2H), 7.31–7.33 (m, 4H), 7.39–7.42 (m, 2H) ppm.

^{13}C{^{1}H} NMR (126 MHz, $CDCl_3$, 298 K): δ = 4.7, 6.7, 31.5, 31.6, 34.6 (2C), 123.2, 124.6, 125.2, 128.6, 129.6, 135.1, 138.2, 138.6, 149.0, 149.3 ppm.

^{29}Si DEPT NMR (99 MHz, $CDCl_3$, 298 K, optimized for *J* = 7 Hz): δ = 23.5 ppm.

HRMS (APCI): calculated for $C_{28}H_{42}OSi^{+\bullet}$ $[M]^{+\bullet}$: 422.3005; found 422.2997.

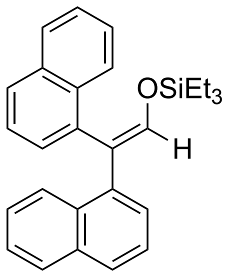

3da
$C_{28}H_{30}OSi$
M = 410.63 g/mol

((2,2-Di(naphthalen-1-yl)vinyl)oxy)triethylsilane (3da): Prepared from 2,2-di(naphthalen-1-yl)ethen-1-one (**1d**, 59 mg, 0.20 mmol, 1.0 equiv) according to **GP 7**. CH_2Br_2 (7.0 µL, 0.10 mmol, 0.50 equiv) was added as internal standard to determine the yield by NMR spectroscopy (67%). Purification by flash column chromatography on alumina using *n*-pentane as eluent afforded the silyl enol ether **3da** (29 mg, 35%) as colorless oil.

R_f = 0.23 (1% ethyl acetate in cyclohexane).

IR (ATR): ṽ = 2952, 2873, 1626, 1506, 1457, 1399, 1226, 1185, 1140, 999, 774, 727 cm^{-1}.

^{1}H NMR (500 MHz, $CDCl_3$, 298 K): δ = 0.60 (q, *J* = 7.9 Hz, 6H), 0.85 (t, *J* = 7.9 Hz, 9H), 6.95 (s, 1H), 7.28–7.31 (m, 2H), 7.34–7.37 (m, 1H), 7.39–7.49 (m, 5H), 7.74–7.79 (m, 2H), 7.83–7.88 (m, 2H), 8.12 (d, *J* = 8.5 Hz, 1H), 8.24–8.26 (m, 1H) ppm.

^{13}C{^{1}H} NMR (126 MHz, $CDCl_3$, 298 K): δ = 4.7, 6.5, 120.3, 125.4 (2C), 125.5 (2C), 125.6, 126.0, 126.2, 127.2, 127.4, 127.6, 128.1, 128.3, 128.4, 131.4, 133.0, 134.0, 134.3, 137.3, 139.2, 141.2 ppm. (one carbon signal is not visible)

^{29}Si DEPT NMR (99 MHz, $CDCl_3$, 298 K, optimized for *J* = 7 Hz): δ = 23.2 ppm.

HRMS (APCI): calculated for $C_{28}H_{30}OSi^{+\bullet}$ $[M]^{+\bullet}$: 410.2066; found 410.2061.

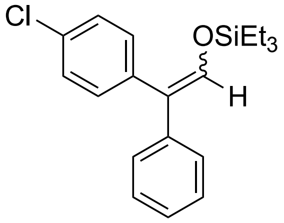

Z/*E*-**3ea**
[*Z*:*E* = 60:40]
$C_{20}H_{25}ClOSi$
M = 344.95 g/mol

***Z*/*E*-((2-(4-chlorophenyl)-2-phenylvinyl)oxy)triethylsilane (3ea):** Prepared from 2-(4-chlorophenyl)-2-phenylethen-1-one (**1e**, 91 mg, 0.40 mmol, 1.0 equiv) according to **GP 7**. CH_2Br_2 (14 µL, 0.20 mmol, 0.50 equiv) was added as internal standard to determine the yield by NMR spectroscopy (68%). Purification by flash column chromatography on alumina using *n*-pentane as eluent afforded the pure silyl enol ether **3ea** (69 mg, 50%, *Z*:*E* = 62:38) as colorless oil.

R*f* = 0.55 (1% ethyl acetate in cyclohexane).
IR (ATR): ṽ = 2954, 2875, 1619, 1489, 1458, 1209, 1117, 1010, 954, 829, 792, 729, 694 cm^{-1}.
^{1}H NMR (500 MHz, $CDCl_3$, 298 K): δ = 0.70–0.75 (m, 10H), 0.98–1.02 (m, 15H), 6.72 (s, 0.6H), 6.73 (s, 1H), 7.14–7.16 (m, 1H), 7.19–7.22 (m, 3H), 7.25–7.28 (m, 5H), 7.29–7.32 (m, 4H), 7.35–7.40 (m, 3H) ppm.
^{13}C{^{1}H} NMR (126 MHz, $CDCl_3$, 298 K): δ = 4.6, 6.7, 122.7, 122.9, 126.6, 126.7, 127.9, 128.0, 128.5 (2C), 128.8, 129.9, 130.0, 131.4, 131.8, 132.2, 136.6, 137.7, 139.3, 129.5, 139.6, 140.6 ppm.
^{29}Si DEPT NMR (99 MHz, $CDCl_3$, 298 K, optimized for *J* = 7 Hz): δ = 24.1 ppm.
HRMS (APCI): calculated for $C_{20}H_{25}ClOSi^{+\bullet}$ $[M]^{+\bullet}$: 344.1363; found 344.1367.

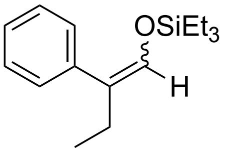

Z/*E*-**3fa**
[*Z*:*E* = 86:14]
$C_{16}H_{26}OSi$
M = 262.47 g/mol

***Z*/*E*-triethyl((2-phenylbut-1-en-1-yl)oxy)silane (3fa):** Prepared from 2-phenylbut-1-en-1-one (**1f**, ~95% pure by ^{1}H NMR, 29 mg, 0.20 mmol, 1.0 equiv) according to **GP 7**. CH_2Br_2 (7.0 µL, 0.10 mmol, 0.50 equiv) was added as internal standard to determine the yield by NMR spectroscopy (67%). Purification by flash column chromatography on alumina using *n*-pentane

as eluent afforded the silyl enol ether **3fa** (27 mg, 51%, *Z*:*E* = 79:21) combined with silyl ether as colorless oil.

R*f* = 0.54 (1% ethyl acetate in cyclohexane).
IR (ATR): ṽ = 2956, 2875, 1637, 1458, 1239, 1164, 1004, 813, 726, 693 cm^{-1}.
^{1}H NMR (500 MHz, $CDCl_3$, 298 K): δ = 0.66 (q, *J* = 7.9 Hz, 6H), 0.72 (q, *J* = 7.9 Hz, 2H), 0.96 (t, *J* = 7.9 Hz, 9H), 0.98–1.04 (m, 7H), 2.33 (qd, *J* = 7.4, 1.0 Hz, 2H), 2.56 (q, *J* = 7.5 Hz, 1H), 6.42 (s, 1H), 6.58 (s, 0.3H), 7.14–7.20 (m, 1H), 7.28–7.32 (m, 3H), 7.53–7.55 (m, 2H) ppm.
^{13}C{^{1}H} NMR (126 MHz, $CDCl_3$, 298 K): δ = 4.6, 4.7, 6.7 (2C), 13.1, 14.5, 20.1, 25.6, 121.4, 125.0, 125.8, 125.9, 126.1, 127.8, 128.4 (2C), 136.1, 137.1, 137.8, 140.2 ppm.
^{29}Si DEPT NMR (99 MHz, $CDCl_3$, 298 K, optimized for *J* = 7 Hz): δ = 21.9 ppm.
HRMS (APCI): calculated for $C_{16}H_{26}OSi^{+\bullet}$ $[M]^{+\bullet}$: 262.1753; found 262.1746.

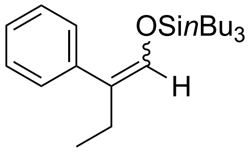

***Z*/*E*-3fb**
[*Z*:*E* = 84:16]
$C_{22}H_{38}OSi$
M = 346.63 g/mol

***Z*/*E*-tributyl((2-phenylbut-1-en-1-yl)oxy)silane (3fb):** Prepared from 2-phenylbut-1-en-1-one (**1f**, ~95% pure by ^{1}H NMR, 37 mg, 0.25 mmol, 1.0 equiv) according to **GP 7**. CH_2Br_2 (8.8 µL, 0.125 mmol, 0.50 equiv) was added as internal standard to determine the yield by NMR spectroscopy (74%). Purification by flash column chromatography on alumina using *n*-pentane as eluent afforded the silyl enol ether **3fb** (68 mg, 78%, *Z*:*E* = 81:19) combined with silyl ether as colorless oil.

R*f* = 0.65 (1% ethyl acetate in cyclohexane).
IR (ATR): ṽ = 2956, 2920, 2870, 1637, 1460, 1376, 1251, 1165, 1076, 884, 814, 762, 693 cm^{-1}.
^{1}H NMR (500 MHz, $CDCl_3$, 298 K): δ = 0.63–0-67 (m, 6H), 0.70–0.73 (m, 1H), 0.86–0.92 (m, 11H), 0.97–1.02 (m, 4H), 1.30–1.33 (m, 13H), 1.36–1.39 (m, 3H), 2.33 (qd, *J* = 7.4, 1.0 Hz, 2H), 2.55 (q, *J* = 7.5 Hz, 1H), 6.40 (s, 1H), 6.56 (s, 0.2H), 7.15–7.20 (m, 1H), 7.28–7.32 (m, 3H), 7.53–7.54 (m, 2H) ppm.
^{13}C{^{1}H} NMR (126 MHz, $CDCl_3$, 298 K): δ = 13.4, 13.9, 14.5, 25.3, 25.7, 26.6, 121.4, 125.8, 127.8, 128.4, 136.1, 137.9 ppm. (for *Z*-isomer)
^{29}Si DEPT NMR (99 MHz, $CDCl_3$, 298 K, optimized for *J* = 7 Hz): δ = 19.6 ppm.
HRMS (APCI): calculated for $C_{22}H_{38}OSi^{+\bullet}$ $[M]^{+\bullet}$: 346.2692; found 346.2690.

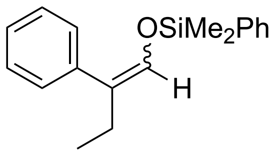

Z/*E*-**3fc**
[*Z*:*E* = 62:38]
$C_{18}H_{22}OSi$
M = 282.46 g/mol

***Z*/*E*-dimethyl(phenyl)((2-phenylbut-1-en-1-yl)oxy)silane (3fc):** Prepared from 2-phenylbut-1-en-1-one (**1f**, ~95% pure by ^{1}H NMR, 37 mg, 0.25 mmol, 1.0 equiv) according to **GP 7**. CH_2Br_2 (8.8 µL, 0.125 mmol, 0.50 equiv) was added as internal standard to determine the yield by NMR spectroscopy (89%). Attempt to purification by flash column chromatography on alumina using *n*-pentane as eluent led to complete decomposition of the silyl enol ether **3fc**.

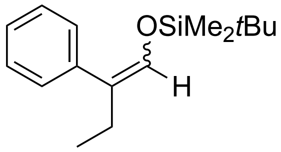

Z/*E*-**3fd**
[*E*:*Z* = 69:31]
$C_{16}H_{26}OSi$
M = 262.47 g/mol

***Z*/*E*-*tert*-butyldimethyl((2-phenylbut-1-en-1-yl)oxy)silane (3fd):** Prepared from 2-phenylbut-1-en-1-one (**1f**, ~95% pure by ^{1}H NMR, 37 mg, 0.25 mmol, 1.0 equiv) according to **GP 7**. CH_2Br_2 (8.8 µL, 0.125 mmol, 0.50 equiv) was added as internal standard to determine the yield by NMR spectroscopy (68%). Purification by flash column chromatography on alumina using *n*-pentane as eluent afforded the silyl enol ether **3fd** (46 mg, 70%, *E*:*Z* = 72:28) combined with silyl ether as colorless oil.

R_f = 0.65 (1% ethyl acetate in cyclohexane).
IR (ATR): ṽ = 2929, 2857, 1638, 1462, 1253, 1167, 939, 832, 778, 694 cm^{-1}.
^{1}H NMR (500 MHz, $CDCl_3$, 298 K): δ = 0.12 (s, 2H), 0.19 (s, 6H), 0.88 (s, 4H), 0.97 (s, 9H), 0.98–1.01 (m, 5H), 2.33 (qd, *J* = 7.4, 1.2 H, 1H), 2.55 (q, *J* = 7.5, 0.6 Hz, 2H), 6.39 (t, *J* = 1.1 Hz, 0.4H), 6.55 (s, 1H), 7.15–7.21 (m, 2H), 7.27–7.32 (m, 5H), 7.52–7.54 (m, 1H) ppm.
$^{13}C\{^{1}H\}$ NMR (126 MHz, $CDCl_3$, 298 K): δ = –5.1, 13.1, 18.4, 20.2, 25.8, 124.9, 126.0, 126.1, 128.4, 137.2, 140.2 ppm. (for *E*-isomer)
^{29}Si DEPT NMR (99 MHz, $CDCl_3$, 298 K, optimized for *J* = 7 Hz): δ = 22.4, 22.5 ppm. (for *Z*- and *E*-isomer respectively)
HRMS (APCI): calculated for $C_{16}H_{26}OSi^{+\bullet}$ $[M]^{+\bullet}$: 262.1753; found 262.1747.

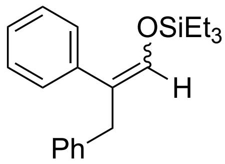

Z/*E*-**3ga**
[*Z*:*E* = 82:18]
$C_{21}H_{28}OSi$
M = 324.54 g/mol

***Z*/*E*-((2,3-diphenylprop-1-en-1-yl)oxy)triethylsilane (3ga):** Prepared from 2,3-diphenylprop-1-en-1-one (**1g**, 42 mg, 0.20 mmol, 1.0 equiv) according to **GP 7**. CH_2Br_2 (7.0 µL, 0.10 mmol, 0.50 equiv) was added as internal standard to determine the yield by NMR spectroscopy (93%). Purification by flash column chromatography on alumina using *n*-pentane as eluent afforded the silyl enol ether **3ga** (26 mg, 40%, *Z*:*E* = 72:28) as colorless oil.

R_f = 0.45 (1% ethyl acetate in cyclohexane).
IR (ATR): ṽ = 2954, 2875, 1634, 1493, 1453, 1238, 1159, 1006, 831, 727, 693 cm^{-1}.
^{1}H NMR (500 MHz, $CDCl_3$, 298 K): δ = 0.67–0.74 (m, 9H), 0.97–1.02 (m, 13H), 3.64 (s, 2H), 3.90 (s, 0.8H), 6.50 (s, 1H), 6.85 (s, 0.4H), 7.09–7.15 (m, 3H), 7.21–7.26 (m, 9H), 7.55–7.57 (m, 2H) ppm.
^{13}C{^{1}H} NMR (126 MHz, $CDCl_3$, 298 K): δ = 4.6, 6.7, 38.6, 117.6, 125.6, 125.9, 126.0, 127.8, 128.4, 128.5, 137.6, 139.3, 141.1 ppm. (for *Z*-isomer)
^{29}Si DEPT NMR (99 MHz, $CDCl_3$, 298 K, optimized for *J* = 7 Hz): δ = 22.9 ppm.
HRMS (APCI): calculated for $C_{21}H_{28}OSi^{+\bullet}$ $[M]^{+\bullet}$: 324.1909; found 324.1900.

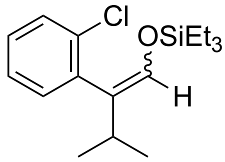

Z/*E*-**3ha**
[*Z*:*E* = 76:24]
$C_{17}H_{27}ClOSi$
M = 310.94 g/mol

***Z*/*E*-((2-(2-chlorophenyl)-3-methylbut-1-en-1-yl)oxy)triethylsilane (3ha):** Prepared from 2-(2-chlorophenyl)-3-methylbut-1-en-1-one (**1h**, ~70% pure by ^{1}H NMR, 39 mg, 0.20 mmol, 1.0 equiv) according to **GP 7**. CH_2Br_2 (7.0 µL, 0.10 mmol, 0.50 equiv) was added as internal standard to determine the yield by NMR spectroscopy (49%). Purification by flash column chromatography on alumina using *n*-pentane as eluent afforded the silyl enol ether **3ha** (39 mg, 63%, *Z*:*E* = 73:27) combined with silyl ether as colorless oil.

R_f = 0.61 (1% ethyl acetate in cyclohexane).

IR (ATR): ṽ = 2955, 2875, 1647, 1461, 1320, 1239, 1178, 1154, 1110, 1004, 975, 803, 727 cm^{-1}.

^{1}H NMR (500 MHz, $CDCl_3$, 298 K): δ = 0.70 (q, *J* = 7.9 Hz, 6H), 1.02 (t, *J* = 7.9 Hz, 9H), 1.08 (d, *J* = 7.0 Hz, 6H), 3.07 (sept, *J* = 7.0 Hz, 1H), 6.11 (s, 1H), 6.33 (d, *J* = 1.2 Hz, 0.1H), 7.07–7.21 (m, 3H), 7.32–7.42 (m, 1H) ppm. (for *E*-isomer)

^{13}C{^{1}H} NMR (126 MHz, $CDCl_3$, 298 K): δ = 4.7, 6.7, 21.2, 28.7, 125.9, 126.1, 127.9, 129.6, 132.9, 135.6, 138.5, 138.8 ppm. (for *E*-isomer)

^{29}Si DEPT NMR (99 MHz, $CDCl_3$, 298 K, optimized for *J* = 7 Hz): δ = 21.9 ppm.

HRMS (APCI): calculated for $C_{21}H_{28}OSi^{+\bullet}$ $[M]^{+\bullet}$: 324.1909; found 324.1900.

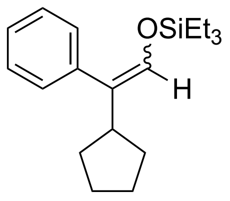

Z/*E*-**3ia**
[*Z*:*E* = 93:7]
$C_{19}H_{30}OSi$
M = 302.53 g/mol

***Z*/*E*-((2-cyclopentyl-2-phenylvinyl)oxy)triethylsilane (3ia):** Prepared from 2-cyclopentyl-2-phenylethen-1-one (**1i**, ~80% pure by ^{1}H NMR, 75 mg, 0.40 mmol, 1.0 equiv) according to **GP 7**. CH_2Br_2 (14 µL, 0.20 mmol, 0.50 equiv) was added as internal standard to determine the yield by NMR spectroscopy (76%). Purification by flash column chromatography on alumina using *n*-pentane as eluent afforded the silyl enol ether **3ia** (102 mg, 84%, *Z*:*E* = 77:23) combined with silyl ether as colorless oil.

R_f = 0.66 (1% ethyl acetate in cyclohexane).

IR (ATR): ṽ = 2956, 2876, 1653, 1459, 1237, 1170, 1004, 815, 728 cm^{-1}.

^{1}H NMR (500 MHz, $CDCl_3$, 298 K): δ = 0.59 (q, *J* = 7.9 Hz, 6H), 0.70 (q, *J* = 7.9 Hz, 2H), 0.90 (t, *J* = 7.9 Hz, 9H), 1.01 (t, *J* = 7.9 Hz, 3H), 1.29–1.37 (m, 2H), 1.54–1.67 (m, 7H), 1.75–1.83 (m, 3H), 2.69–2.77 (m, 1H), 3.07–3.14 (m, 0.3H), 6.32 (s, 0.3H), 6.36 (d, *J* = 1.4 Hz, 1H), 7.15–7.21 (m, 2H), 7.25–7.29 (m, 3H), 7.32–7.34 (m, 2H) ppm.

^{13}C{^{1}H} NMR (126 MHz, $CDCl_3$, 298 K): δ = 4.6, 6.6, 24.6, 32.2, 43.4, 124.8, 125.8, 127.6, 129.2, 135.0, 139.1 ppm. (for *Z*-isomer)

^{29}Si DEPT NMR (99 MHz, $CDCl_3$, 298 K, optimized for *J* = 7 Hz): δ = 21.2 ppm

HRMS (APCI): calculated for $C_{19}H_{30}OSi^{+\bullet}$ $[M]^{+\bullet}$: 302.2066; found 302.2061.

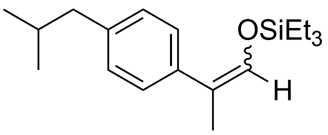

Z/*E*-**3ja**
[*Z*:*E* = 79:21]
$C_{19}H_{32}OSi$
M = 304.55 g/mol

***Z*/*E*-triethyl((2-(4-isobutylphenyl)prop-1-en-1-yl)oxy)silane (3ja):** Prepared from 2-(4-isobutylphenyl)prop-1-en-1-one (**1j**, 75 mg, 0.40 mmol, 1.0 equiv) according to **GP 7**. CH_2Br_2 (14 µL, 0.20 mmol, 0.50 equiv) was added as internal standard to determine the yield by NMR spectroscopy (95%). Purification by flash column chromatography on alumina using *n*-pentane as eluent afforded the silyl enol ether **3ja** (104 mg, 85%, *Z*:*E* = 89:11) as colorless oil.

R*f* = 0.59 (1% ethyl acetate in cyclohexane).
IR (ATR): ṽ = 2953, 2875, 1637, 1509, 1459, 1253, 1164, 1121, 1001, 820, 728 cm^{-1}.
^{1}H NMR (500 MHz, $CDCl_3$, 298 K): δ = 0.70 (q, *J* = 7.9 Hz, 6H), 0.91 (dd, *J* = 6.6, 1.0 Hz, 6H), 0.99 (t, *J* = 7.9 Hz, 9H), 1.83–1.88 (m, 1H), 1.90 (t, *J* = 1.2 Hz, 3H), 2.46 (d, *J* = 7.1 Hz, 2H), 6.42 (t, *J* = 1.2 Hz, 1H), 7.10 (d, *J* = 8.1 Hz, 2H), 7.60 (d, *J* = 8.3 Hz, 2H) ppm. (for *Z*-isomers)
^{13}C{^{1}H} NMR (126 MHz, $CDCl_3$, 298 K): δ = 4.6, 6.7, 18.3, 22.6, 30.4, 45.3, 113.6, 127.4, 128.6, 136.1, 136.5, 139.3 ppm. (for *Z*-isomers)
^{29}Si DEPT NMR (99 MHz, $CDCl_3$, 298 K, optimized for *J* = 7 Hz): δ = 22.2 ppm.
HRMS (APCI): calculated for $C_{19}H_{32}OSi^{+\bullet}$ $[M]^{+\bullet}$: 304.2222; found 304.2215.

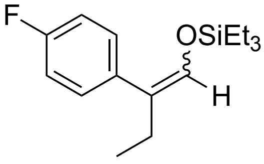

Z/*E*-**3ka**
[*Z*:*E* = 77:23]
$C_{16}H_{25}FOSi$
M = 280.46 g/mol

***Z*/*E*-triethyl((2-(4-fluorophenyl)but-1-en-1-yl)oxy)silane (3ka):** Prepared from 2-(4-fluorophenyl)but-1-en-1-one (**1k**, 66 mg, 0.40 mmol, 1.0 equiv) according to **GP 7**. CH_2Br_2 (14 µL, 0.20 mmol, 0.50 equiv) was added as internal standard to determine the yield by NMR spectroscopy (quant.). Purification by flash column chromatography on alumina using *n*-pentane as eluent afforded the silyl enol ether **3ka** (99 mg, 88%, *Z*:*E* = 77:23) as colorless oil.

R*f* = 0.67 (1% ethyl acetate in cyclohexane).
IR (ATR): ṽ = 2957, 2876, 1639, 1507, 1459, 1229, 1166, 1005, 809, 727 cm^{-1}.
^{1}H NMR (500 MHz, $CDCl_3$, 298 K): δ = 0.66 (q, *J* = 7.9 Hz, 6H), 0.72 (q, *J* = 7.9 Hz, 2H), 0.96 (t, *J* = 7.8 Hz, 9H), 0.97–1.00 (m, 4H), 1.02 (t, *J* = 7.9 Hz, 3H), 2.30 (qd, *J* = 7.4, 1.2 Hz, 2H),

2.53 (q, J = 7.5 Hz, 1H), 6.40 (s, 1H), 6.50 (s, 0.3H), 6.96–7.01 (m, 2H), 7.20–7.23 (m, 1H), 7.49–7.53 (m_c, 2H) ppm.

^{13}C{^{1}H} NMR (126 MHz, $CDCl_3$, 298 K): δ = 4.6, 6.6, 14.4, 25.7, 114.6 ($J_{C–F}$ = 20.9 Hz), 120.5, 129.9 ($J_{C–F}$ = 7.8 Hz), 133.7 ($J_{C–F}$ = 3.3 Hz), 135.9, 161.1 ($J_{C–F}$ = 244.2 Hz) ppm. (for *Z*-isomer)

^{19}F{^{1}H} NMR (471 MHz, $CDCl_3$, 298 K): δ = –117.0, –117.7 ppm. (for *Z*- and *E*- isomer respectively)

^{29}Si DEPT NMR (99 MHz, $CDCl_3$, 298 K, optimized for J = 7 Hz): δ = 22.2 ppm.

HRMS (APCI): calculated for $C_{16}H_{25}FOSi^{+•}$ $[M]^{+•}$: 280.1659; found 280.1651.

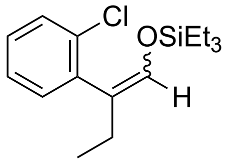

Z/*E*-**3la**
[*Z*:*E* = 79:21]
$C_{16}H_{25}ClOSi$
M = 296.91 g/mol

***Z*/*E*-((2-(2-chlorophenyl)but-1-en-1-yl)oxy)triethylsilane (3la):** Prepared from 2-(2-chlorophenyl)but-1-en-1-one (**1l**, 72 mg, 0.40 mmol, 1.0 equiv) according to **GP 7**. CH_2Br_2 (14 µL, 0.20 mmol, 0.50 equiv) was added as internal standard to determine the yield by NMR spectroscopy (quant.). Purification by flash column chromatography on alumina using *n*-pentane as eluent afforded the silyl enol ether **3la** (111 mg, 93%, *Z*:*E* = 75:25) as colorless oil.

R_f = 0.63 (1% ethyl acetate in cyclohexane).

IR (ATR): ṽ = 2956, 2876, 1655, 1458, 1432, 1237, 1170, 1004, 814, 728 cm^{-1}.

^{1}H NMR (500 MHz, $CDCl_3$, 298 K): δ = 0.56 (q, J = 8.1 Hz, 6H), 0.71 (q, J = 7.9 Hz, 2H), 0.86 (t, J = 7.9 Hz, 9H), 0.91–0.95 (m, 4H), 1.02 (t, J = 7.9 Hz, 3H), 2.27 (qd, J = 7.4, 1.2 Hz, 2H), 2.53 (qd, J = 7.5, 0.9 Hz, 1H), 6.25 (s, 0.3H), 6.35 (t, J = 1.2 Hz, 1H), 7.13–7.21 (m, 4H), 7.35–7.37 (m, 1H) ppm.

^{13}C{^{1}H} NMR (126 MHz, $CDCl_3$, 298 K): δ = 4.6, 4.7, 6.5, 6.7, 12.4, 13.7, 21.2, 25.9, 122.0, 123.9, 126.1, 126.4, 127.6, 127.9, 129.3, 129.6, 131.8, 132.1, 133.9, 134.6, 136.0, 138.0, 138.6, 139.3 ppm.

^{29}Si DEPT NMR (99 MHz, $CDCl_3$, 298 K, optimized for J = 7 Hz): δ = 21.0 ppm.

HRMS (APCI): calculated for $C_{16}H_{25}ClOSi^{+•}$ $[M]^{+•}$: 296.1363; found 296.1357.

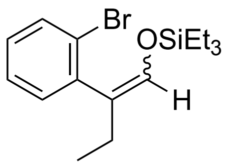

Z/*E*-**3ma**
[*Z*:*E* = 85:15]
$C_{16}H_{25}BrOSi$
M = 341.36 g/mol

***Z*/*E*-((2-(2-bromophenyl)but-1-en-1-yl)oxy)triethylsilane (3ma):** Prepared from 2-(2-bromophenyl)but-1-en-1-one (**1m**, 90 mg, 0.40 mmol, 1.0 equiv) according to **GP 7**. CH_2Br_2 (14 µL, 0.20 mmol, 0.50 equiv) was added as internal standard to determine the yield by NMR spectroscopy (quant.). Purification by flash column chromatography on alumina using *n*-pentane as eluent afforded the silyl enol ether **3ma** (124 mg, 91%, *Z*:*E* = 83:17) as colorless oil.

R*f* = 0.70 (1% ethyl acetate in cyclohexane).
IR (ATR): ṽ = 2956, 2875, 1653, 1459, 1237, 1169, 1004, 940, 814, 726 cm^{-1}.
^{1}H NMR (500 MHz, $CDCl_3$, 298 K): δ = 0.56 (q, *J* = 8.0 Hz, 6H), 0.71 (q, *J* = 8.0 Hz, 1H), 0.86 (t, *J* = 8.0 Hz, 9H), 0.94 (t, *J* = 7.5 Hz, 3H), 1.02 (t, *J* = 8.0 Hz, 2H), 2.27 (qd, *J* = 7.4, 1.2 Hz, 2H), 2.52 (q, *J* = 7.5, 0.8 Hz, 1H), 6.23 (s, 0.2H), 6.32 (t, *J* = 1.2 Hz, 1H), 7.05–7.09 (m, 1H), 7.10–7.16 (m, 1H), 7.21–7.25 (m, 1H), 7.55 (dd, *J* = 8.0, 1.2 Hz, 1H) ppm.
^{13}C{^{1}H} NMR (126 MHz, $CDCl_3$, 298 K): δ = 4.7, 6.6, 13.6, 26.0, 123.5, 124.2, 126.7, 127.9, 131.9, 132.5, 135.8, 140.1 ppm. (for *Z*-isomer)
^{29}Si DEPT NMR (99 MHz, $CDCl_3$, 298 K, optimized for *J* = 7 Hz): δ = 21.0 ppm.
HRMS (APCI): calculated for $C_{16}H_{25}BrOSi^{+\bullet}$ $[M]^{+\bullet}$: 340.0858; found 340.0854.

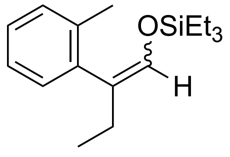

Z/*E*-**3na**
[*Z*:*E* = 91:9]
$C_{17}H_{28}OSi$
M = 276.50 g/mol

***Z*/*E*-triethyl((2-(*o*-tolyl)but-1-en-1-yl)oxy)silane (3na):** Prepared from 2-(*o*-tolyl)but-1-en-1-one (**1n**, 64 mg, 0.40 mmol, 1.0 equiv) according to **GP 7**. CH_2Br_2 (14 µL, 0.20 mmol, 0.50 equiv) was added as internal standard to determine the yield by NMR spectroscopy (91%). Purification by flash column chromatography on alumina using *n*-pentane as eluent afforded the silyl enol ether **3na** (90 mg, 81%, *Z*:*E* = 91:9) as colorless oil.

R*f* = 0.66 (1% ethyl acetate in cyclohexane).

IR (ATR): ṽ = 2956, 2876, 1652, 1457, 1239, 1164, 1004, 820, 725 cm^{-1}.

^{1}H NMR (500 MHz, $CDCl_3$, 298 K): δ = 0.54 (q, *J* = 8.0 Hz, 6H), 0.85 (t, *J* = 8.0 Hz, 9H), 0.93 (t, *J* = 7.4 Hz, 3H), 2.22 (qd, *J* = 7.5, 1.3 Hz, 2H), 2.26 (s, 3H), 6.31 (t, *J* = 1.2 Hz, 1H), 7.05–7.07 (m, 1H), 7.11–7.13 (m, 2H), 7.15–7.18 (m, 1H) ppm. (for *Z*-isomer)

^{13}C{^{1}H} NMR (126 MHz, $CDCl_3$, 298 K): δ = 4.6, 6.6, 13.7, 19.9, 27.3, 123.3, 125.1, 126.2, 129.1, 129.5, 134.5, 137.0, 138.7 ppm. (for *Z*-isomer)

^{29}Si DEPT NMR (99 MHz, $CDCl_3$, 298 K, optimized for *J* = 7 Hz): δ = 20.5, 21.4 ppm. (for *Z*- and *E*-isomer respectively)

HRMS (APCI): calculated for $C_{17}H_{28}OSi^{+\bullet}$ $[M]^{+\bullet}$: 276.1909; found 276.1903.

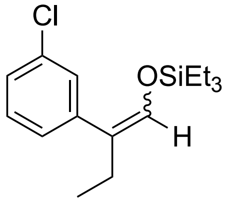

Z/*E*-**3oa**
[*Z*:*E* = 67:33]
$C_{16}H_{25}ClOSi$
M = 296.91 g/mol

***Z*/*E*-((2-(3-chlorophenyl)but-1-en-1-yl)oxy)triethylsilane (3oa):** Prepared from 2-(3-chlorophenyl)but-1-en-1-one (**1o**, 72 mg, 0.40 mmol, 1.0 equiv) according to **GP 7**. CH_2Br_2 (14 µL, 0.20 mmol, 0.50 equiv) was added as internal standard to determine the yield by NMR spectroscopy (quant.). Purification by flash column chromatography on alumina using *n*-pentane as eluent afforded the silyl enol ether **3oa** (96 mg, 81%, *Z*:*E* = 67:33) as colorless oil.

R*f* = 0.69 (1% ethyl acetate in cyclohexane).

IR (ATR): ṽ = 2957, 2875, 1634, 1591, 1459, 1412, 1240, 1168, 1004, 952, 804, 778, 729, 691 cm^{-1}.

^{1}H NMR (500 MHz, $CDCl_3$, 298 K): δ = 0.65–0.75 (m, 9H), 0.96–1.04 (m, 18H), 2.31 (qd, *J* = 7.4, 1.1 Hz, 2H), 2.53 (q, *J* = 7.5 Hz, 1H), 6.44 (s, 1H), 6.59 (s, 0.5H), 7.13–7.16 (m, 2H), 7.19–7.25 (m, 2H), 7.32 (td, *J* = 7.9, 1.3 Hz, 1H), 7.63 (dd, *J* = 2.8, 1.0 Hz, 1H) ppm.

^{13}C{^{1}H} NMR (126 MHz, $CDCl_3$, 298 K): δ = 4.6, 4.7, 6.6, 6.7, 13.0, 14.4, 20.0, 25.4, 120.0, 123.9, 124.1, 125.8, 125.9, 126.0, 126.1, 128.7, 129.0, 129.6, 133.8, 134.3, 137.2, 138.0, 139.6, 142.3 ppm.

^{29}Si DEPT NMR (99 MHz, $CDCl_3$, 298 K, optimized for *J* = 7 Hz): δ = 22.7 (2Si) ppm.

HRMS (APCI): calculated for $C_{16}H_{25}ClOSi^{+\bullet}$ $[M]^{+\bullet}$: 296.1363; found 296.1361.

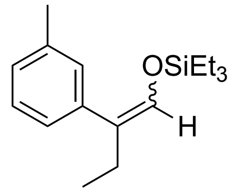

Z/*E*-**3pa**
[*Z*:*E* = 80:20]
$C_{17}H_{28}OSi$
M = 276.50 g/mol

***Z*/*E*-triethyl((2-(*m*-tolyl)but-1-en-1-yl)oxy)silane (3pa):** Prepared from 2-(*m*-tolyl)but-1-en-1-one (**1p**, 64 mg, 0.40 mmol, 1.0 equiv) according to **GP 7**. CH_2Br_2 (14 µL, 0.20 mmol, 0.50 equiv) was added as internal standard to determine the yield by NMR spectroscopy (quant.). Purification by flash column chromatography on alumina using *n*-pentane as eluent afforded the silyl enol ether **3pa** (93 mg, 84%, *Z*:*E* = 77:23) as colorless oil.

R*f* = 0.64 (1% ethyl acetate in cyclohexane).
IR (ATR): ṽ = 2965, 2912, 2875, 1636, 1458, 1260, 1161 ,1004, 955, 812, 780, 728 cm^{-1}.
^{1}H NMR (500 MHz, $CDCl_3$, 298 K): δ = 0.67 (q, *J* = 7.9 Hz, 6H), 0.72 (q, *J* = 8.0 Hz, 2H), 0.96–1.05 (m, 16H), 2.31–2.36 (m, 6H), 2.55 (q, *J* = 7.6 Hz, 1H), 6.41 (s, 1H), 6.57 (s, 0.3H), 6.99–7.10 (m, 2H), 7.17–7.22 (m, 1H), 7.32 (d, *J* = 7.5 Hz, 1H), 7.42 (s, 1H) ppm.
^{13}C{^{1}H} NMR (126 MHz, $CDCl_3$, 298 K): δ = 4.6, 4.7, 6.7 (2C), 13.1, 14.6, 20.2, 21.7 (2C), 25.7, 121.4, 123.3, 125.1, 125.3, 126.6, 126.8, 126.9, 127.7, 128.3, 129.3, 136.0, 137.0, 137.1, 137.7, 137.9, 140.2 ppm.
^{29}Si DEPT NMR (99 MHz, $CDCl_3$, 298 K, optimized for *J* = 7 Hz): δ = 21.8 ppm.
HRMS (APCI): calculated for $C_{17}H_{28}Si^{+•}$ $[M]^{+•}$: 276.1902; found 276.1909.

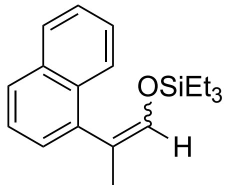

Z/*E*-**3qa**
[*Z*:*E* = 93:7]
$C_{20}H_{28}OSi$
M = 312.53 g/mol

***Z*/*E*-triethyl((2-(naphthalen-1-yl)but-1-en-1-yl)oxy)silane (3qa):** Prepared from 2-(naphthalen-1-yl)but-1-en-1-one (**1q**, 39 mg, 0.20 mmol, 1.0 equiv) according to **GP 7**. CH_2Br_2 (7.0 µL, 0.10 mmol, 0.50 equiv) was added as internal standard to determine the yield by NMR spectroscopy (77%). Purification by flash column chromatography on alumina using *n*-pentane as eluent afforded the silyl enol ether **3qa** (40 mg, 64%, *Z*:*E* = 84:16) as colorless oil.

R*f* = 0.63 (1% ethyl acetate in cyclohexane).

IR (ATR): ṽ = 2955, 2874, 1651, 1458, 1237, 1166, 1001, 774, 727 cm^{-1}.

^{1}H NMR (500 MHz, $CDCl_3$, 298 K): δ = 0.45 (q, *J* = 8.0 Hz, 6H), 0.73 (t, *J* = 7.9 Hz, 9H), 0.95 (t, *J* = 7.3 Hz, 3H), 1.05 (t, *J* = 8.0 Hz, 2H), 2.38 (q, *J* = 7.4 Hz, 2H), 2.63 (q, *J* = 7.4 Hz, 0.4H), 6.34 (s, 0.2H), 6.54 (s, 1H), 7.26–7.29 (m, 1H), 7.42–7.47 (m, 3H), 7.73–7.76 (m, 1H), 7.82–7.84 (m, 1H), 7.93–7.95 (m, 1H) ppm.

^{13}C{^{1}H} NMR (126 MHz, $CDCl_3$, 298 K): δ = 4.5, 6.4, 14.1, 27.7, 122.2, 125.2, 125.3, 125.4, 126.2, 126.7, 126.8, 128.1, 132.0, 133.7, 135.7, 137.4 ppm. (for *Z*-isomers)

^{29}Si DEPT NMR (99 MHz, $CDCl_3$, 298 K, optimized for *J* = 7 Hz): δ = 20.6 ppm.

HRMS (APCI): calculated for $C_{20}H_{28}OSi^{+\bullet}$ $[M]^{+\bullet}$: 312.1909; found 312.1901.

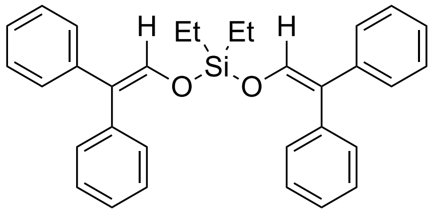

4af
$C_{32}H_{32}O_2Si$
M = 476.69 g/mol

Bis((2,2-diphenylvinyl)oxy)diethylsilane (4af): Prepared from 2,2-diphenylethen-1-one (**1a**, 39 mg, 0.2 mmol, 1.0 equiv) according to **GP 7**. Solvent and other volatiles were removed in vacuo under Schlenk line. Filtration through a small pad of Celite® layered with alumina using *n*-pentane afforded the silyl enol ether **4af** (41 mg, 43%) as colorless viscous oil.

R*f* = 0.25 (1% ethyl acetate in cyclohexane).

IR (ATR): ṽ = 2958, 2877, 1659, 1623, 1494, 1443, 1197, 1109, 1004, 954, 802, 745, 693 cm^{-1}.

^{1}H NMR (500 MHz, $CDCl_3$, 298 K): δ = 0.76–0.81 (m, 4H), 0.99–1.03 (m, 6H), 6.80 (s, 2H), 7.18–7.24 (m, 8H), 7.28–7.32 (m, 8H), 7.39–7.41 (m, 4H) ppm.

^{13}C{^{1}H} NMR (126 MHz, $CDCl_3$, 298 K): δ = 4.3, 6.0, 125.7, 126.7, 126.8, 127.9, 128.4, 128.6, 130.2, 137.0, 137.6, 140.4 ppm.

^{29}Si DEPT NMR (99 MHz, $CDCl_3$, 298 K, optimized for *J* = 7 Hz): δ = –1.8 ppm.

HRMS (APCI): calculated for $C_{32}H_{32}O_2Si^{+\bullet}$ $[M]^{+\bullet}$: 476.2172; found 476.2167.

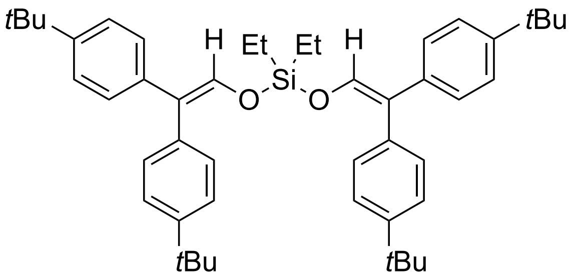

4cf
$C_{48}H_{64}O_2Si$
M = 701.12 g/mol

Bis((2,2-bis(4-(*tert*-butyl)phenyl)vinyl)oxy)diethylsilane (4cf): Prepared from 2,2-bis(4-(*tert*-butyl)phenyl)ethen-1-one (**1c**, 61 mg, 0.20 mmol, 1.0 equiv) according to **GP 7**. Solvent and other volatiles were removed in vacuo under Schlenk line. Filtration through a small pad of Celite® layered with alumina using *n*-pentane afforded the silyl enol ether **4cf** (70 mg, 50%) as colorless viscous oil.

R*f* = 0.15 (1% ethyl acetate in cyclohexane).
IR (ATR): ṽ = 2957, 1622, 1512, 1460, 1361, 1210, 1115, 1007, 961, 907, 833, 730 cm^{-1}.
^{1}H NMR (500 MHz, $CDCl_3$, 298 K): δ = 0.77 (q, *J* = 7.9 Hz, 4H), 0.99 (t, *J* = 7.9 Hz, 6H), 1.32 (s, 18H), 1.33 (s, 18H), 6.77 (s, 2H), 7.14–7.16 (m, 4H), 7.29–7.31 (m, 8H), 7.35–7.34 (m, 4H) ppm.
^{13}C{^{1}H} NMR (126 MHz, $CDCl_3$, 298 K): δ = 4.3, 6.1, 31.5 (2C), 34.6 (2C), 124.7, 125.0, 125.2, 128.4, 129.7, 134.7, 136.6, 137.6, 149.4, 149.6 ppm.
^{29}Si DEPT NMR (99 MHz, $CDCl_3$, 298 K, optimized for *J* = 7 Hz): δ = –1.9 ppm.
HRMS (APCI): calculated for $C_{48}H_{64}O_2Si^{+•}$ $[M]^{+•}$: 700.4676; found 700.4678.

7 Gram-Scale Synthesis of Silyl Enol Ether 3aa

In an argon-filled glovebox, $B(C_6F_5)_3$ (51 mg, 0.10 mmol, 2.0 mol%) was dissolved in C_6H_5F (3 mL) in a GC vial, and the solution of 2,2-diphenylethen-1-one (**1a**, 0.97 g, 5.0 mmol, 1.0 equiv) in C_6H_5F (4 mL) was added dropwise at room temperature. Et_3SiH (0.96 mL, 6.0 mmol, 1.2 equiv) was then added dropwise by a syringe. The resulting mixture was maintained at this temperature for additional 16 h. The solvent and other volatiles were carefully removed in vacuo using Schlenk techniques. The crude oil was purified by flash column chromatography on alumina using *n*-pentane as eluent to afford the pure silyl enol ether **3aa** (1.31 g, 84%) as a colorless oil.

8 Experimental Details for the Attempted One-Pot Sequence

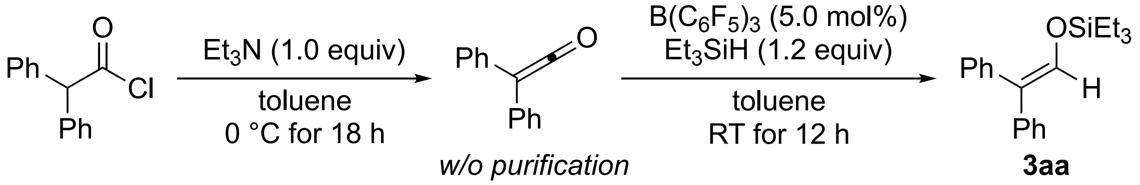

3aa

Run 1: Following **GP 5**, 2,2-diphenylacetyl chloride (115 mg, 0.500 mmol, 1.00 equiv) was dissolved in dry toluene (1 mL). The mixture was cooled to 0 °C and freshly distilled Et_3N (73.0 µL, 0.525 mmol, 1.05 equiv) was added dropwise by a microsyringe. The reaction mixture was maintained at 0 °C for 18 h and then warmed to room temperature. To this mixture, a solution of $B(C_6F_5)_3$ (12.8 mg, 0.025 mmol, 5.00 mol%) in toluene (1 mL) and Et_3SiH (96.0 µL, 0.600 mmol, 1.20 equiv) were added dropwise. The resulting mixture was maintained at this temperature for additional 12 h and filtered through a small pad of Celite®. CH_2Br_2 (17.5 µL, 0.250 mmol, 0.500 equiv) was added as internal standard to determine the yield by NMR spectroscopy (3%).

Run 2: Following **GP 5**, 2,2-diphenylacetyl chloride (115 mg, 0.500 mmol, 1.00 equiv) was dissolved in dry toluene (1 mL). The mixture was cooled to 0 °C and freshly distilled Et_3N (73.0 µL, 0.525 mmol, 1.05 equiv) was added dropwise by a microsyringe. The reaction mixture was maintained at 0 °C for 18 h and then warmed to room temperature. The white precipitate was filtered off using a syringe filter. The yellow filtrate was then directly added to a stirred solution of $B(C_6F_5)_3$ (12.8 mg, 0.025 mmol, 5.00 mol%) in toluene (1 mL) and Et_3SiH (96.0 µL, 0.600 mmol, 1.20 equiv). The resulting mixture was maintained at this temperature for additional 12 h. CH_2Br_2 (17.5 µL, 0.250 mmol, 0.500 equiv) was added as internal standard to determine the yield by NMR spectroscopy (8%).

9 NMR Spectra

Figure S1. ^{1}H NMR spectrum (500 MHz, CD_2Cl_2, 298 K) of **1b**.

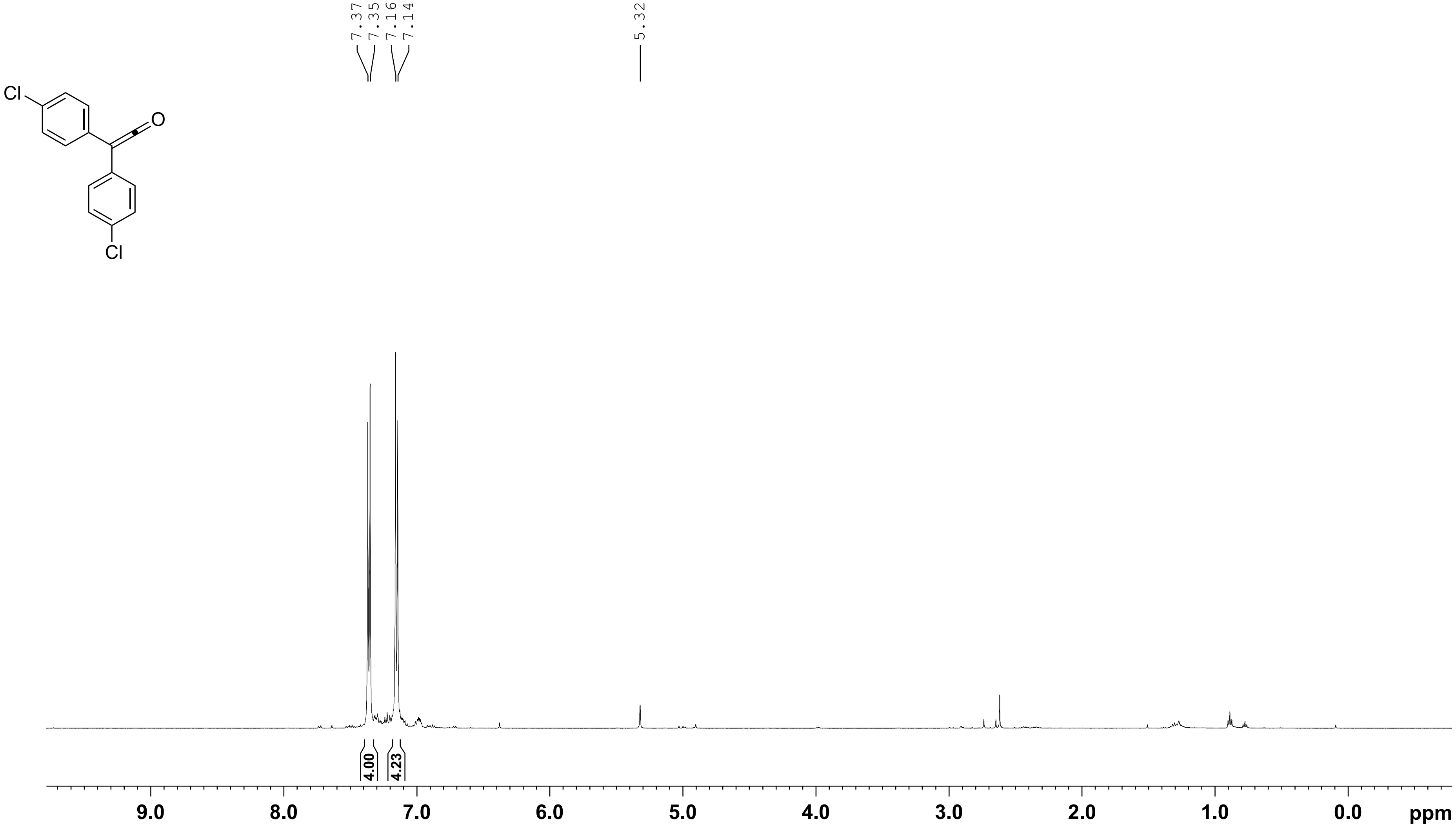

Figure S2. $^{13}C\{^{1}H\}$ NMR spectrum (126 MHz, CD_2Cl_2, 298 K) of **1b**.

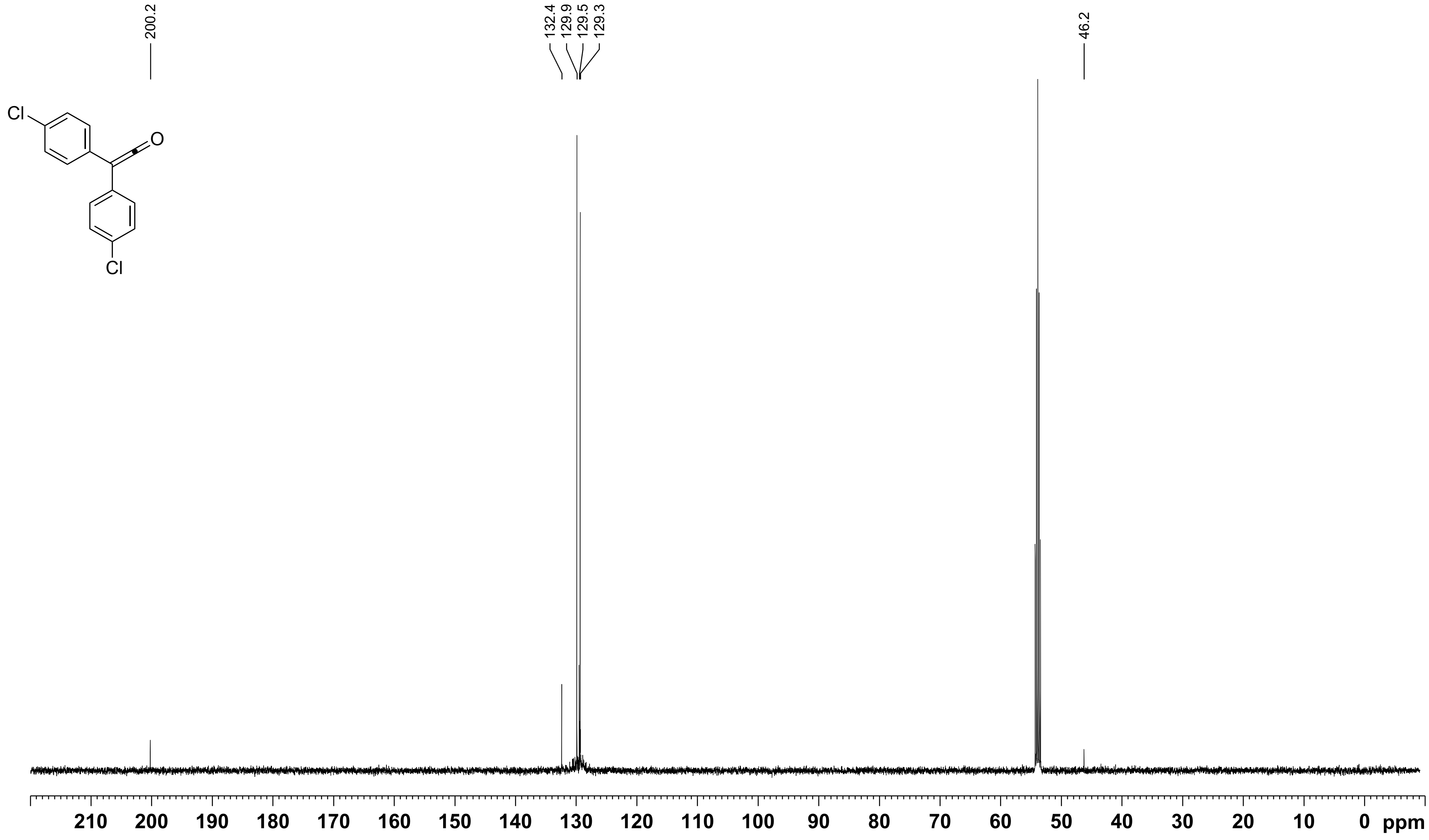

Figure S3. ^{1}H NMR spectrum (500 MHz, CD_2Cl_2, 298 K) of **1c**.

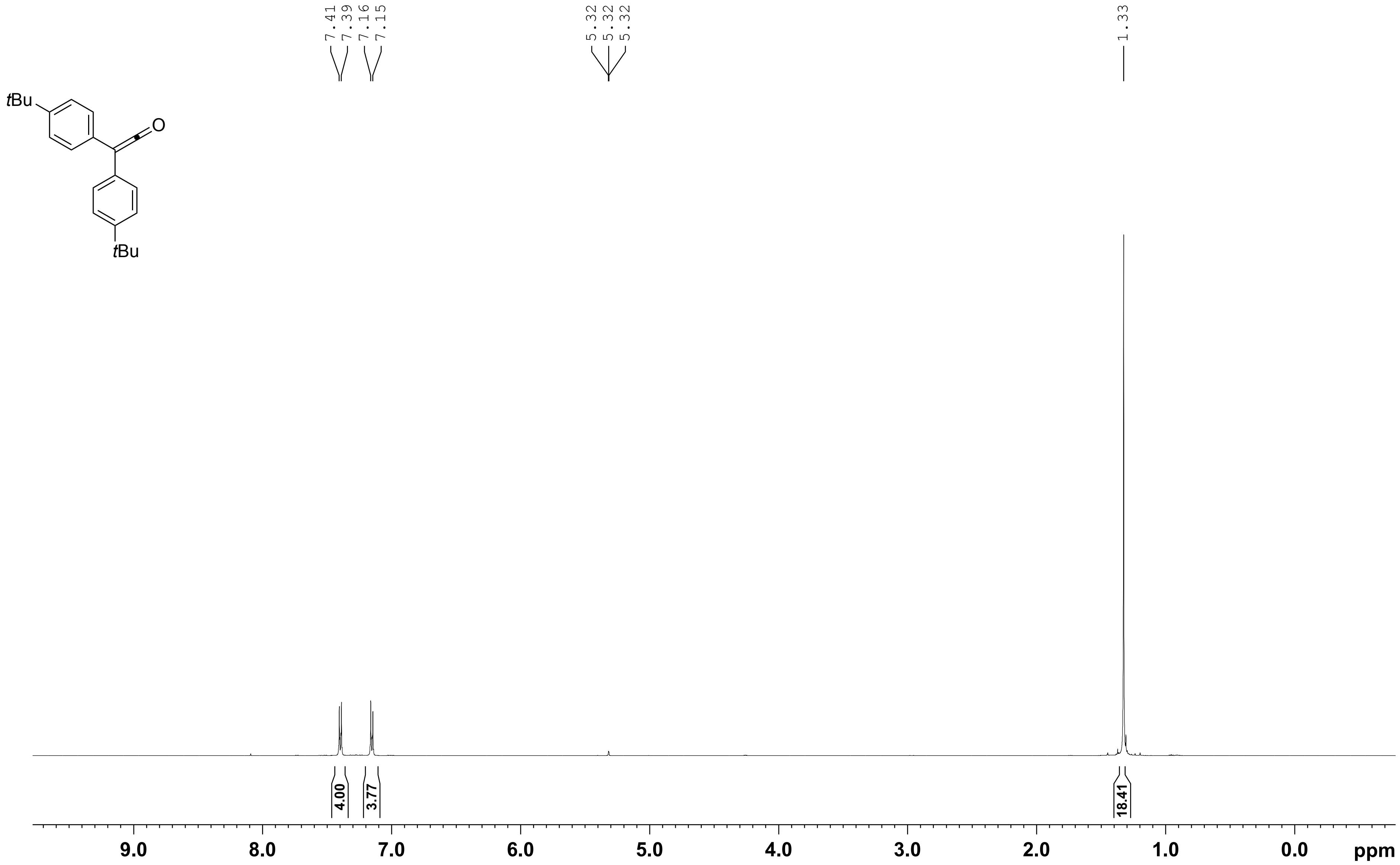

Figure S4. $^{13}C\{^1H\}$ NMR spectrum (126 MHz, CD_2Cl_2, 298 K) of **1c**.

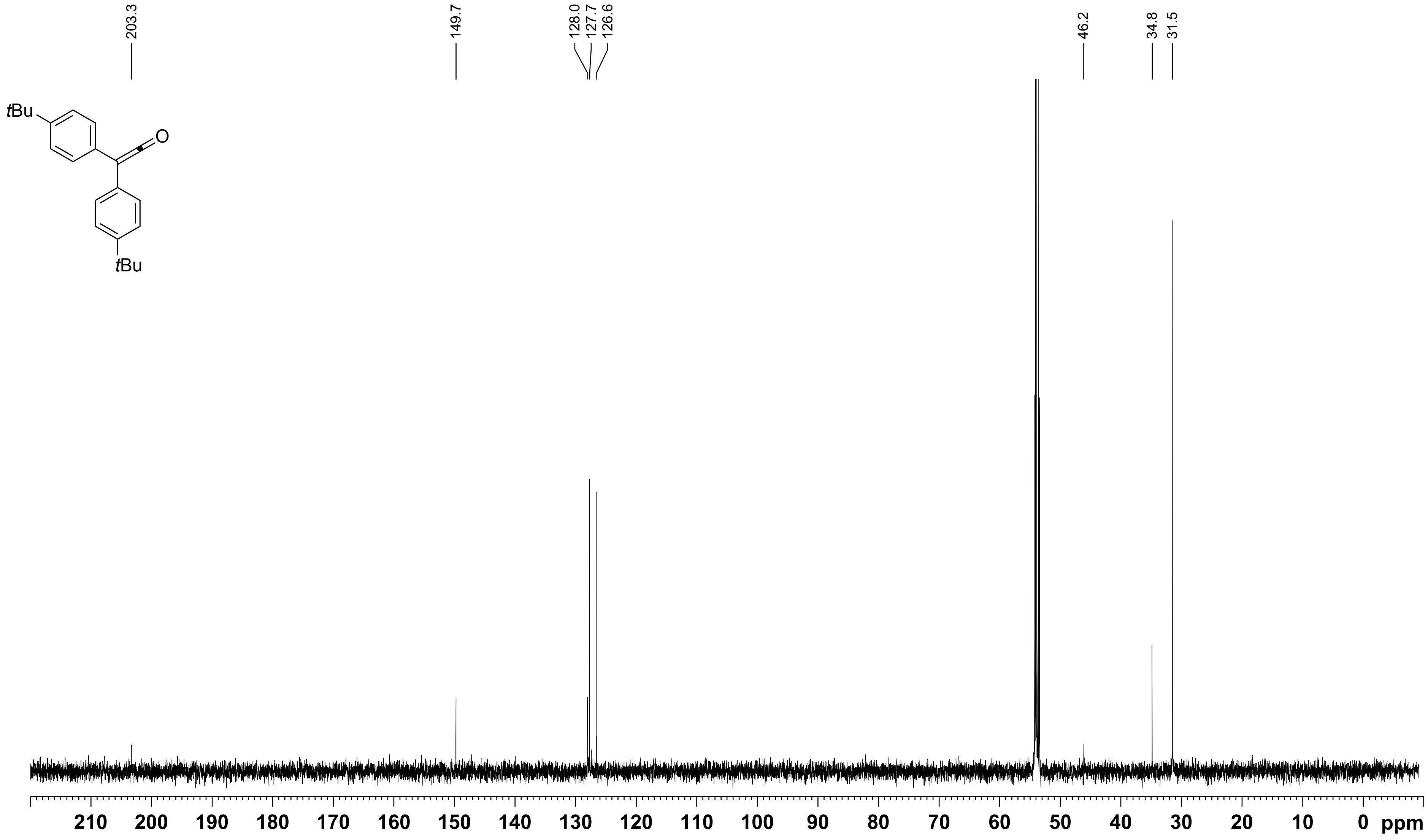

Figure S5. ^{1}H NMR spectrum (500 MHz, $CDCl_3$, 298 K) of **1d**. (# = *n*-pentane)

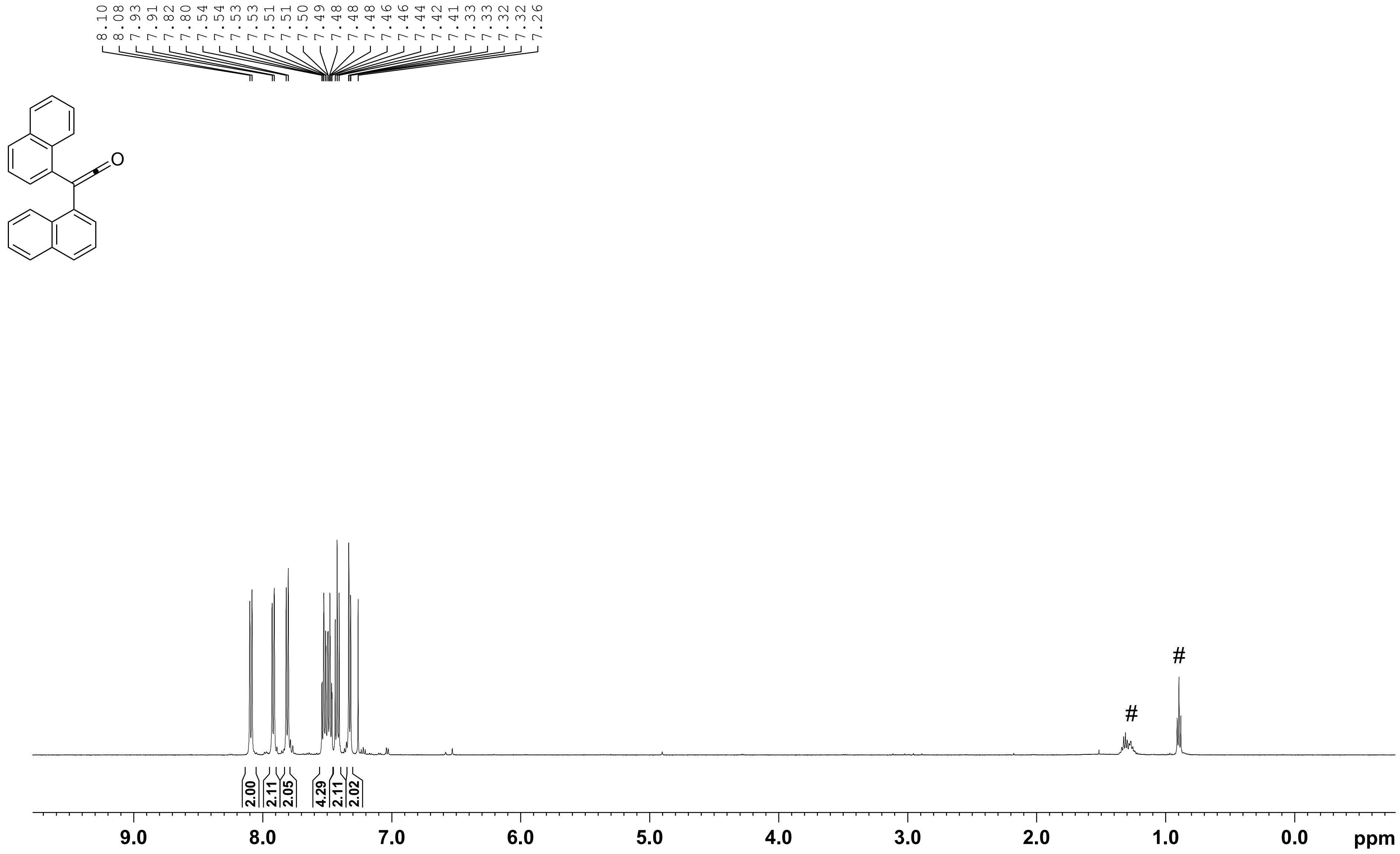

Figure S6. $^{13}C\{^1H\}$ NMR spectrum (126 MHz, $CDCl_3$, 298 K) of **1d**.

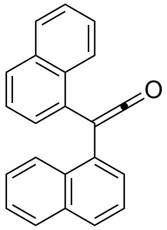

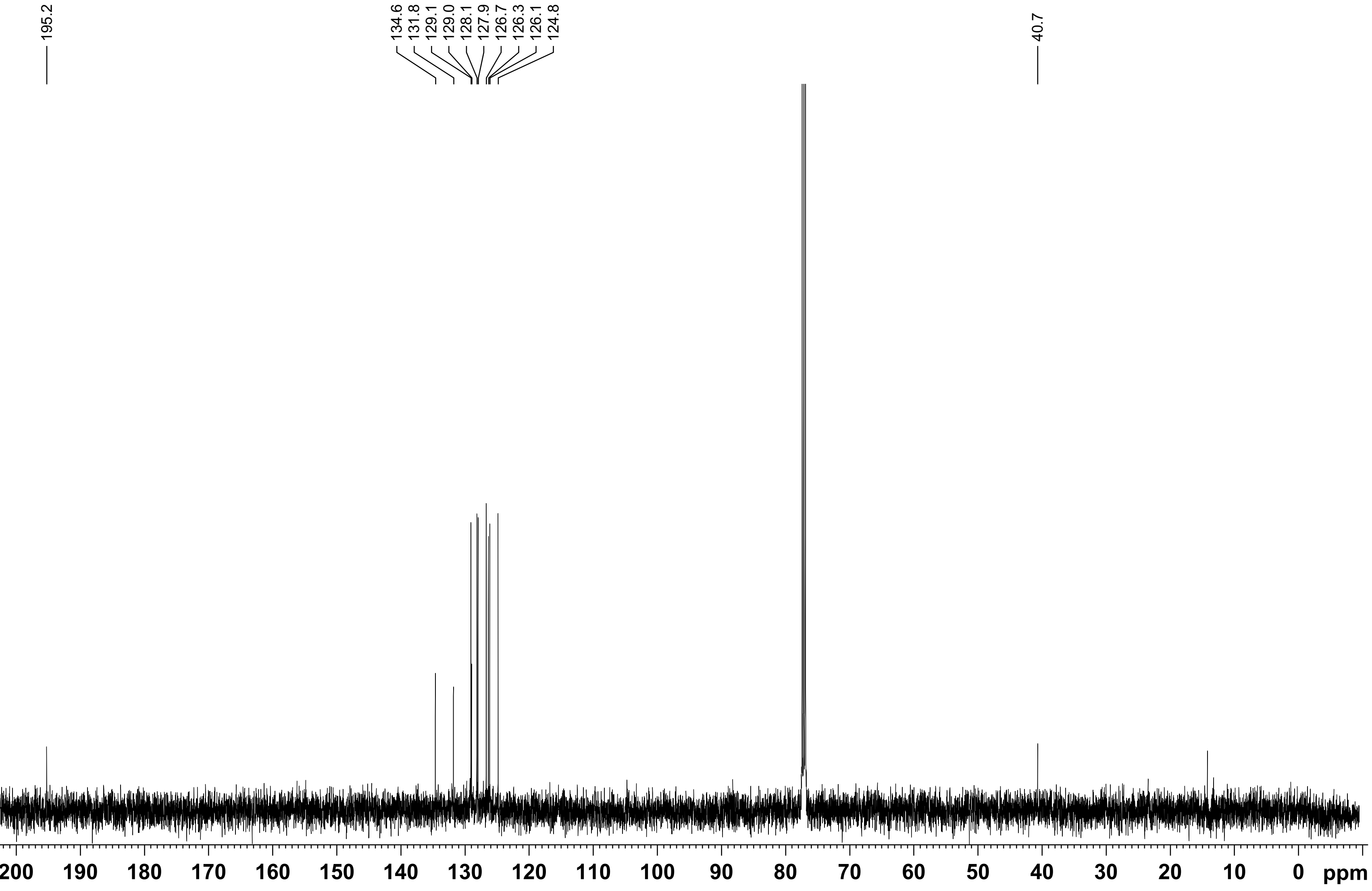

Figure S7. ^{1}H NMR spectrum (500 MHz, CD_2Cl_2, 298 K) of **1e**.

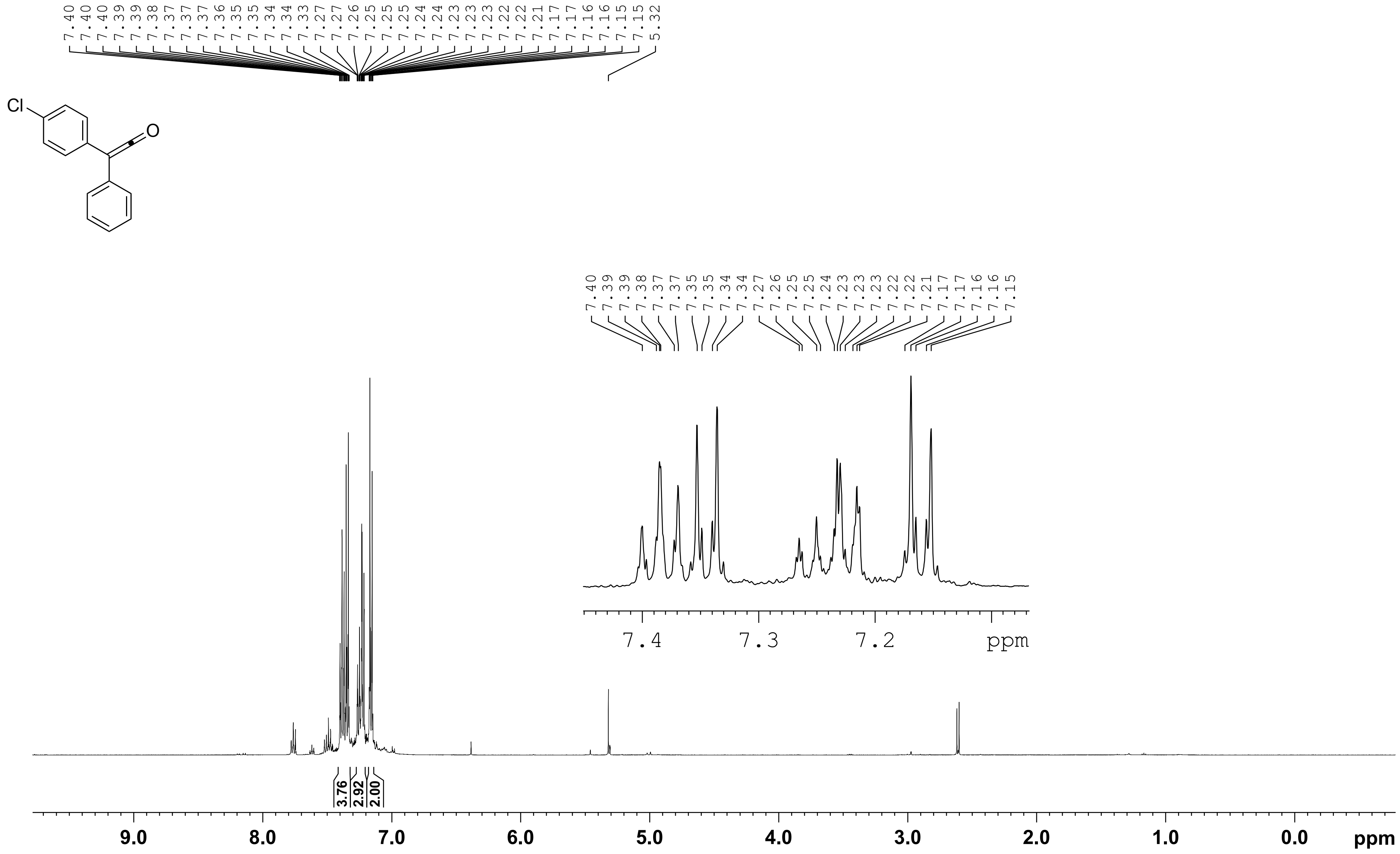

Figure S8. $^{13}C\{^{1}H\}$ NMR spectrum (126 MHz, CD_2Cl_2, 298 K) of **1e**.

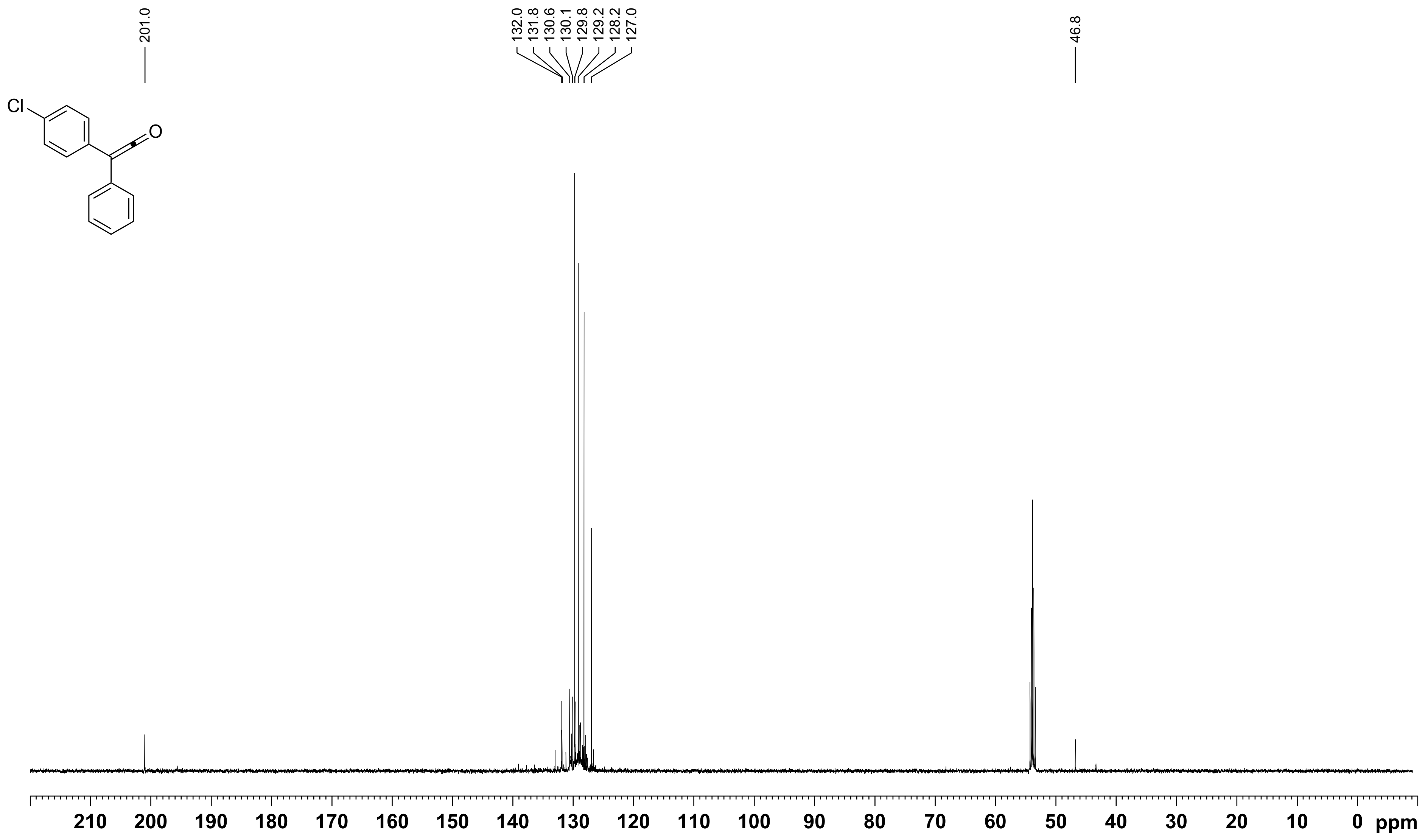

Figure S9. ^{1}H NMR spectrum (400 MHz, $CDCl_3$, 298 K) of **1r**.

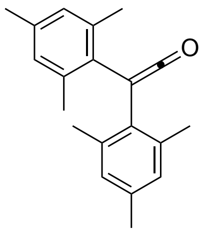

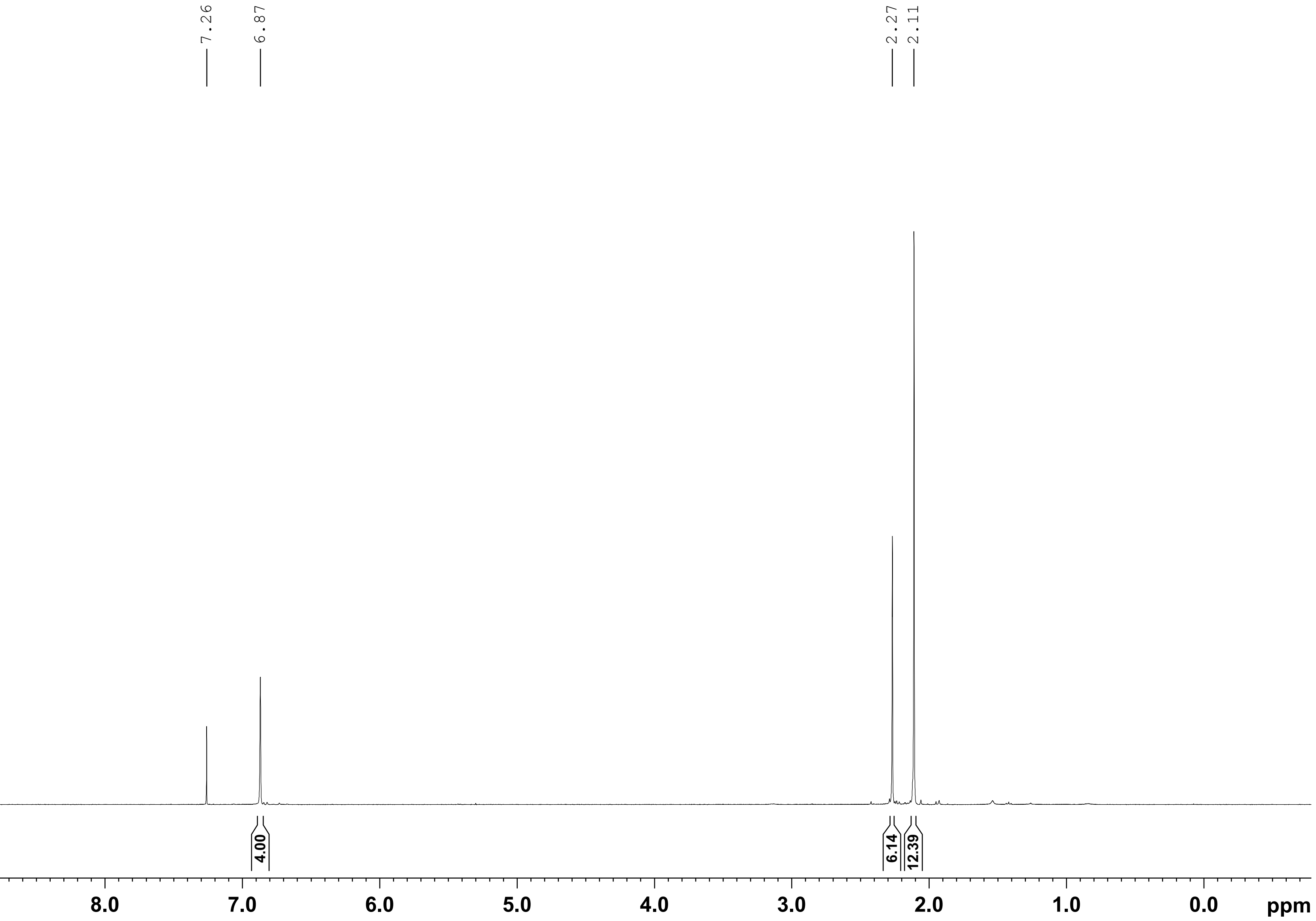

Figure S10. $^{13}C\{^1H\}$ NMR spectrum (101 MHz, $CDCl_3$, 298 K) of **1r**.

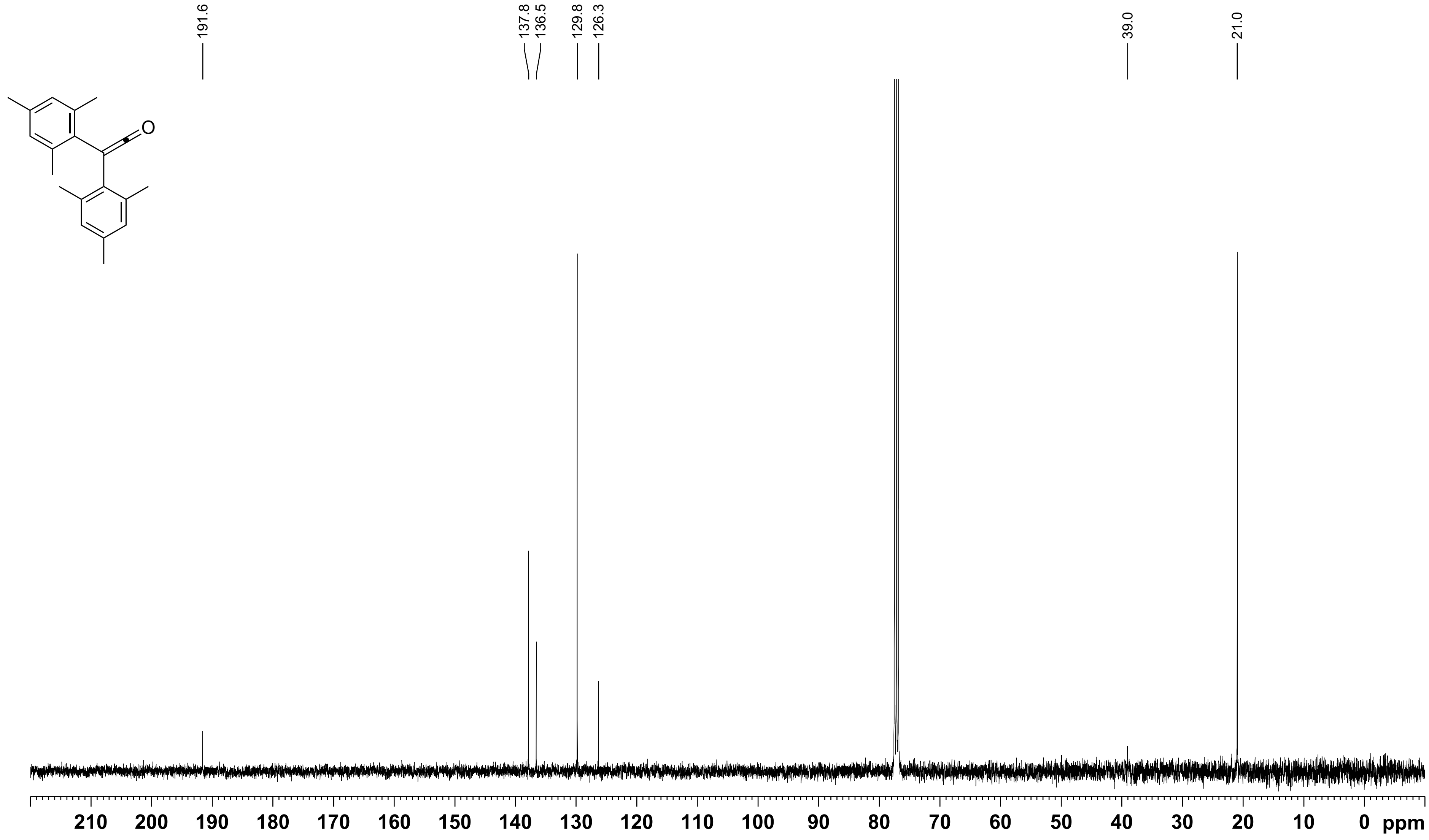

Figure S11. ^{1}H NMR spectrum (400 MHz, CD_2Cl_2, 298 K) of **1h**. (* = unreacted acid chloride)

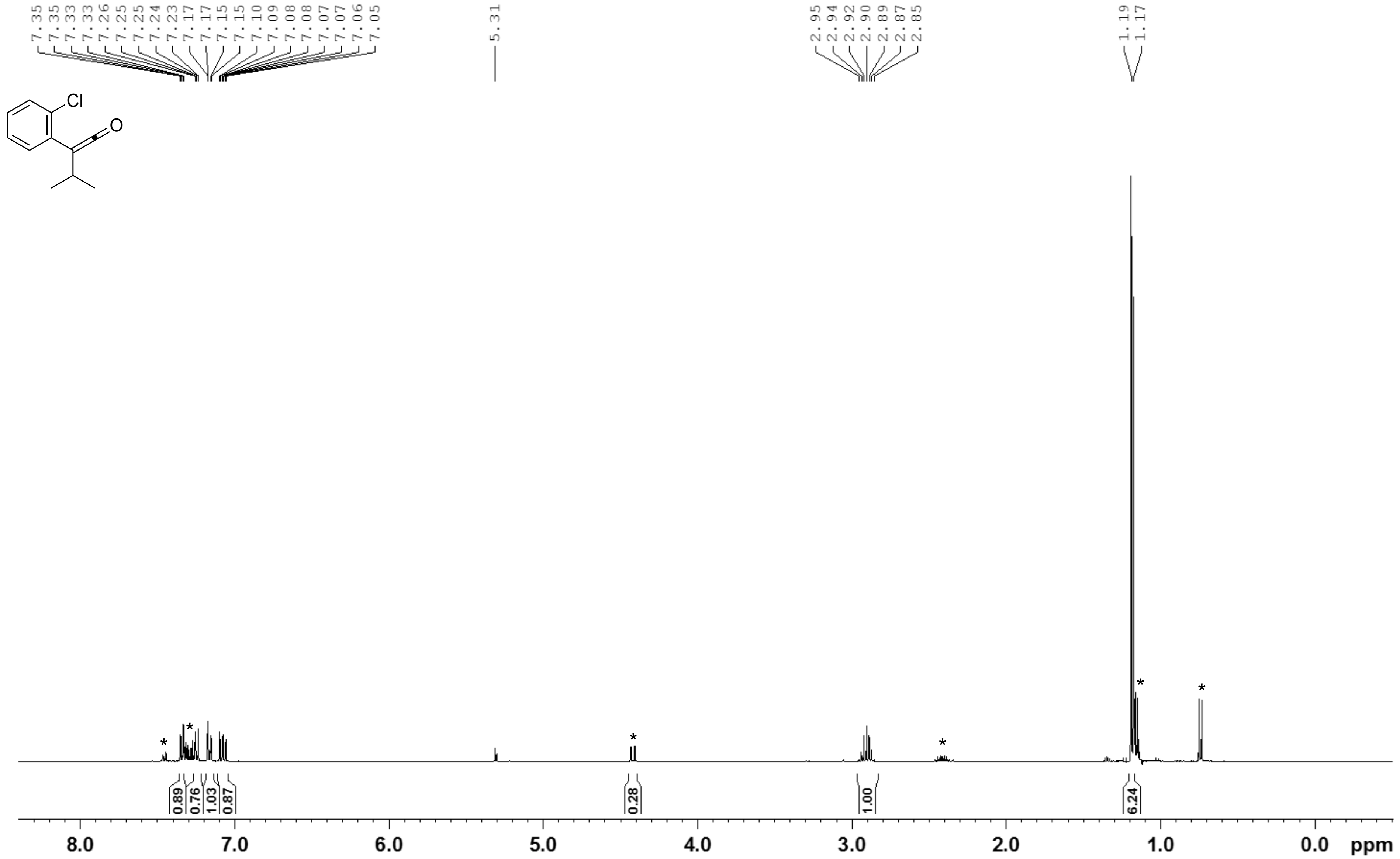

Figure S12. $^{13}C\{^{1}H\}$ NMR spectrum (101 MHz, CD_2Cl_2, 298 K) of **1h**. (* = unreacted acid chloride)

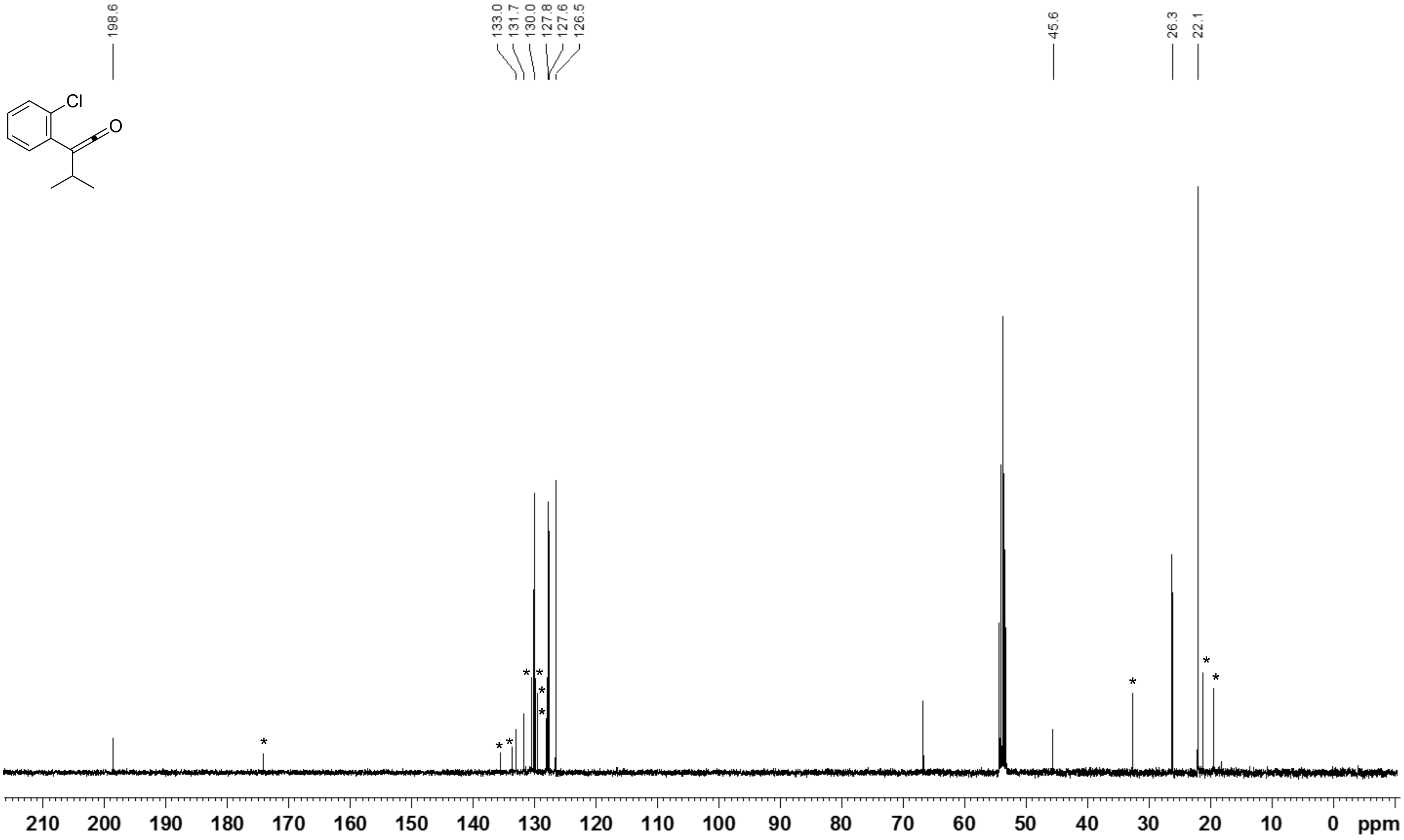

Figure S13. ^{1}H NMR spectrum (500 MHz, CD_2Cl_2, 298 K) of **1j**.

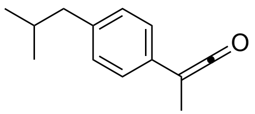

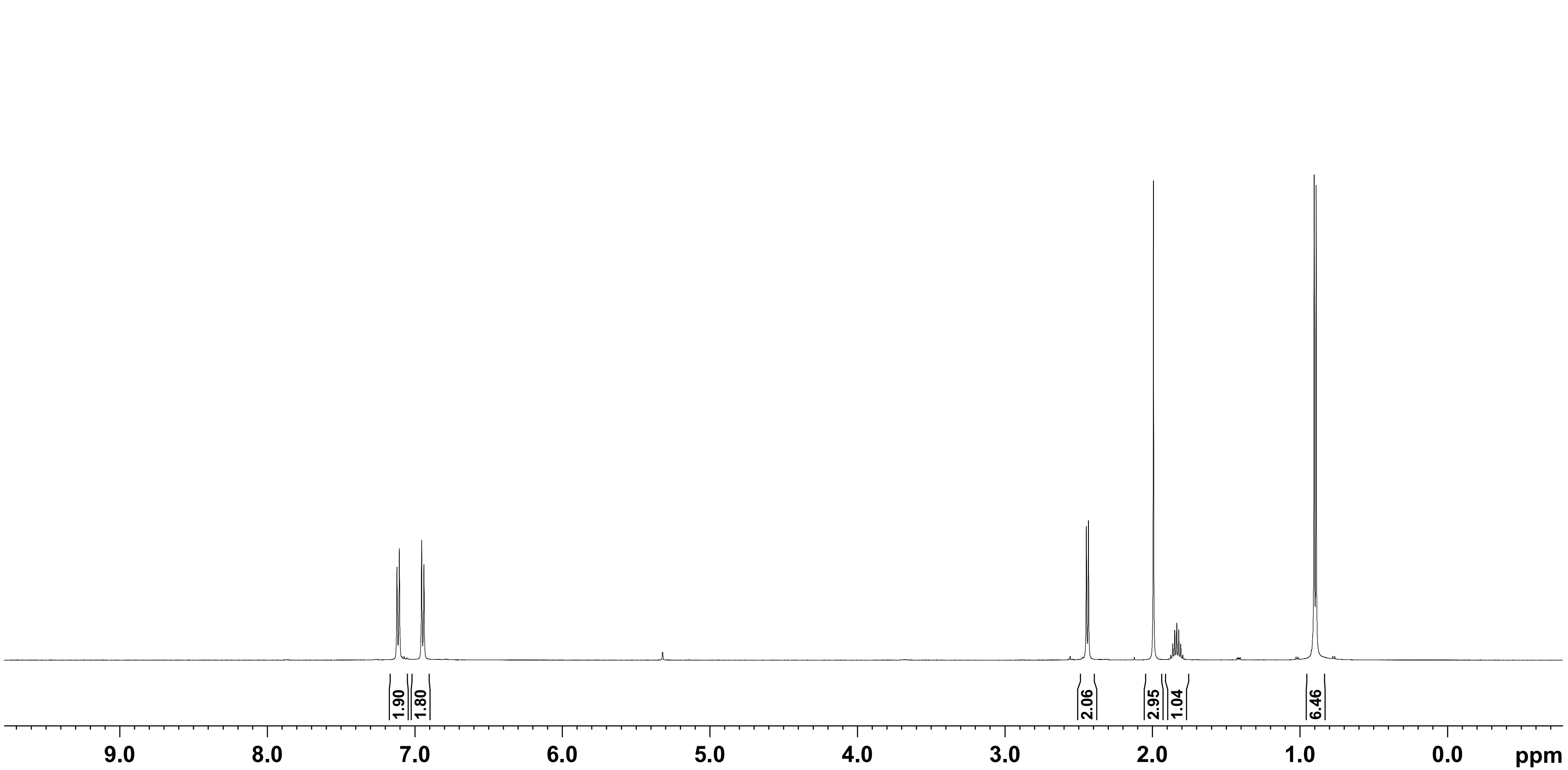

Figure S14. $^{13}C\{^{1}H\}$ NMR spectrum (126 MHz, CD_2Cl_2, 298 K) of **1j**.

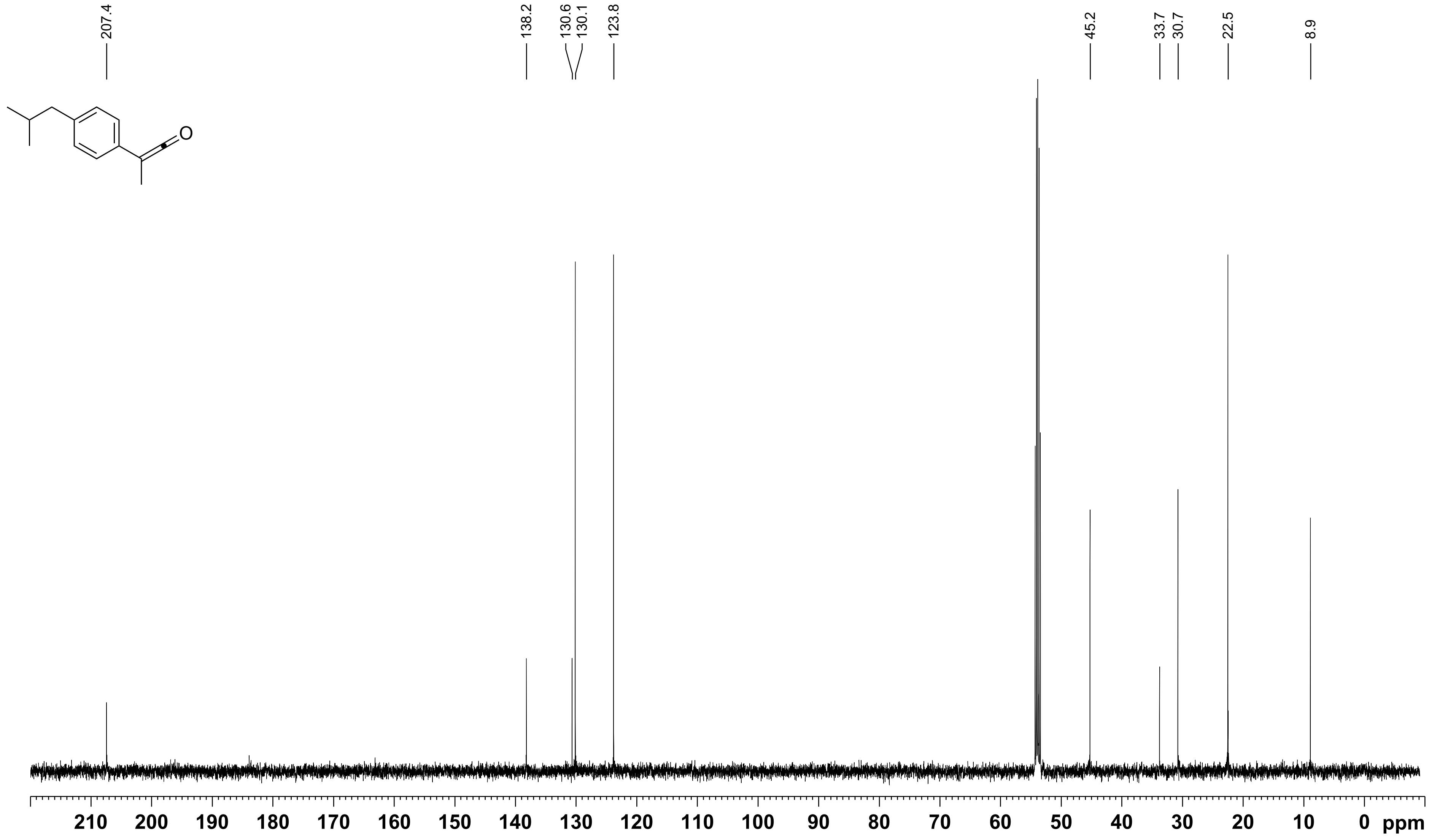

Figure S15. ^{1}H NMR spectrum (500 MHz, CD_2Cl_2, 298 K) of **1m**.

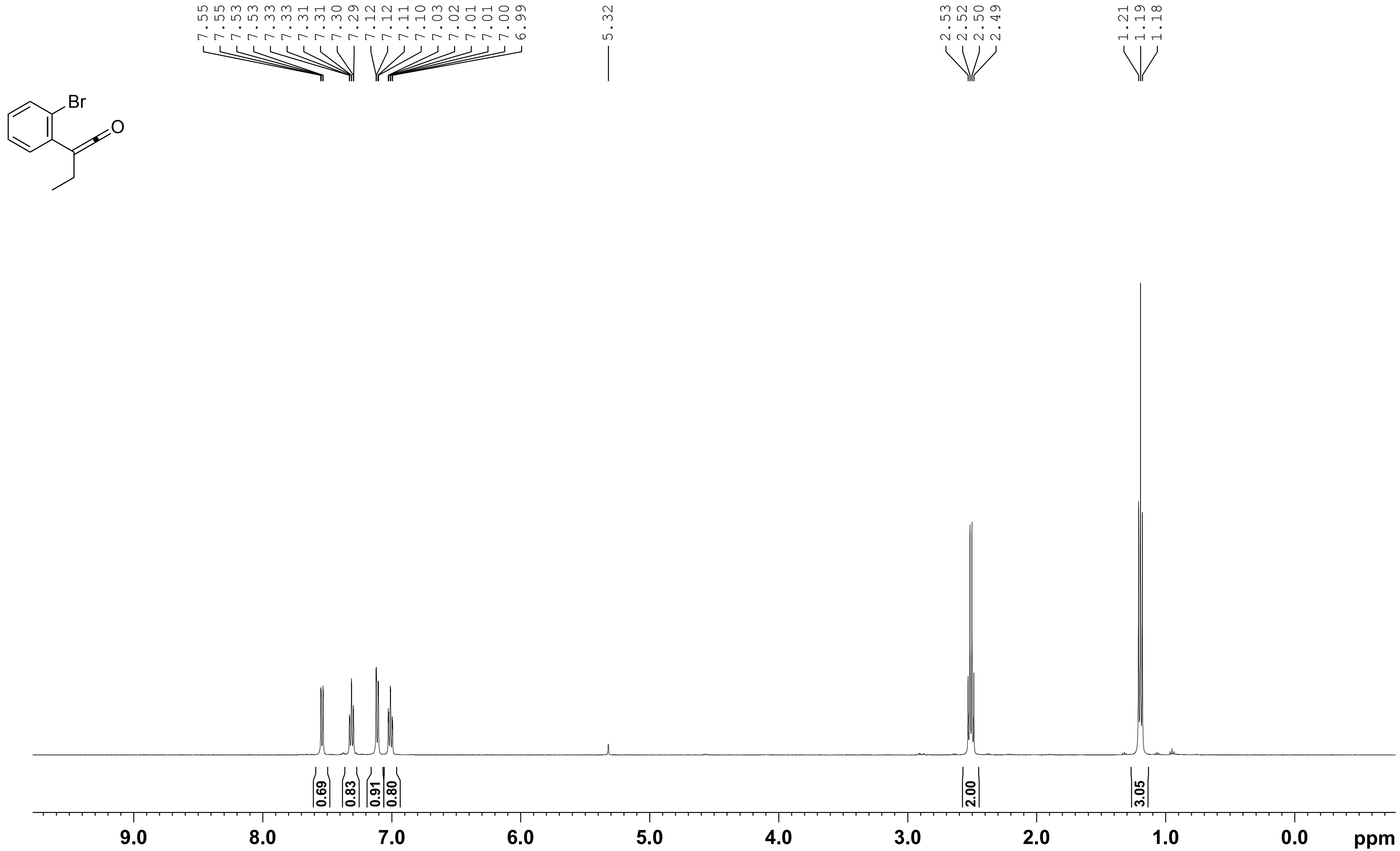

Figure S16. $^{13}C\{^{1}H\}$ NMR spectrum (126 MHz, CD_2Cl_2, 298 K) of **1m**.

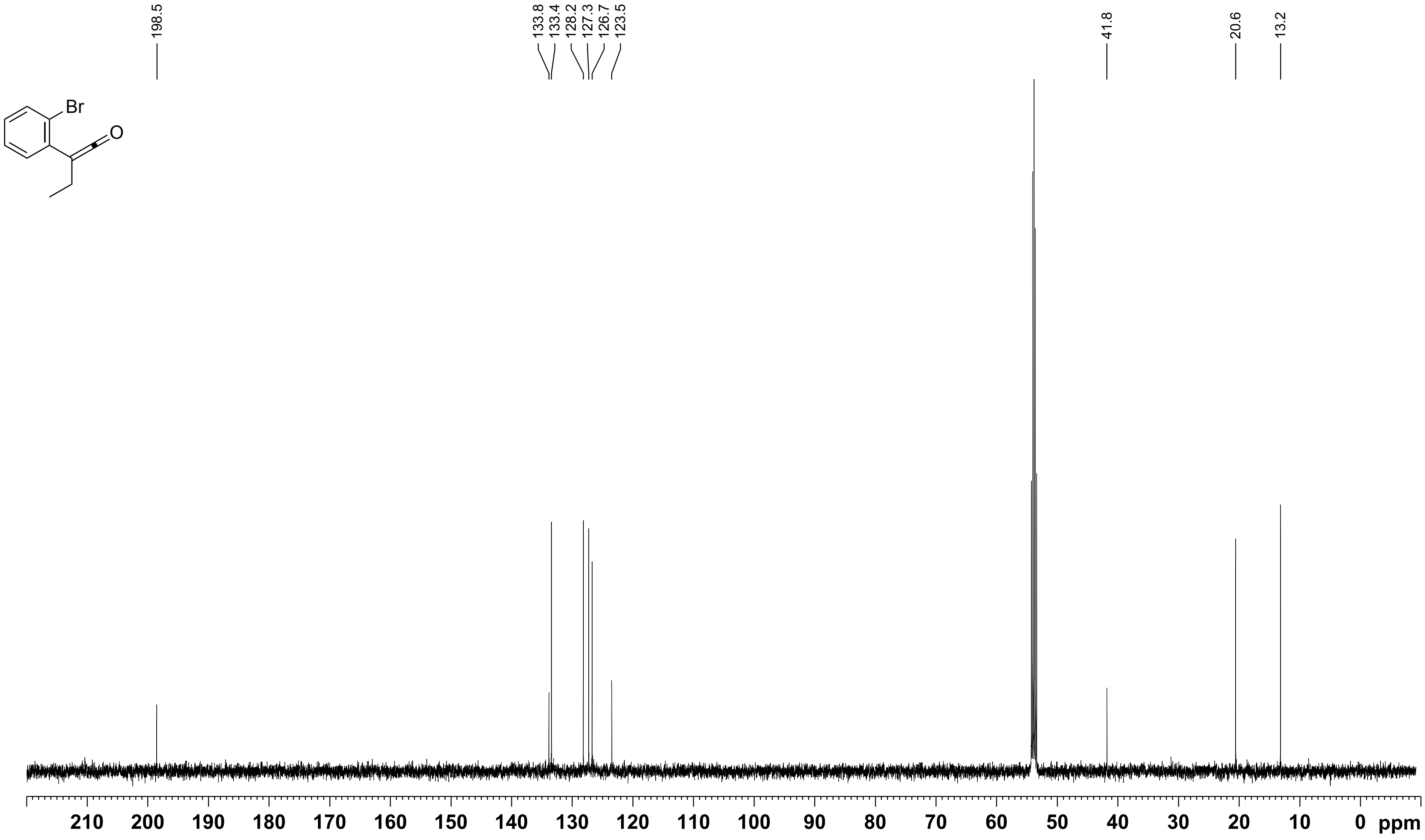

Figure S17. ^{1}H NMR spectrum (500 MHz, $CDCl_3$, 298 K) of **3aa**.

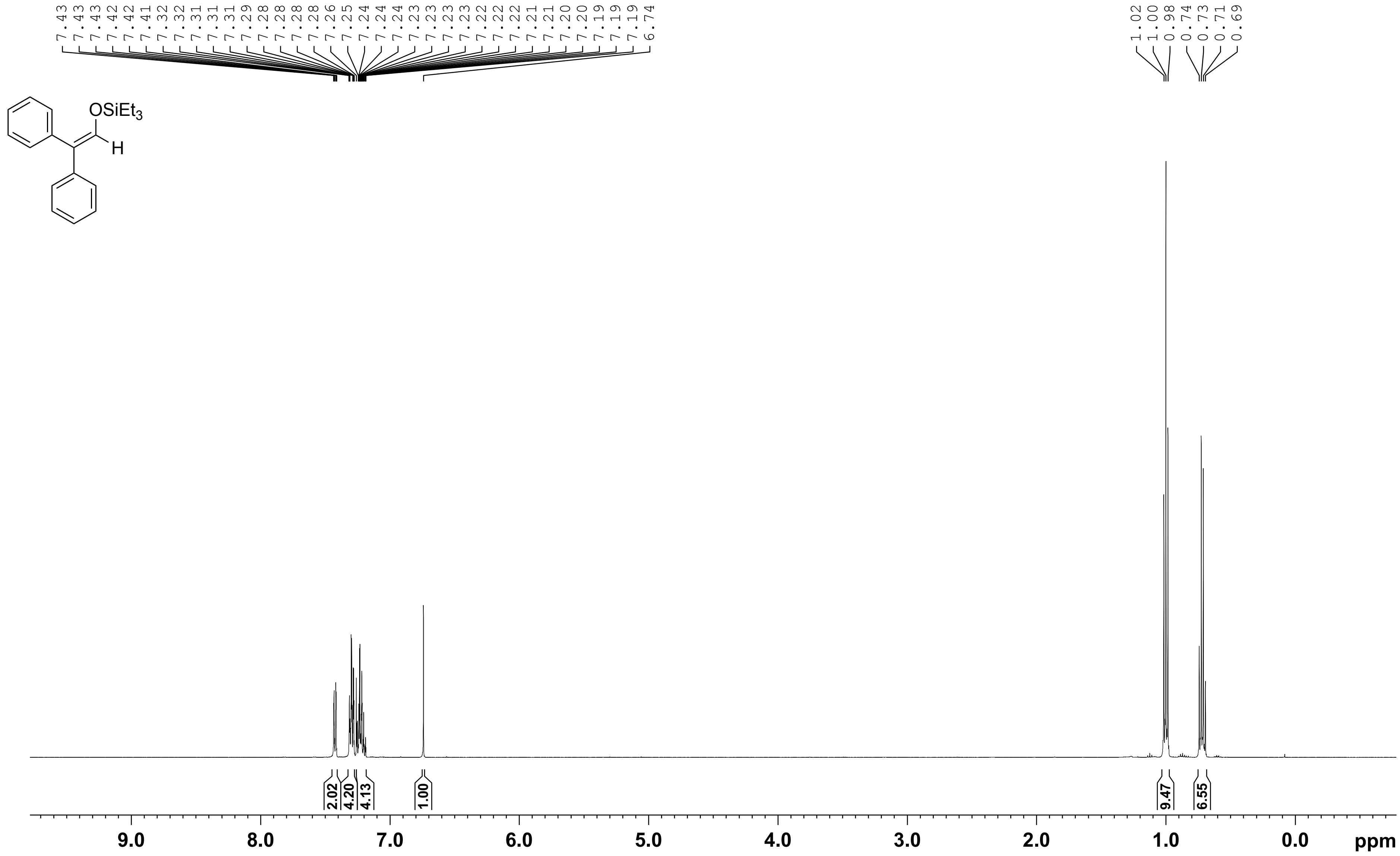

Figure S18. $^{13}C\{^{1}H\}$ NMR spectrum (126 MHz, $CDCl_3$, 298 K) of **3aa**.

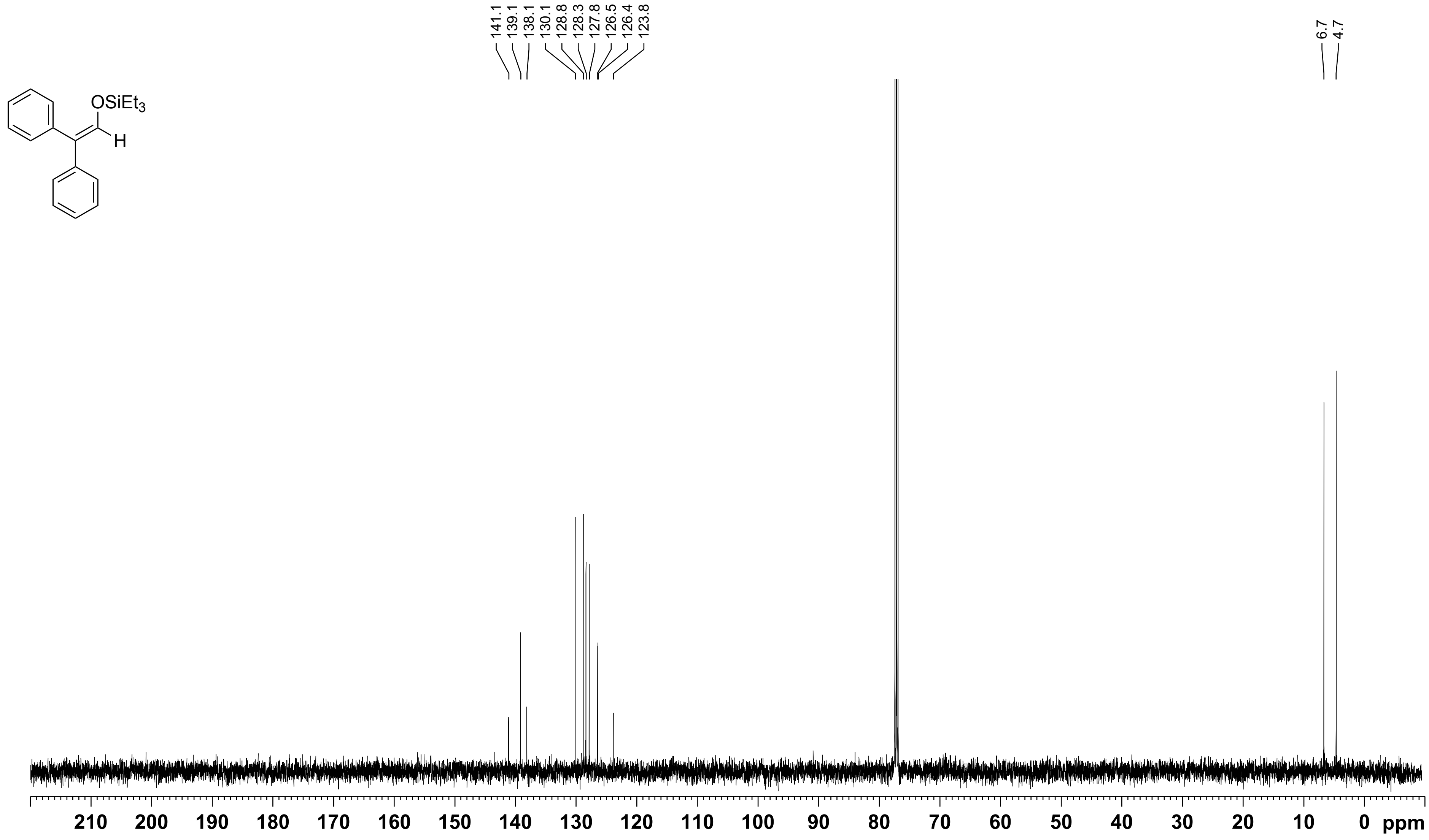

Figure S19. ^{29}Si DEPT NMR spectrum (99 MHz, $CDCl_3$, 298 K, optimized for J = 7 Hz) of **3aa**.

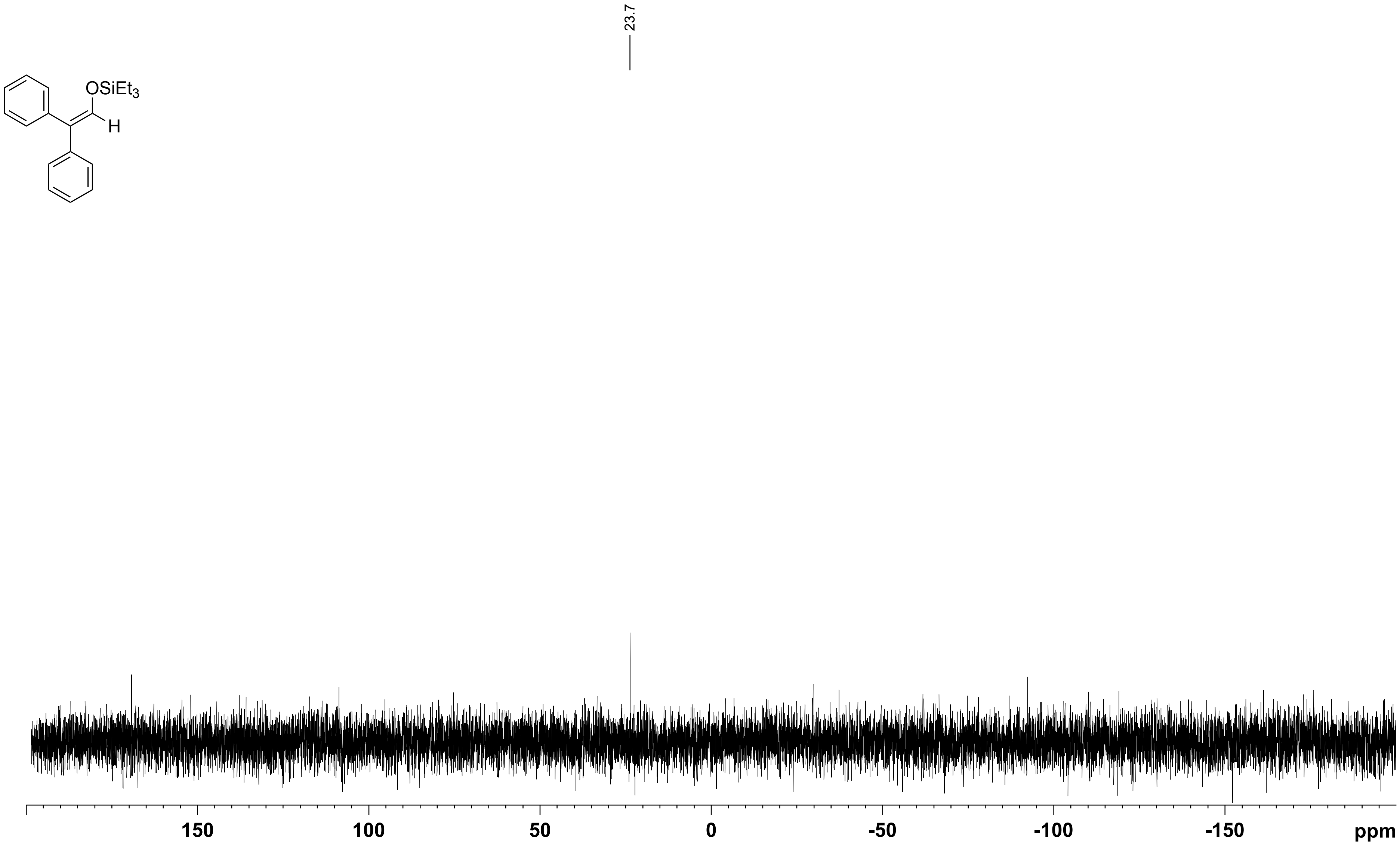

Figure S20. ^{1}H NMR spectrum (500 MHz, $CDCl_3$, 298 K) of **3ab**.

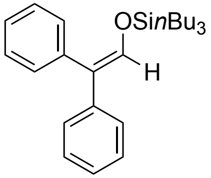

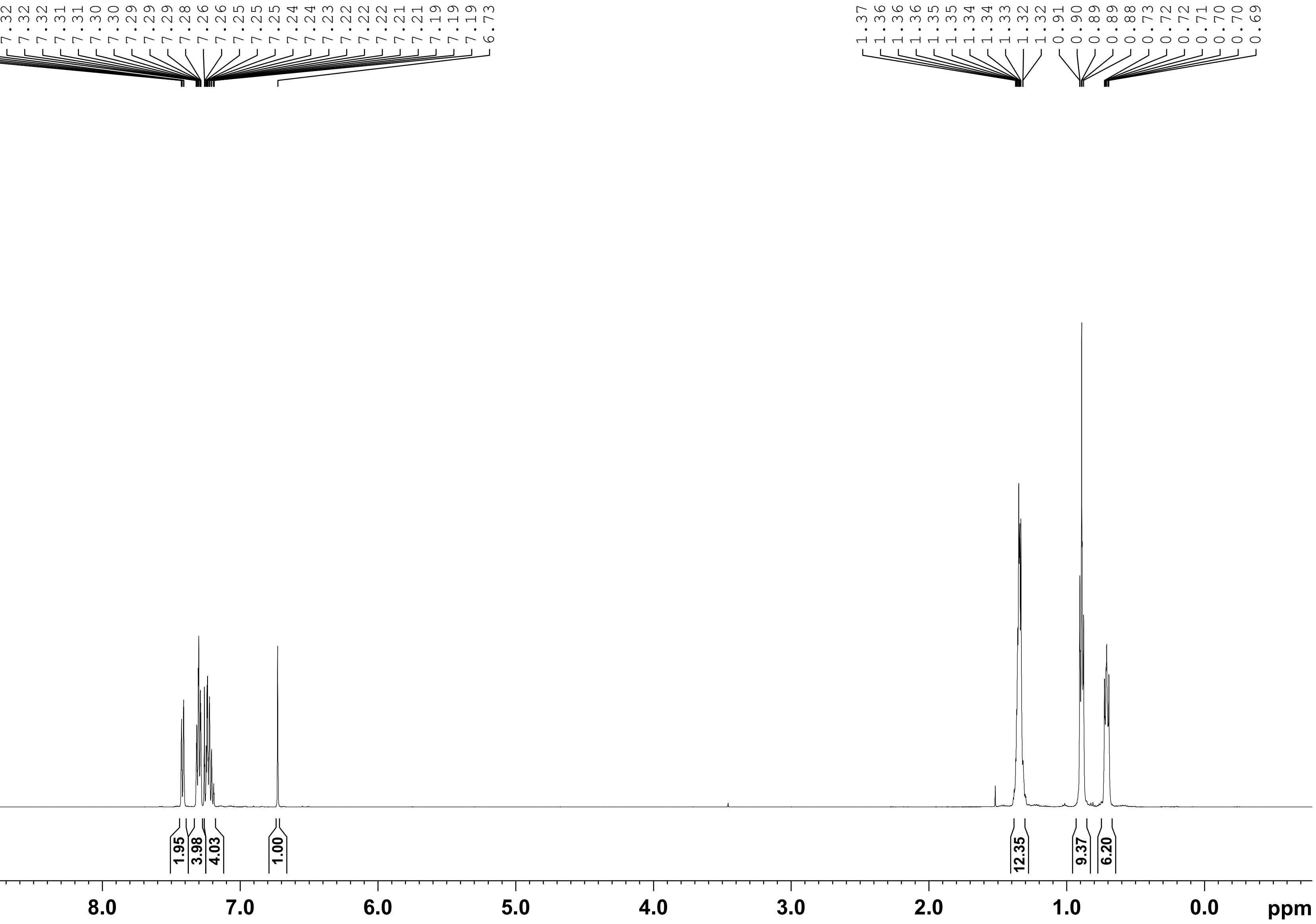

Figure S21. $^{13}C\{^{1}H\}$ NMR spectrum (126 MHz, $CDCl_3$, 298 K) of **3ab**.

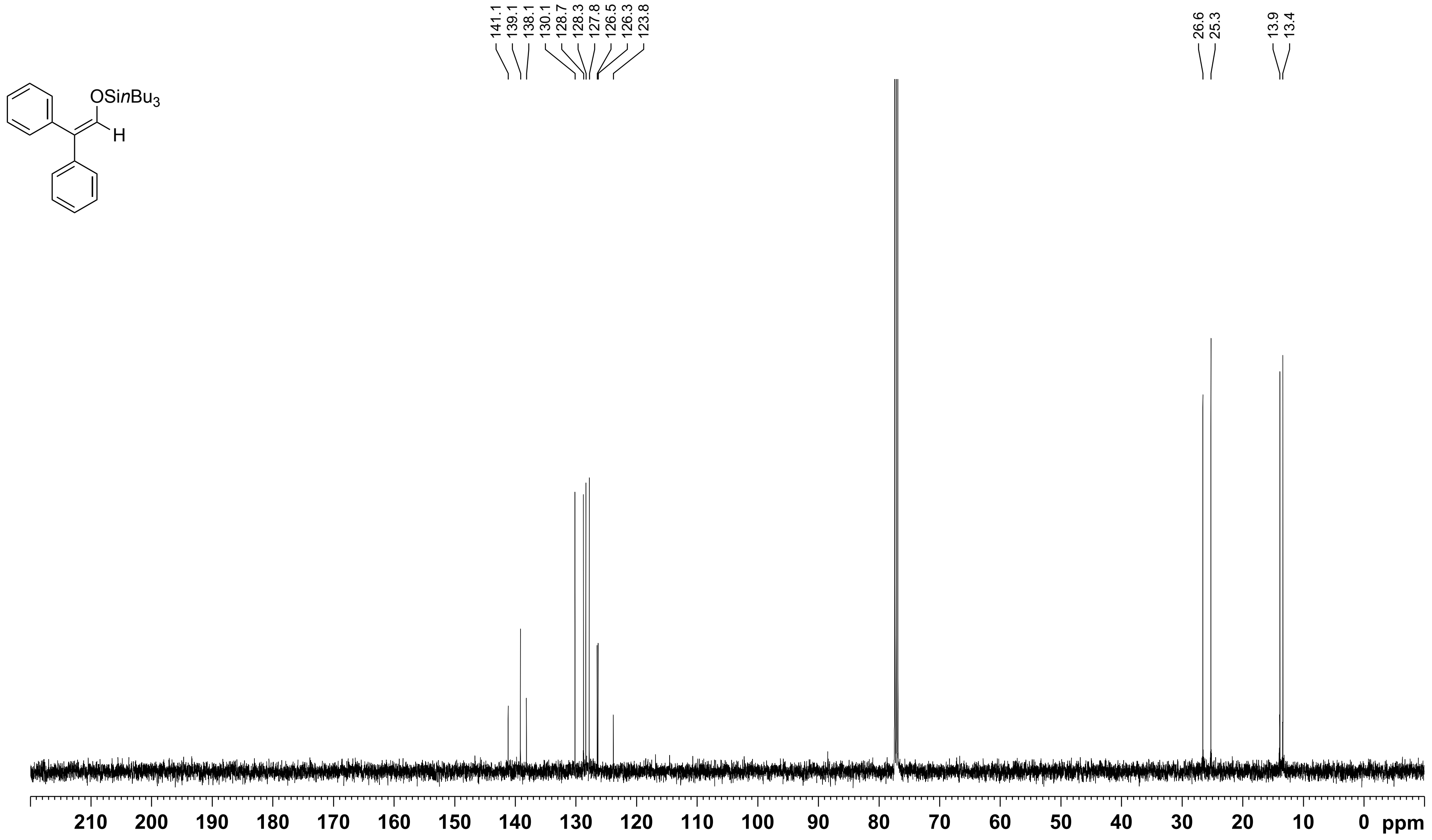

Figure S22. ^{29}Si DEPT NMR spectrum (99 MHz, $CDCl_3$, 298 K, optimized for J = 7 Hz) of **3ab**.

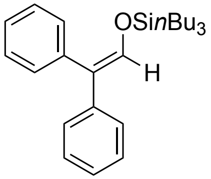

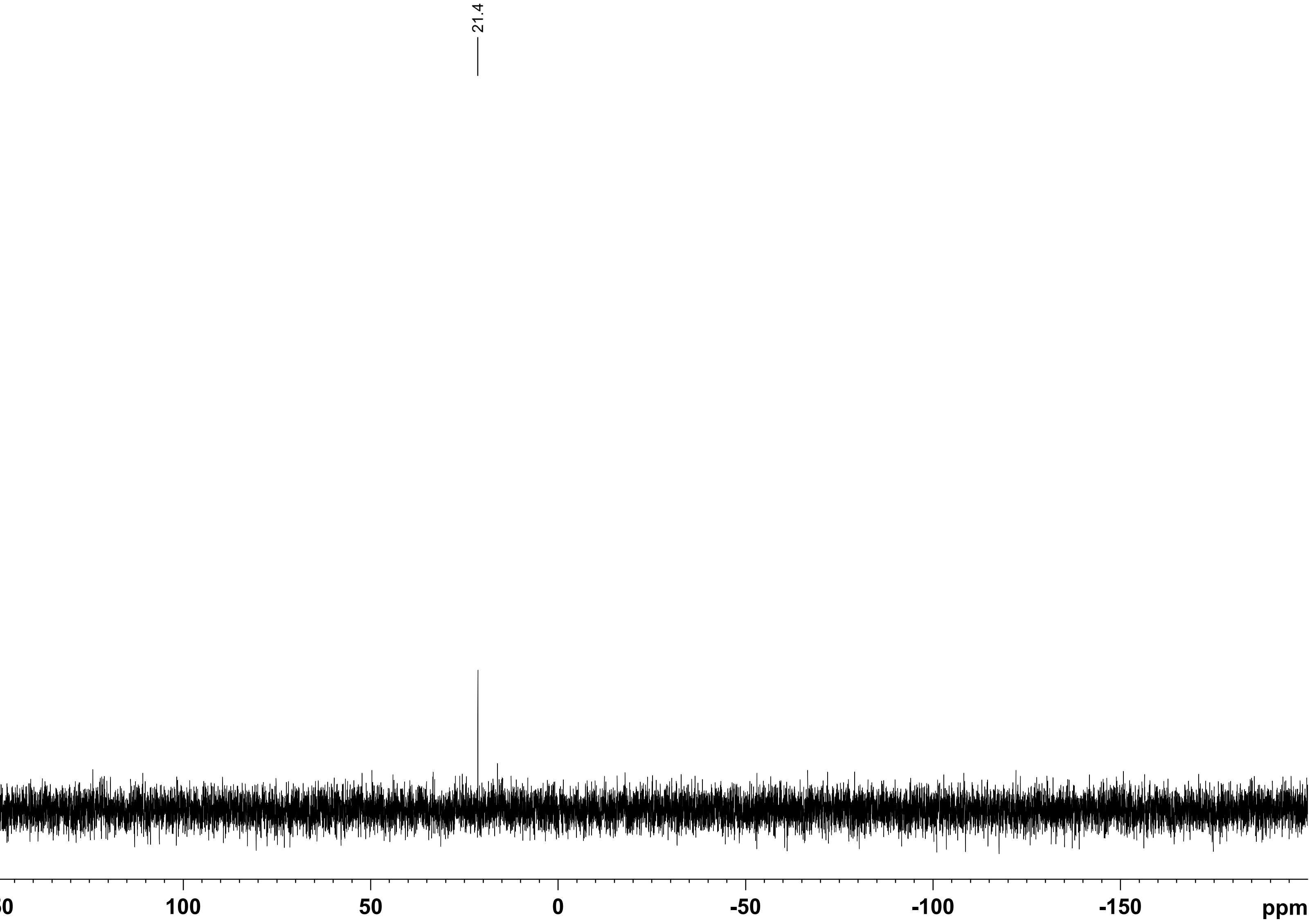

Figure S23. ^{1}H NMR spectrum (500 MHz, $CDCl_3$, 298 K) of **3ad**.

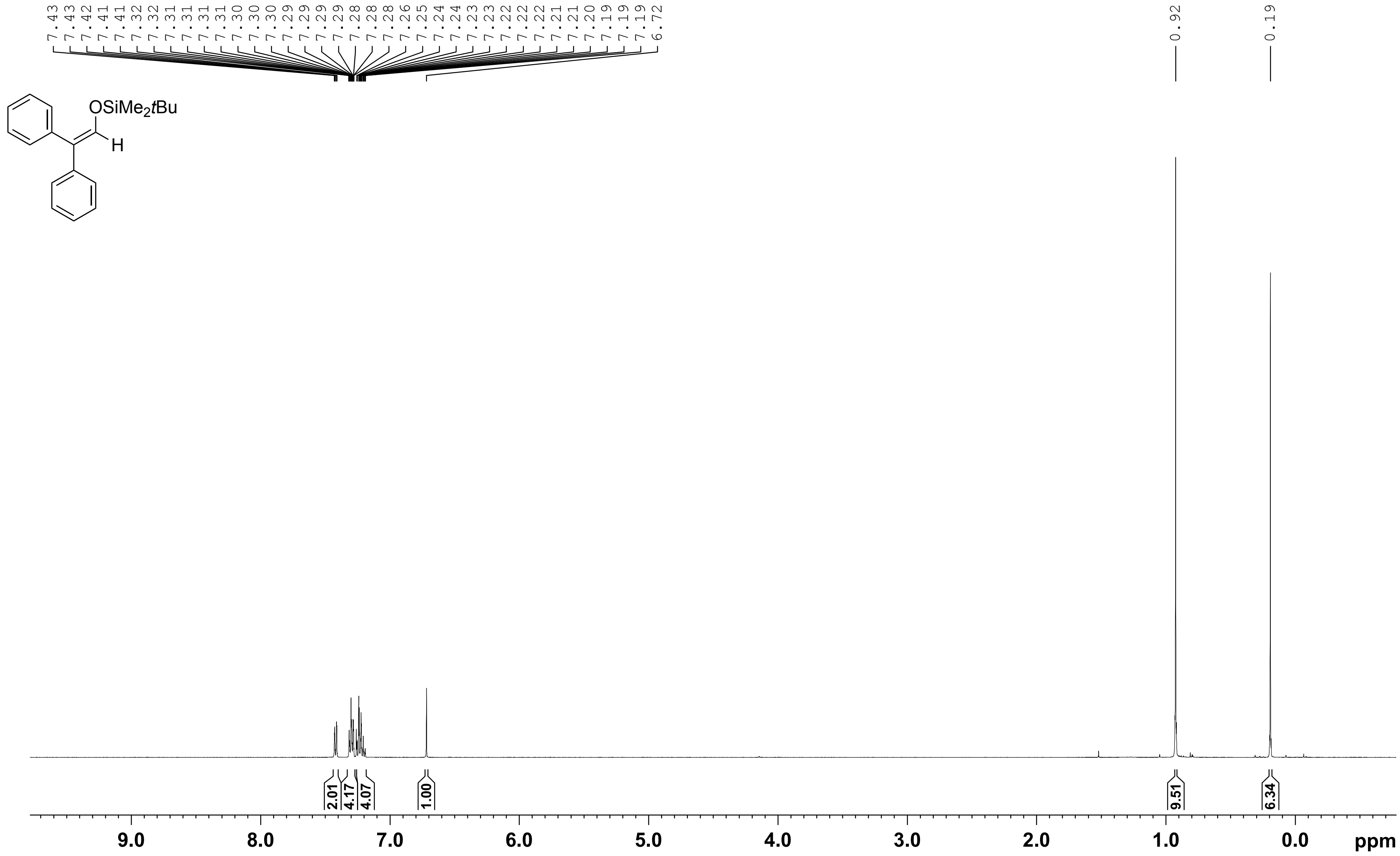

Figure S24. $^{13}C\{^{1}H\}$ NMR spectrum (126 MHz, $CDCl_3$, 298 K) of **3ad**.

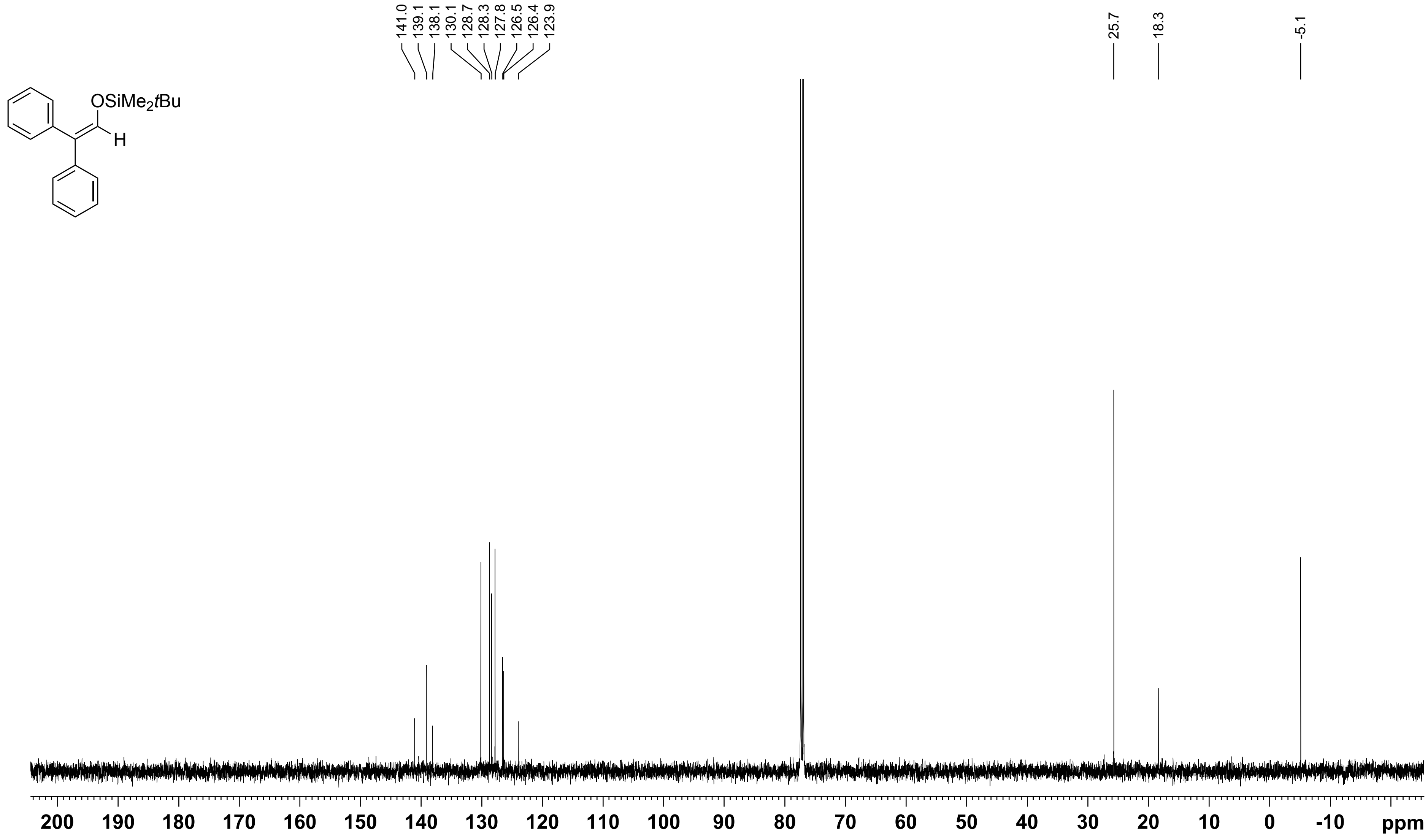

Figure S25. ^{29}Si DEPT NMR spectrum (99 MHz, $CDCl_3$, 298 K, optimized for J = 7 Hz) of **3ad**.

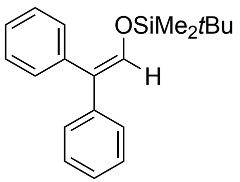

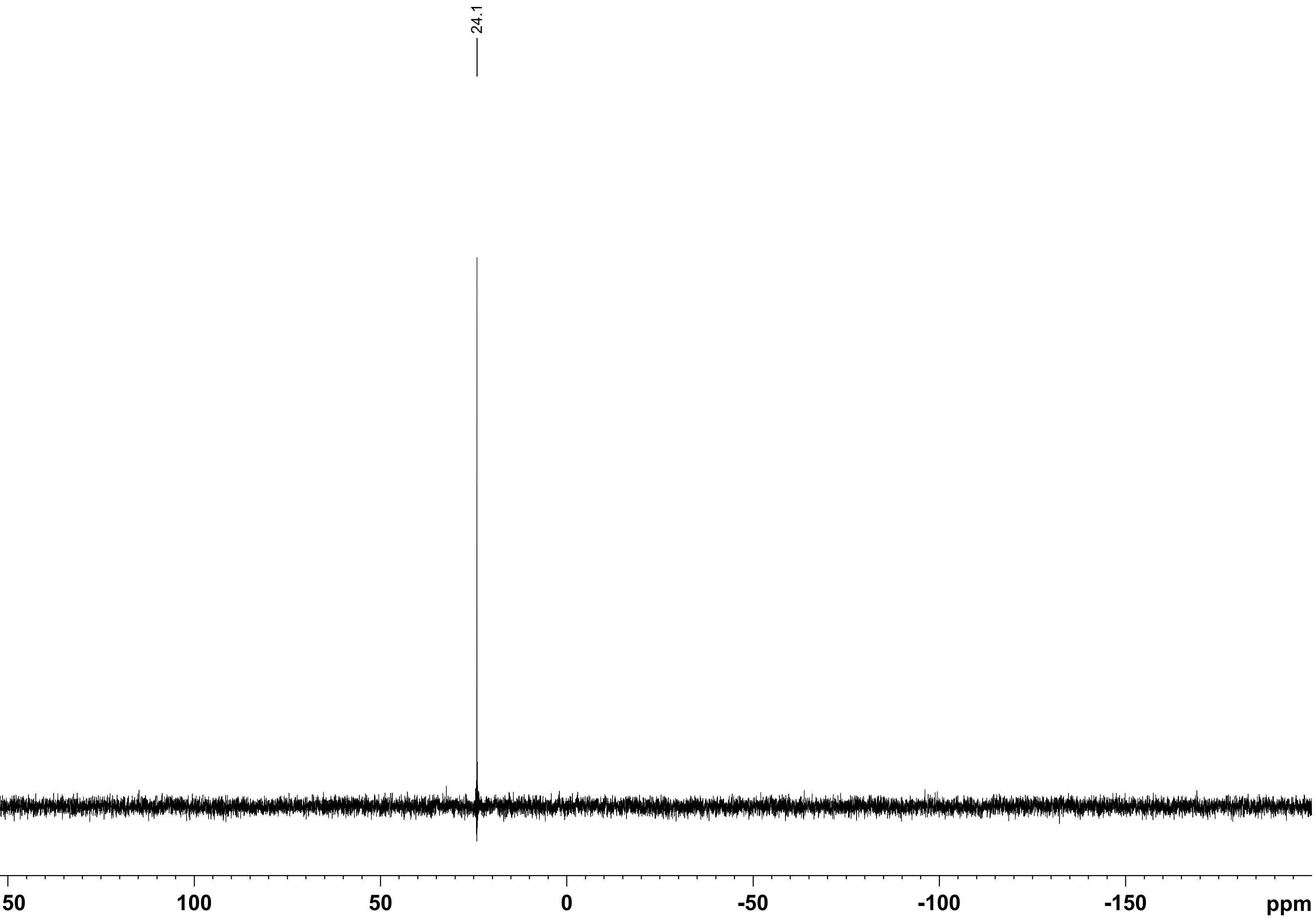

Figure S26. ^{1}H NMR spectrum (500 MHz, $CDCl_3$, 298 K) of **3ae** from crude mixture. (* = excess Me_2PhSiH)

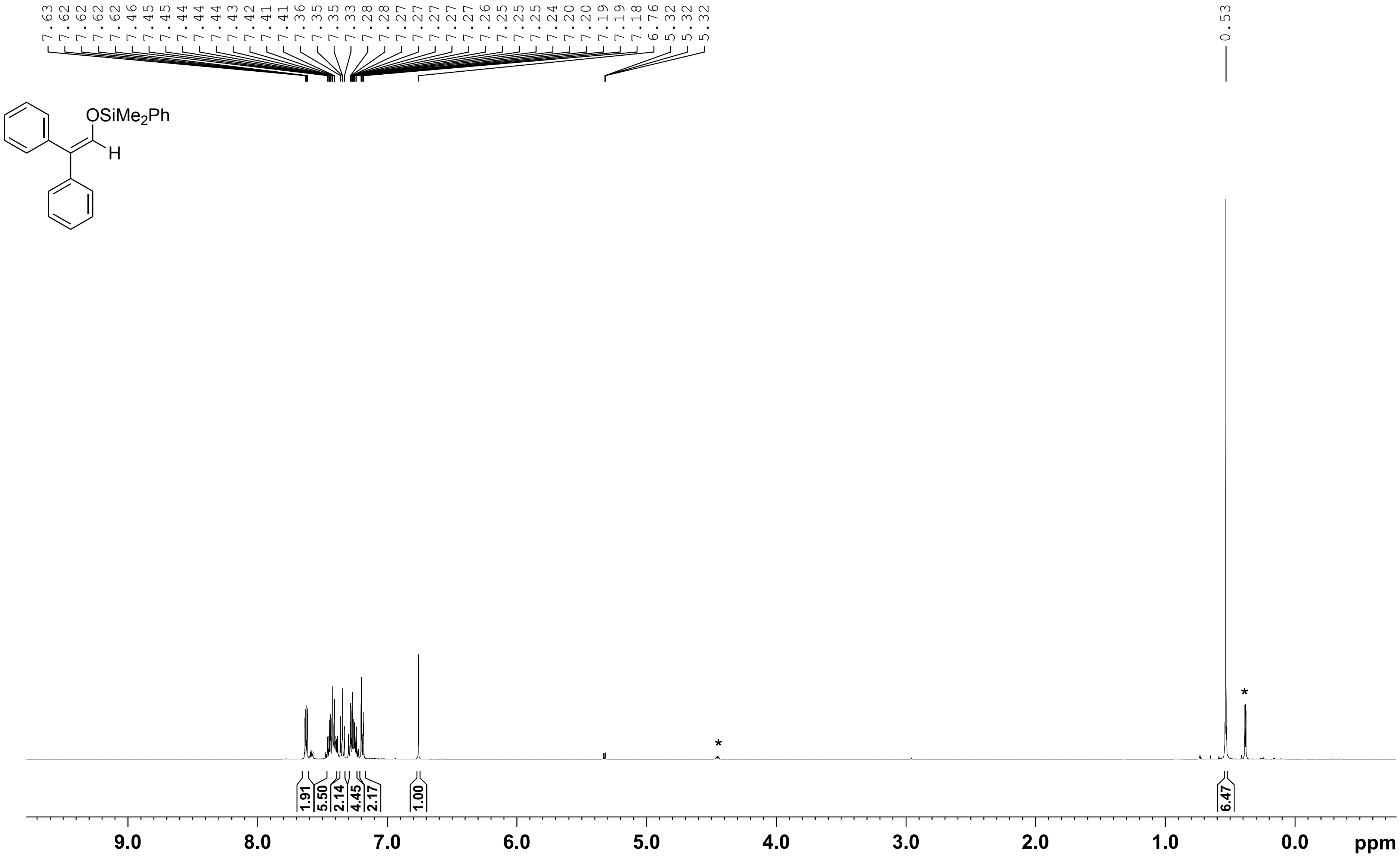

Figure S27. ^{29}Si DEPT NMR spectrum (99 MHz, $CDCl_3$, 298 K, optimized for J = 7 Hz) of **3ae** from crude mixture. (* = excess Me_2PhSiH)

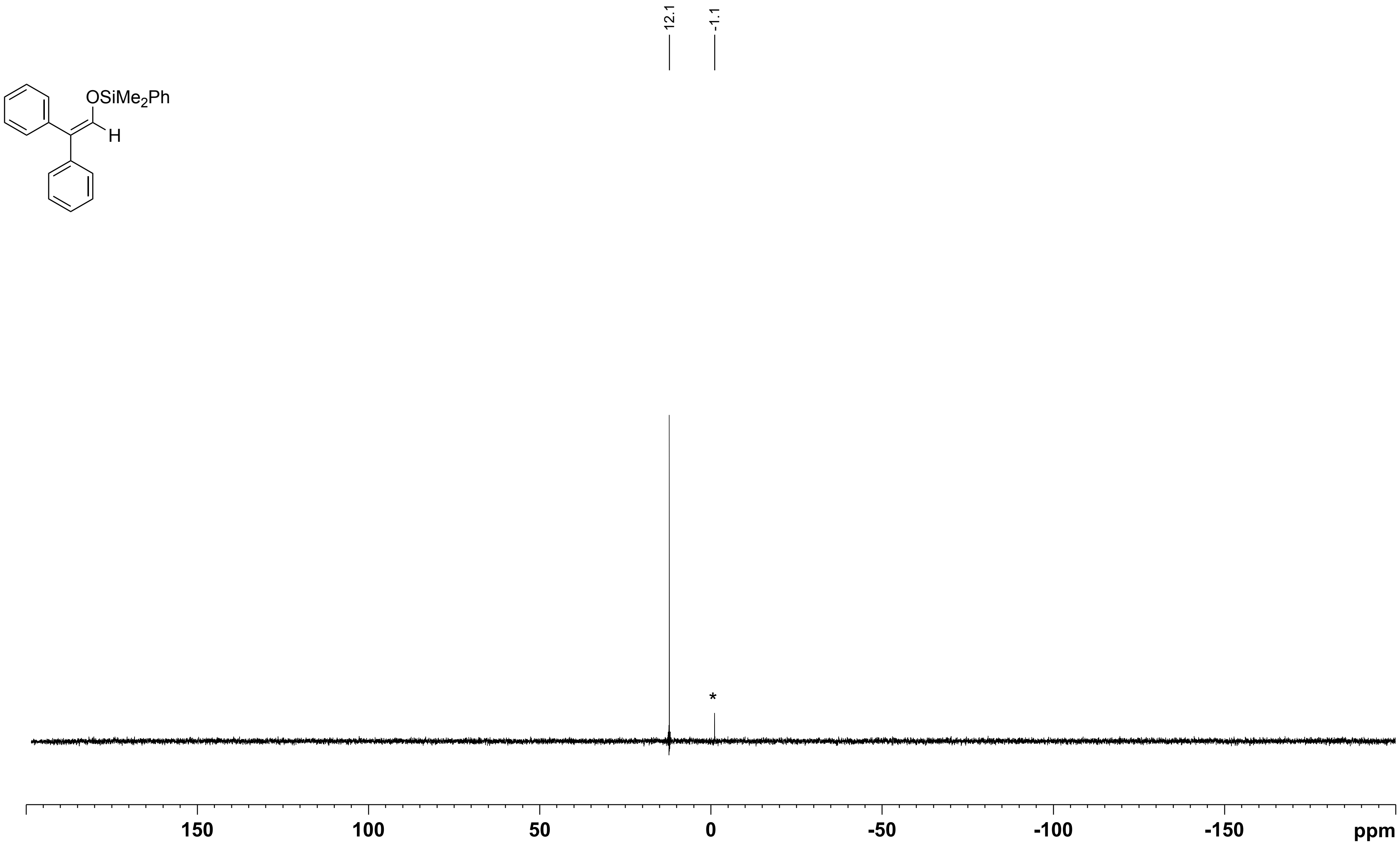

Figure S28. ^{1}H NMR spectrum (500 MHz, $CDCl_3$, 298 K) of **3aa**-d_1. (# = H_2O)

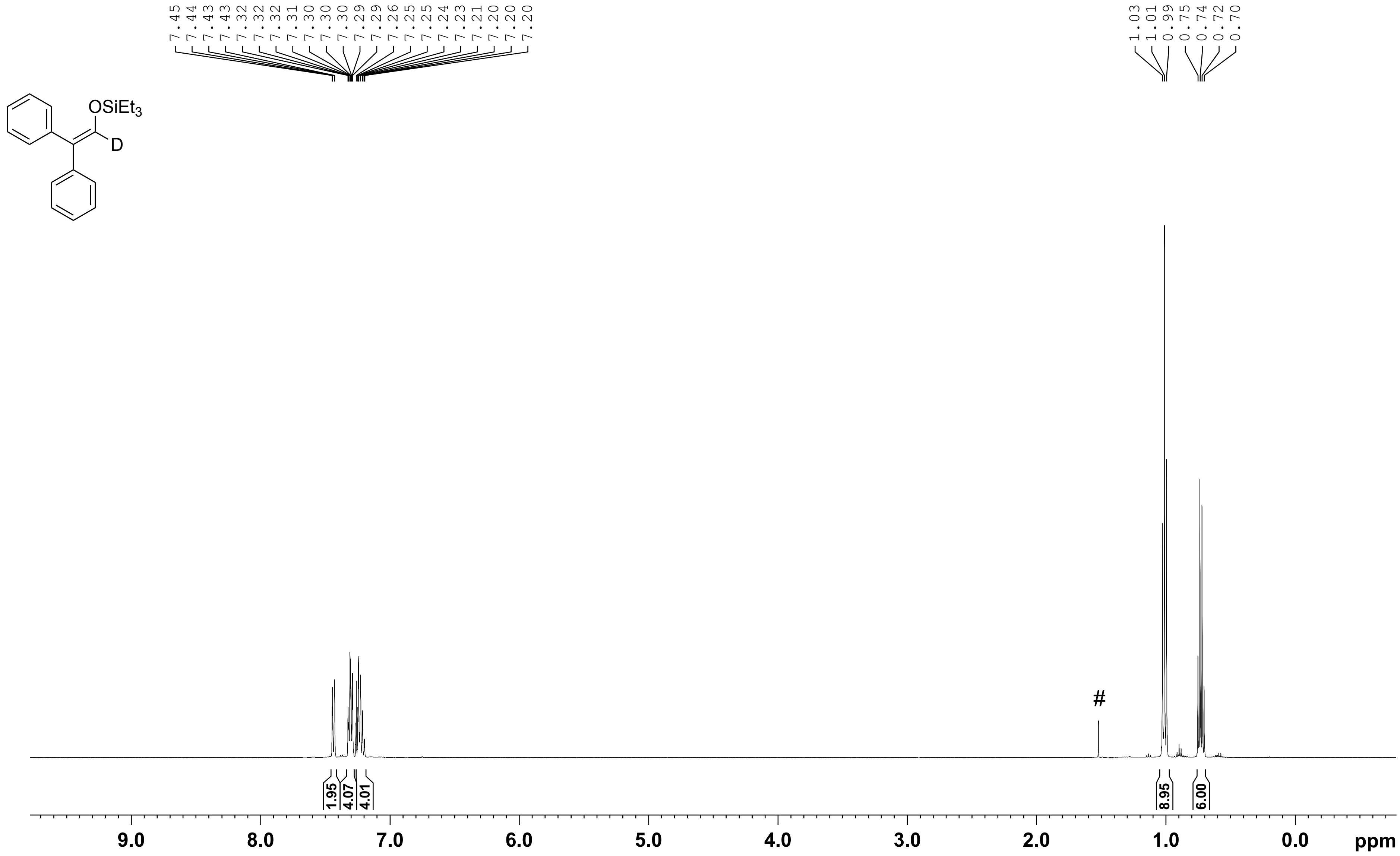

Figure S29. $^{13}C\{^{1}H\}$ NMR spectrum (126 MHz, $CDCl_3$, 298 K) of **3aa**-d_1.

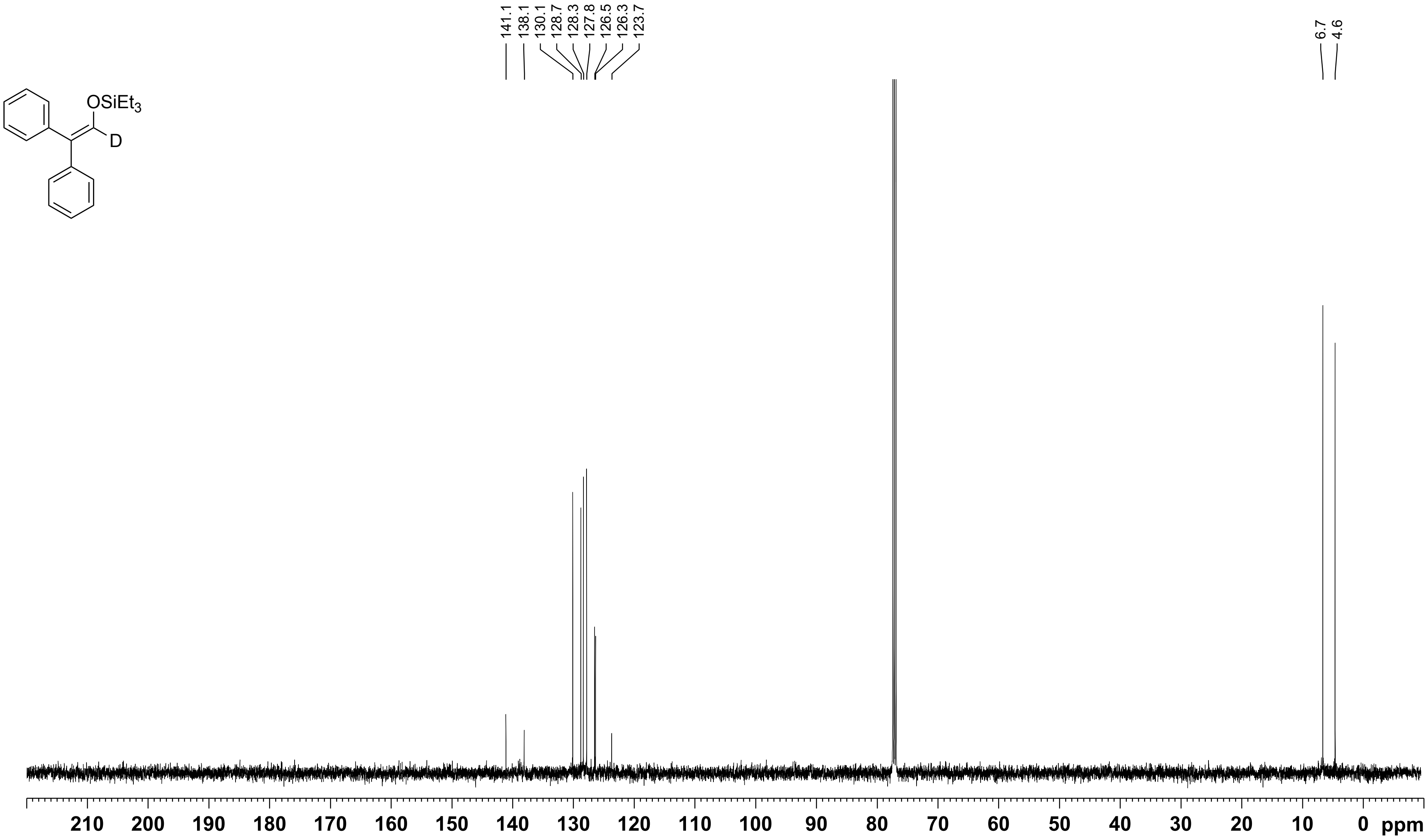

Figure S30. ^{29}Si DEPT NMR spectrum (99 MHz, $CDCl_3$, 298 K, optimized for J = 7 Hz) of **3aa**-d_1.

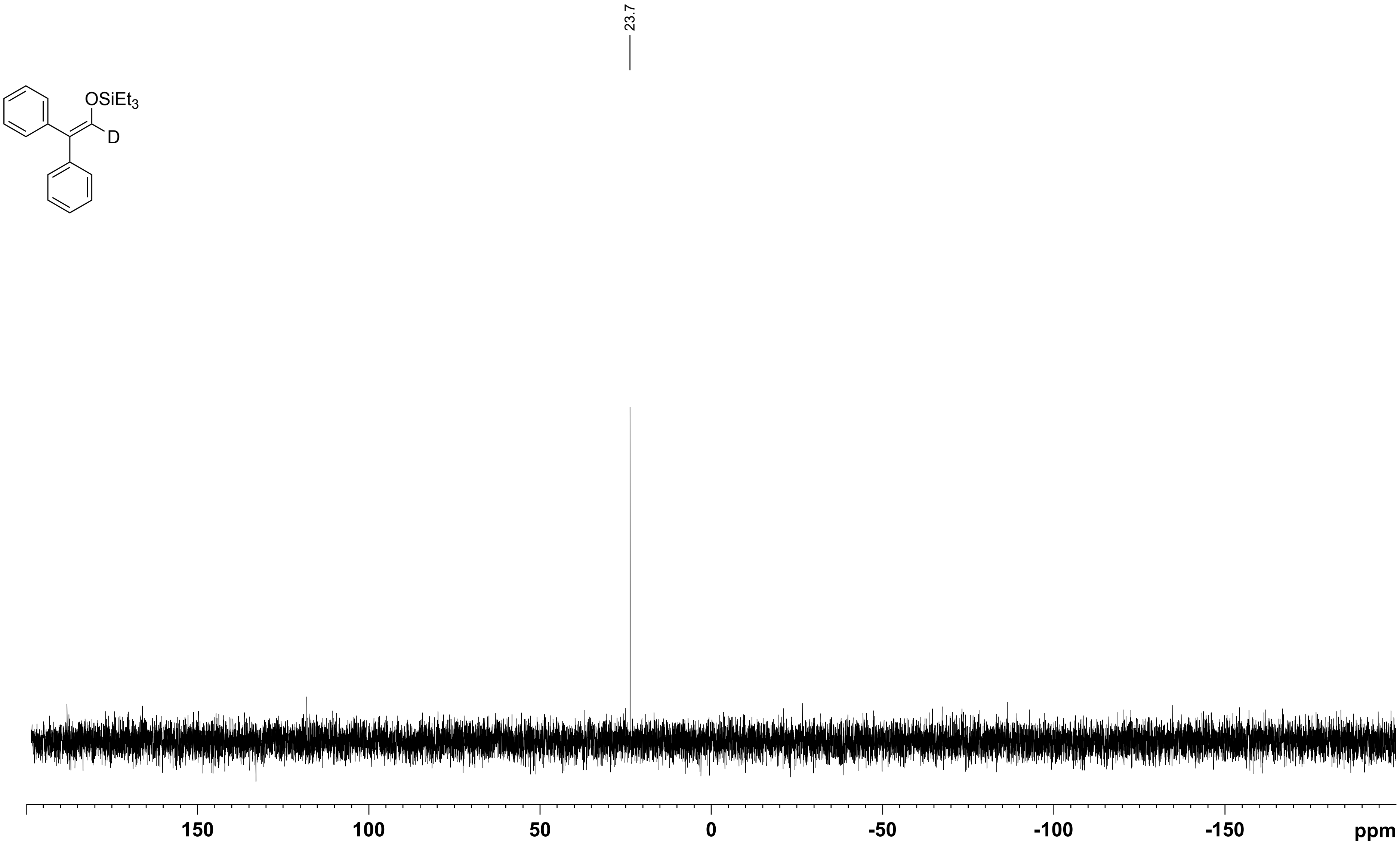

Figure S31. ^2H NMR spectrum (500 MHz, $CDCl_3$, 298 K) of **3aa**-d_1.

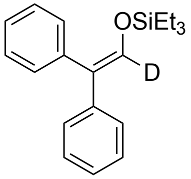

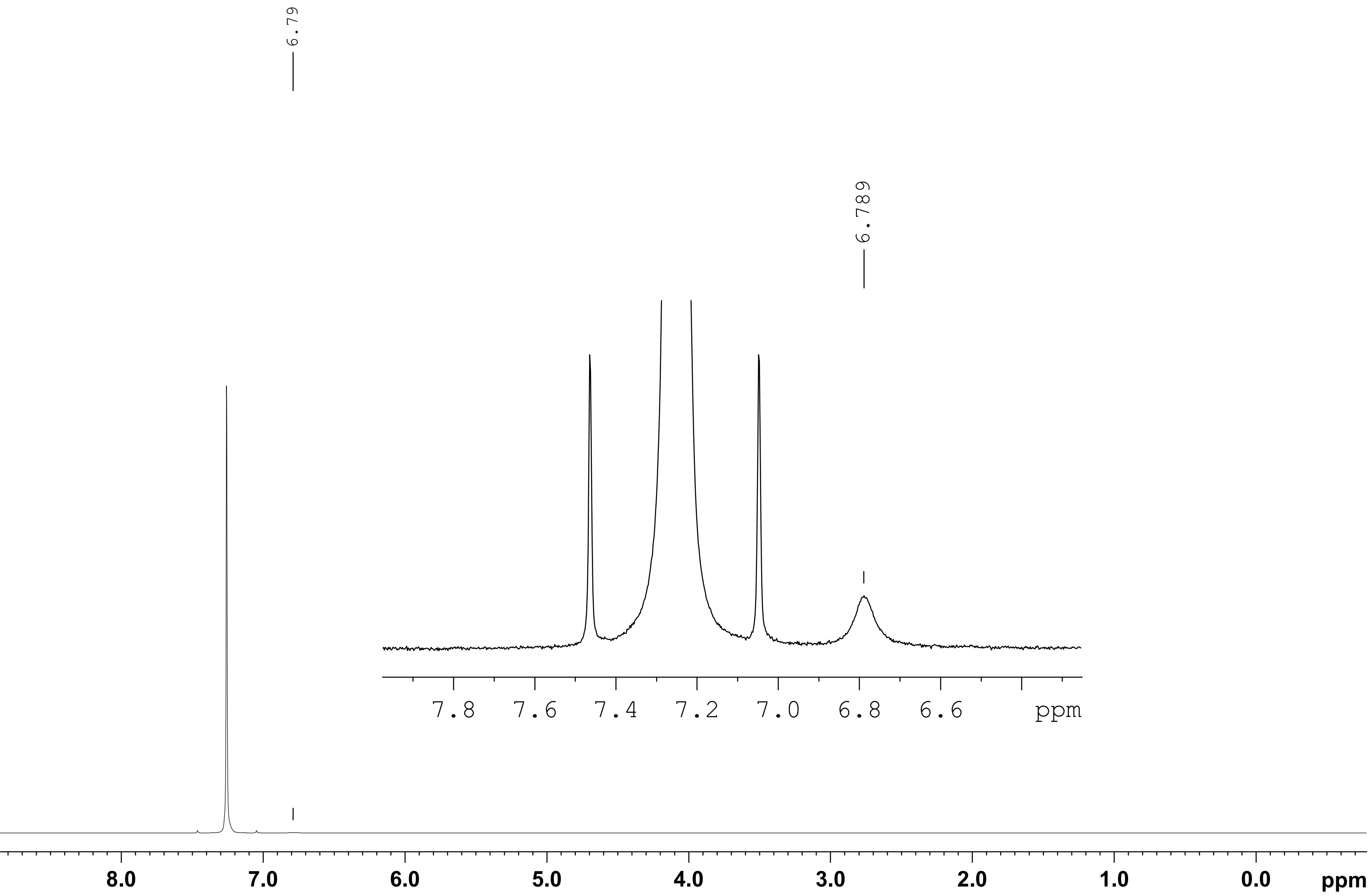

Figure S32. ^{1}H NMR spectrum (500 MHz, $CDCl_3$, 298 K) of **3ba**. (# = H_2O)

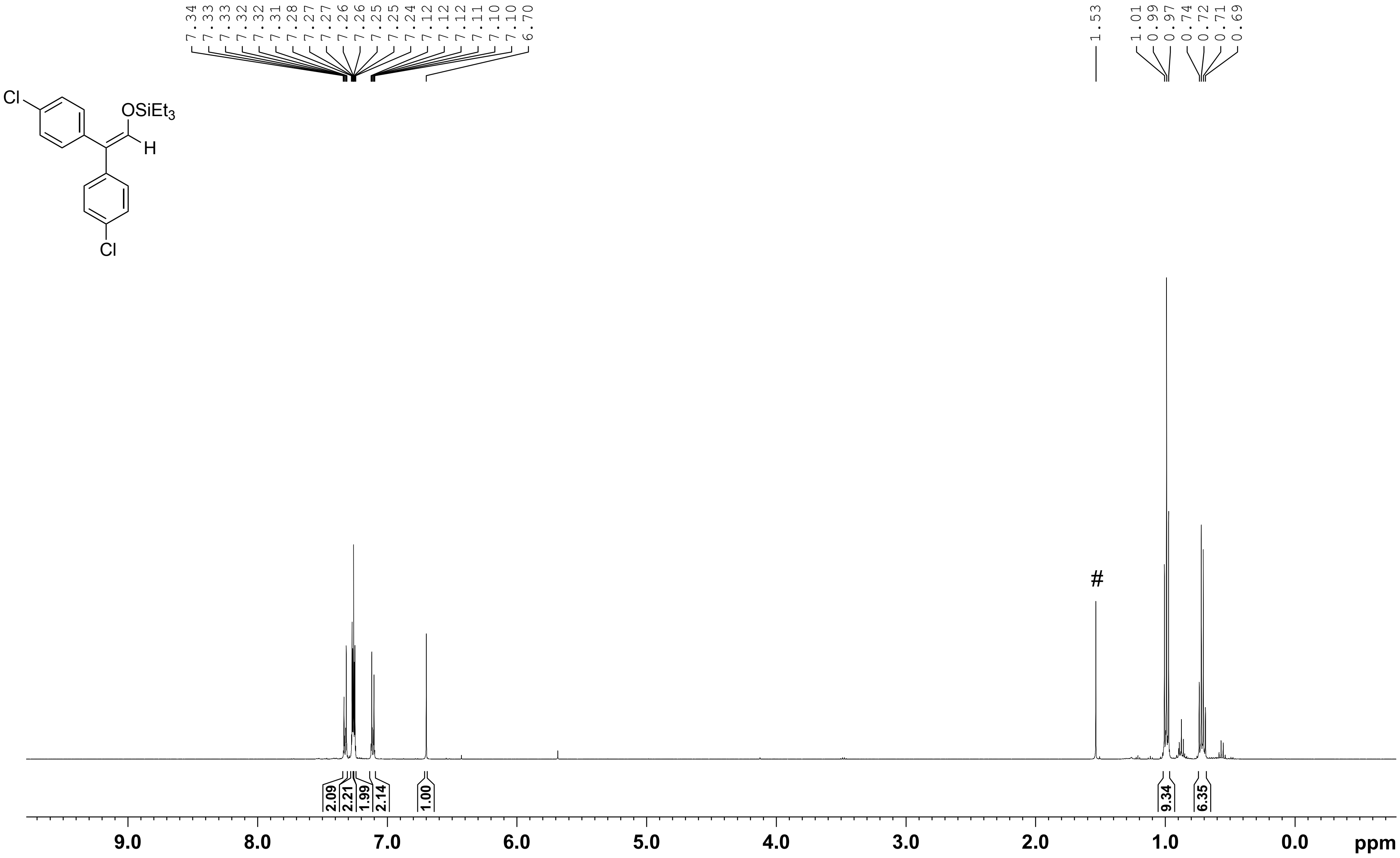

Figure S33. $^{13}C\{^{1}H\}$ NMR spectrum (126 MHz, $CDCl_3$, 298 K) of **3ba**.

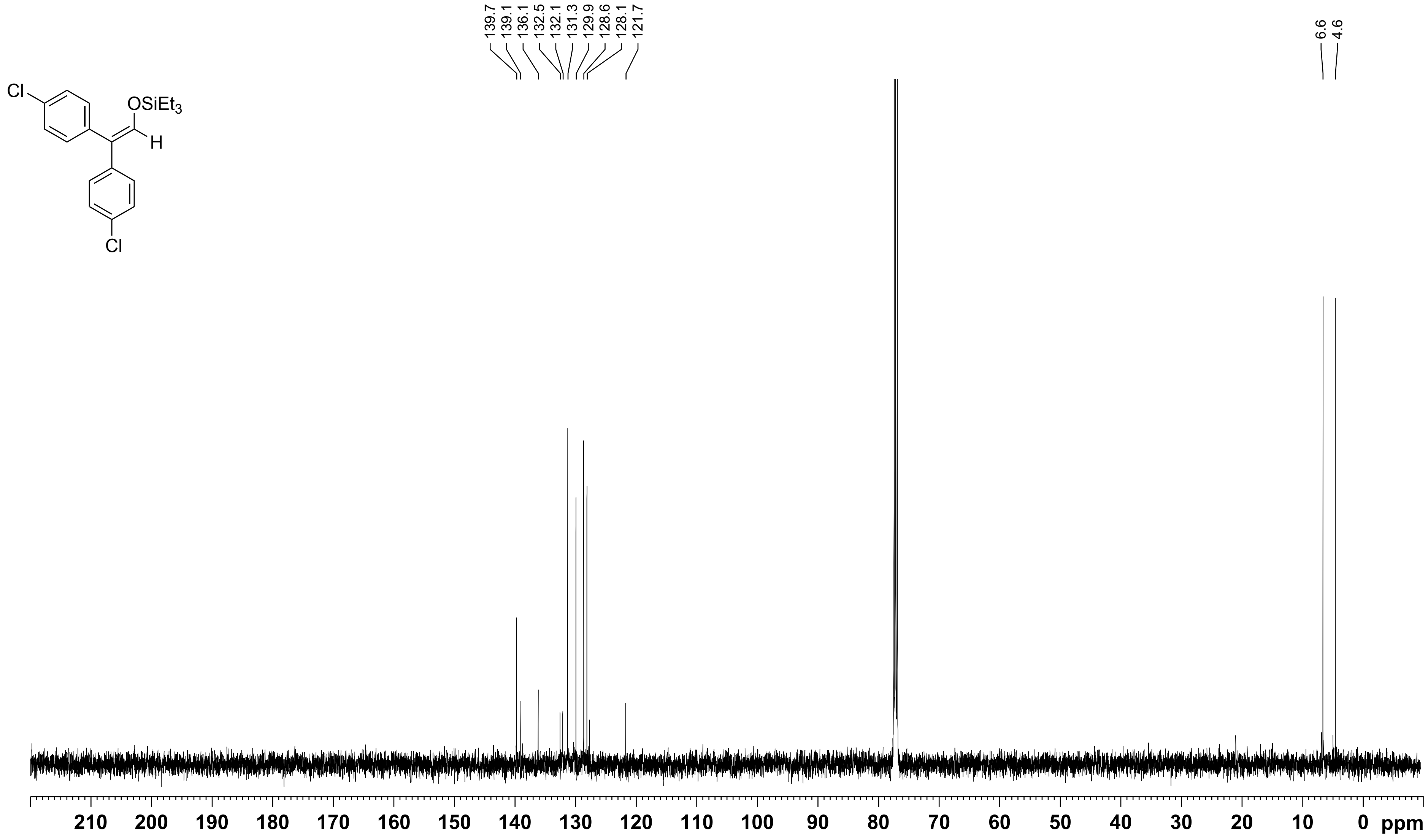

Figure S34. ^{29}Si DEPT NMR spectrum (99 MHz, $CDCl_3$, 298 K, optimized for J = 7 Hz) of **3ba**.

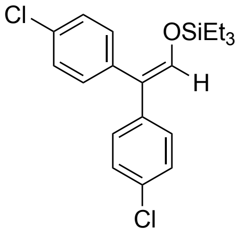

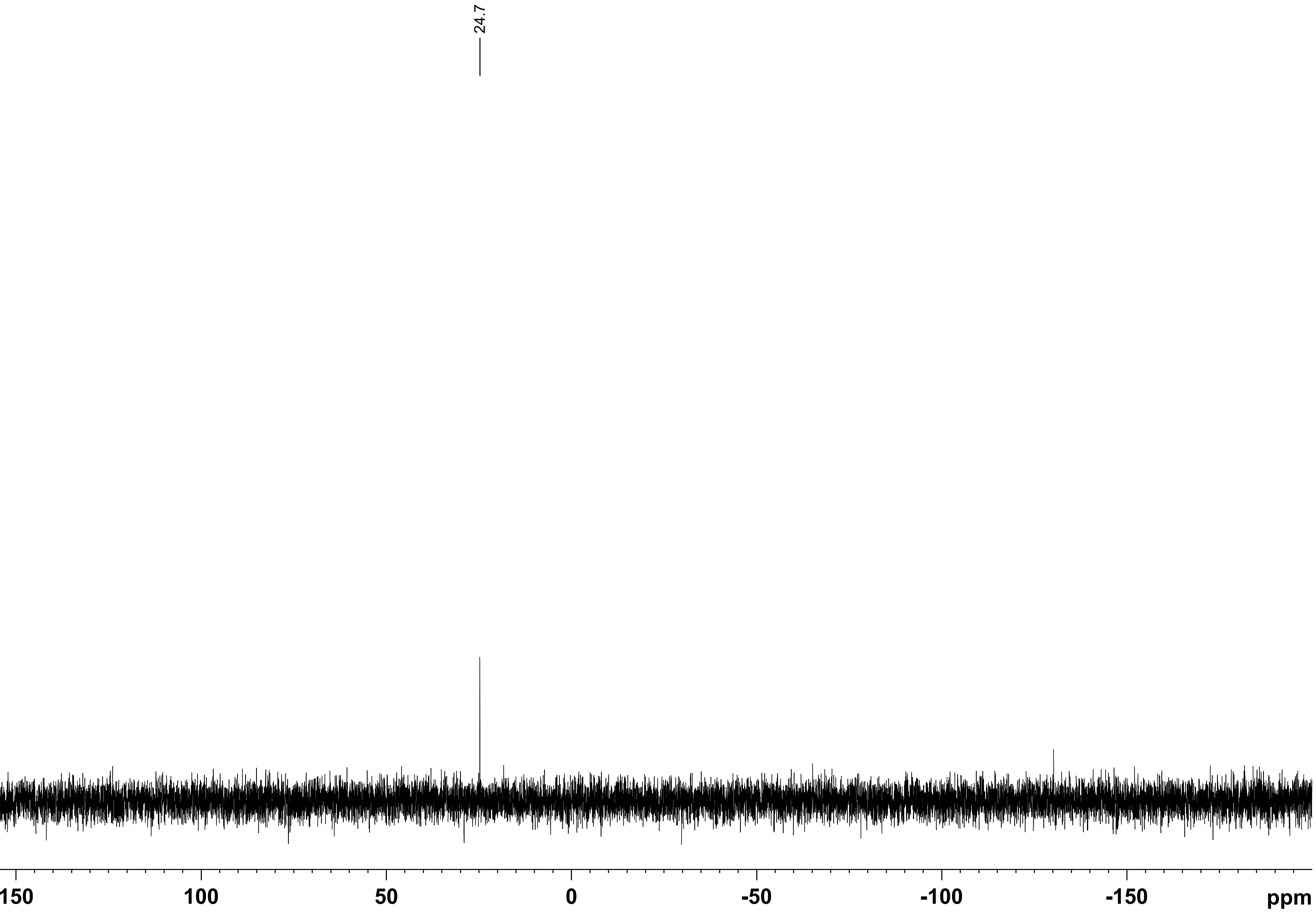

Figure S35. ^{1}H NMR spectrum (500 MHz, $CDCl_3$, 298 K) of **3ca**.

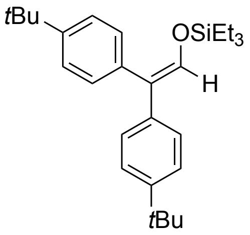

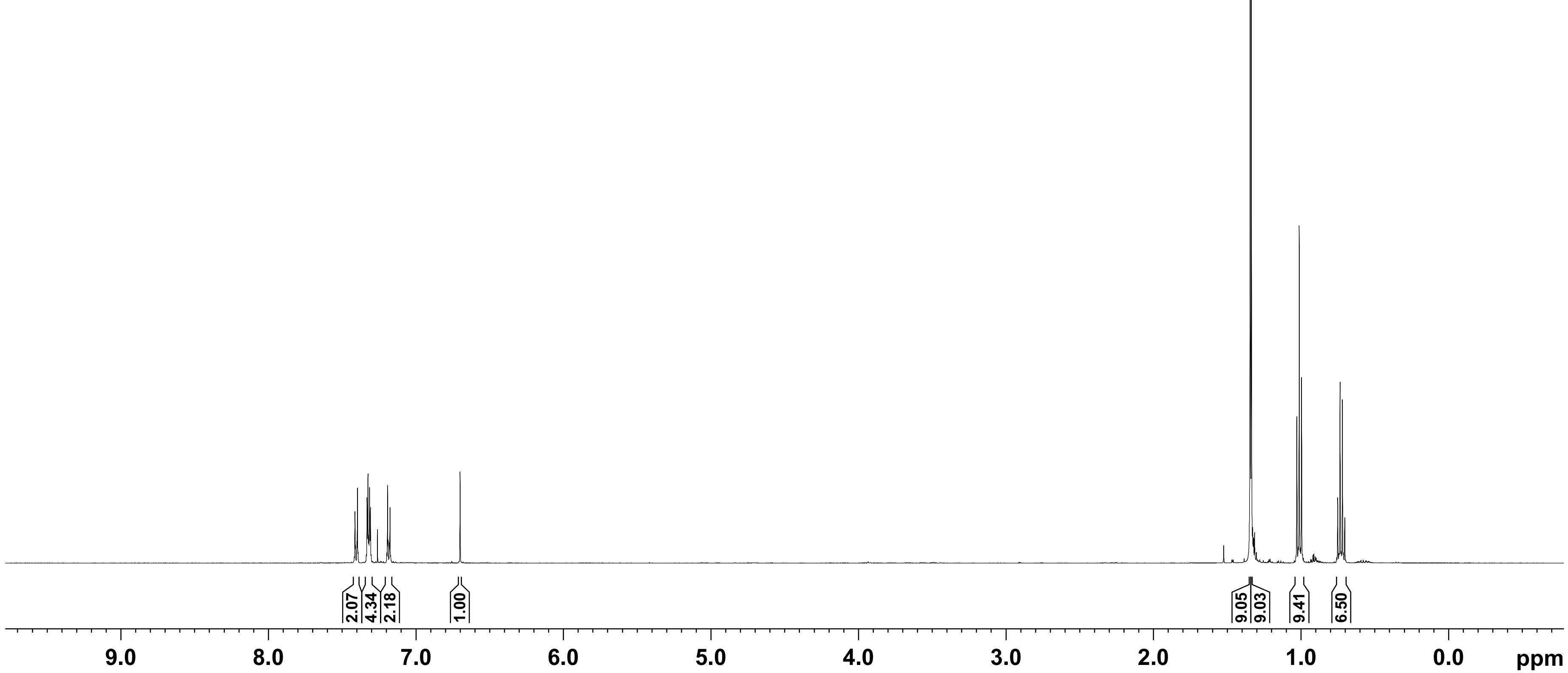

Figure S36. $^{13}C\{^{1}H\}$ NMR spectrum (126 MHz, $CDCl_3$, 298 K) of **3ca**.

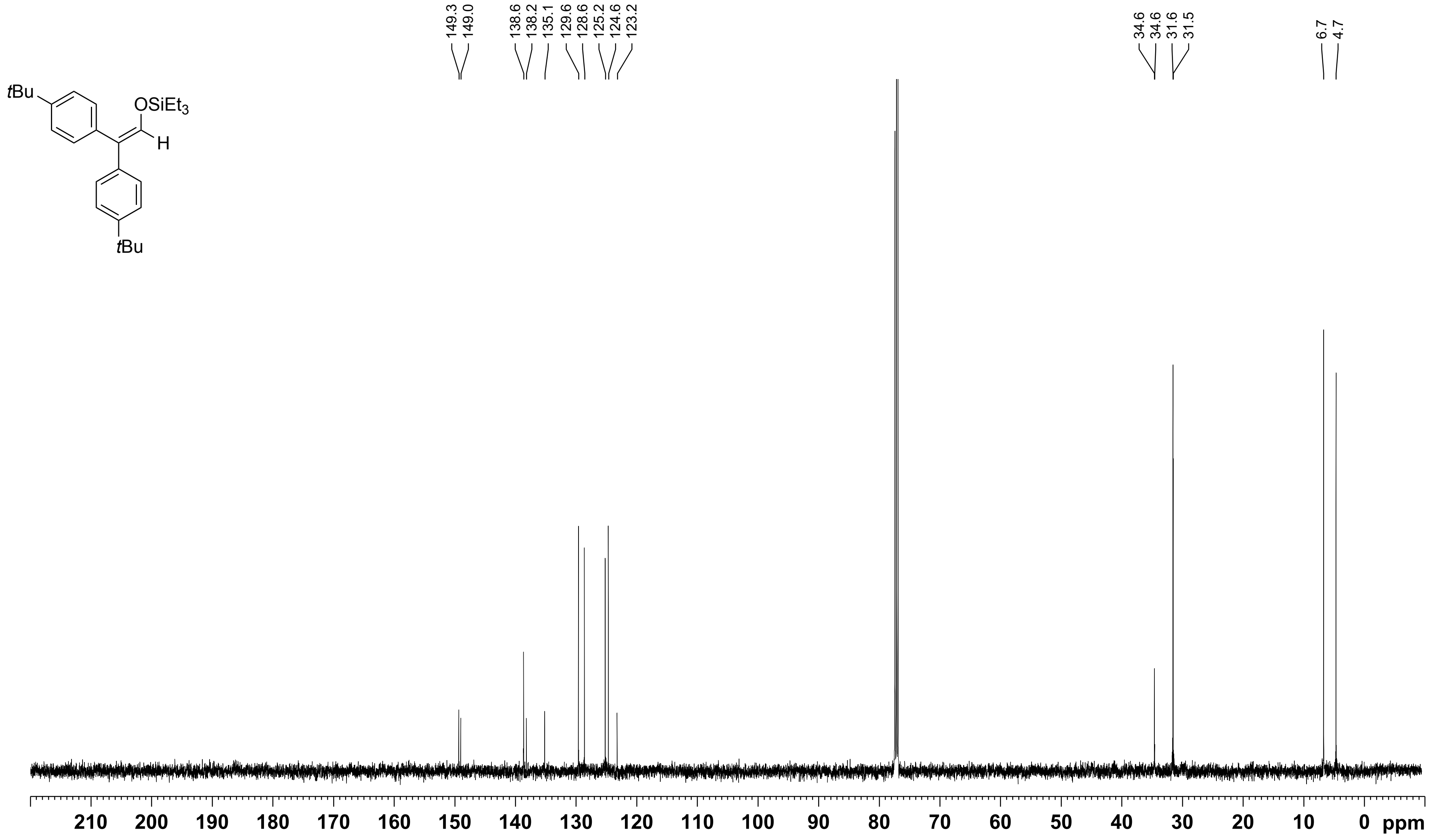

Figure S37. ^{29}Si DEPT NMR spectrum (99 MHz, $CDCl_3$, 298 K, optimized for J = 7 Hz) of **3ca**.

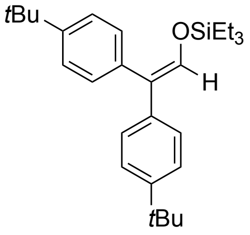

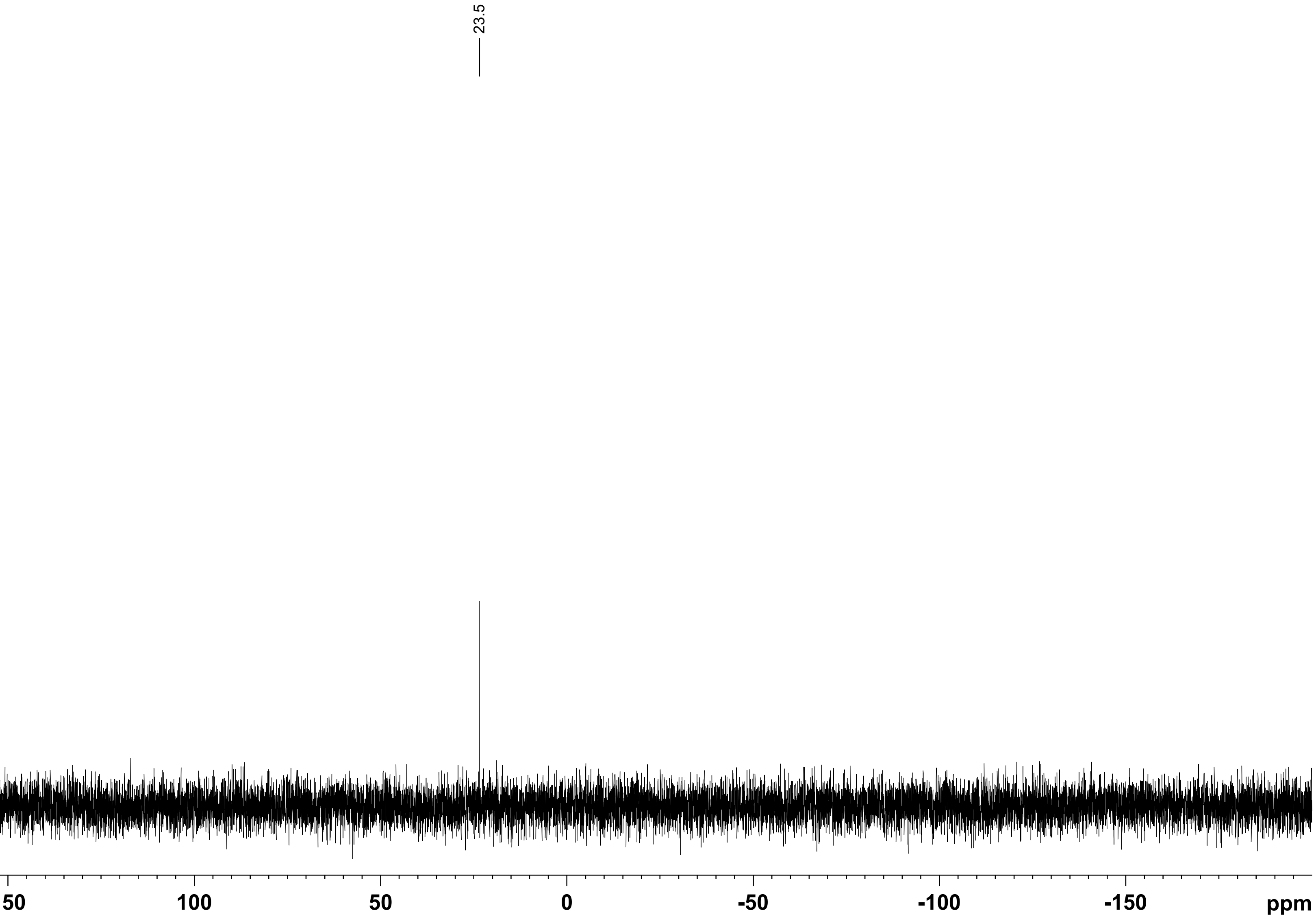

Figure S38. ^1H NMR spectrum (500 MHz, $CDCl_3$, 298 K) of **3da**.

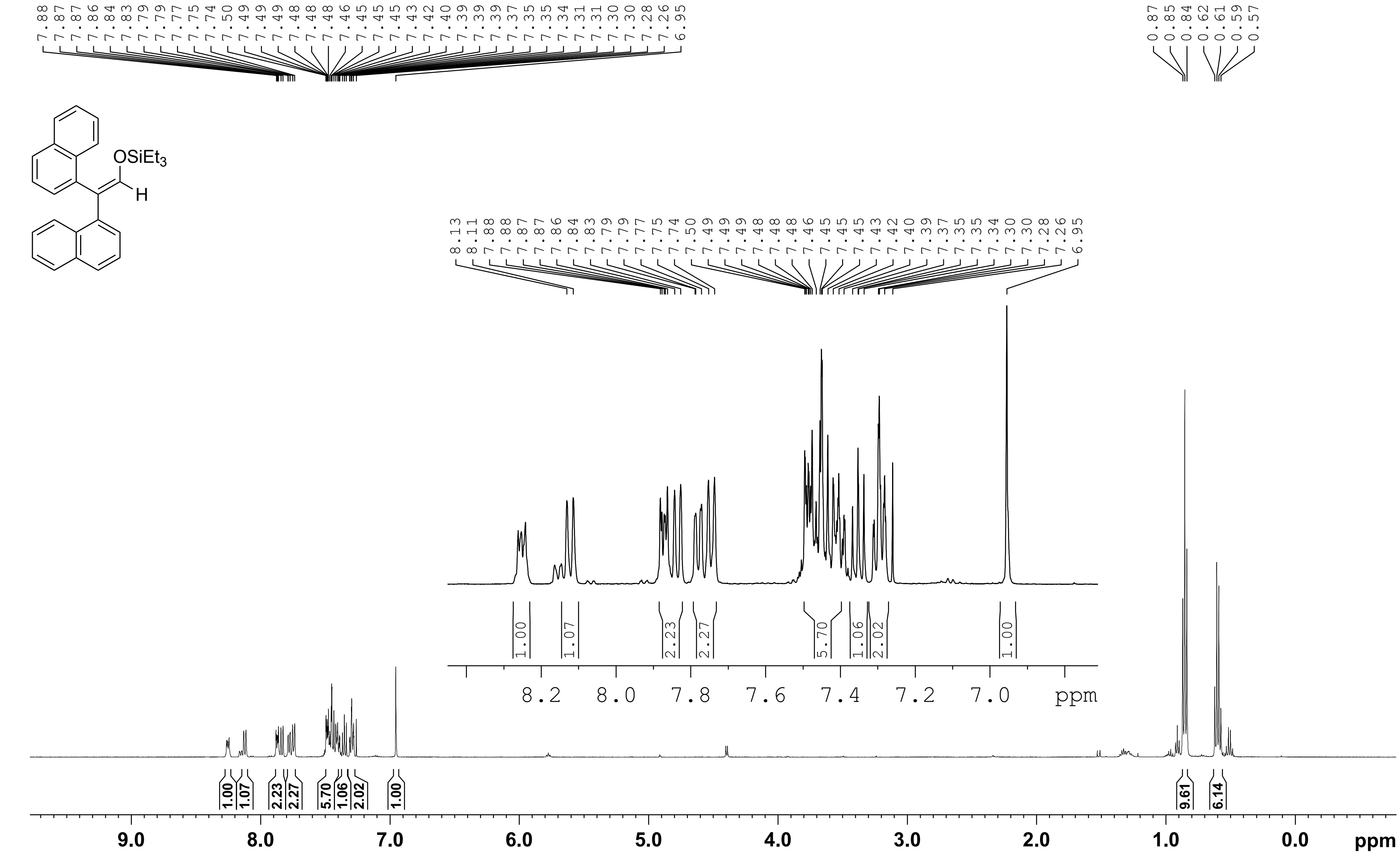

Figure S39. $^{13}C\{^{1}H\}$ NMR spectrum (126 MHz, $CDCl_3$, 298 K) of **3da**. (# = *n*-pentane)

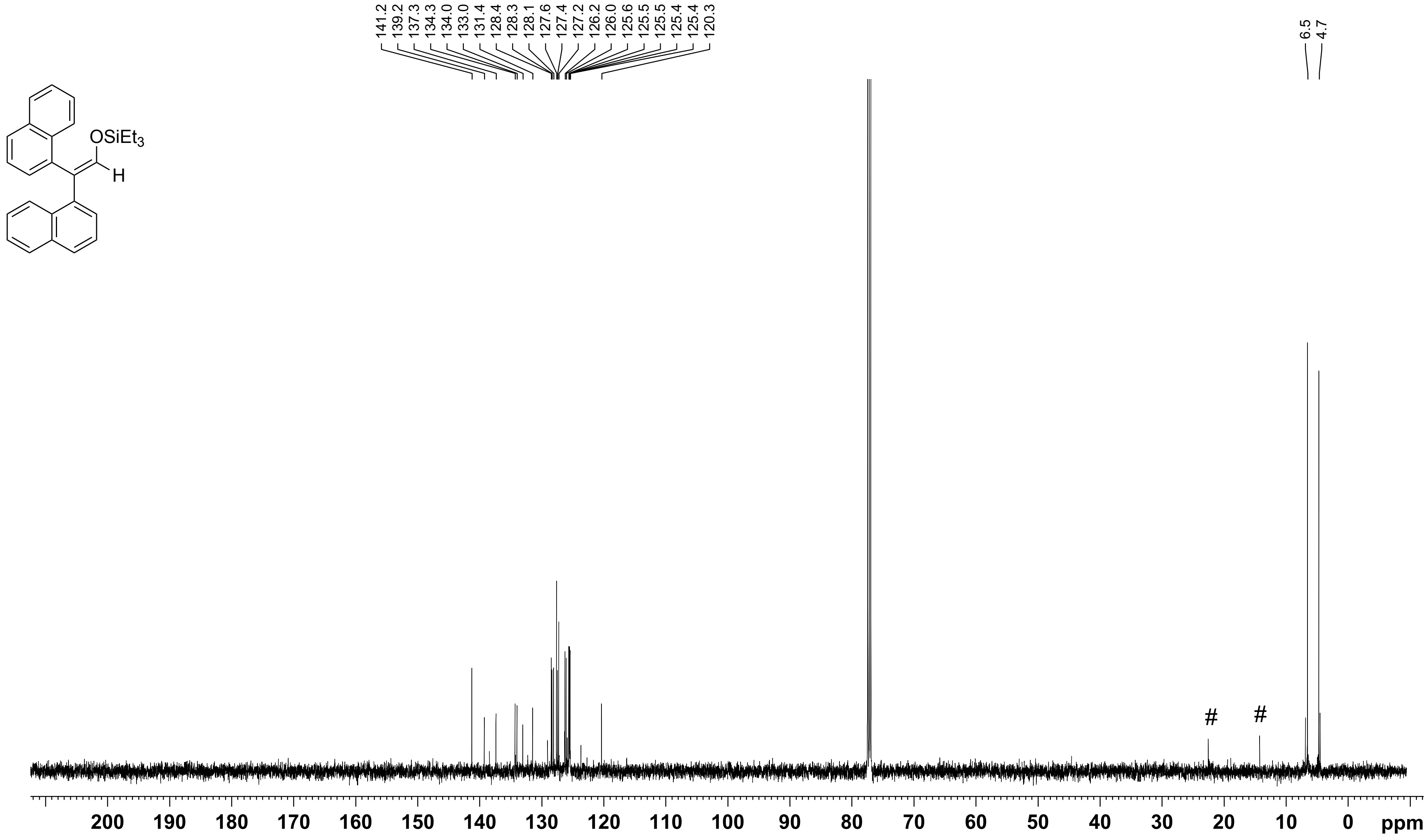

Figure S40. ^{29}Si DEPT NMR spectrum (99 MHz, $CDCl_3$, 298 K, optimized for J = 7 Hz) of **3da**.

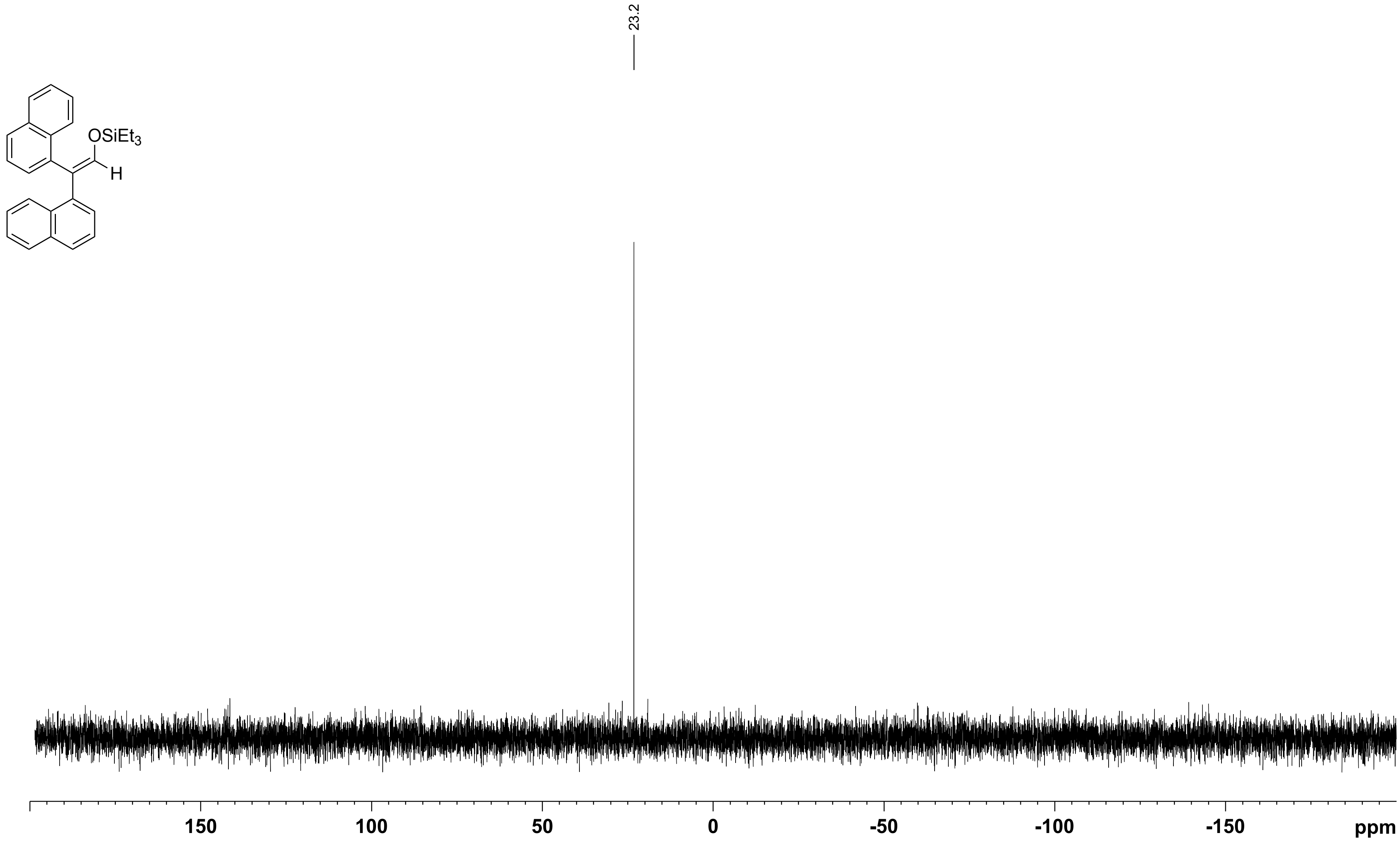

Figure S41. ^{1}H NMR spectrum (500 MHz, $CDCl_3$, 298 K) of **3ea** (*Z*:*E* = 62:38). (* = $(Et_3Si)_2O$)

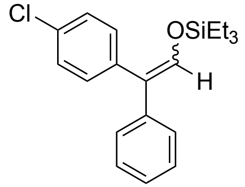

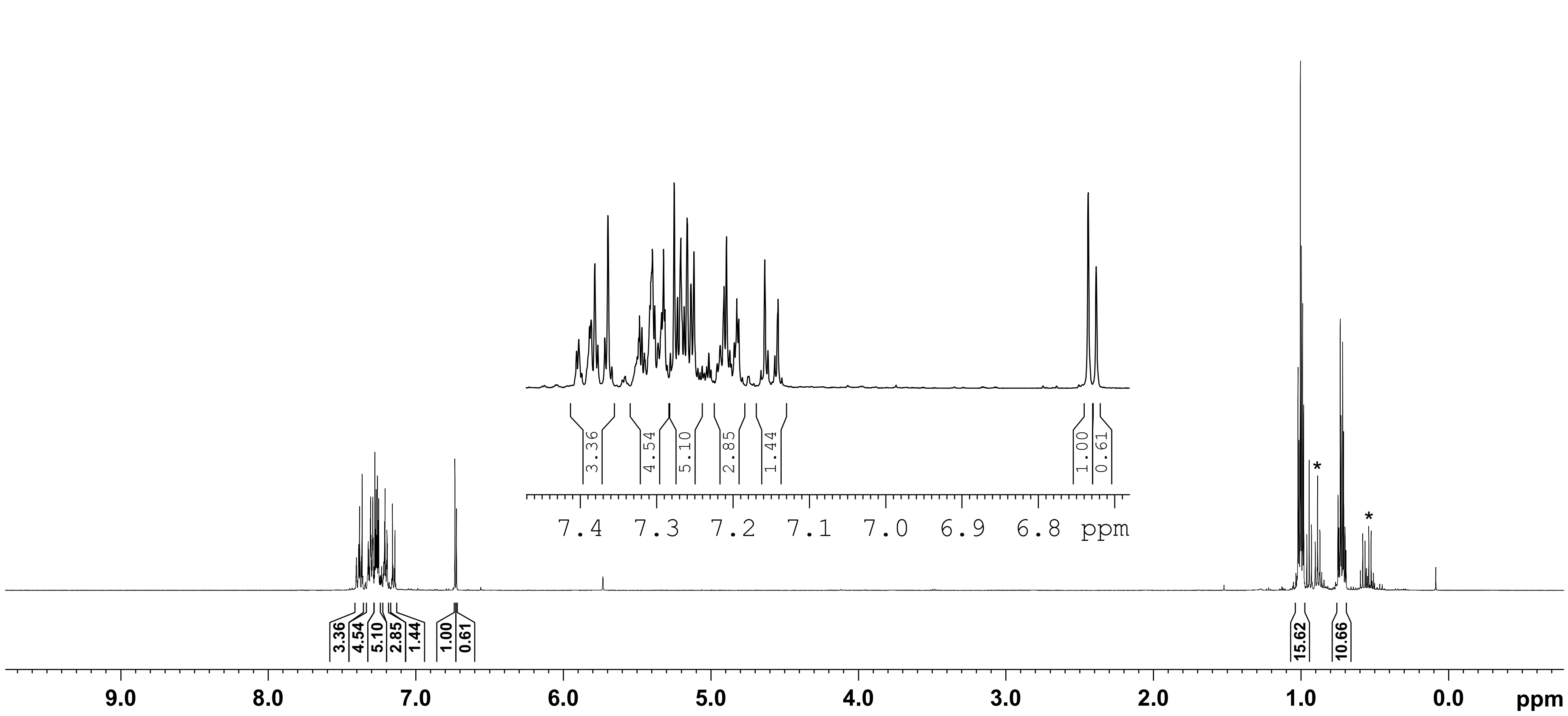

Figure S42. $^{13}C\{^1H\}$ NMR spectrum (126 MHz, $CDCl_3$, 298 K) of **3ea** (*Z*:*E* = 62:38). (* = $(Et_3Si)_2O$)

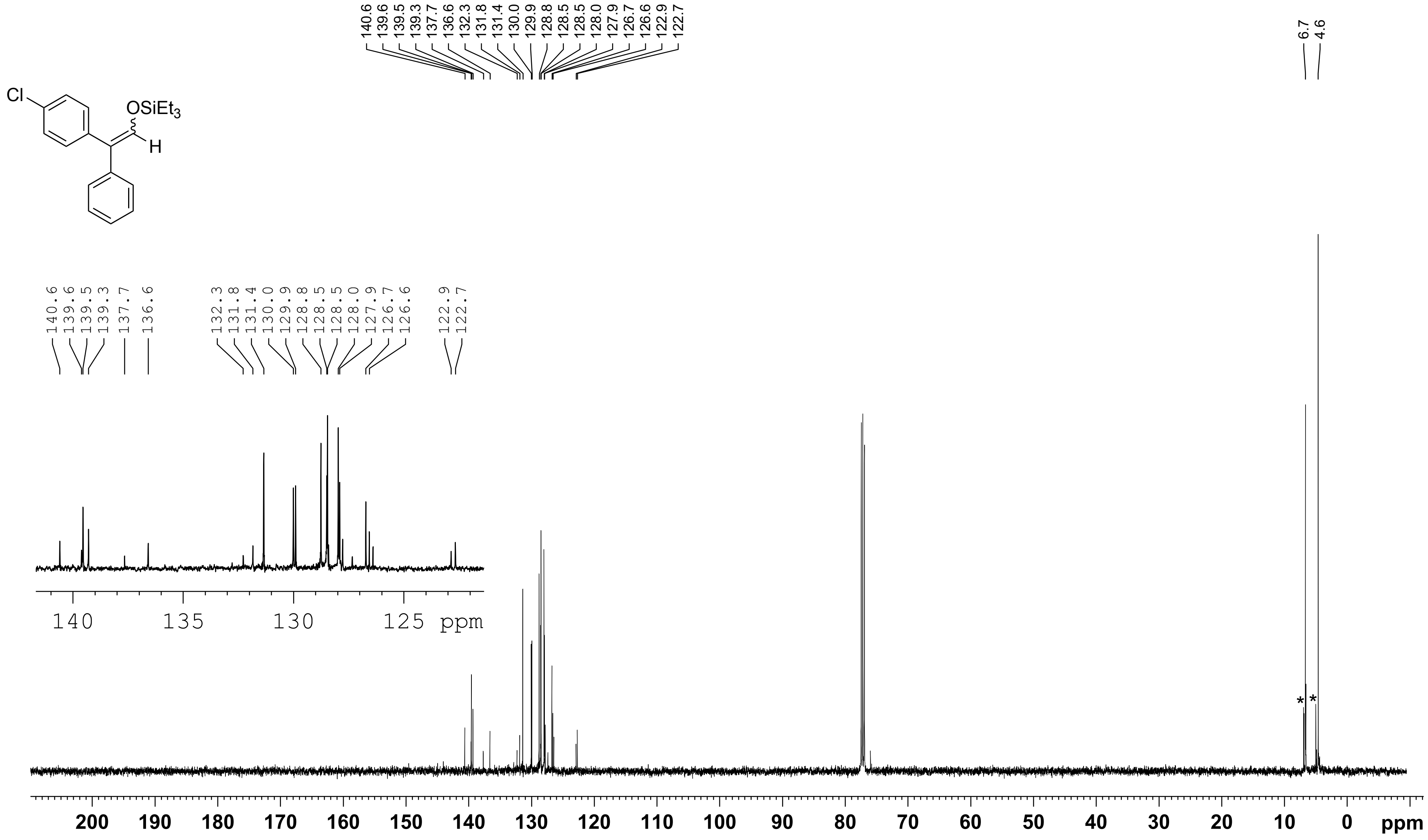

Figure S43. ^{29}Si DEPT NMR spectrum (99 MHz, $CDCl_3$, 298 K, optimized for J = 7 Hz) of **3ea** (Z:E = 62:38).

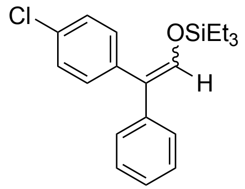

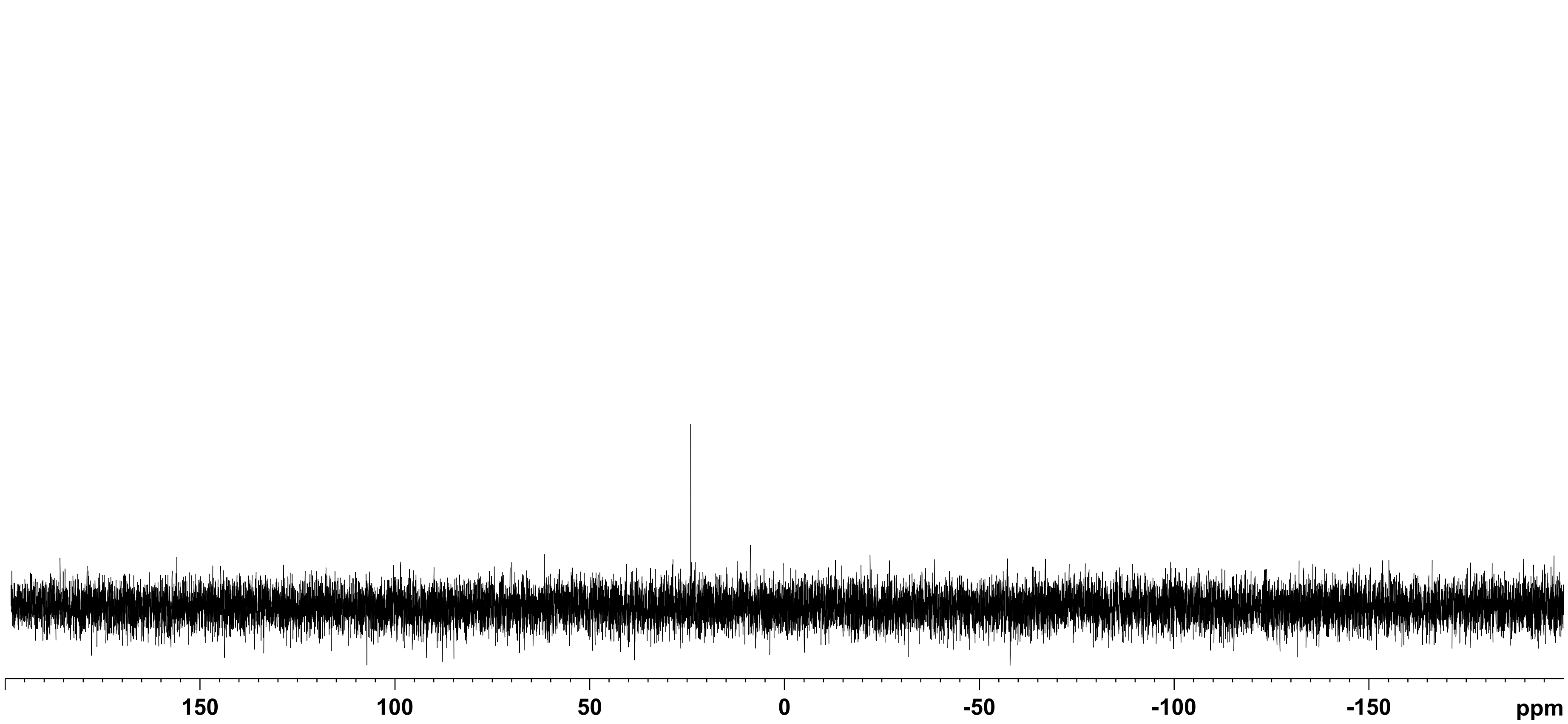

Figure S44. 2D-NOESY NMR spectrum (126 MHz, $CDCl_3$, 298 K) of **3ea** (*Z*:*E* = 62:38).

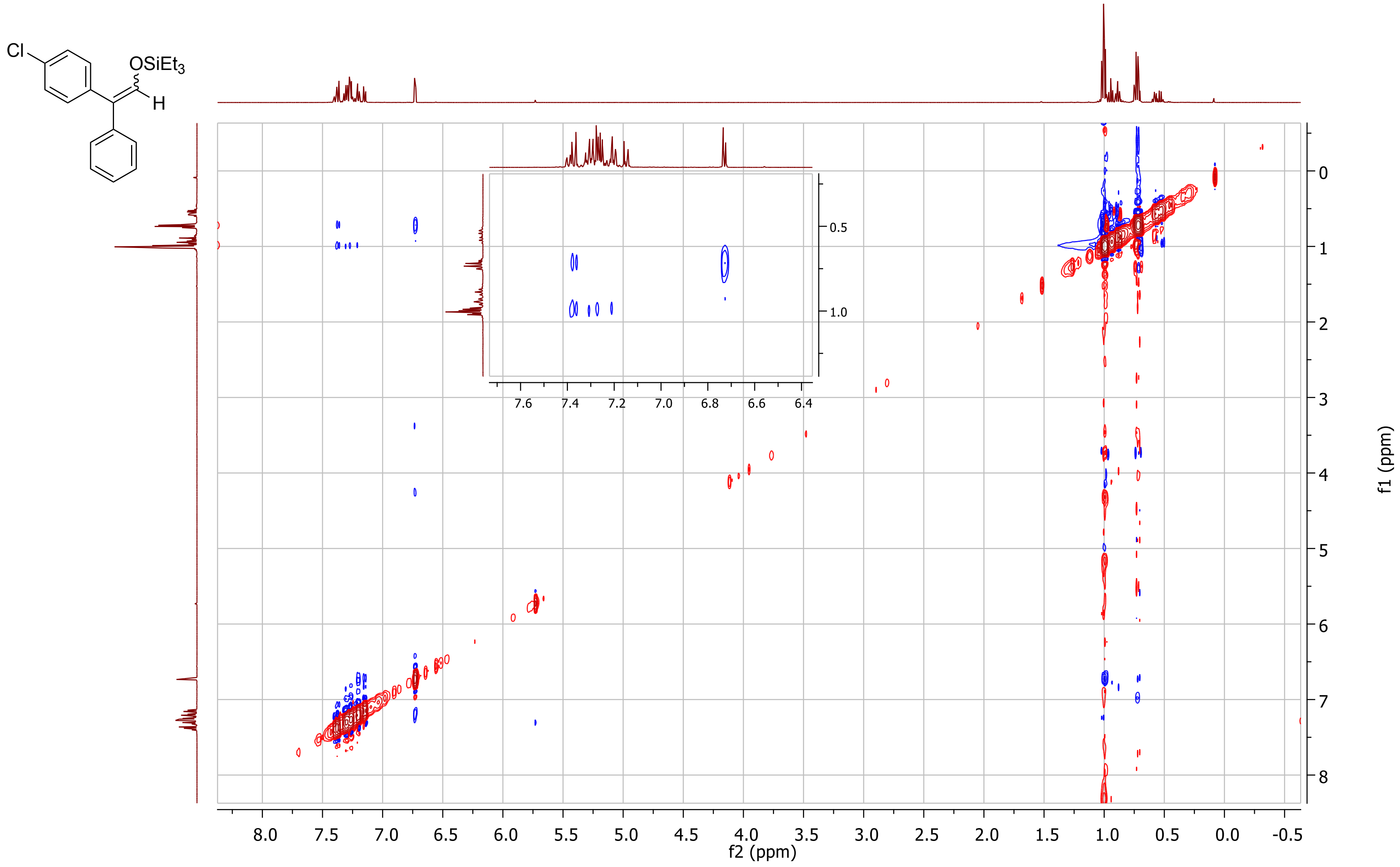

Figure S45. ^{1}H NMR spectrum (500 MHz, $CDCl_3$, 298 K) of **3fa** (*Z*:*E* = 79:21).

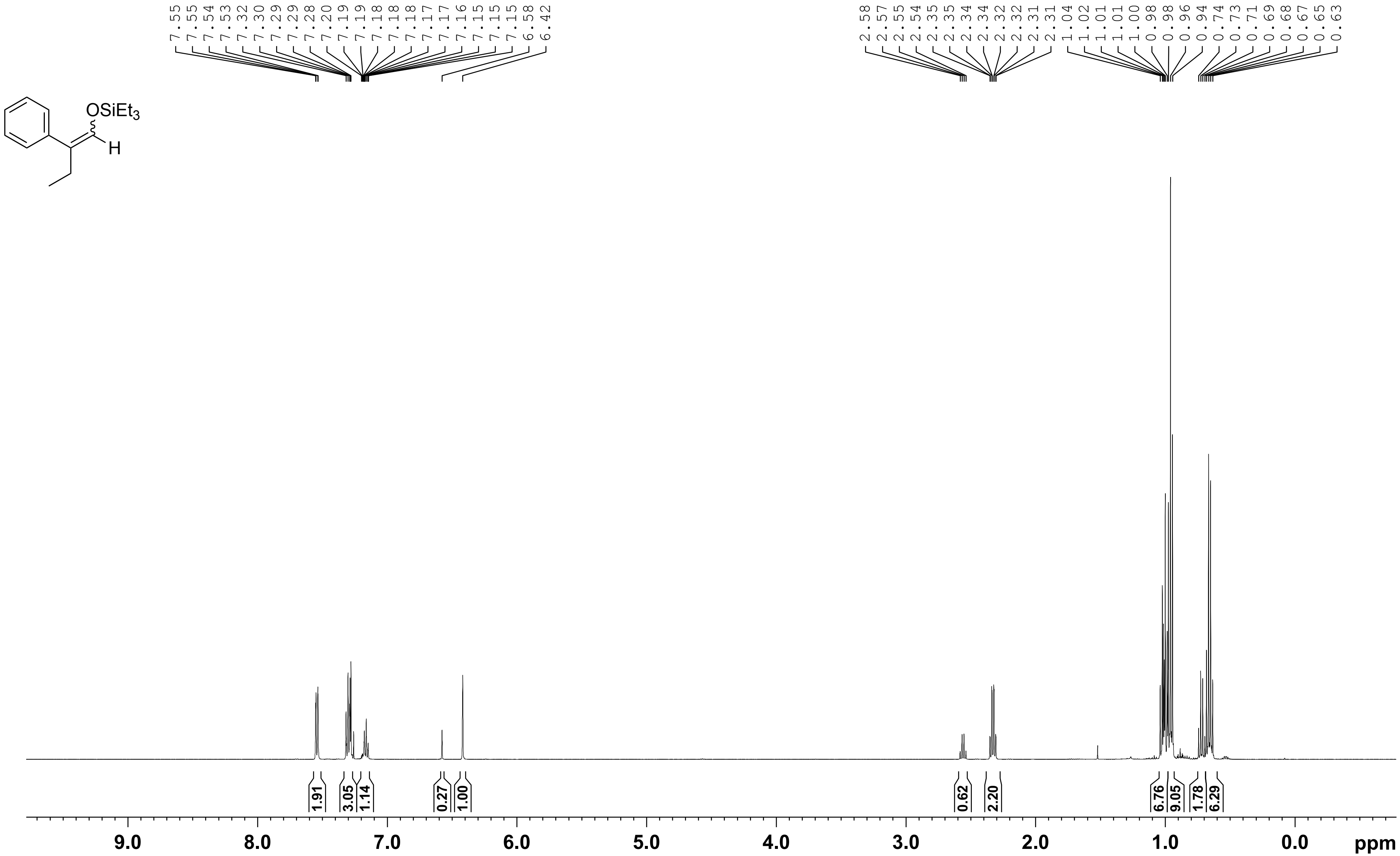

Figure S46. $^{13}C\{^{1}H\}$ NMR spectrum (126 MHz, $CDCl_3$, 298 K) of **3fa** (*Z*:*E* = 79:21).

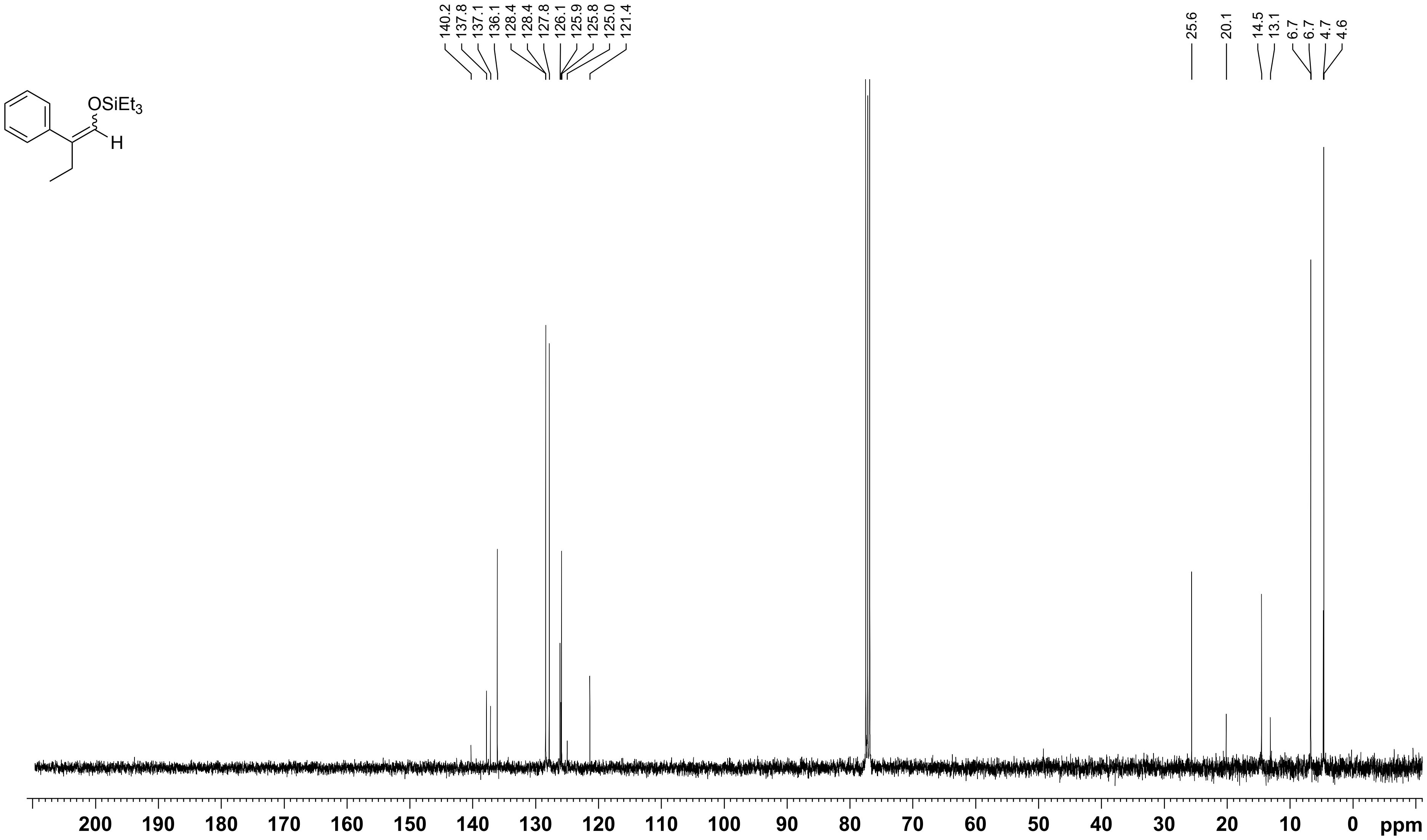

Figure S47. ^{29}Si DEPT NMR spectrum (99 MHz, $CDCl_3$, 298 K, optimized for *J* = 7 Hz) of **3fa** (*Z*:*E* = 79:21).

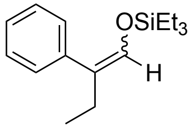

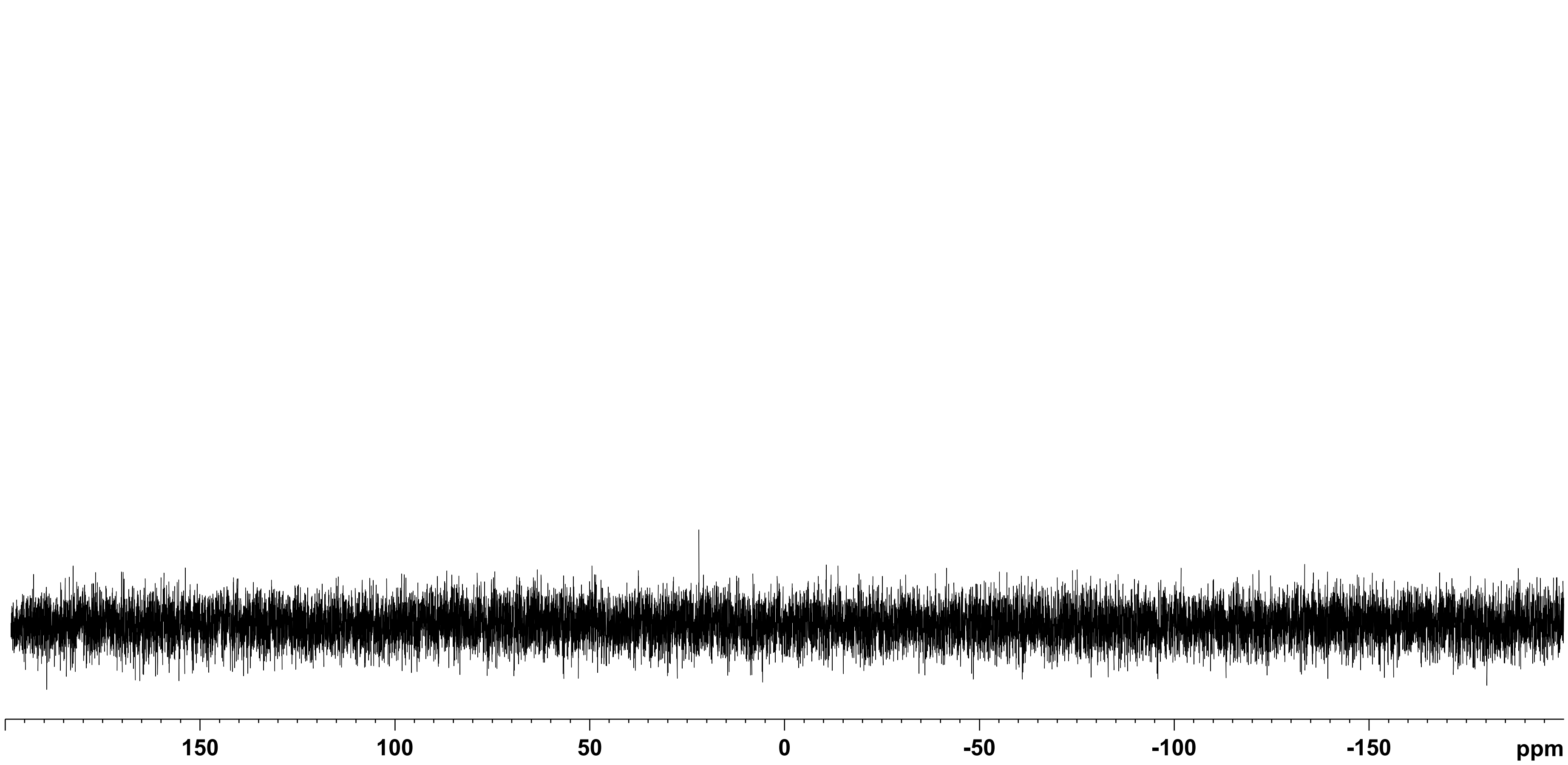

Figure S48. 2D-NOESY NMR spectrum (126 MHz, $CDCl_3$, 298 K) of **3fa** (*Z*:*E* = 79:21).

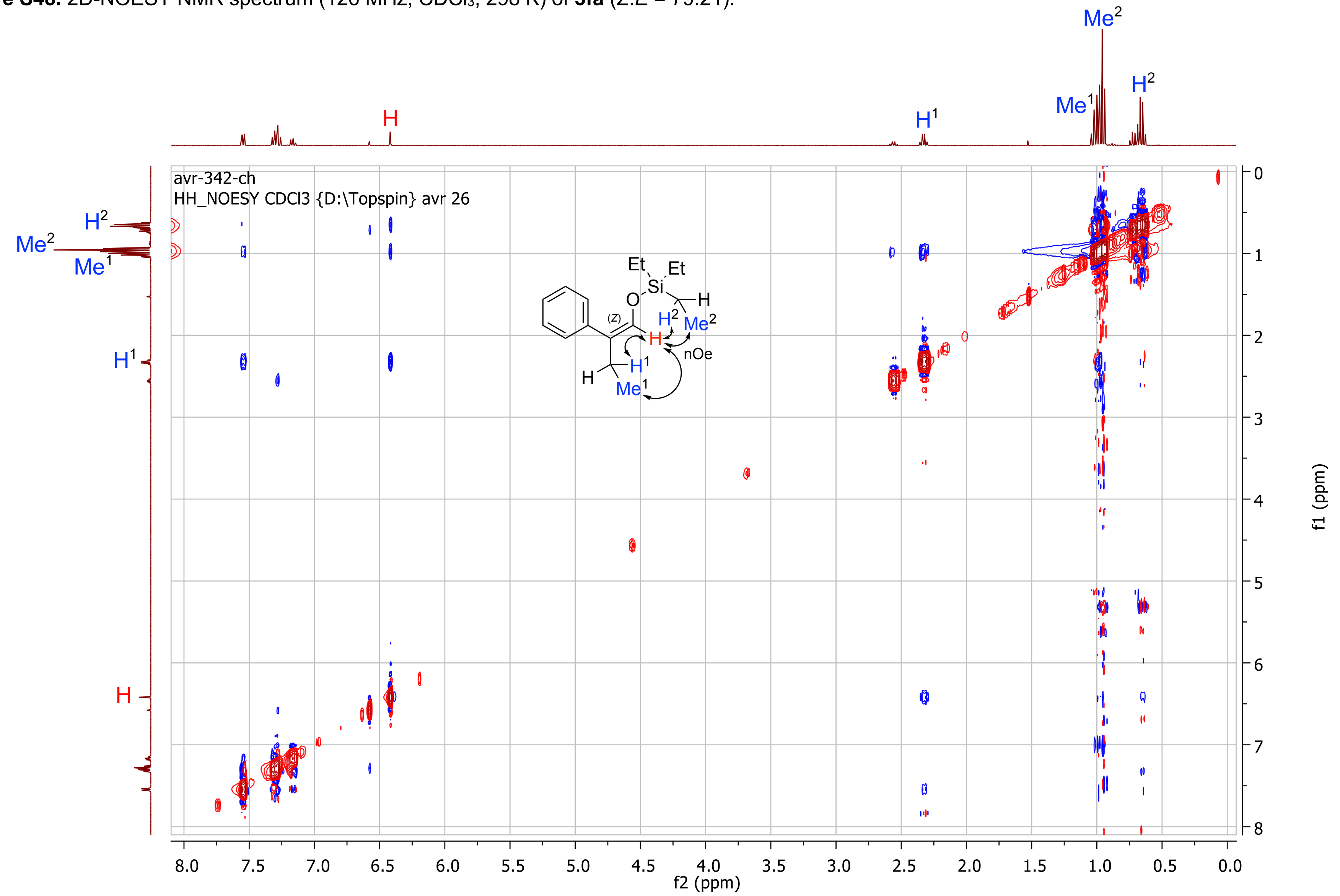

Figure S49. ^{1}H NMR spectrum (500 MHz, $CDCl_3$, 298 K) of **3fb** (*Z*:*E* = 81:19).

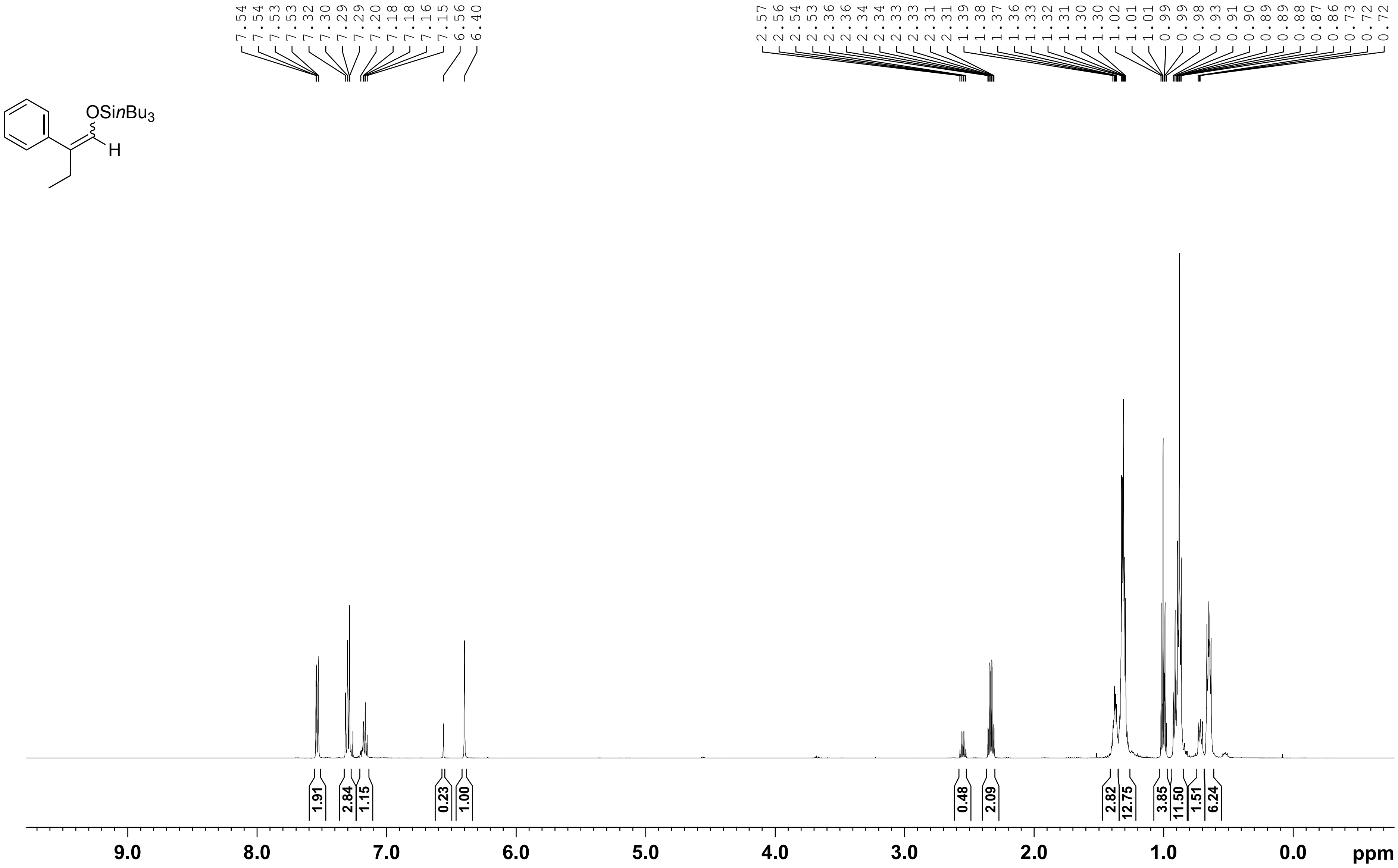

Figure S50. $^{13}C\{^1H\}$ NMR spectrum (126 MHz, $CDCl_3$, 298 K) of **3fb** (*Z*:*E* = 81:19).

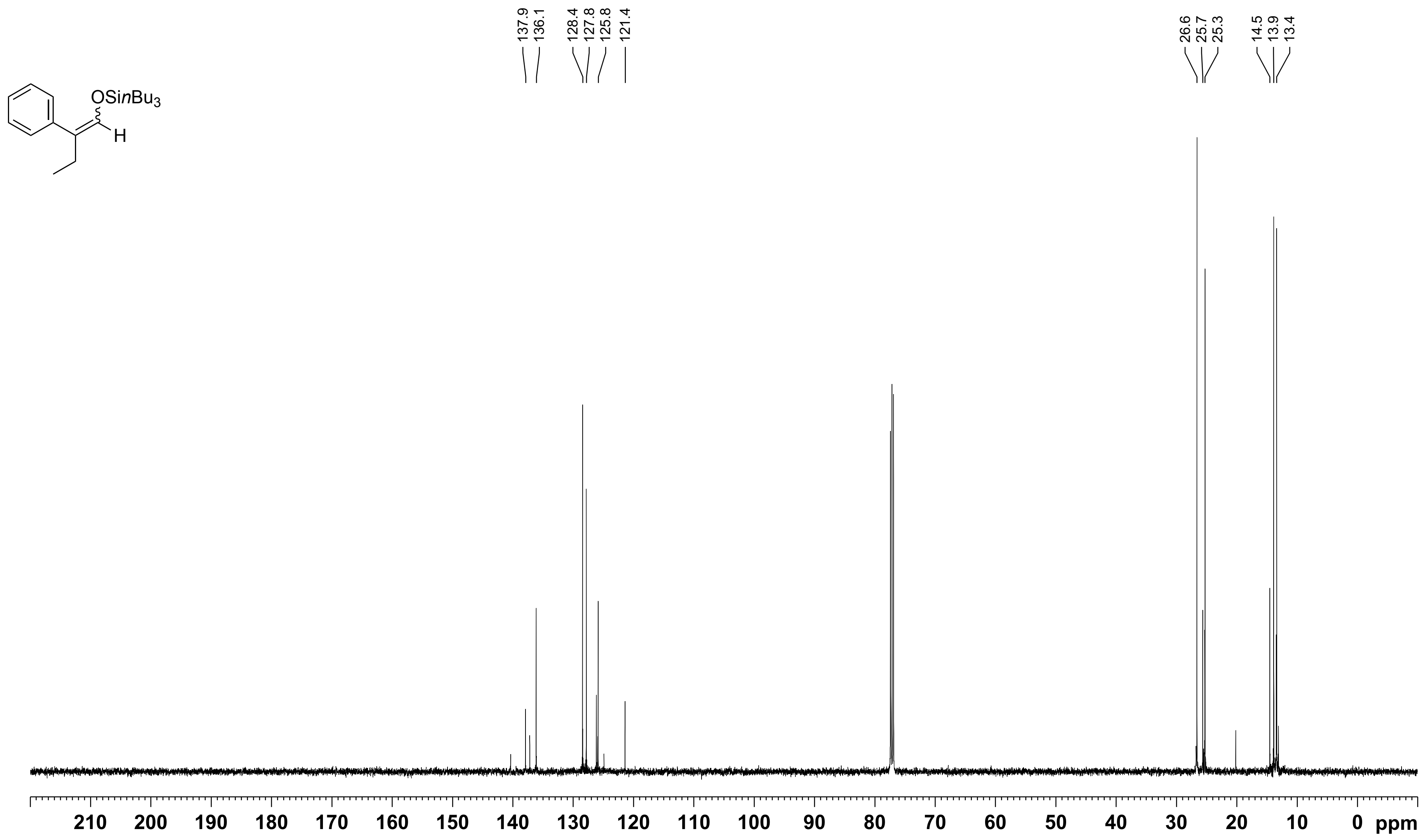

Figure S51. ^{29}Si DEPT NMR spectrum (99 MHz, $CDCl_3$, 298 K, optimized for *J* = 7 Hz) of **3fb** (*Z*:*E* = 81:19).

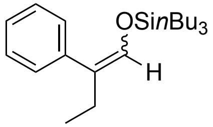

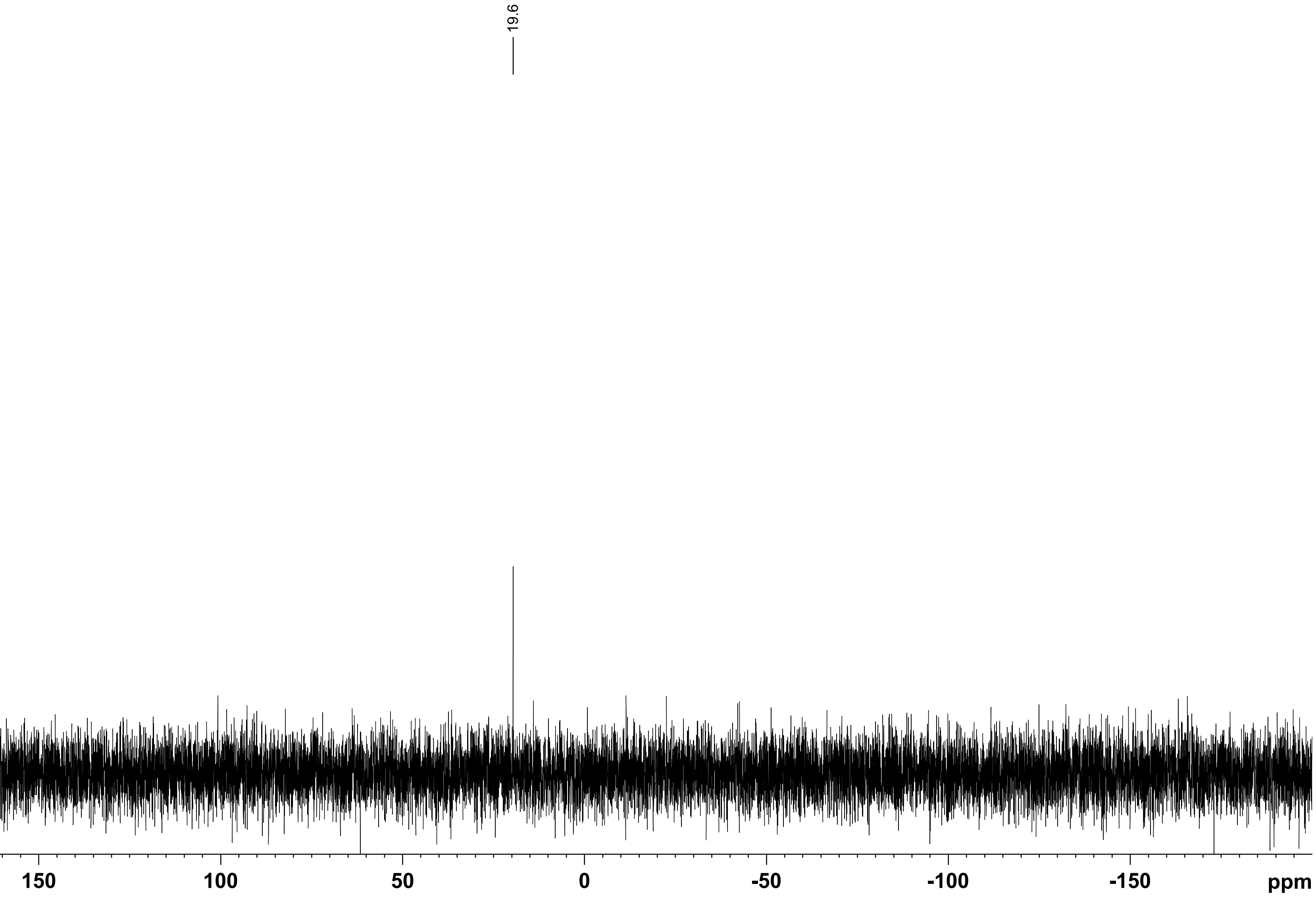

Figure S52. ^{1}H NMR spectrum (500 MHz, $CDCl_3$, 298 K) of **3fd** (*E*:*Z* = 72:28).

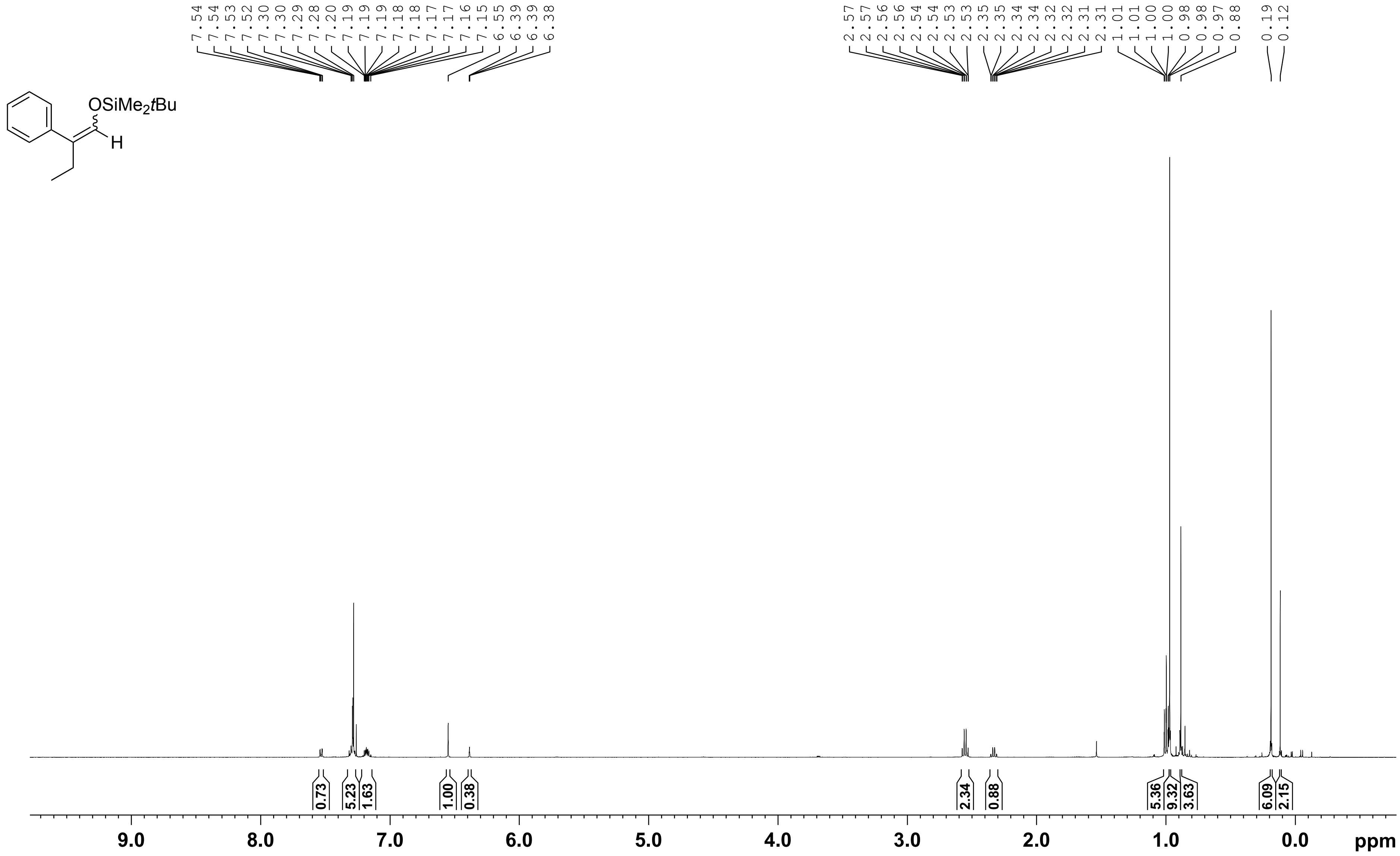

Figure S53. $^{13}C\{^1H\}$ NMR spectrum (126 MHz, $CDCl_3$, 298 K) of **3fd** (*E*:*Z* = 72:28).

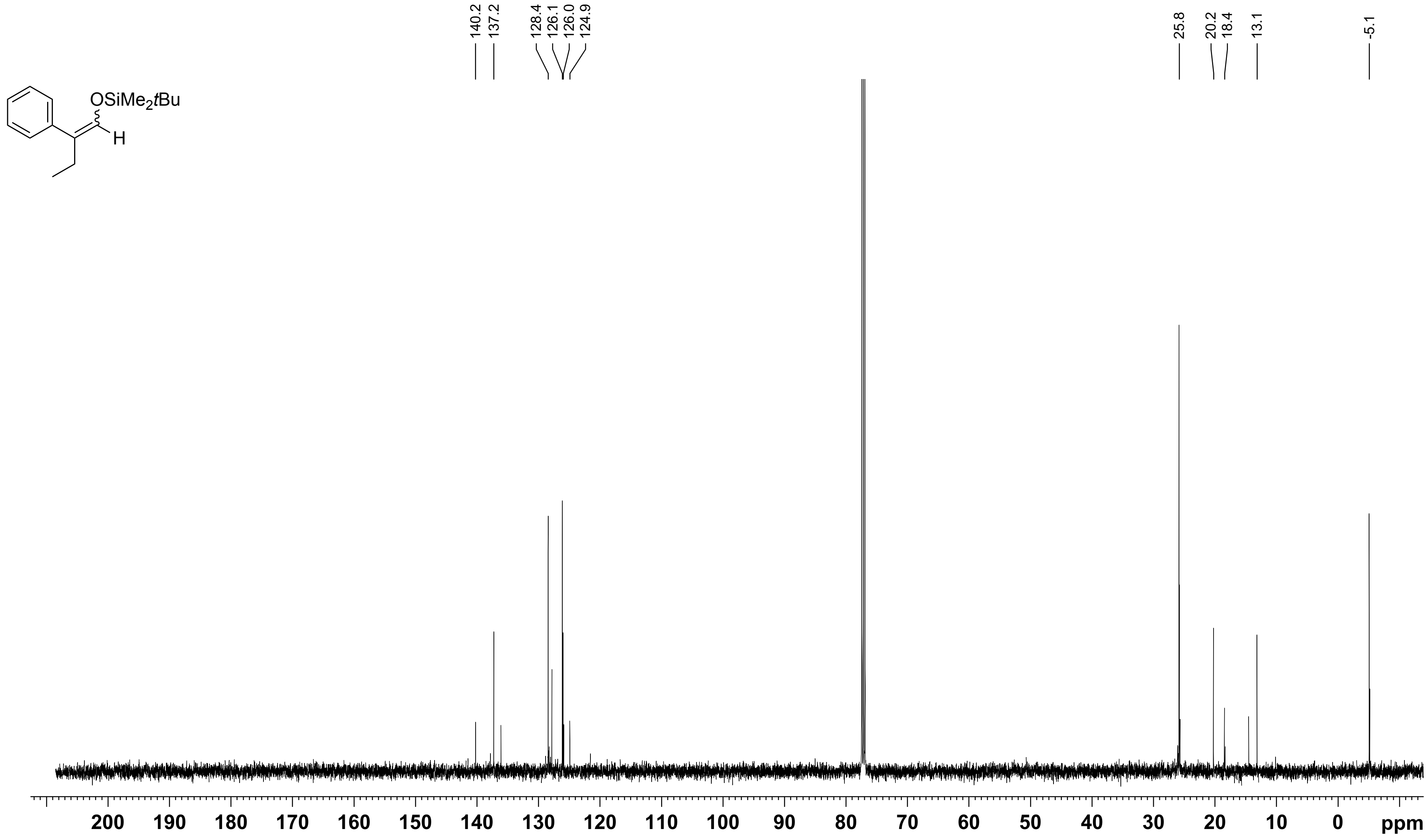

Figure S54. ^{29}Si DEPT NMR spectrum (99 MHz, $CDCl_3$, 298 K, optimized for J = 7 Hz) of **3fd** (E:Z = 72:28).

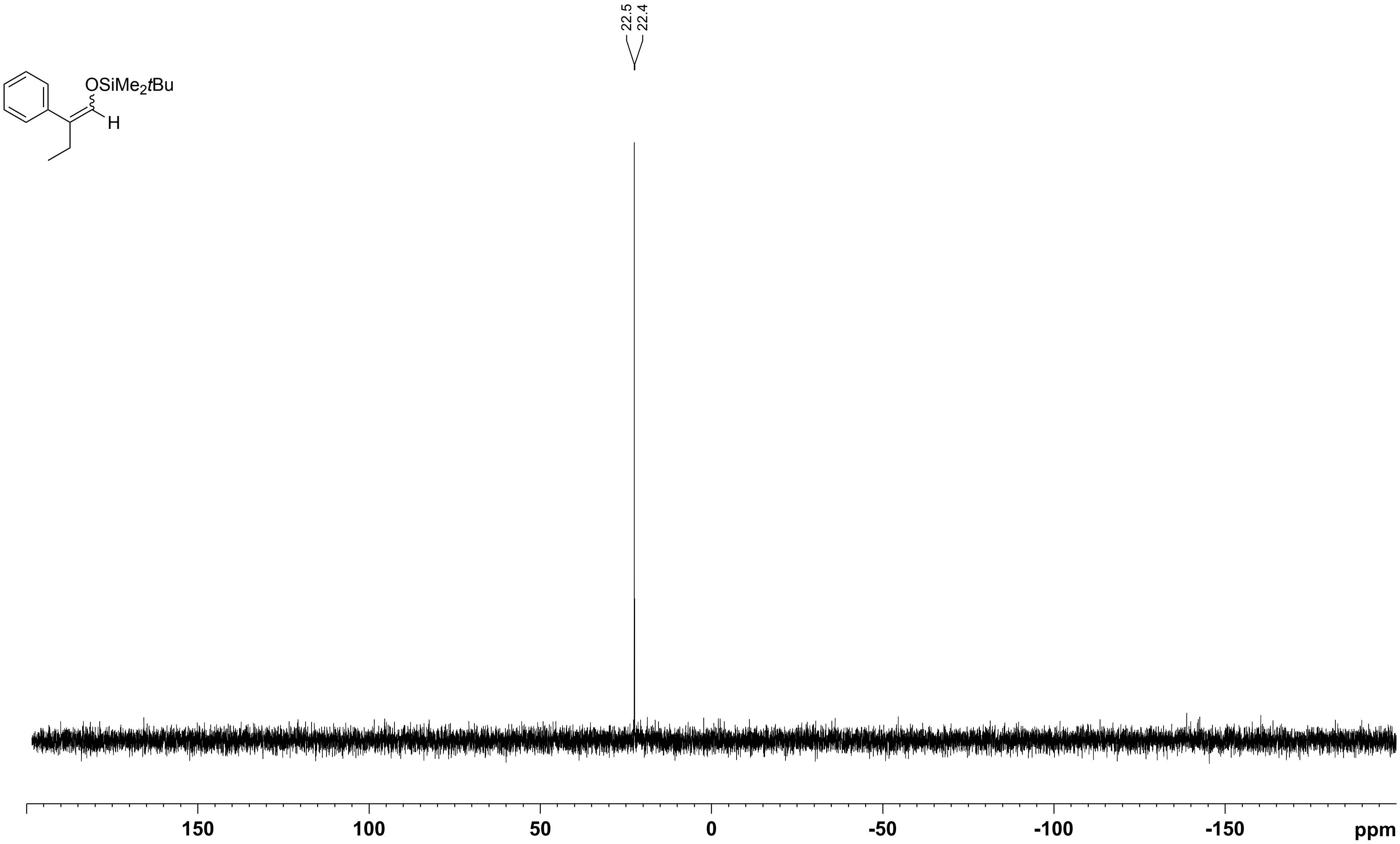

Figure S55. ^{1}H NMR spectrum (500 MHz, $CDCl_3$, 298 K) of **3ga** (*Z*:*E* = 72:28).

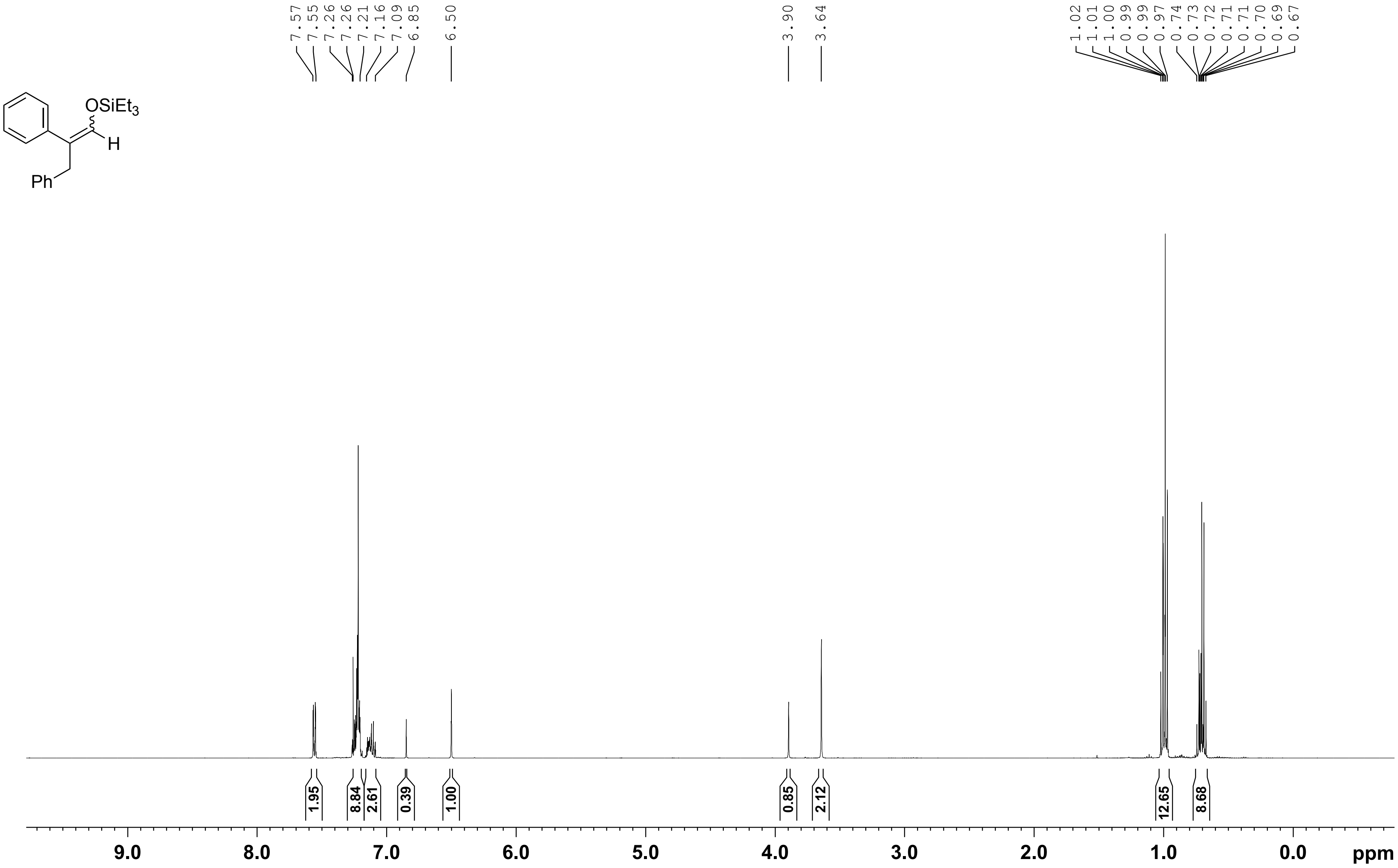

Figure S56. $^{13}C\{^1H\}$ NMR spectrum (126 MHz, $CDCl_3$, 298 K) of **3ga** (*Z*:*E* = 72:28).

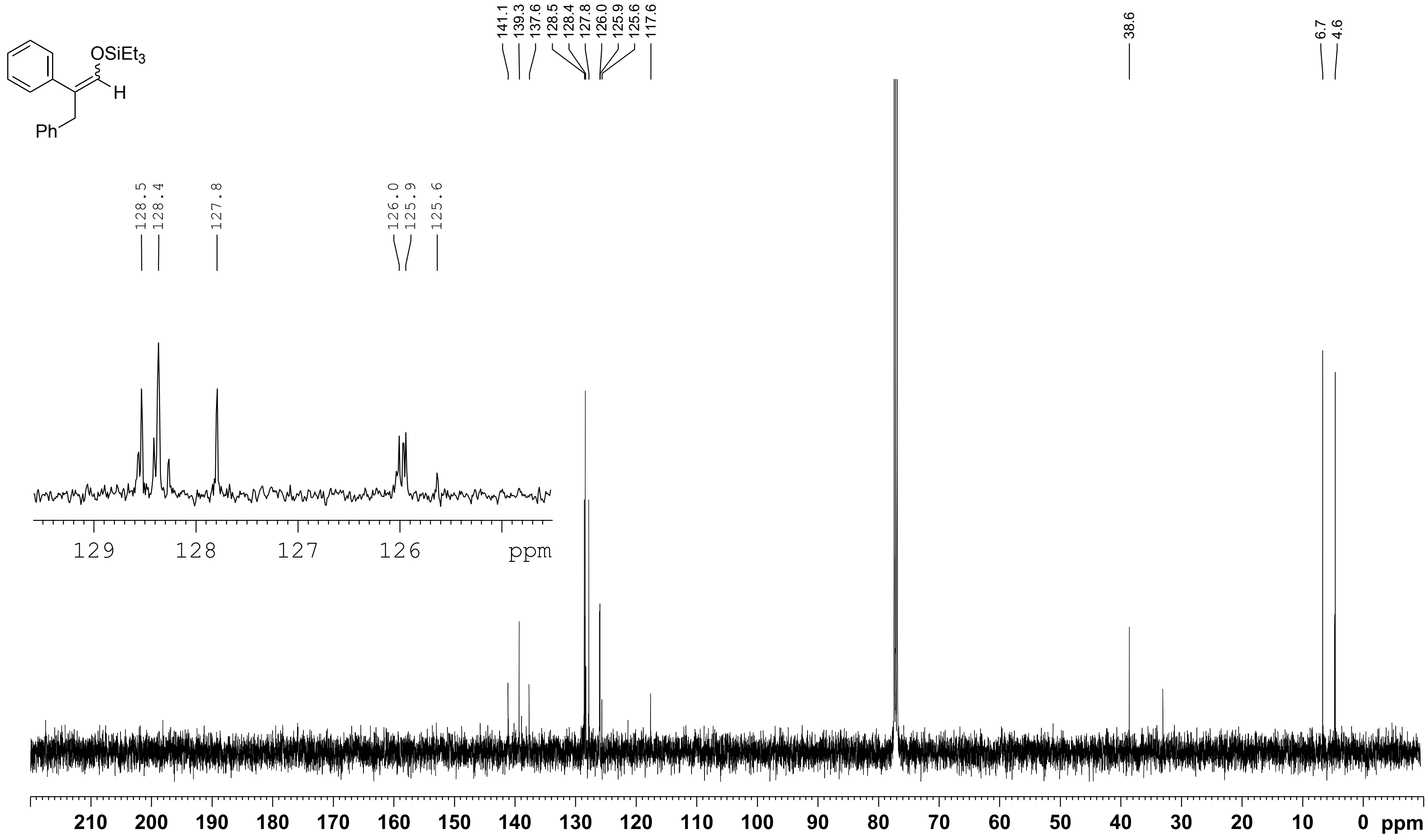

Figure S57. ^{29}Si DEPT NMR spectrum (99 MHz, $CDCl_3$, 298 K, optimized for J = 7 Hz) of **3ga** (Z:E = 72:28).

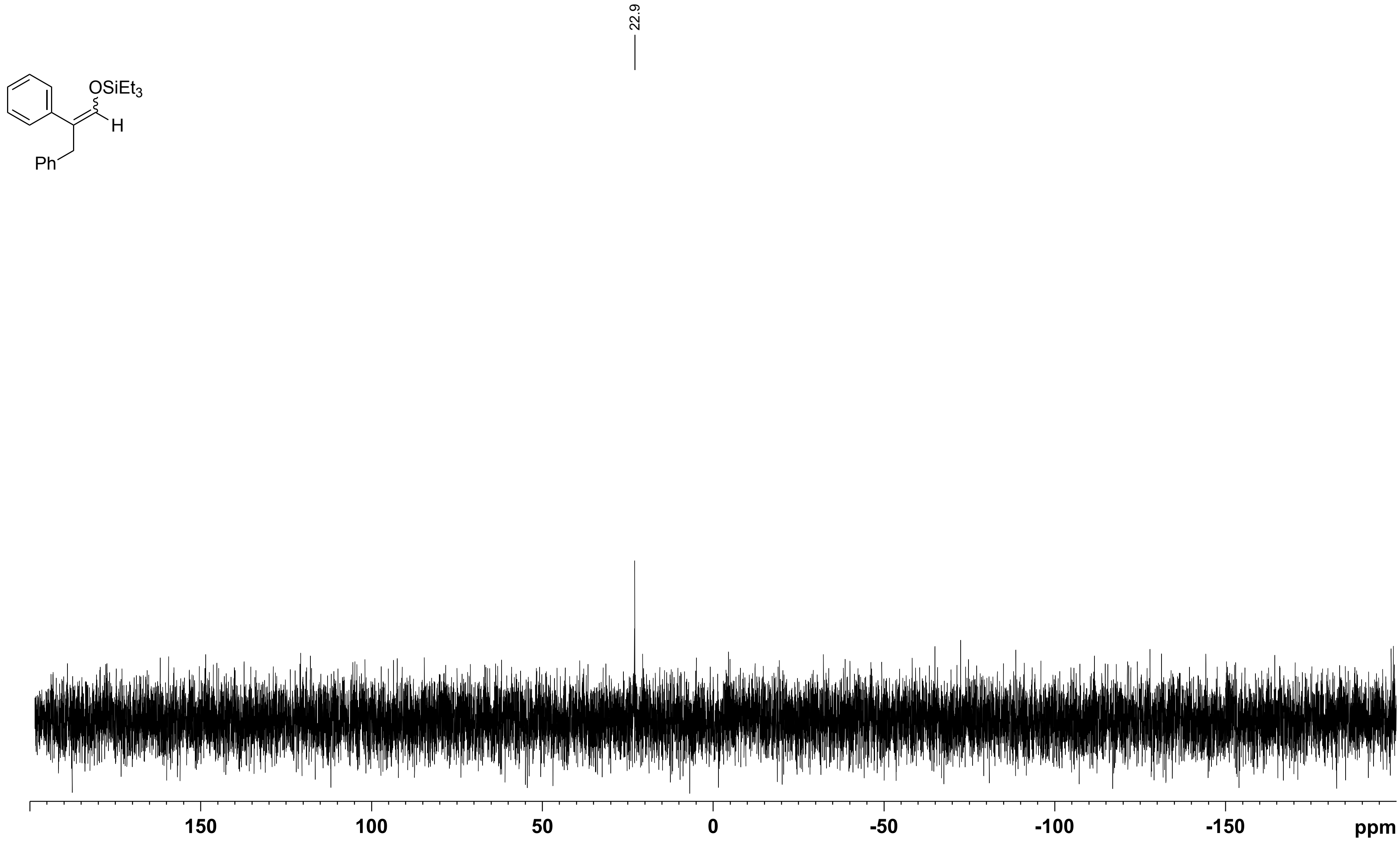

Figure S58. 2D-NOESY NMR spectrum (126 MHz, $CDCl_3$, 298 K) of **3ga** (*Z*:*E* = 72:28).

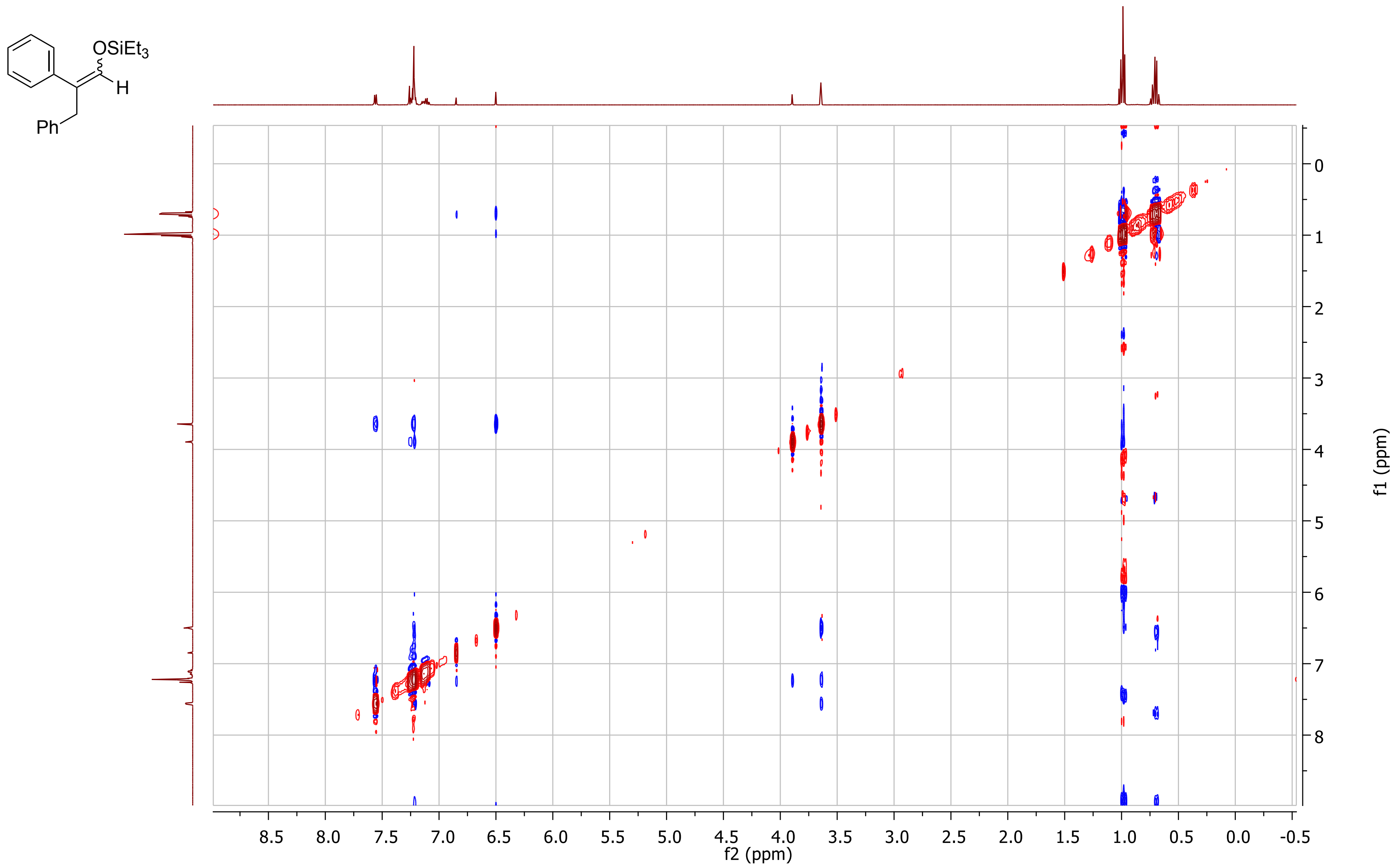

Figure S59. ^{1}H NMR spectrum (500 MHz, $CDCl_3$, 298 K) of **3ha** (*E*:*Z* = 93:7).

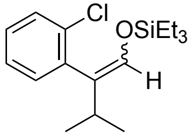

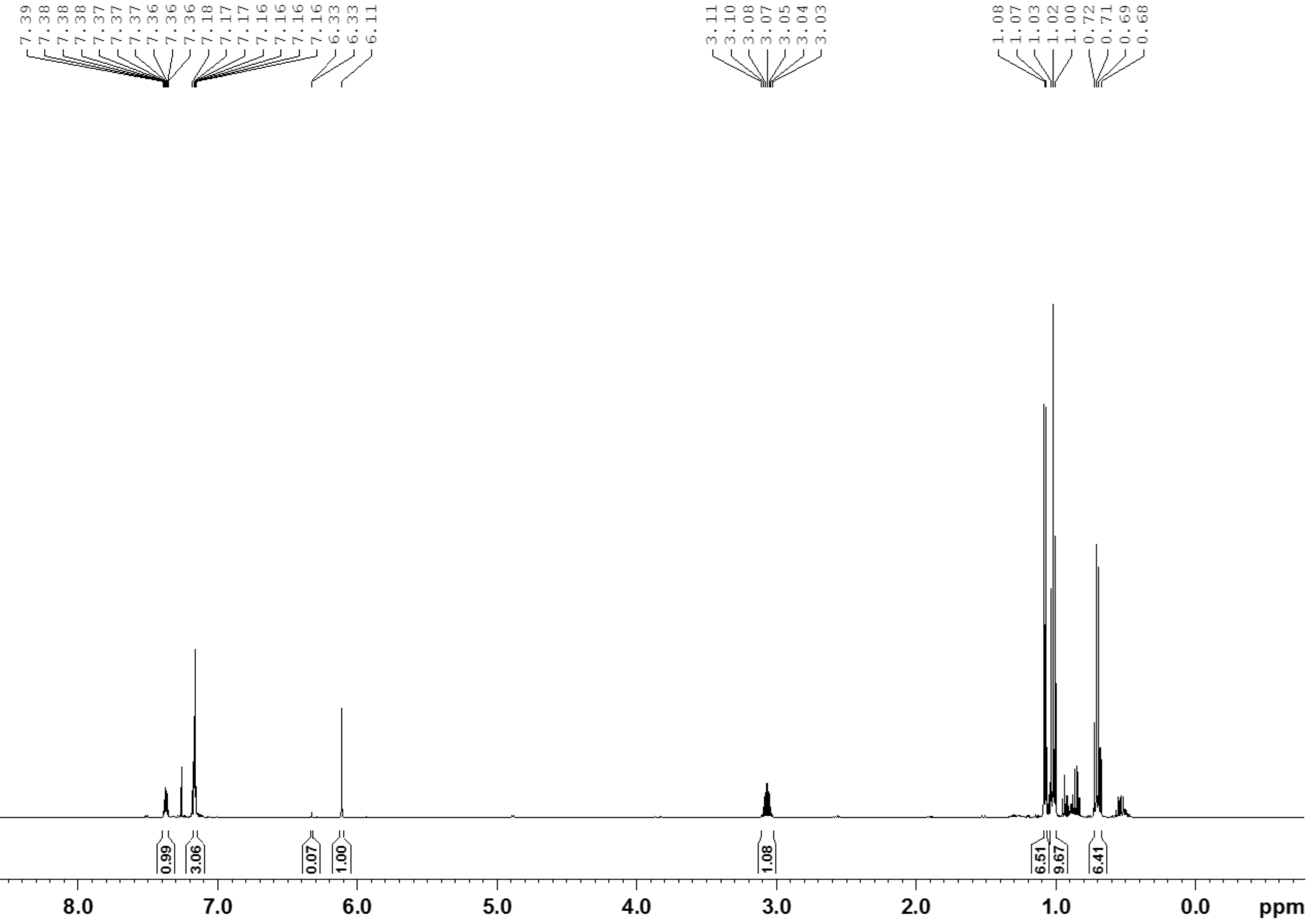

Figure S60. $^{13}C\{^{1}H\}$ NMR spectrum (126 MHz, $CDCl_3$, 298 K) of **3ha** (*E*:*Z* = 93:7).

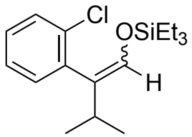

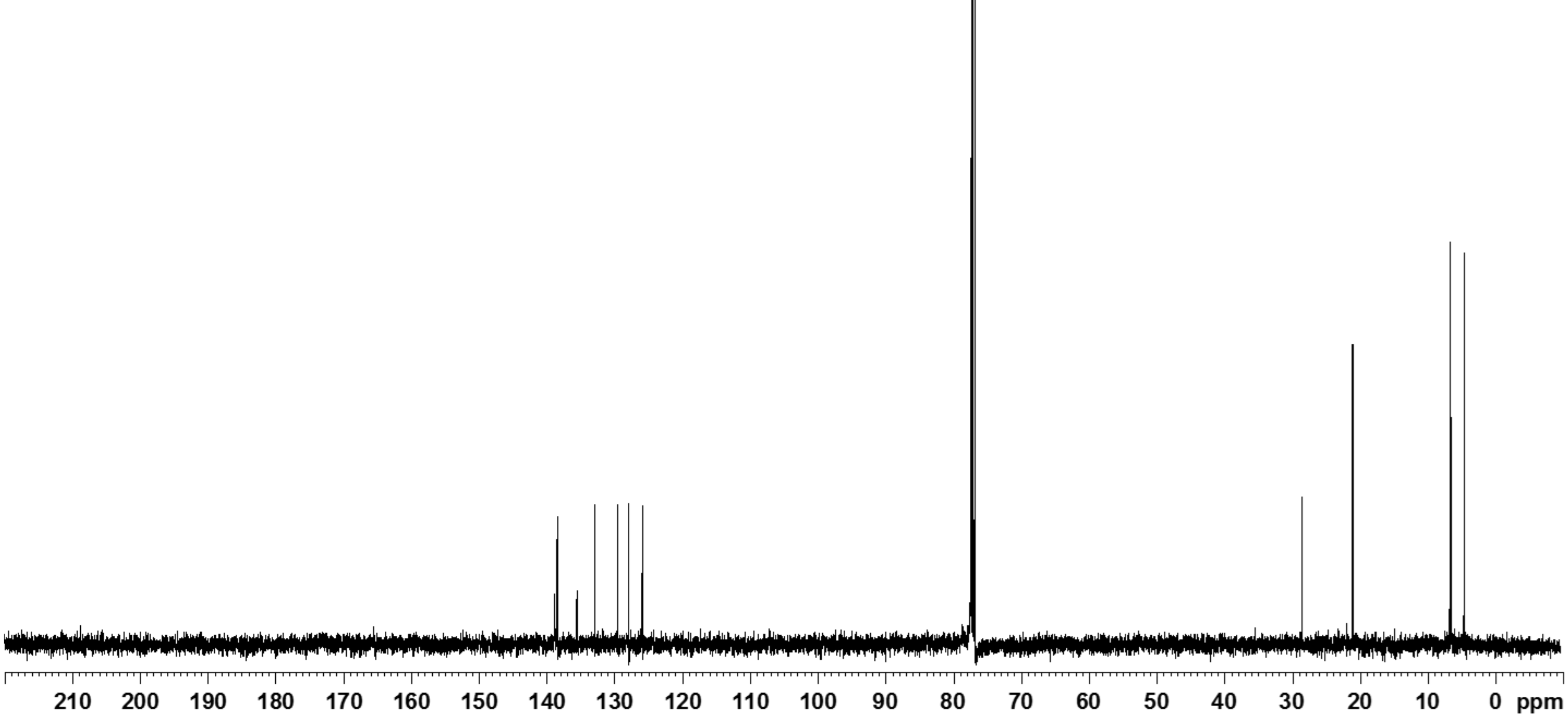

Figure S61. ^{29}Si DEPT NMR spectrum (99 MHz, $CDCl_3$, 298 K, optimized for *J* = 7 Hz) of **3ha** (*E*:*Z* = 93:7).

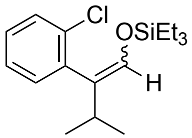

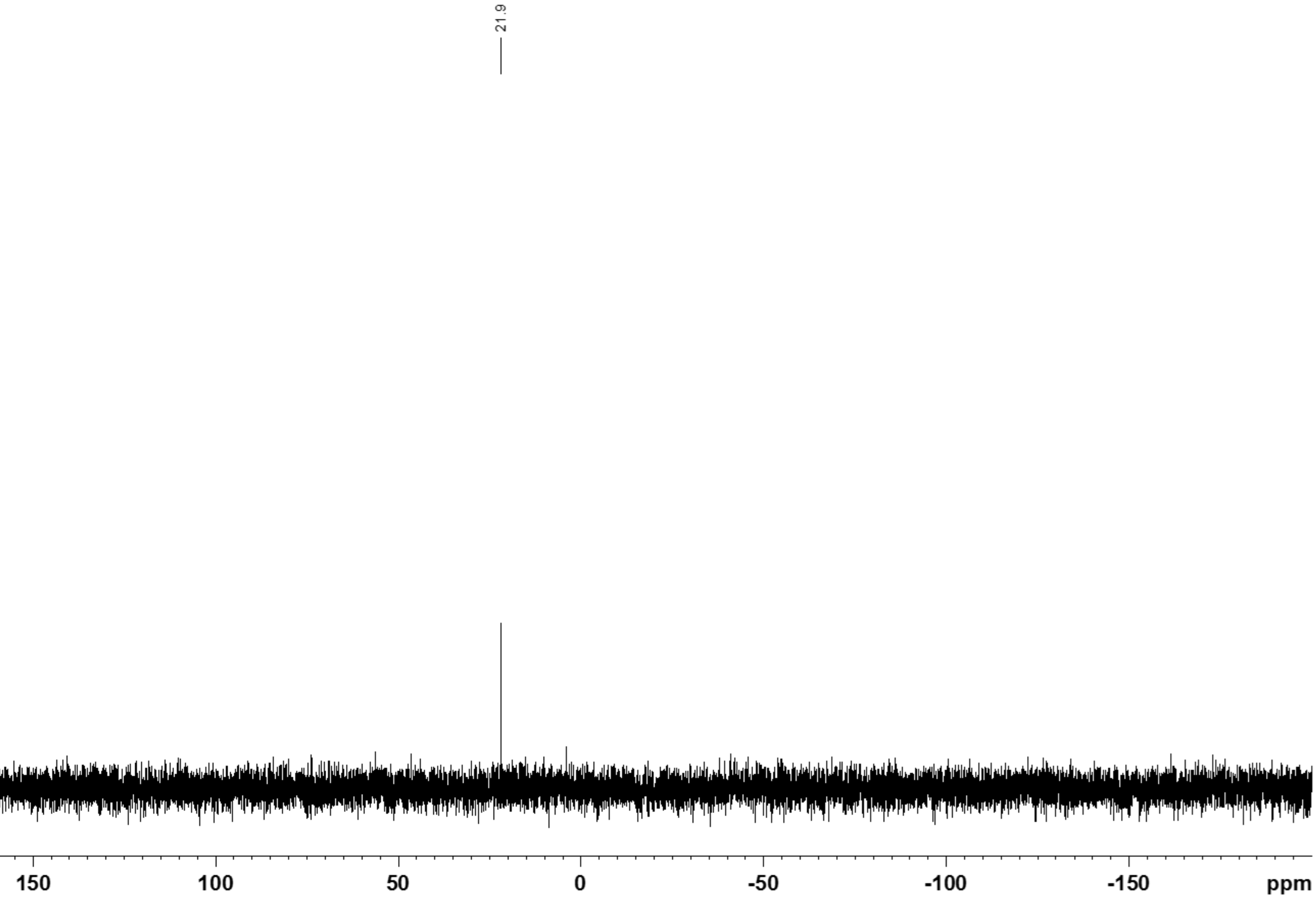

Figure S62. ^{1}H NMR spectrum (500 MHz, $CDCl_3$, 298 K) of **3ia** (*Z*:*E* = 77:23).

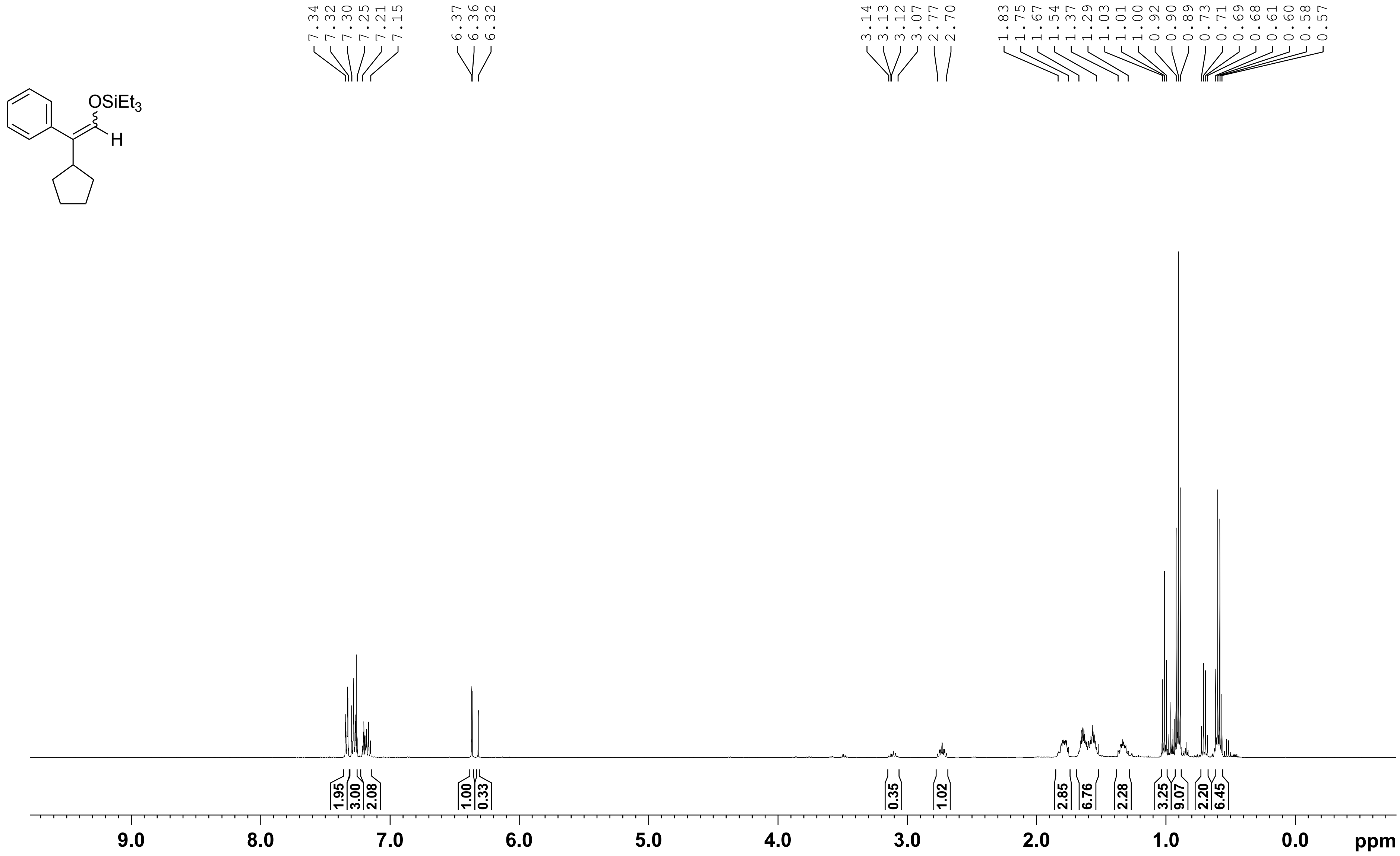

Figure S63. $^{13}C\{^1H\}$ NMR spectrum (126 MHz, $CDCl_3$, 298 K) of **3ia** (*Z*:*E* = 77:23).

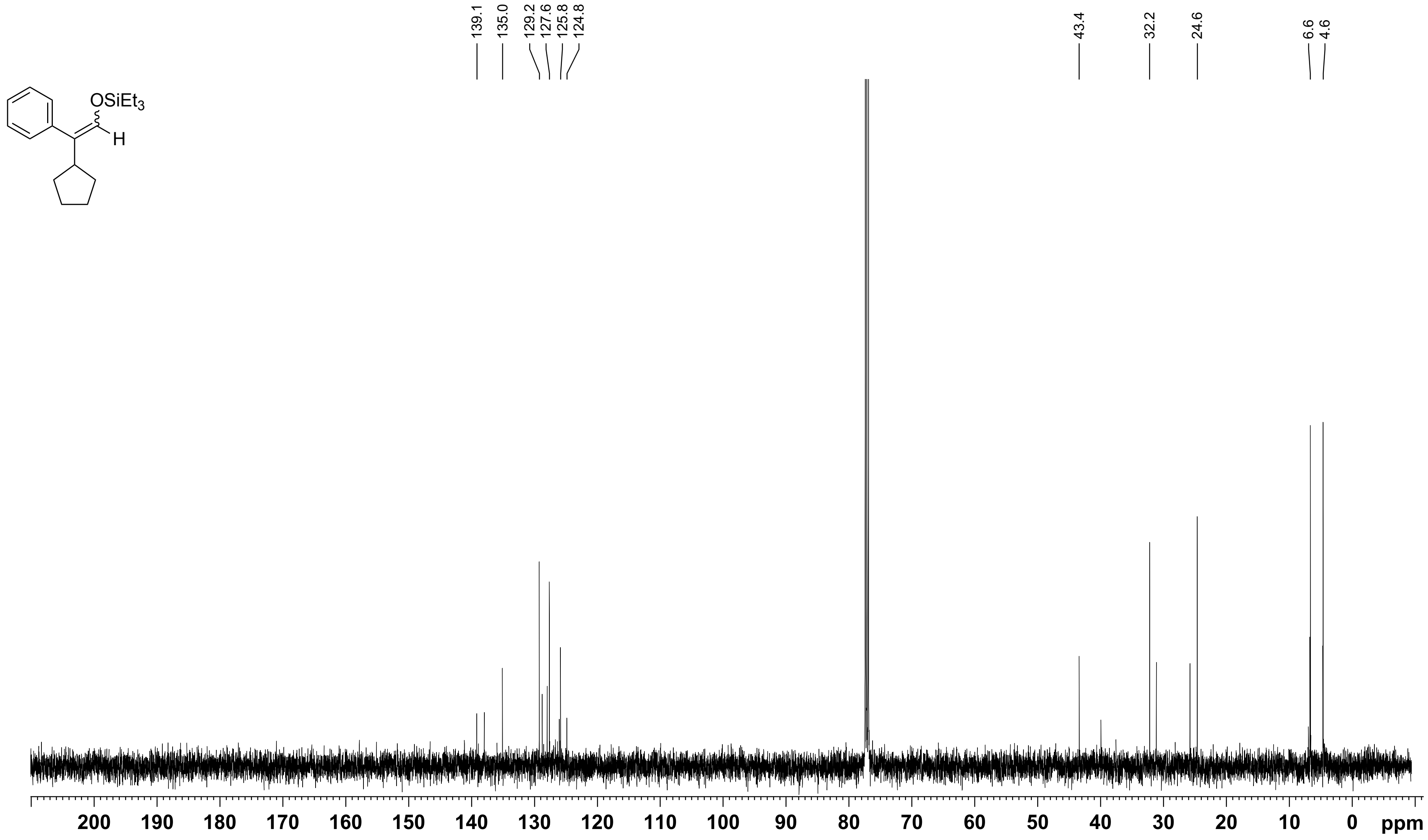

Figure S64. ^{29}Si DEPT NMR spectrum (99 MHz, $CDCl_3$, 298 K, optimized for *J* = 7 Hz) of **3ia** (*Z*:*E* = 77:23).

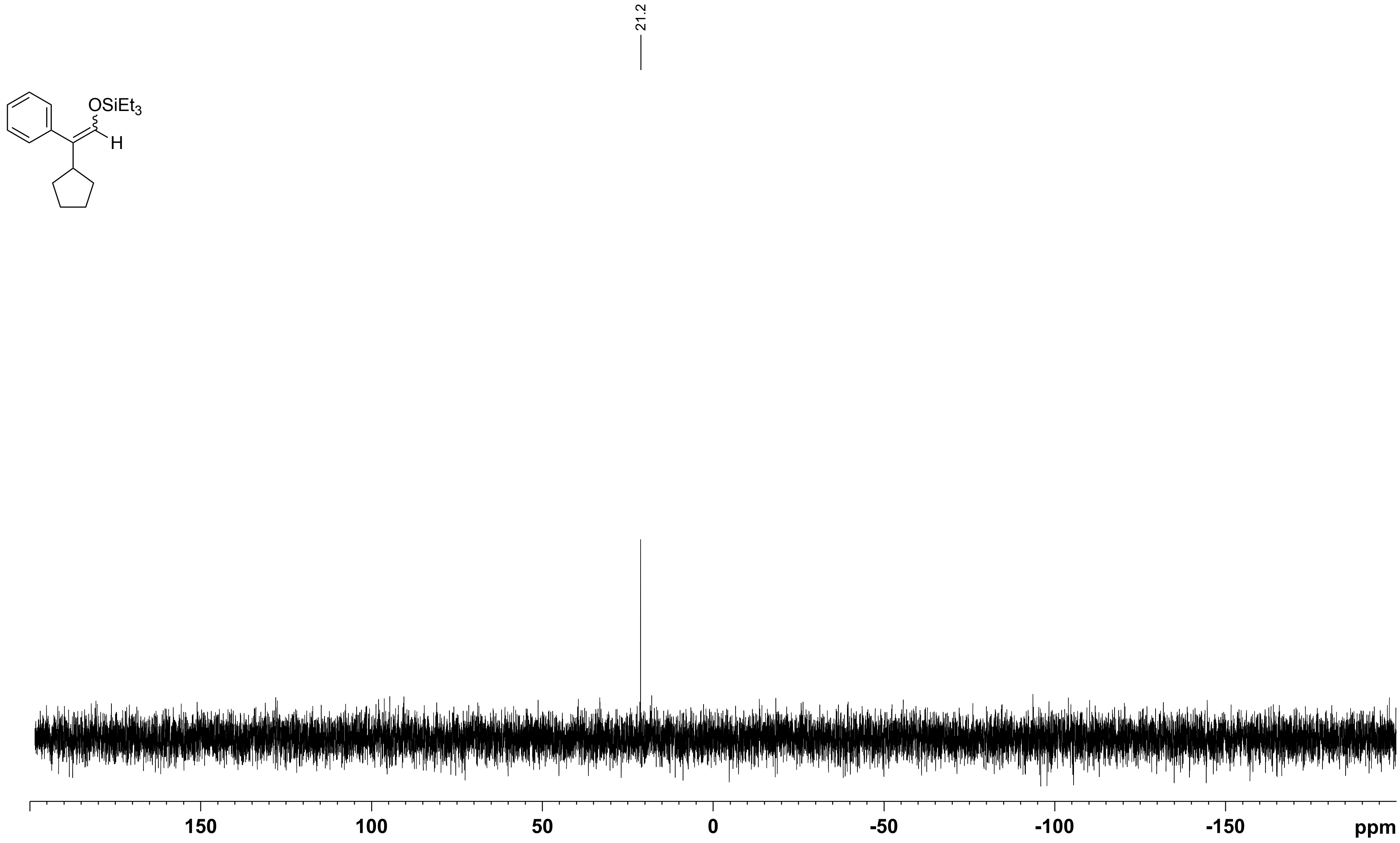

Figure S65. ^{1}H NMR spectrum (500 MHz, $CDCl_3$, 298 K) of **3ja** (*Z*:*E* = 89:11).

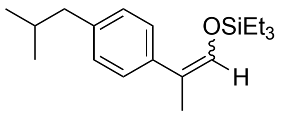

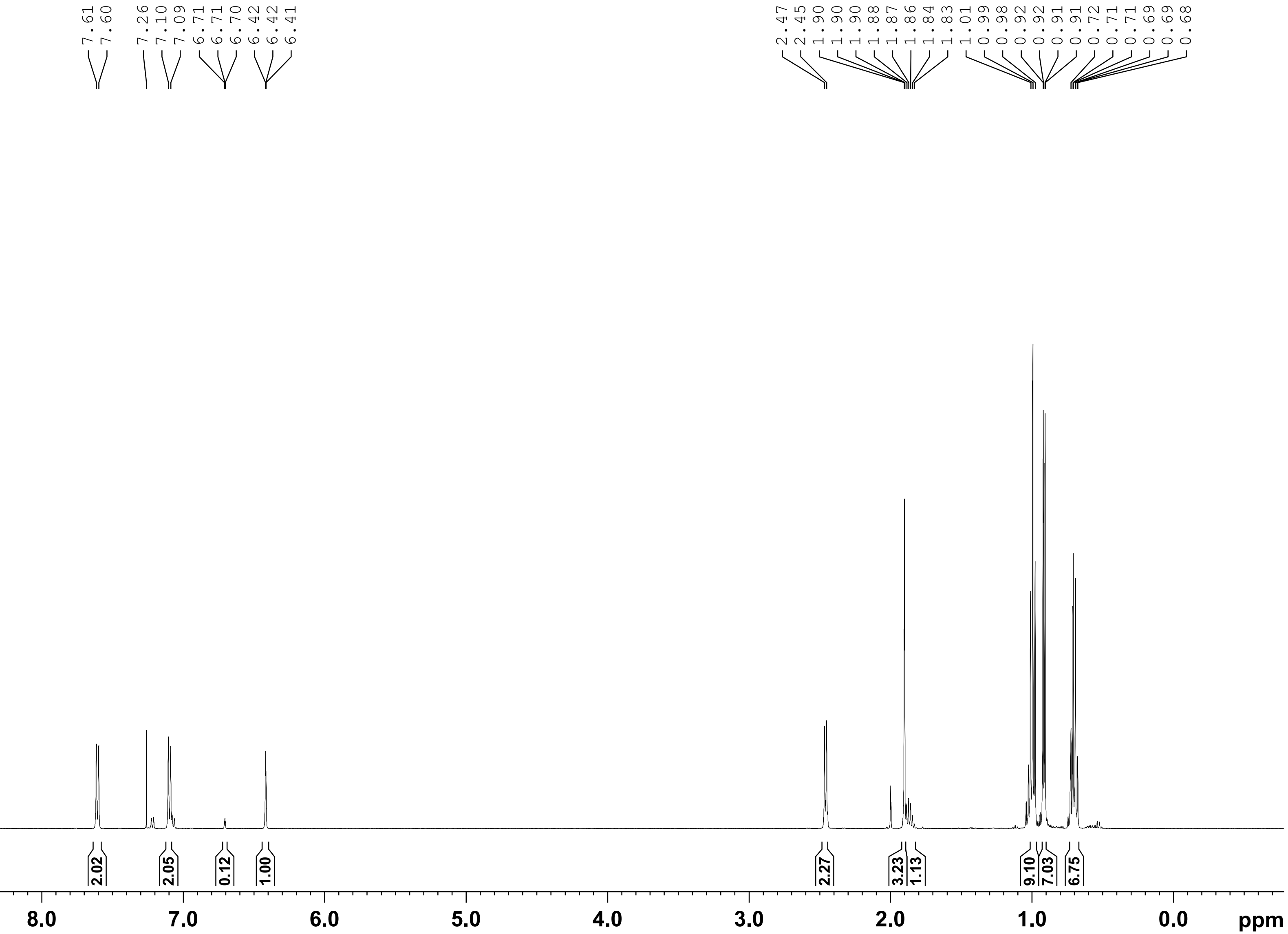

Figure S66. $^{13}C\{^{1}H\}$ NMR spectrum (126 MHz, $CDCl_3$, 298 K) of **3ja** (*Z*:*E* = 89:11).

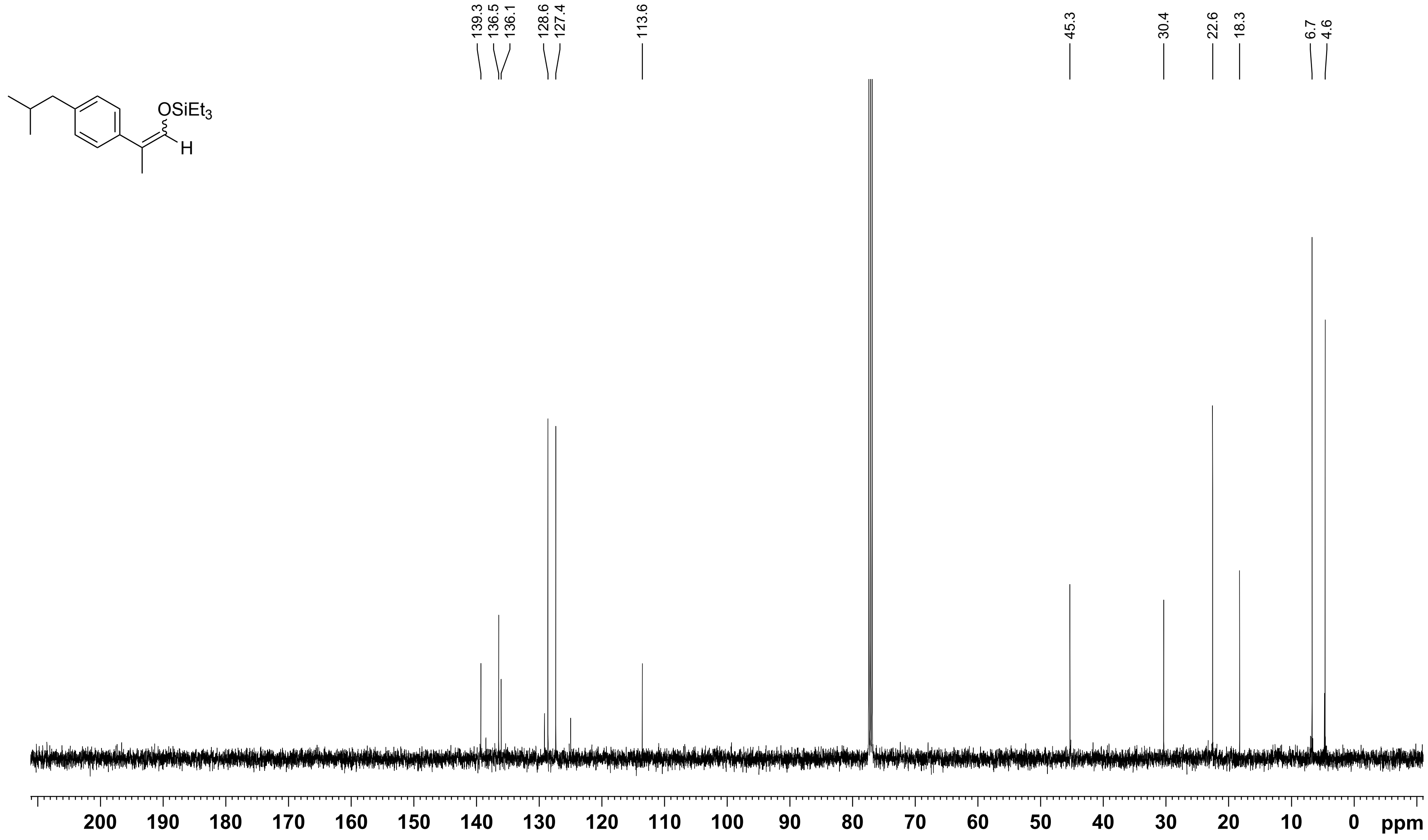

Figure S67. ^{29}Si DEPT NMR spectrum (99 MHz, $CDCl_3$, 298 K, optimized for J = 7 Hz) of **3ja** (Z:E = 89:11).

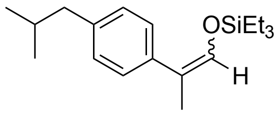

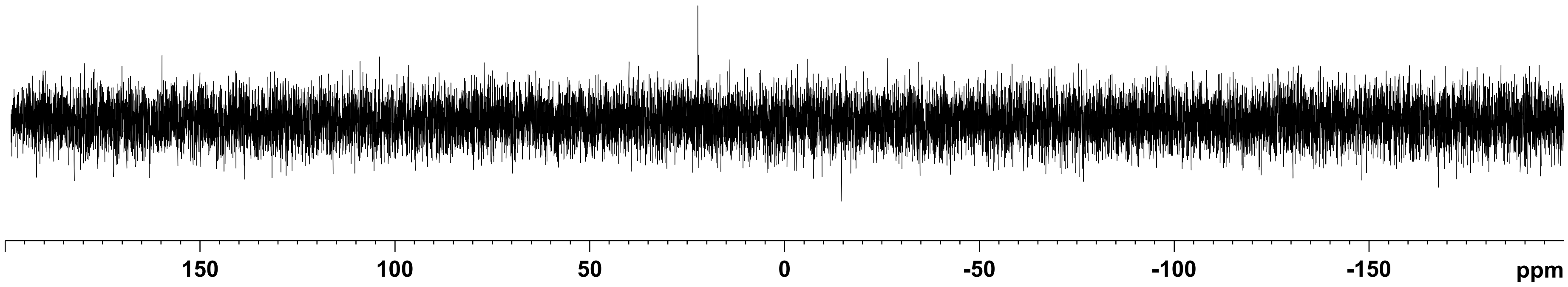

Figure S68. ^{1}H NMR spectrum (500 MHz, $CDCl_3$, 298 K) of **3ka** (*Z*:*E* = 77:23).

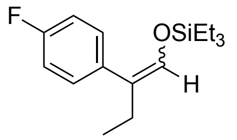

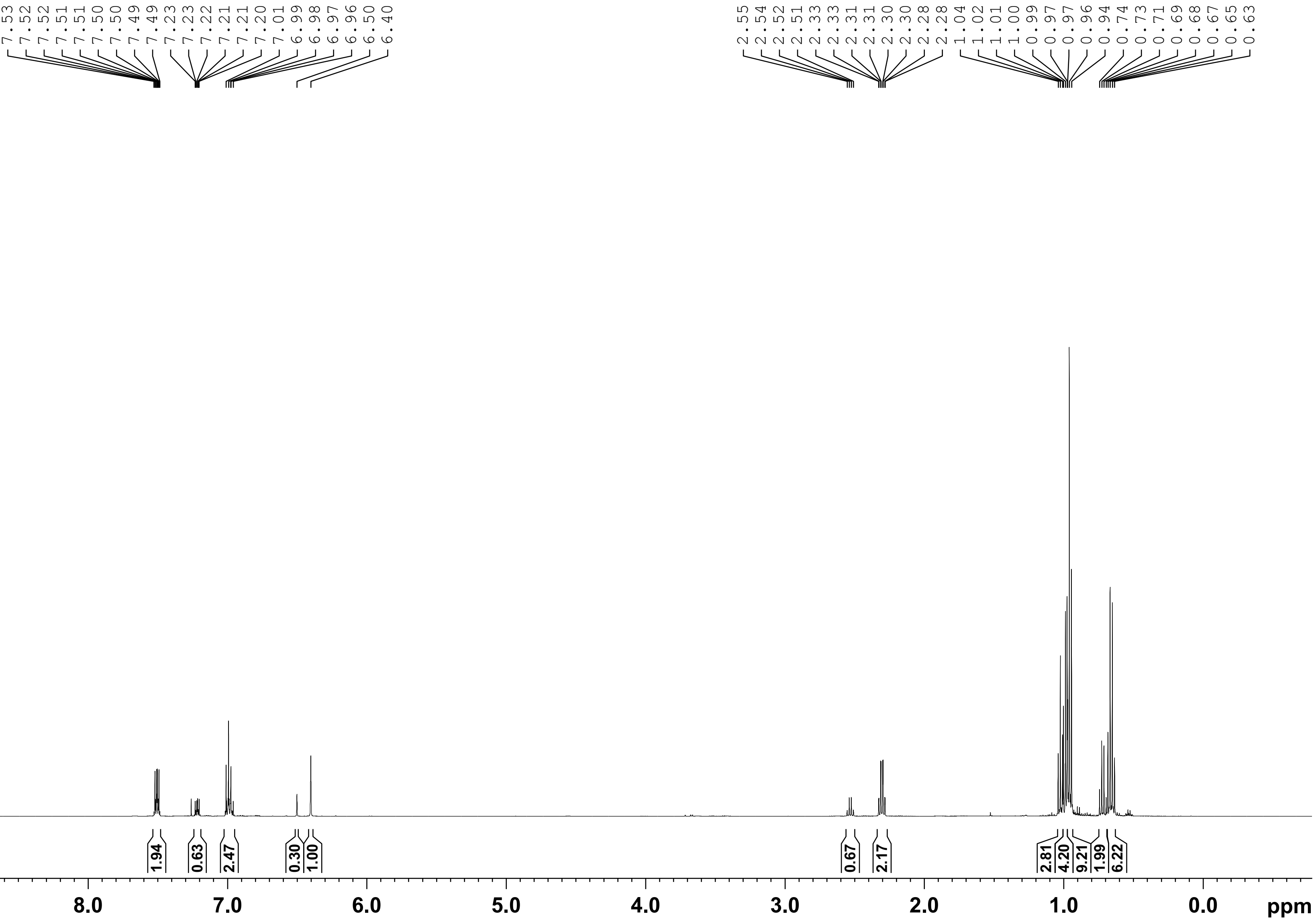

Figure S69. $^{13}C\{^{1}H\}$ NMR spectrum (126 MHz, $CDCl_3$, 298 K) of **3ka** (*Z*:*E* = 77:23).

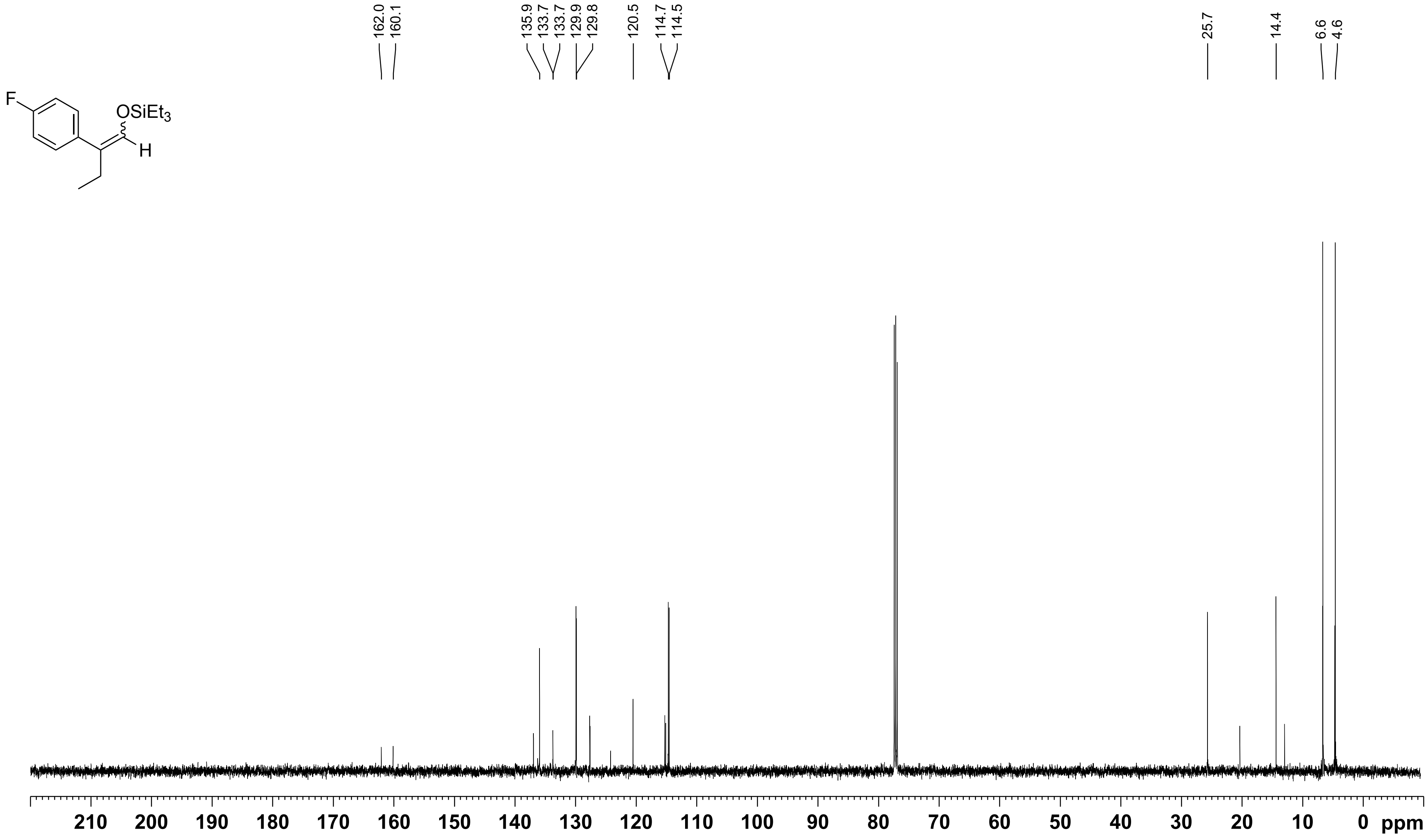

Figure S70. $^{19}F\{^{1}H\}$ NMR spectrum (126 MHz, $CDCl_3$, 298 K) of **3ka** (*Z*:*E* = 77:23).

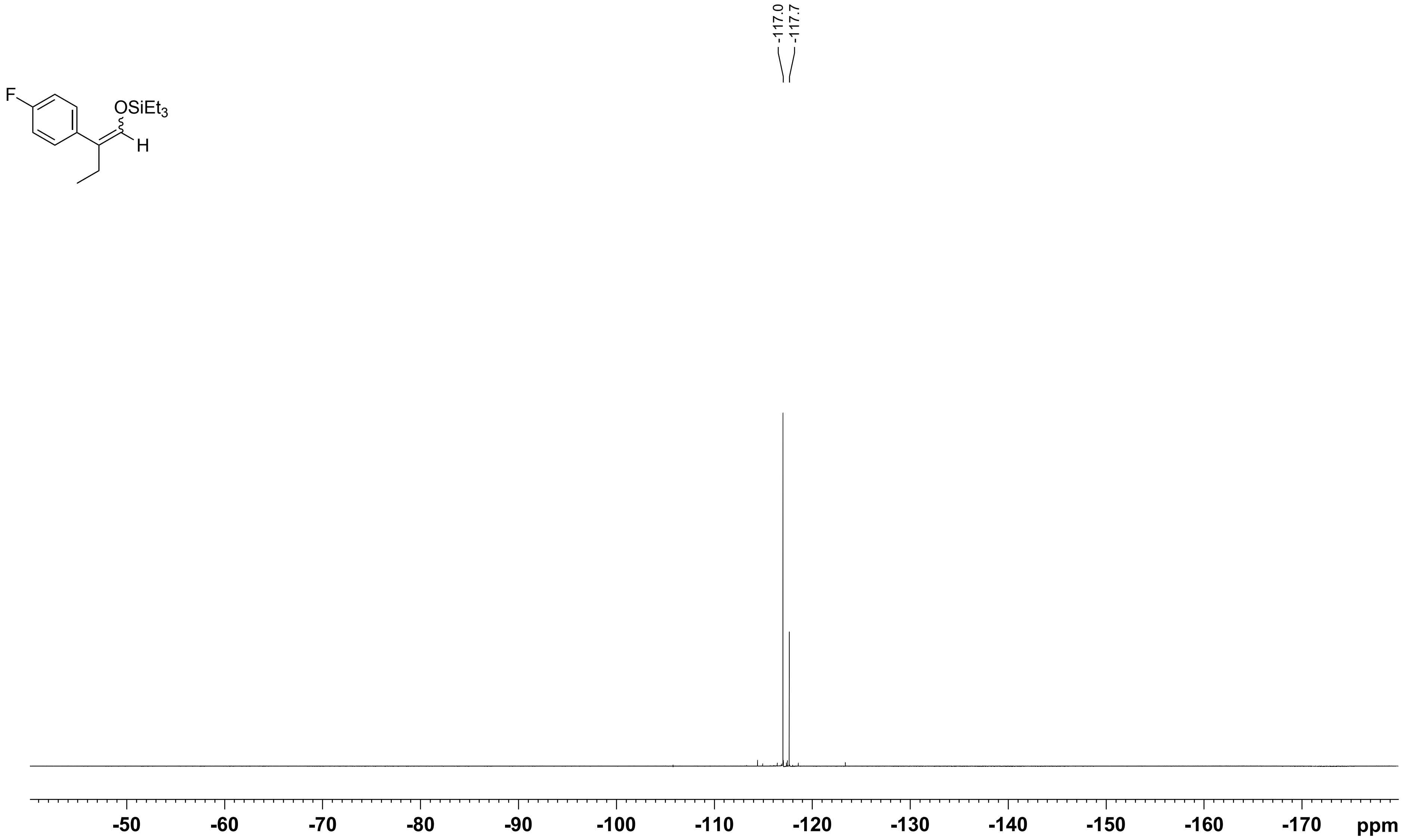

Figure S71. ^{29}Si DEPT NMR spectrum (99 MHz, $CDCl_3$, 298 K, optimized for *J* = 7 Hz) of **3ka** (*Z*:*E* = 77:23).

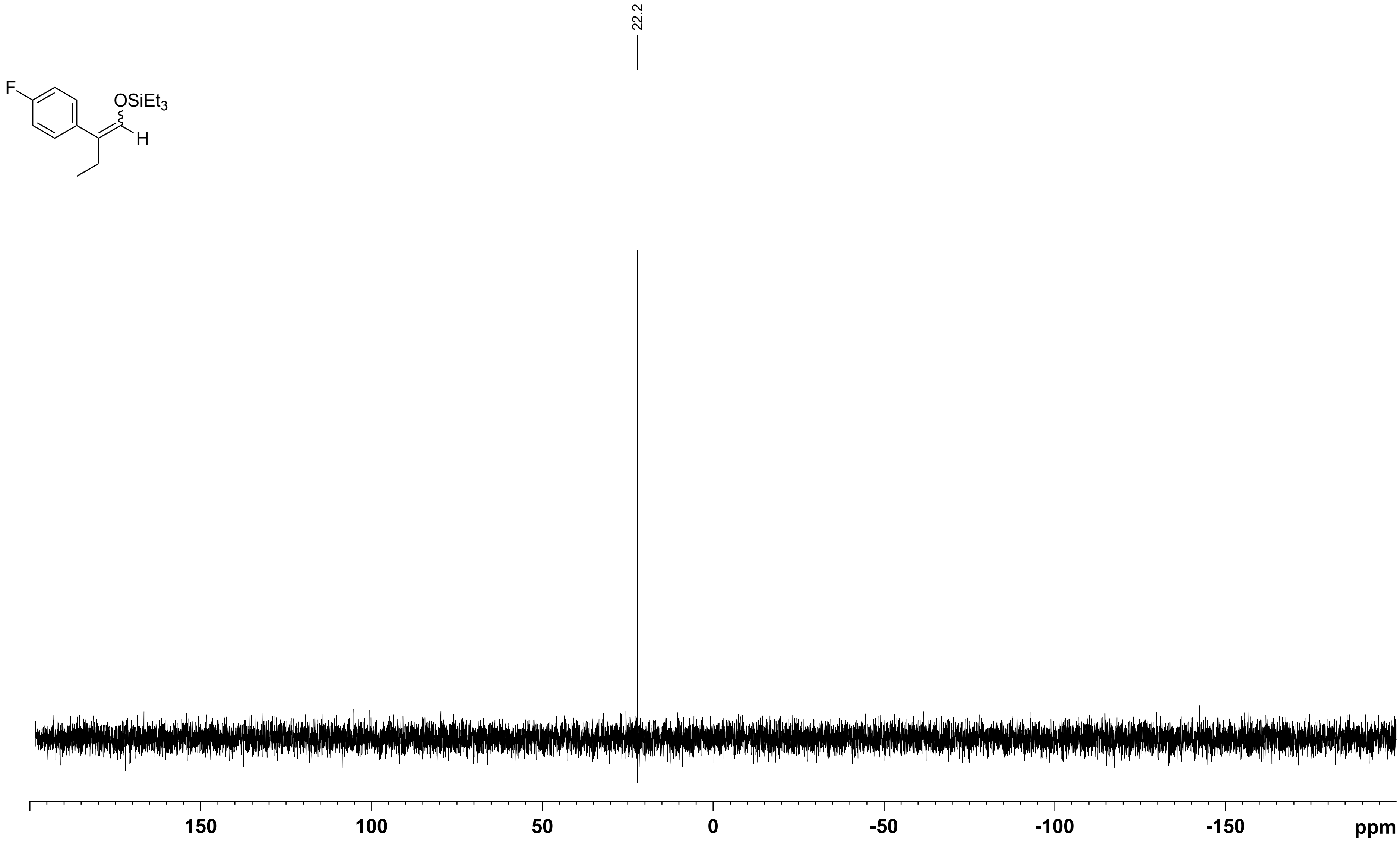

Figure S72. ^{1}H NMR spectrum (500 MHz, $CDCl_3$, 298 K) of **3la** (*Z*:*E* = 75:25).

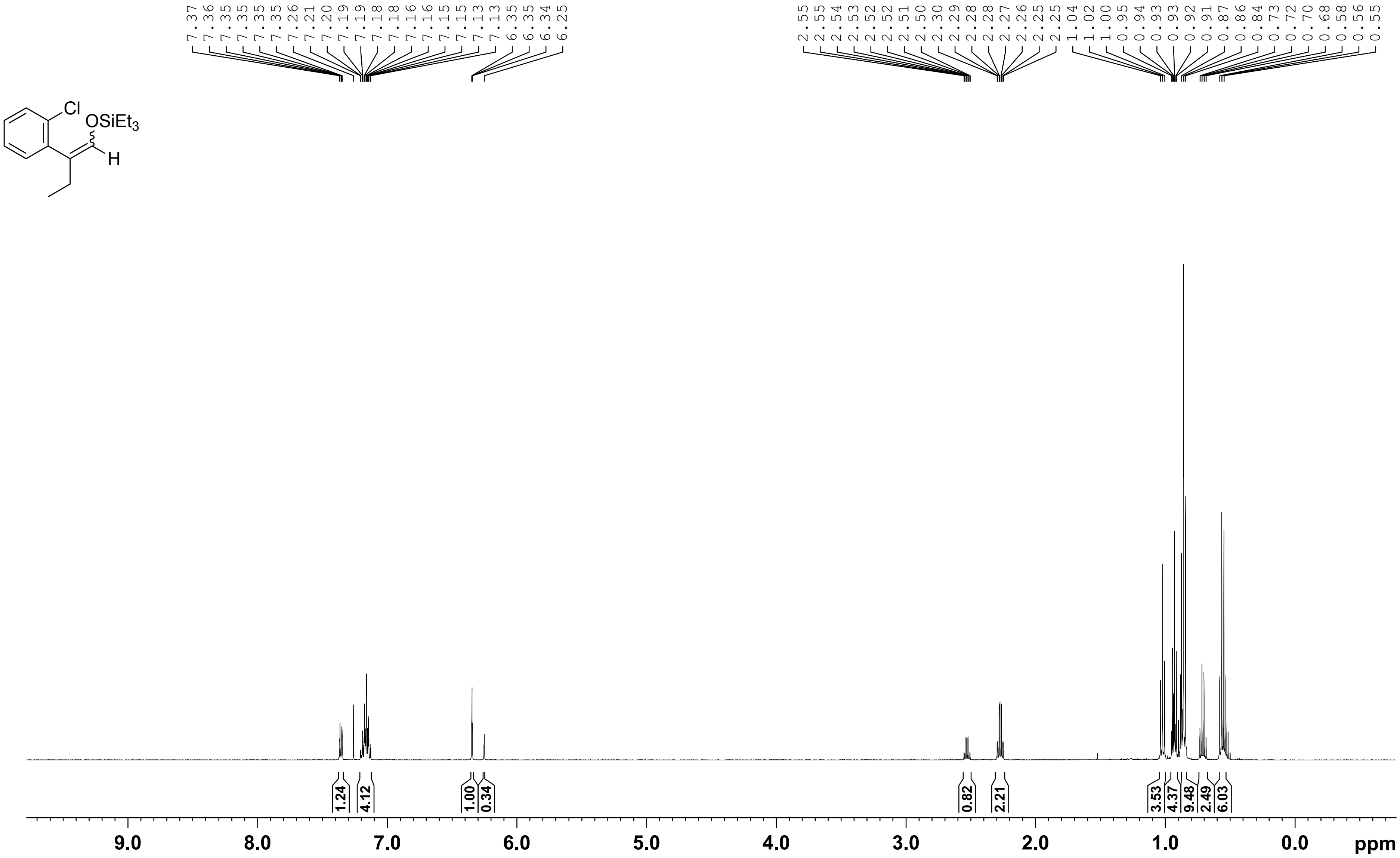

Figure S73. $^{13}C\{^{1}H\}$ NMR spectrum (126 MHz, $CDCl_3$, 298 K) of **3la** (*Z*:*E* = 75:25).

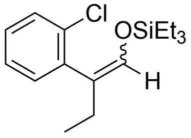

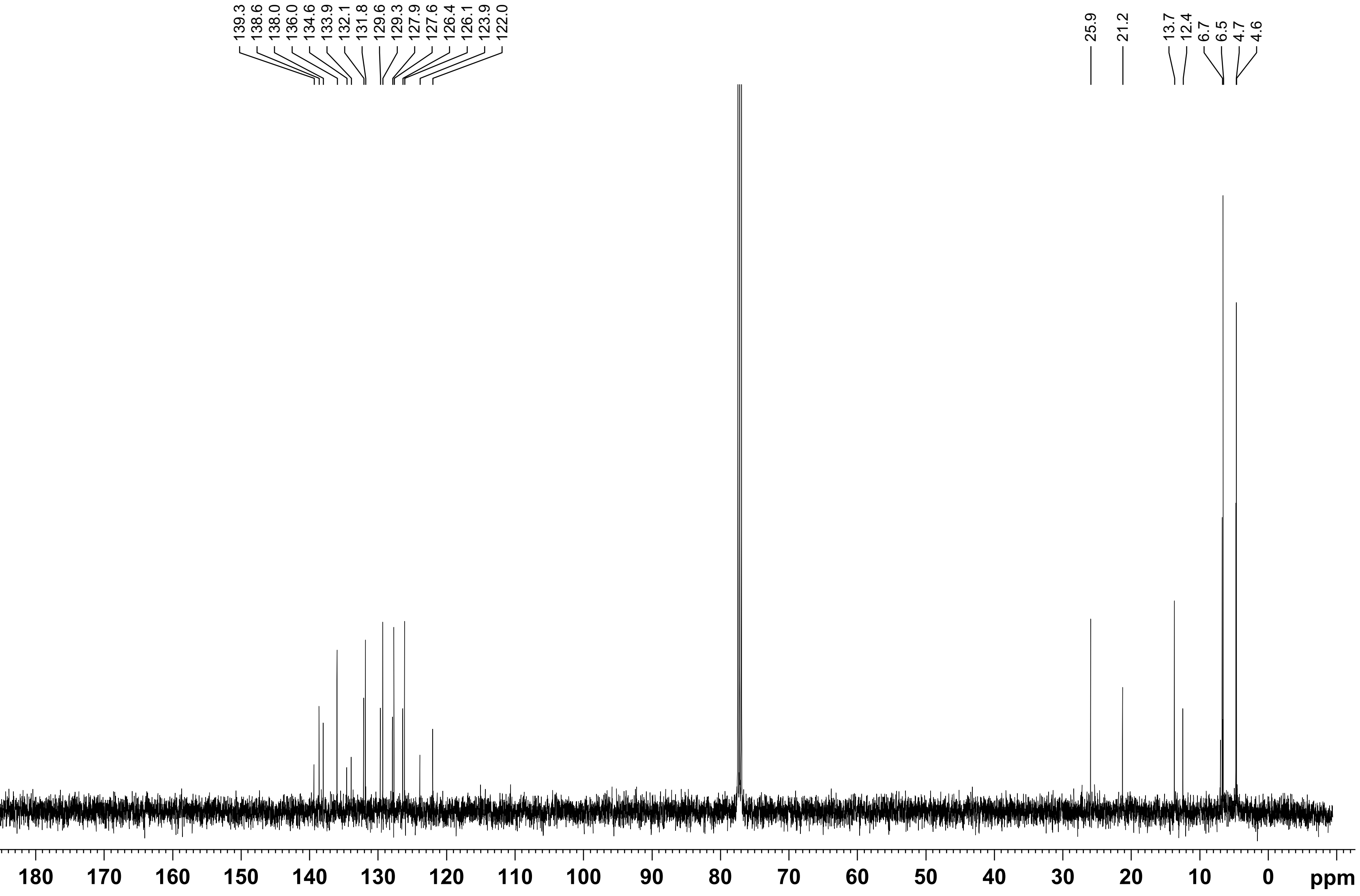

Figure S74. ^{29}Si DEPT NMR spectrum (99 MHz, $CDCl_3$, 298 K, optimized for J = 7 Hz) of **3la** (Z:E = 75:25).

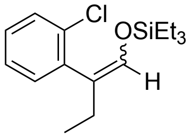

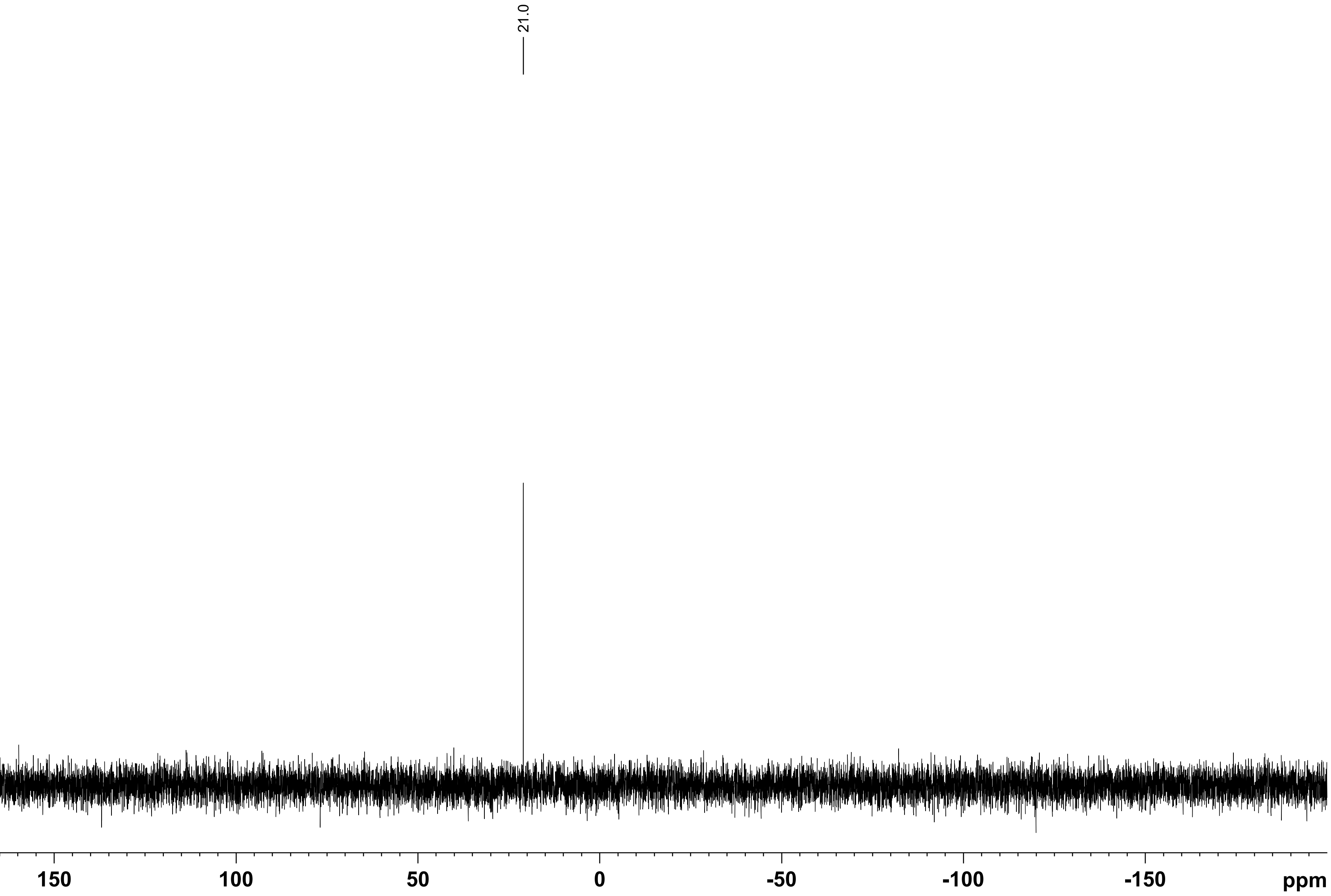

Figure S75. ^{1}H NMR spectrum (500 MHz, $CDCl_3$, 298 K) of **3ma** (*Z*:*E* = 83:17).

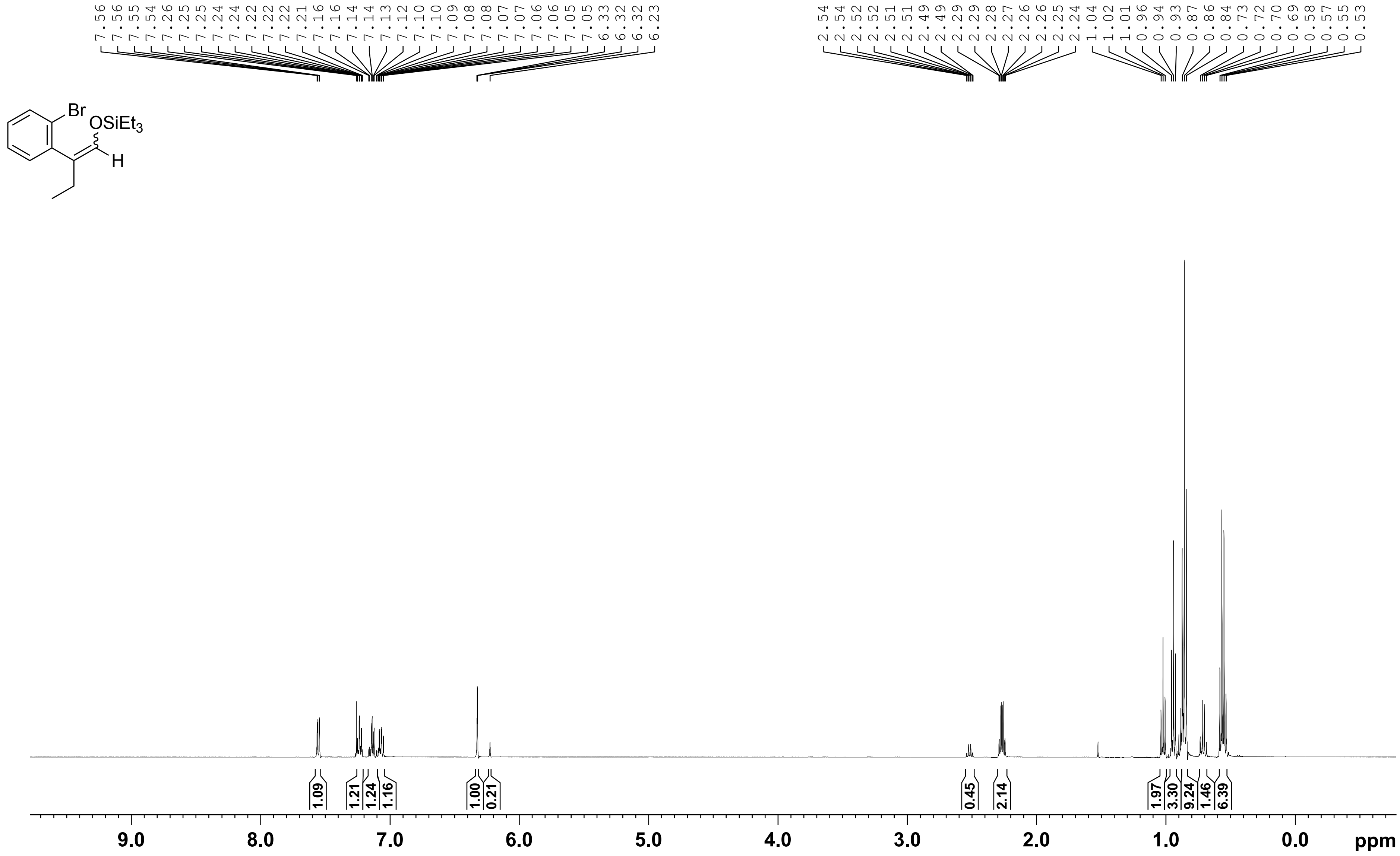

Figure S76. $^{13}C\{^1H\}$ NMR spectrum (126 MHz, $CDCl_3$, 298 K) of **3ma** (*Z*:*E* = 83:17).

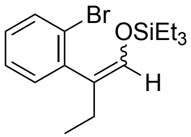

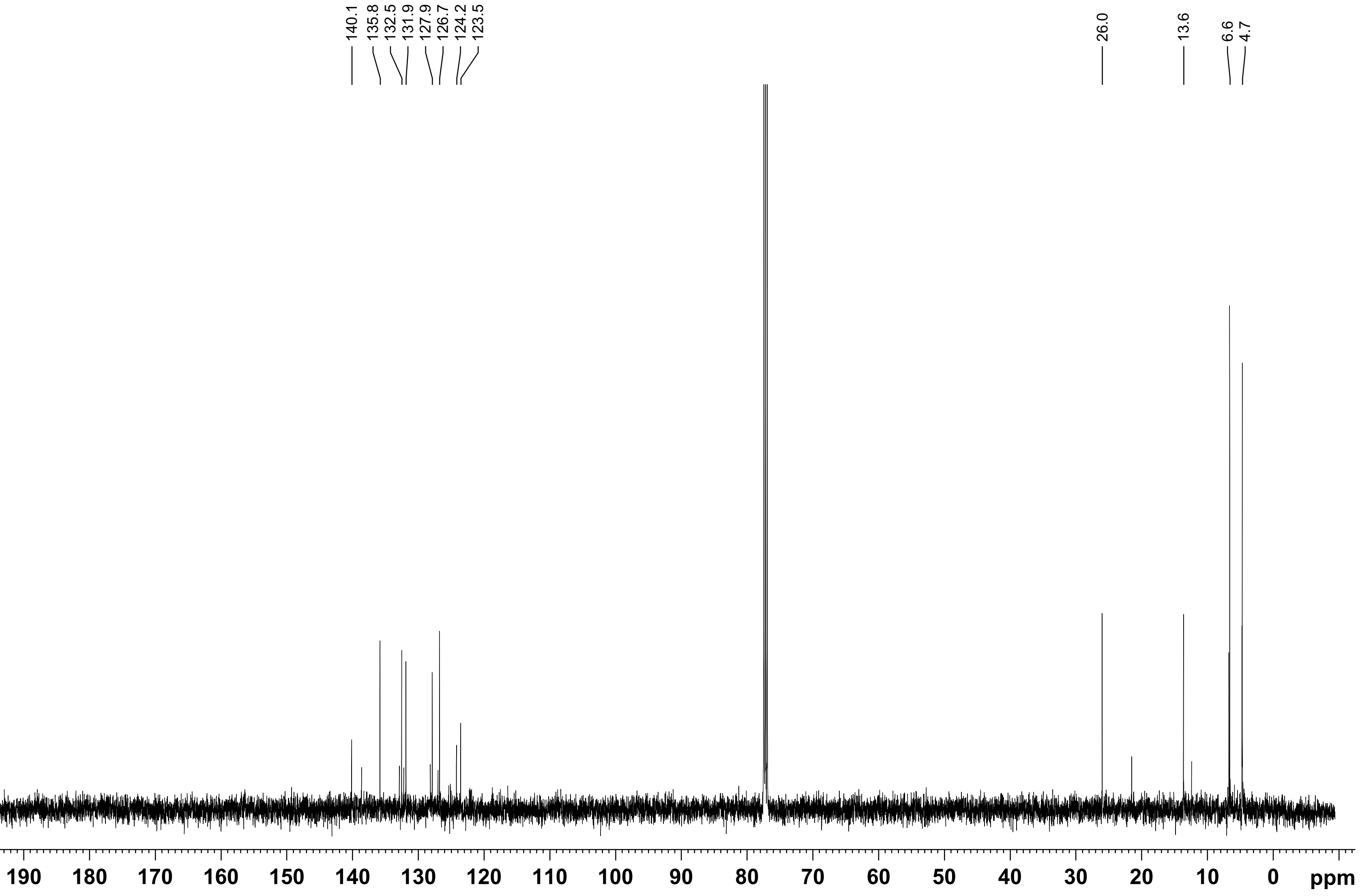

Figure S77. ^{29}Si DEPT NMR spectrum (99 MHz, $CDCl_3$, 298 K, optimized for J = 7 Hz) of **3ma** (Z:E = 83:17).

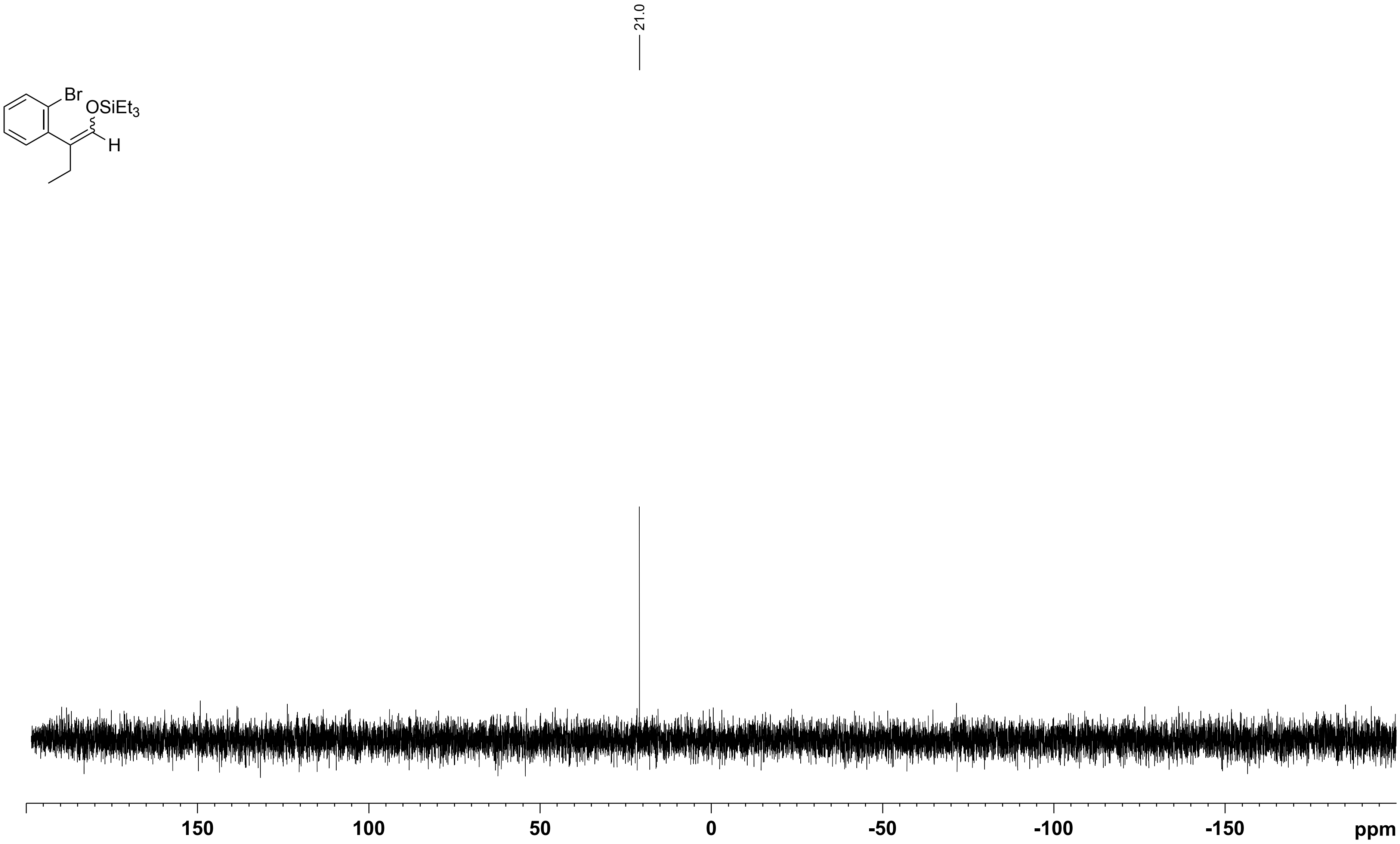

Figure S78. ^{1}H NMR spectrum (500 MHz, $CDCl_3$, 298 K) of **3na** (*Z*:*E* = 91:9).

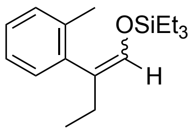

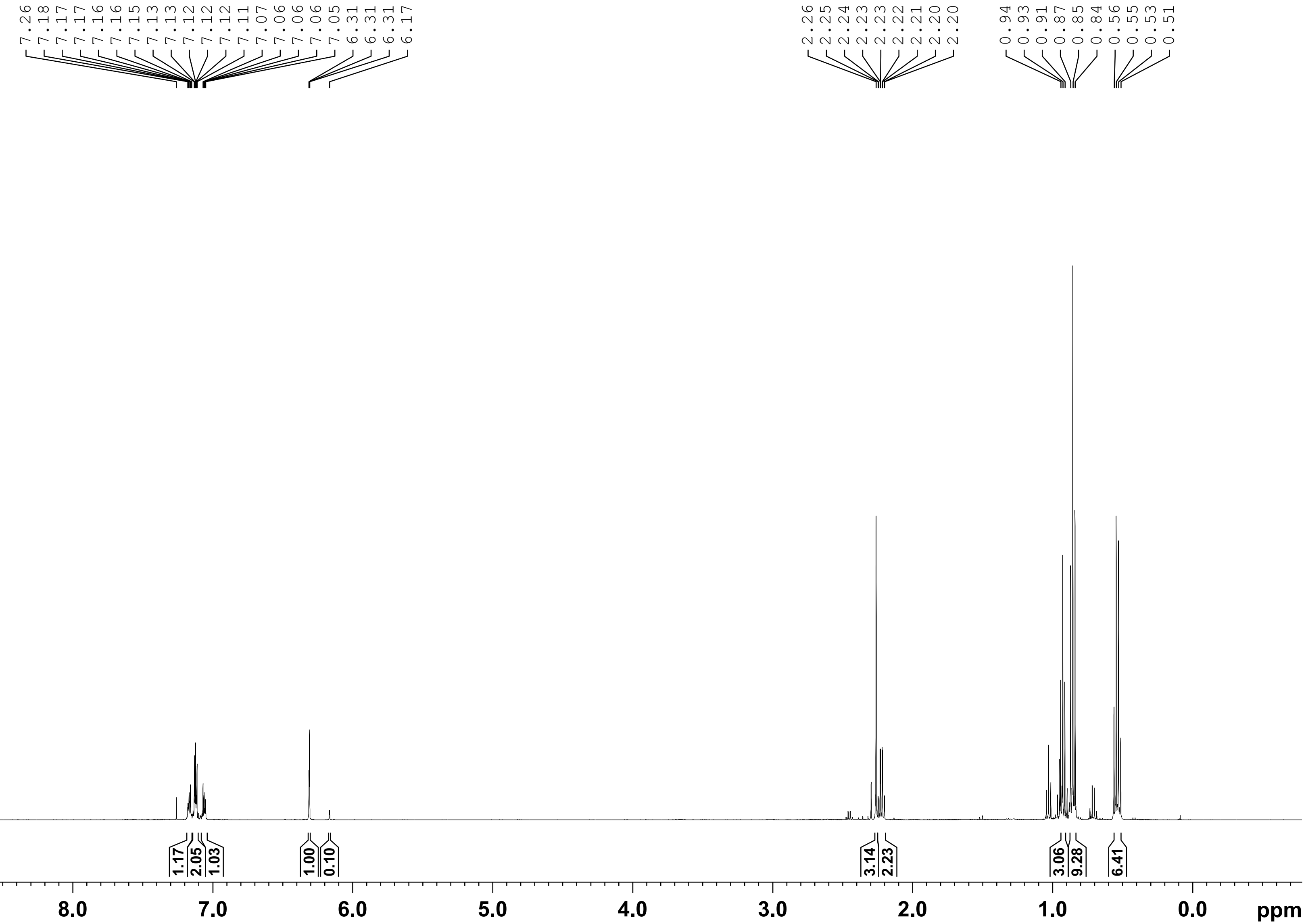

Figure S79. $^{13}C\{^1H\}$ NMR spectrum (126 MHz, $CDCl_3$, 298 K) of **3na** (*Z*:*E* = 91:9).

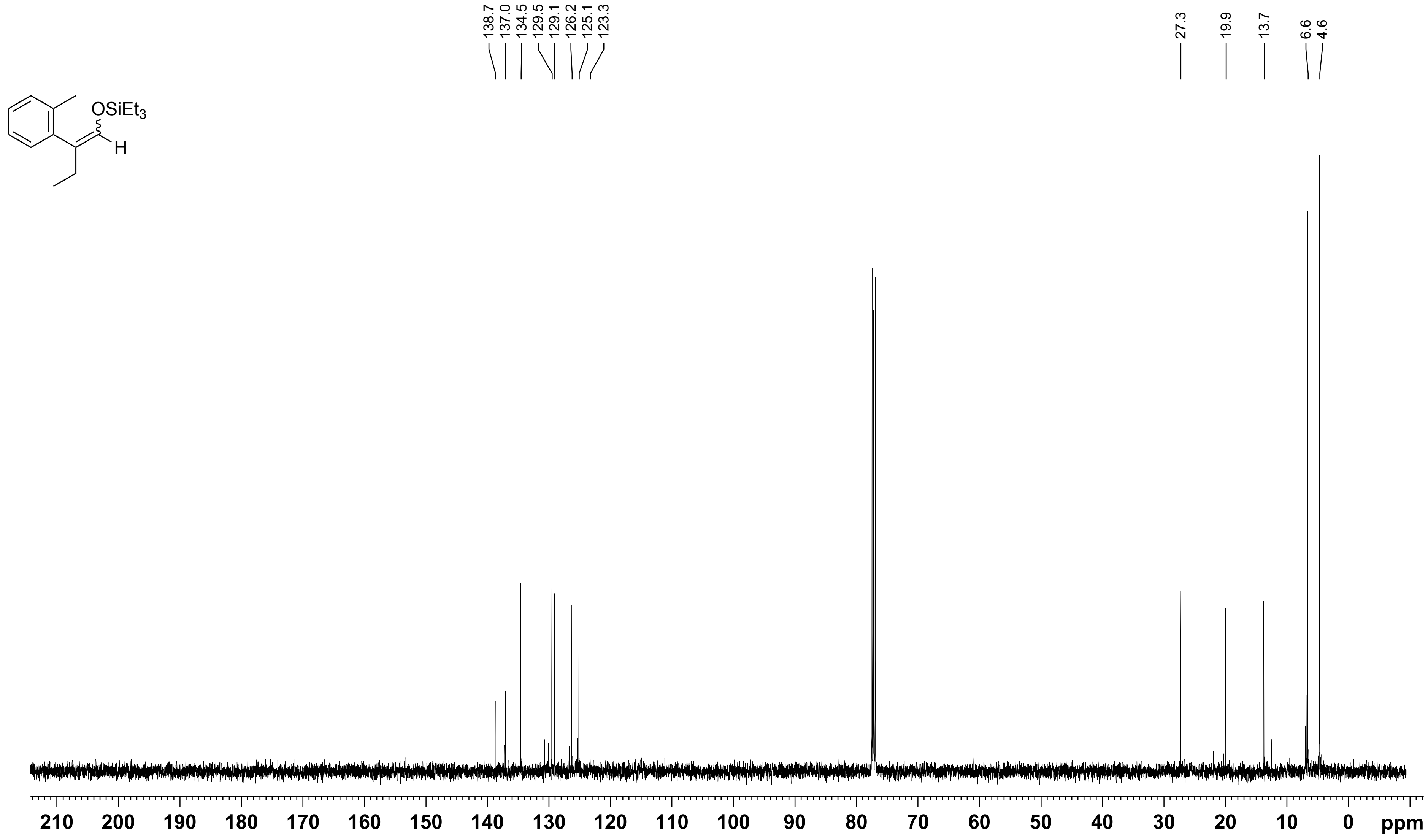

Figure S80. ^{29}Si DEPT NMR spectrum (99 MHz, $CDCl_3$, 298 K, optimized for *J* = 7 Hz) of **3na** (*Z*:*E* = 91:9).

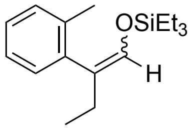

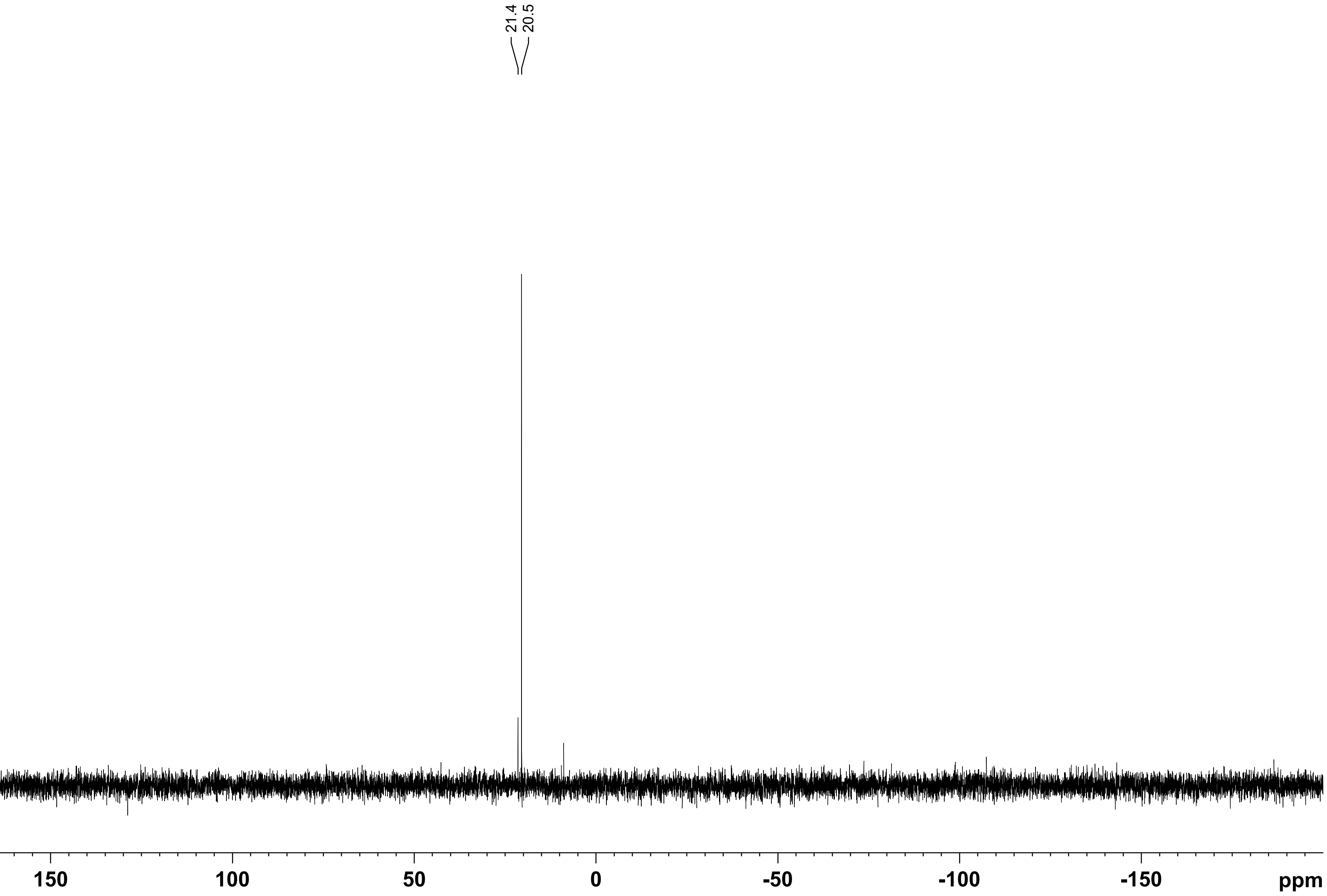

Figure S81. ^{1}H NMR spectrum (500 MHz, $CDCl_3$, 298 K) of **3oa** (*Z*:*E* = 67:33).

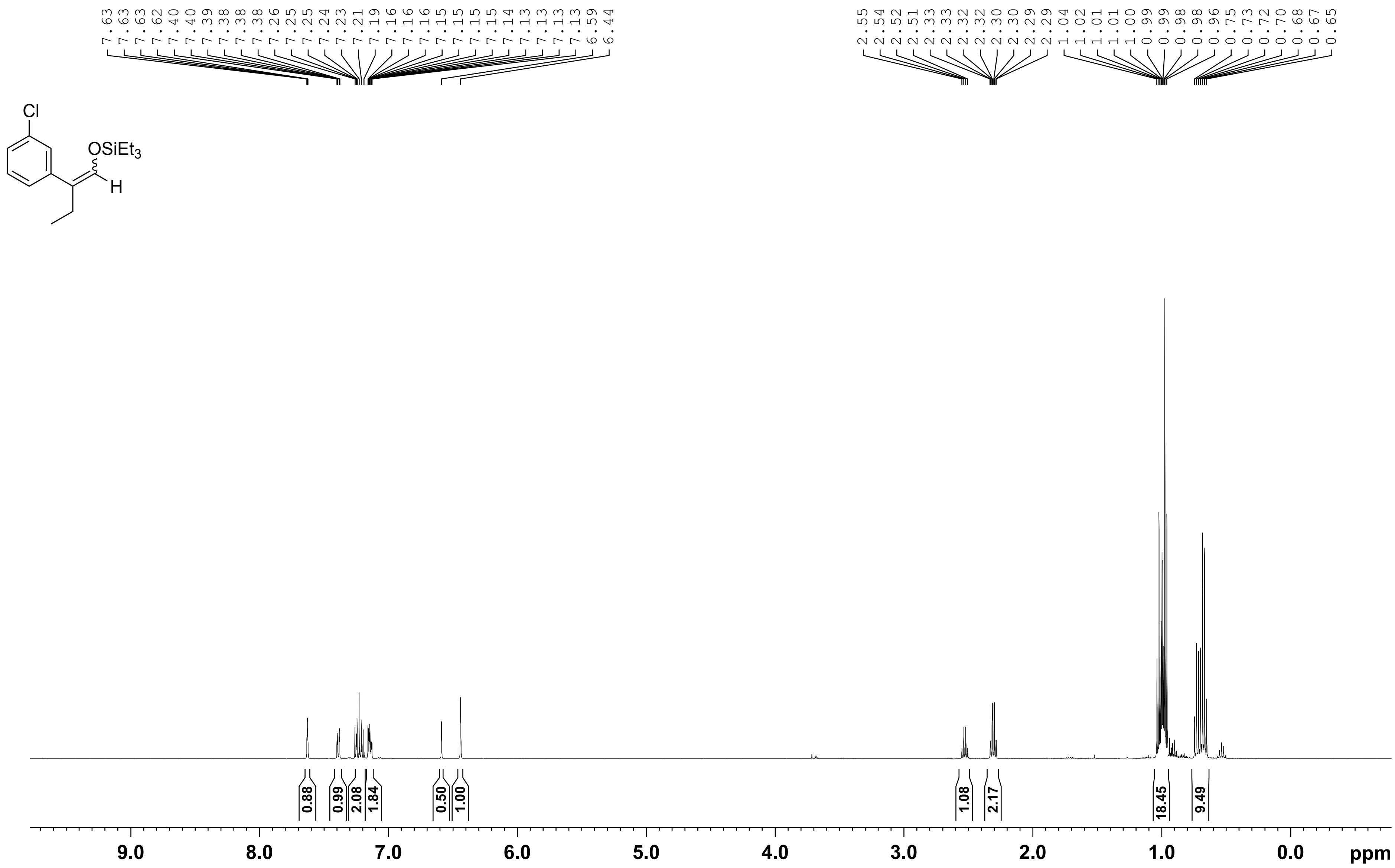

Figure S82. $^{13}C\{^{1}H\}$ NMR spectrum (126 MHz, $CDCl_3$, 298 K) of **3oa** (*Z*:*E* = 67:33).

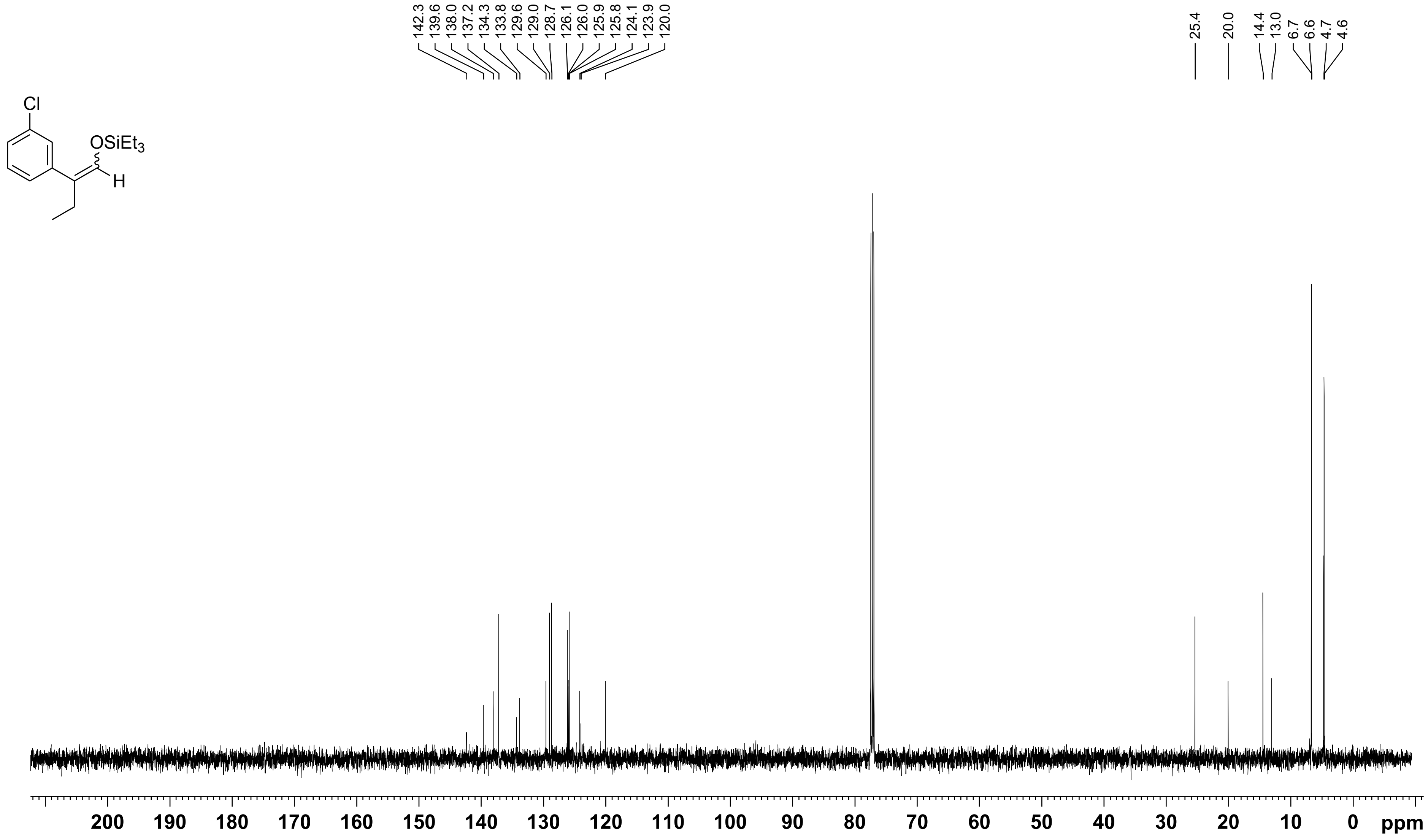

Figure S83. ^{29}Si DEPT NMR spectrum (99 MHz, $CDCl_3$, 298 K, optimized for *J* = 7 Hz) of **3oa** (*Z*:*E* = 67:33).

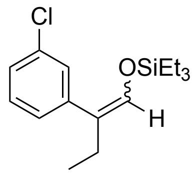

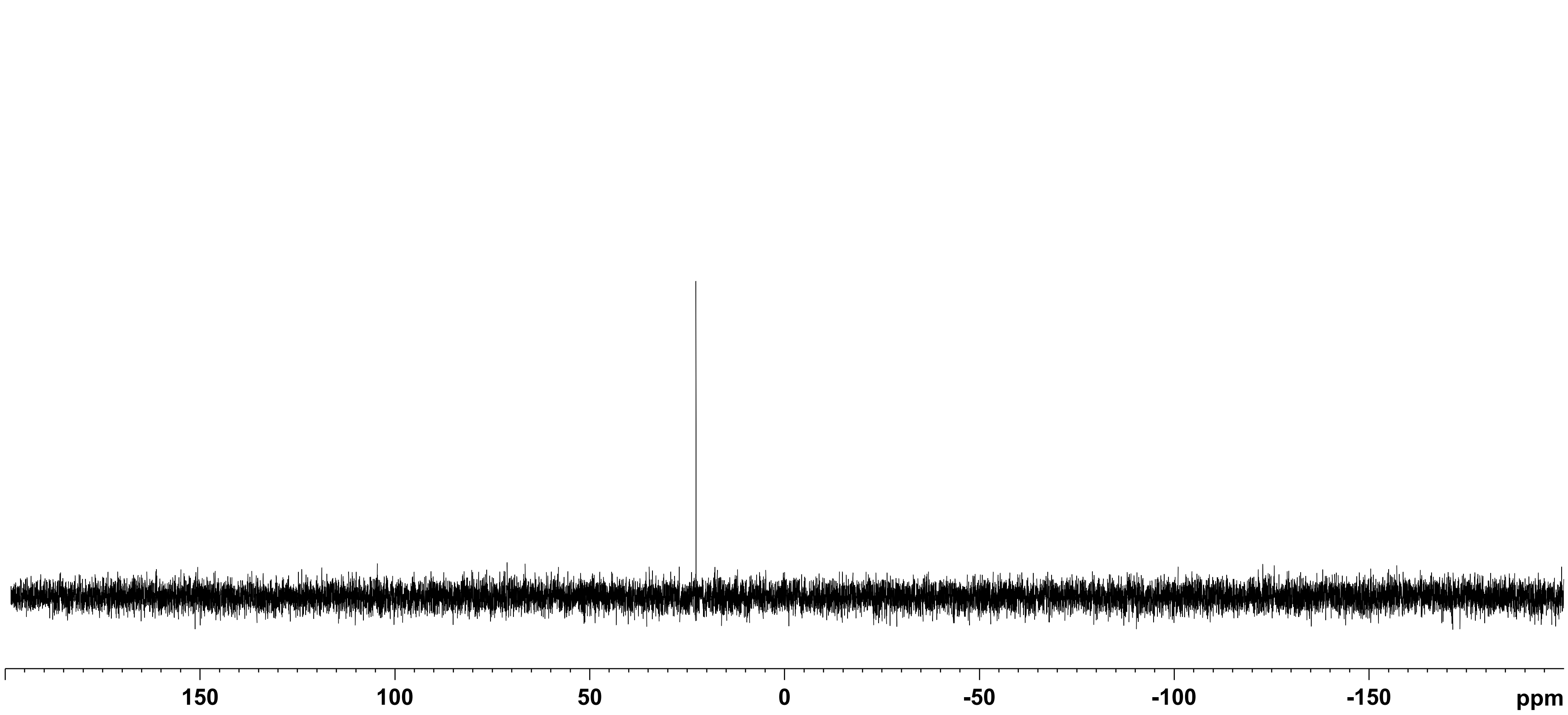

Figure S84. ^{1}H NMR spectrum (500 MHz, $CDCl_3$, 298 K) of **3pa** (*Z*:*E* = 77:23).

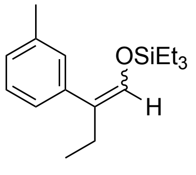

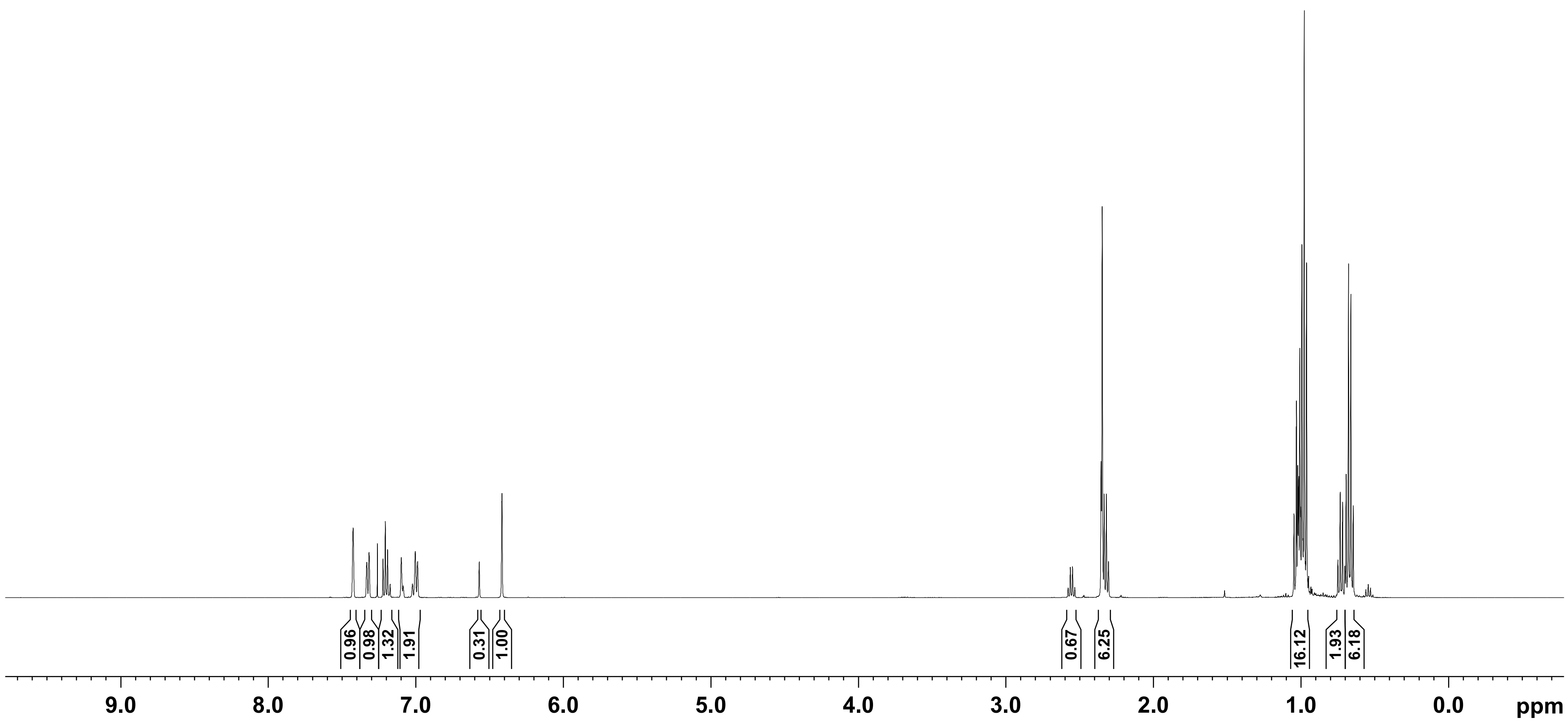

Figure S85. $^{13}C\{^1H\}$ NMR spectrum (126 MHz, $CDCl_3$, 298 K) of **3pa** (*Z*:*E* = 77:23).

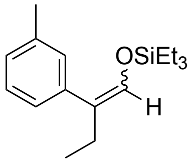

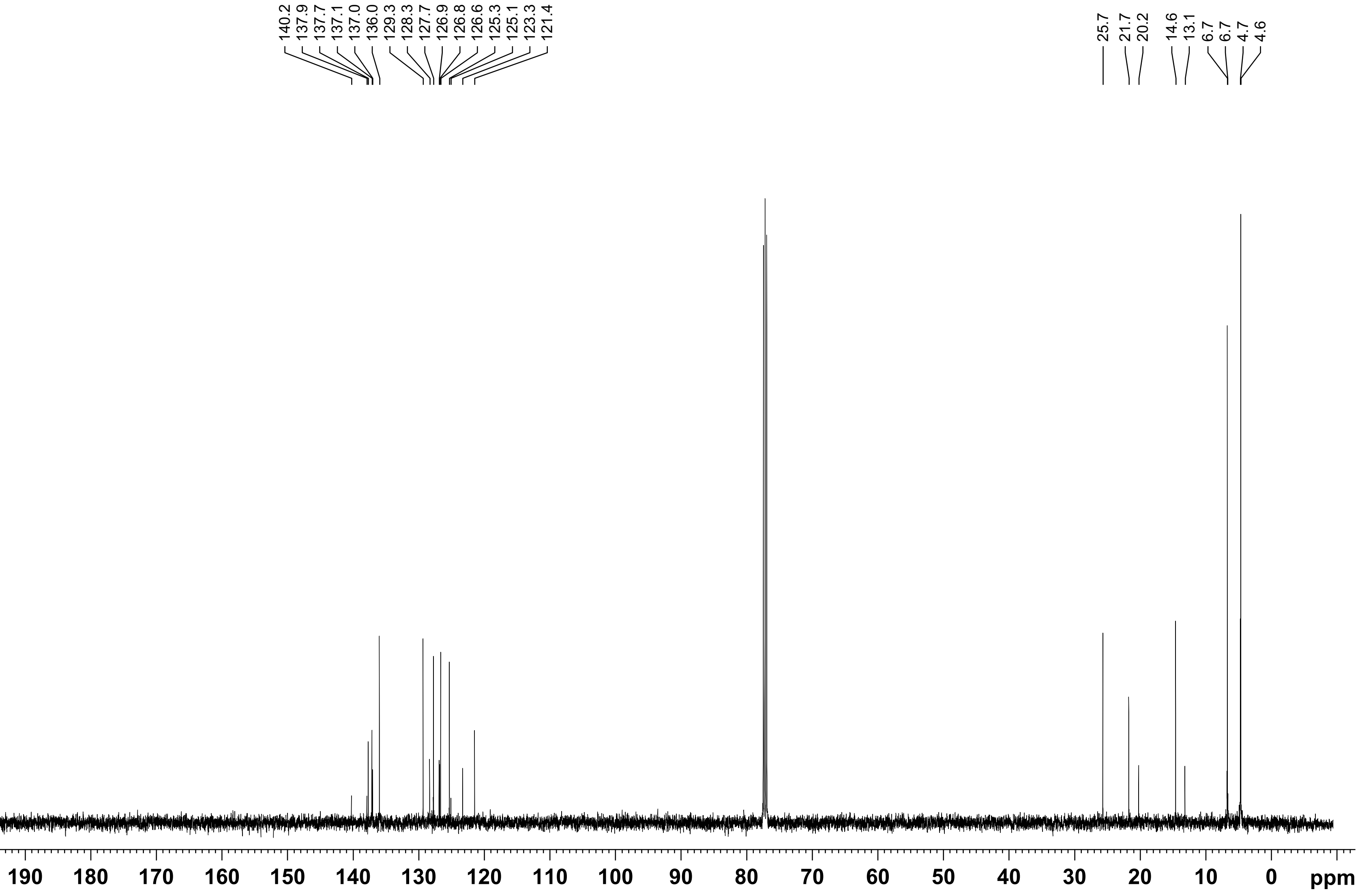

Figure S86. ^{29}Si DEPT NMR spectrum (99 MHz, $CDCl_3$, 298 K, optimized for *J* = 7 Hz) of **3pa** (*Z*:*E* = 77:23).

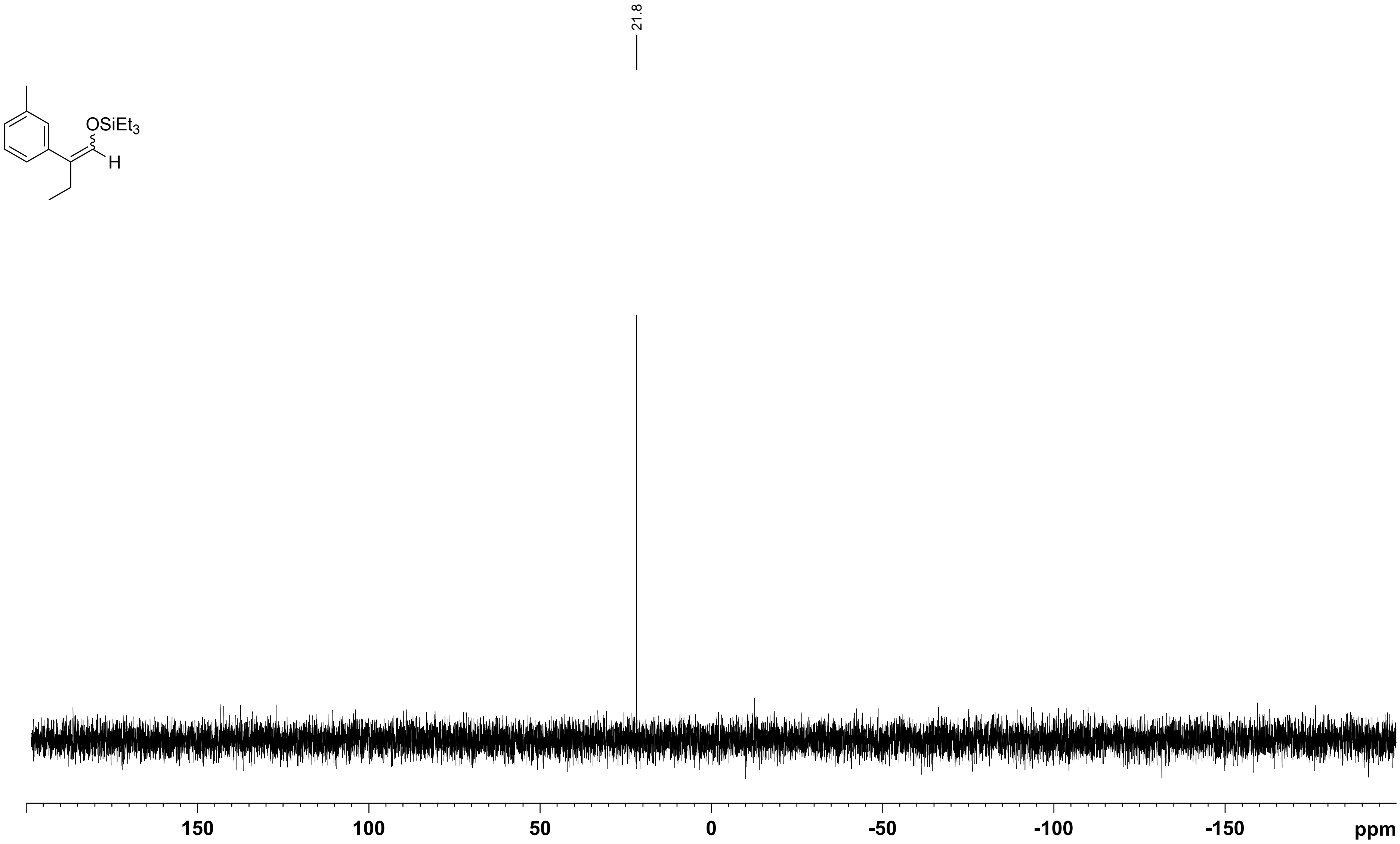

Figure S87. ^{1}H NMR spectrum (500 MHz, $CDCl_3$, 298 K) of **3qa** (*Z*:*E* = 84:16).

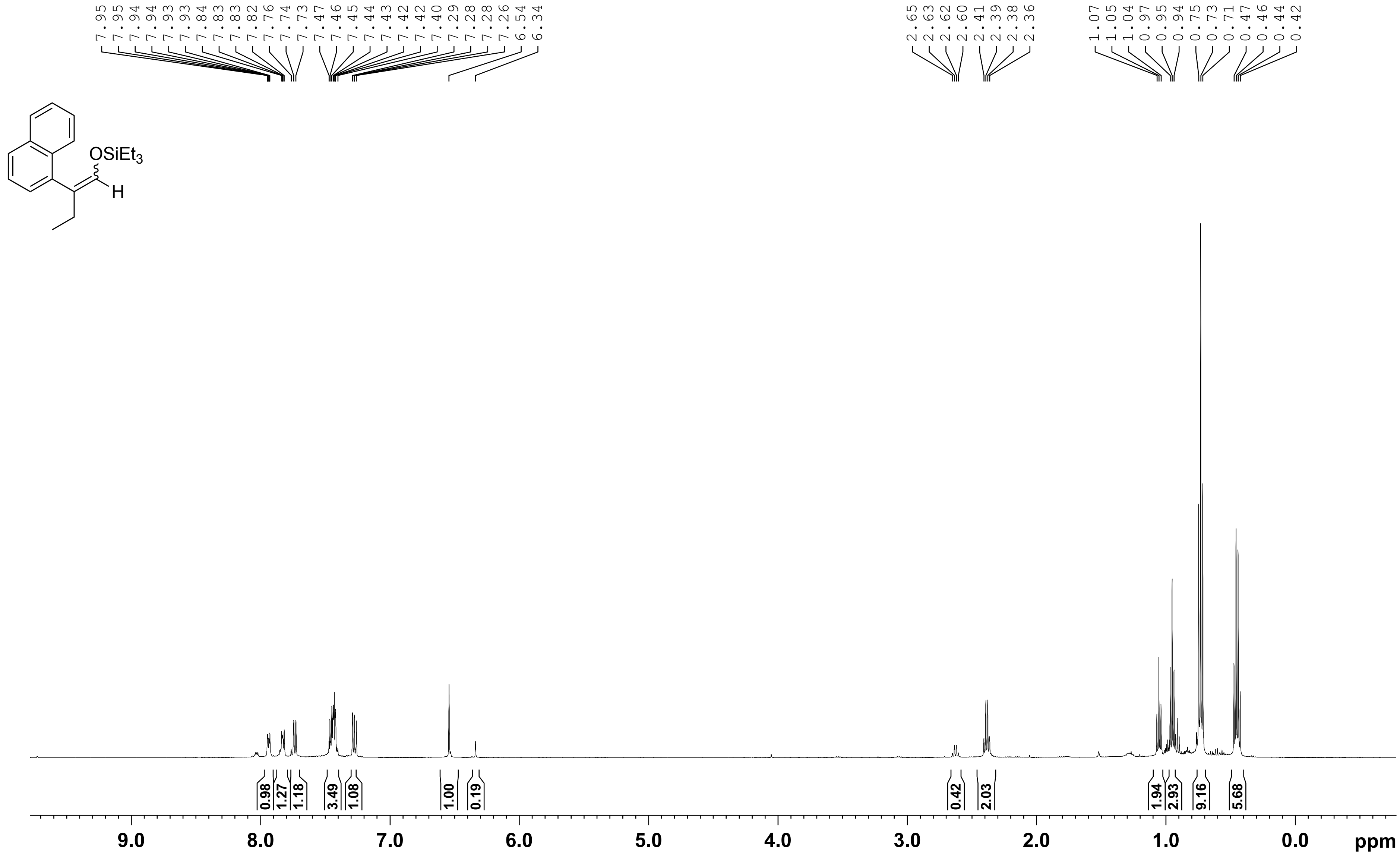

Figure S88. $^{13}C\{^1H\}$ NMR spectrum (126 MHz, $CDCl_3$, 298 K) of **3qa** (*Z*:*E* = 84:16).

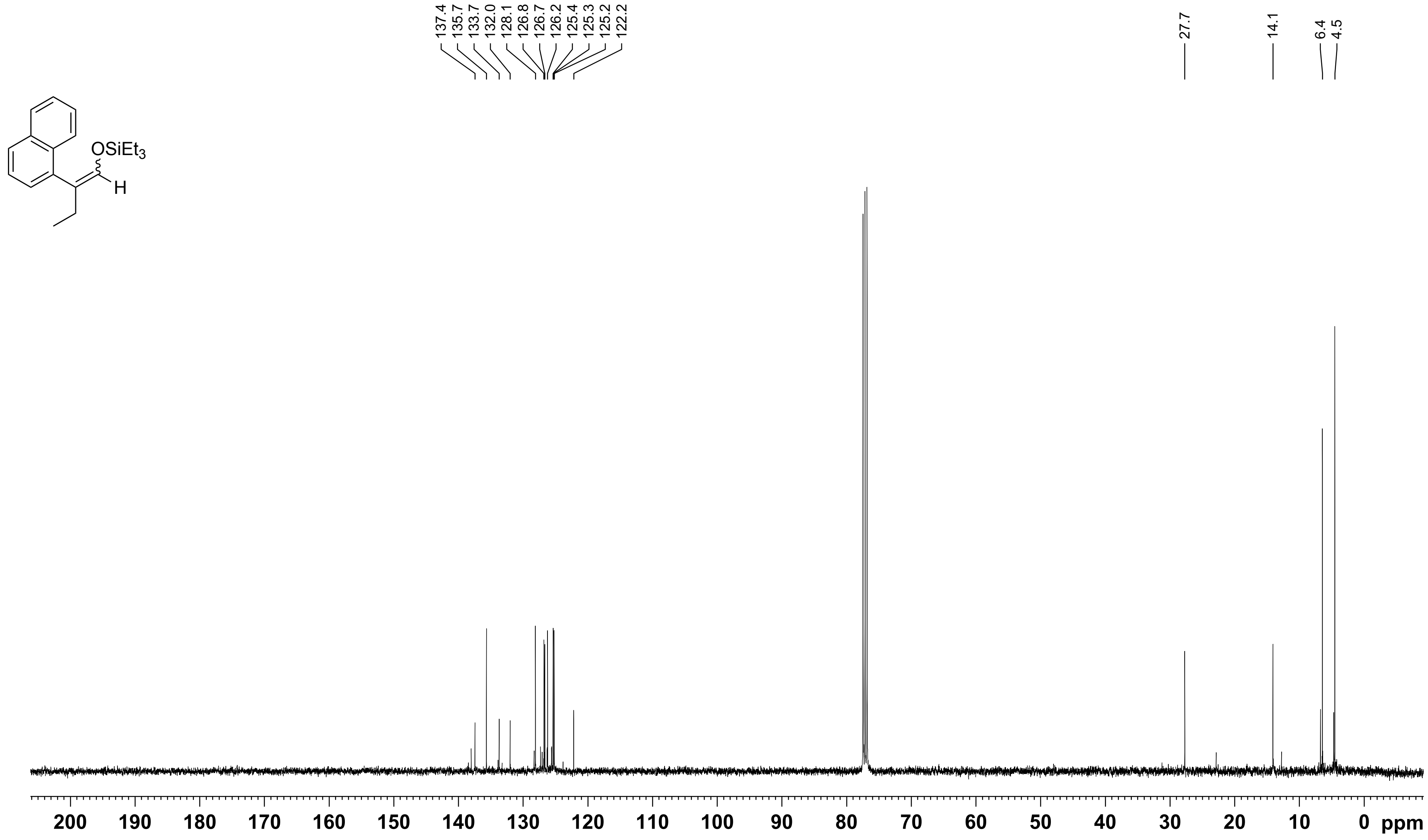

Figure S89. ^{29}Si DEPT NMR spectrum (99 MHz, $CDCl_3$, 298 K, optimized for J = 7 Hz) of **3qa** (Z:E = 84:16).

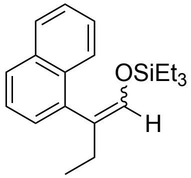

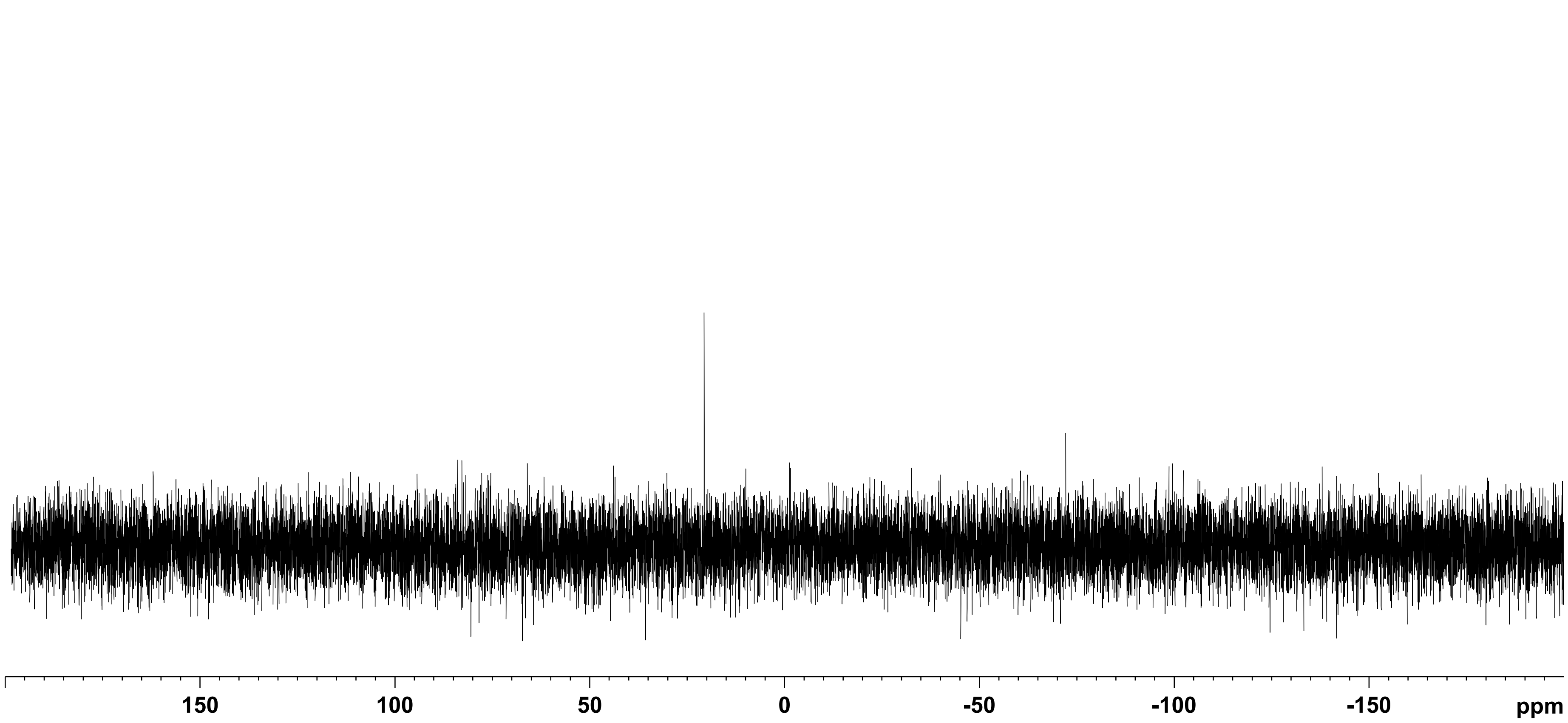

Figure S90. ^{1}H NMR spectrum (500 MHz, $CDCl_3$, 298 K) of **4af**. (* = aldehyde derived from partial decomposition, # = silyl ethers derived from trace Et_2SiH_2)

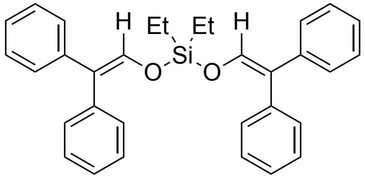

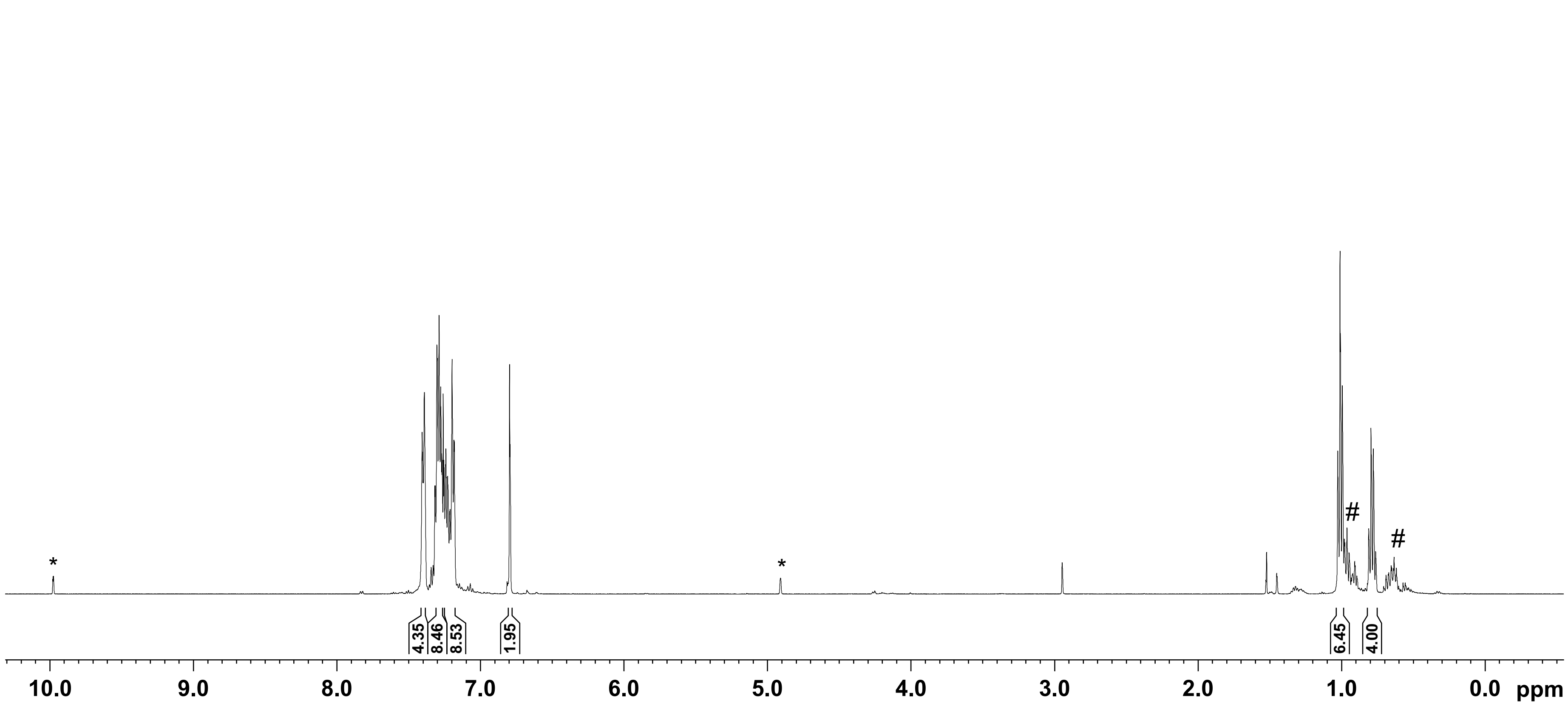

Figure S91. $^{13}C\{^{1}H\}$ NMR spectrum (126 MHz, $CDCl_3$, 298 K) of **4af**. (* = aldehyde derived from partial decomposition, # = silyl ethers derived from trace Et_2SiH_2)

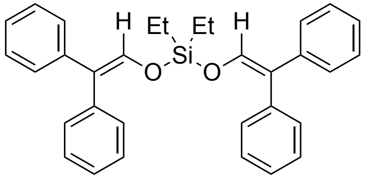

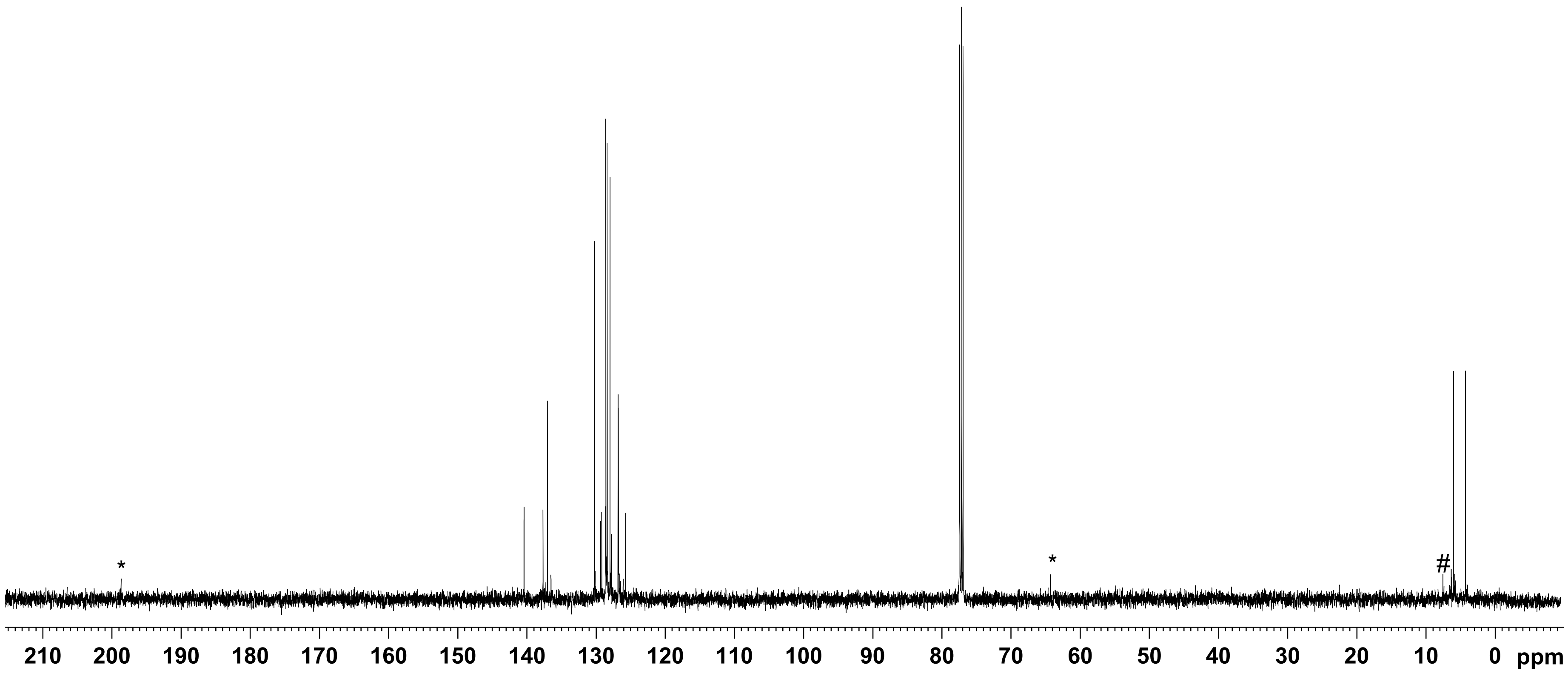

Figure S92. ^{29}Si DEPT NMR spectrum (99 MHz, $CDCl_3$, 298 K, optimized for J = 7 Hz) of **4af**.

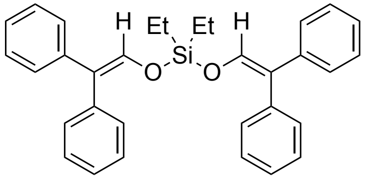

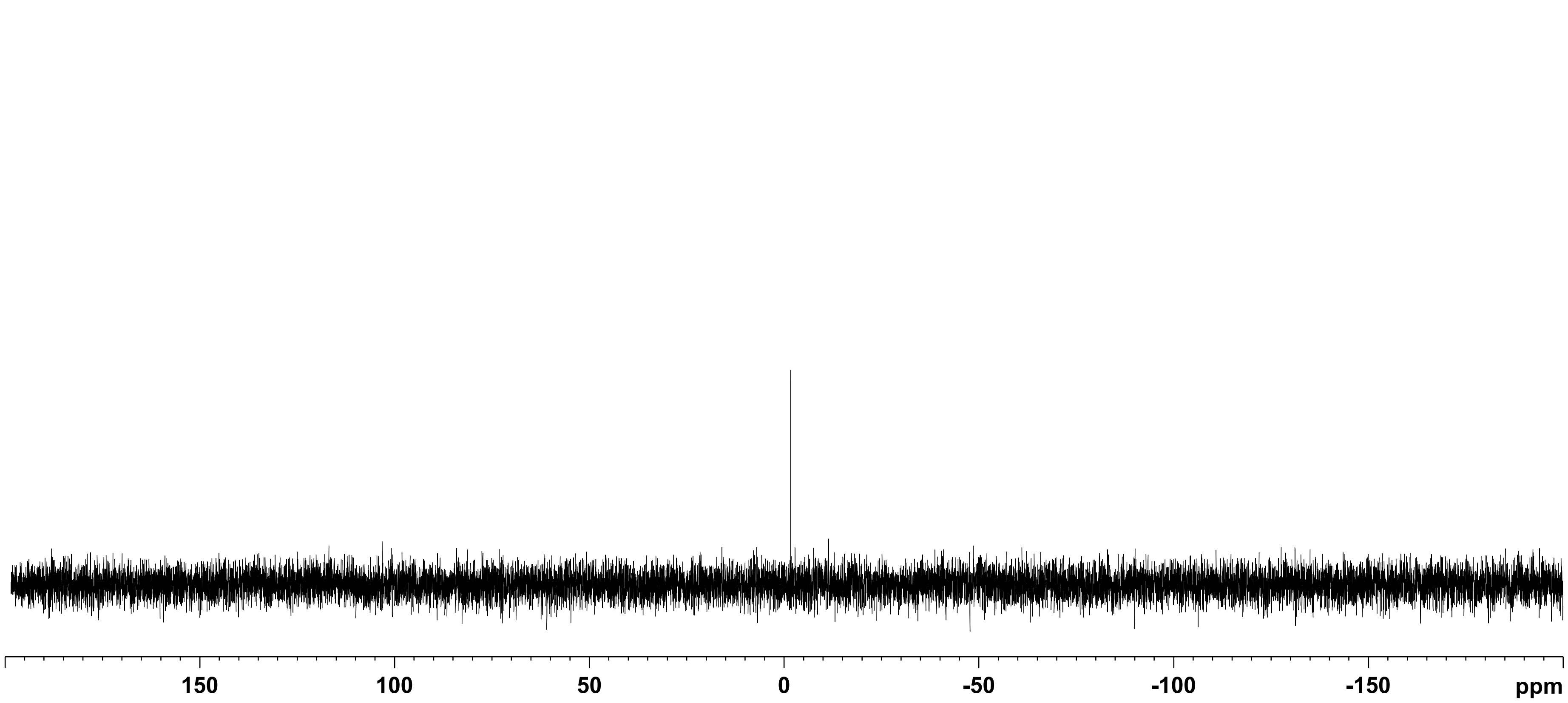

Figure S93. ^{1}H NMR spectrum (500 MHz, $CDCl_3$, 298 K) of **4cf**. (* = silyl ethers derived from trace Et_2SiH_2)

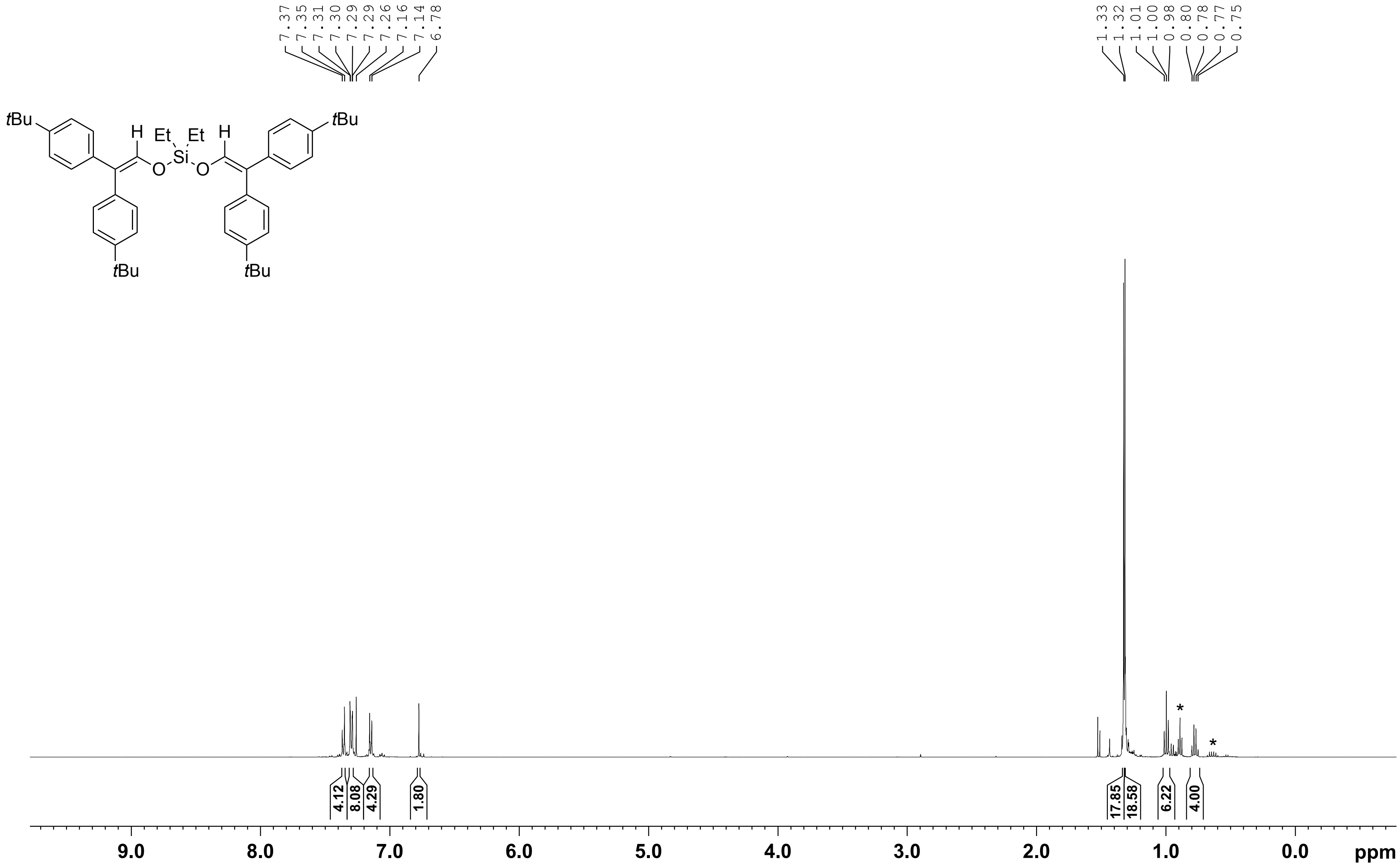

Figure S94. $^{13}C\{^1H\}$ NMR spectrum (126 MHz, $CDCl_3$, 298 K) of **4cf**.

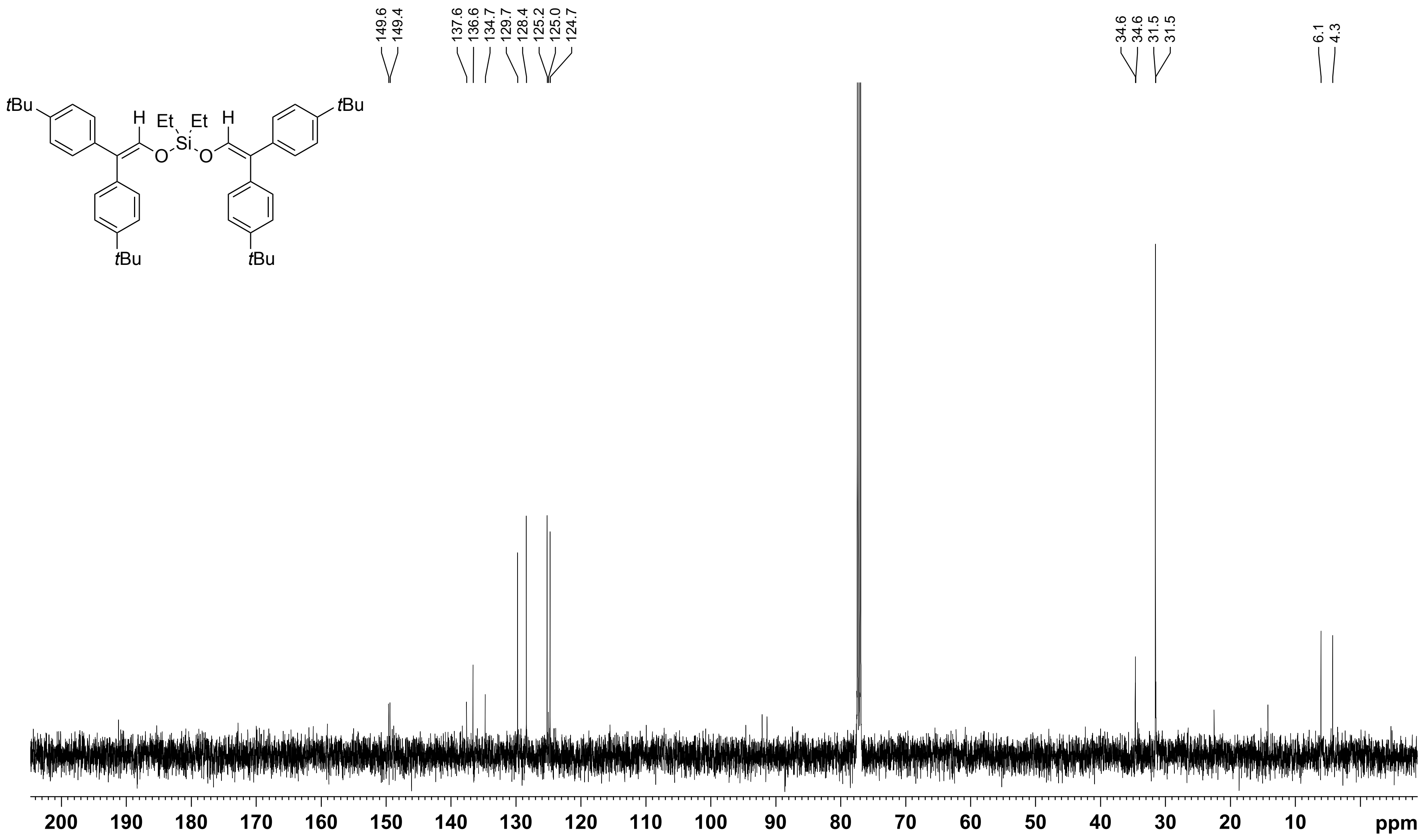

Figure S95. ^{29}Si DEPT NMR spectrum (99 MHz, $CDCl_3$, 298 K, optimized for J = 7 Hz) of **4cf**.

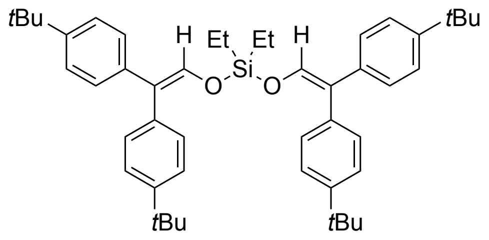

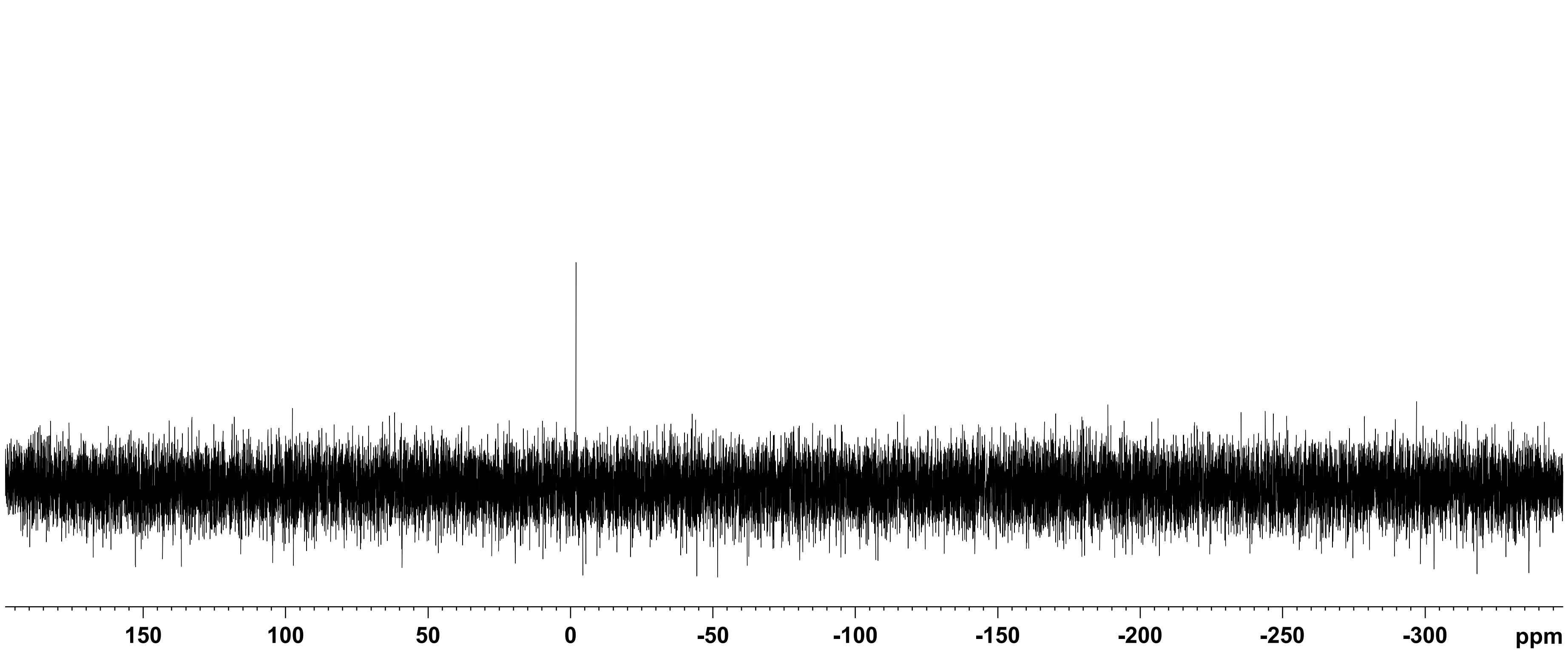

10 References

[1] R. K. Harris, E. D. Becker, S. M. Cabral de Menezes, R. Goodfellow, P. Granger, *Solid State Nucl. Magn. Reson.* **2002**, *22*, 458–483.

[2] R. Csuk, A. Barthel, D. Ströhl, *Z. Naturforsch.* **2011**, *66b*, 95–97.

[3] S.-C. Sha, J. Zhang, P. J. Walsh, *Org. Lett.* **2015**, *17*, 410–413.

[4] D. L. Moore, A. E. Denton, R. M. Kohinke, B. R. Craig, W. E. Brenzovich, *Synth. Commun.* **2016**, *46*, 604–612.

[5] J. M. Goll, E. Fillion, *Organometallics* **2008**, *27*, 3622–3625.

[6] W. W. Huang, H. Henry-Riyad, T. T. Tidwell, *J. Am. Chem. Soc.* **1999**, *121*, 3939–3943.

[7] E. V Dehmlow, M. Slopianka, J. Pickardt, *Liebigs Ann. Chem.* **1979**, *1979*, 572–593.

[8] T.-Y. Jian, L. He, C. Tang, S. Ye, *Angew. Chem. Int. Ed.* **2011**, *50*, 9104–9107; *Angew. Chem.* **2011**, *123*, 9270–9273.

[9] M. Dochnahl, G. C. Fu, *Angew. Chem. Int. Ed.* **2009**, *48*, 2391–2393; *Angew. Chem.* **2009**, *121*, 2427–2429.

[10] J. Douglas, J. E. Taylor, G. Churchill, A. M. Z. Slawin, A. D. Smith, *J. Org. Chem.* **2013**, *78*, 3925–3938.

[11] T. Cruchter, M. G. Medvedev, X. Shen, T. Mietke, K. Harms, M. Marsch, E. Meggers, *ACS Catal.* **2017**, *7*, 5151–5162.
